# Davidones F and G, Two Novel Flavonoids from *Sophora davidii* (Franch.) Skeels

**DOI:** 10.3390/molecules26144182

**Published:** 2021-07-09

**Authors:** Ping Song, Xuecui Li, Tongxi Zhou, Yu Peng, Ho-Young Choi, Yuanren Ma, Xinzhou Yang

**Affiliations:** 1 School of Chemistry and Chemical Engineering, Qinghai Nationalities University, Xining 810007, China; spzhe@126.com (P.S.); 18697145746@139.com (X.L.); 2 School of Pharmaceutical Sciences, South-Central University for Nationalities, Wuhan 430074, China; tc13627123095@163.com (T.Z.); PengY1020@163.com (Y.P.); 3 College of Korean Medicine, Kyung Hee University, Seoul 02447, Korea; hychoi@khu.ac.kr

**Keywords:** *Sophora davidii* (Franch.) Skeels, flavonoids, electronic circular dichroism (ECD), glucose transporter 4 (GLUT-4)

## Abstract

An unprecedented novel flavanone davidone F (**1**) with a seven-membered ring side chain, and a novel flavanonol davidone G (**2**), along with 11 known flavonoids, were isolated from the ethyl acetate fraction of *Sophora davidii* (Franch.) Skeels. Their planar structures were established by UV, IR, HRESIMS, 1D and 2D NMR data. The relative configurations of **1** and **2** were determined by calculation of NMR chemical shift values, the absolute configuration of **1** and **2** were assigned by comparing their experimental and calculated electronic circular dichroism (ECD) spectra. Moreover, compounds **1**–**13** were screened for the translocation activity of glucose transporter 4 (GLUT-4), and the fluorescence intensity was increased to the range of 1.56 and 2.79 folds. Compounds **1** and **2** showed moderate GLUT-4 translocation activity with 1.64 and 1.79 folds enhancement, respectively, at a concentration of 20 μg/mL.

## 1. Introduction

**Figure 1:**
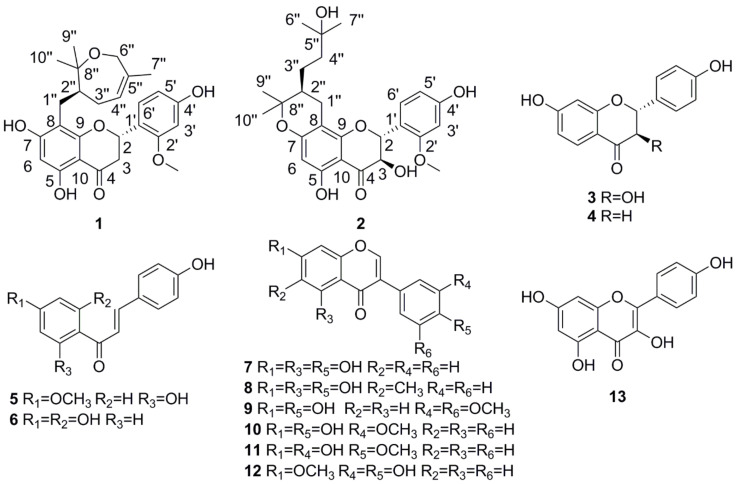
The structures of compounds **1**–**13**.

*Sophora davidii* (Franch.) Skeels are shrubs or dungarungas, which belong to the family Fabaceae, mainly distributed in Guizhou, Ningxia, Yunnan and Sichuan provinces of China [1]. As a folk herbal medicine, this plant is grown in hillsides, roadsides and bushes at an altitude of 1300–2500 m, and its roots, leaves, flowers and fruits are traditionally used to treat diarrhea, cystitis, stomachache, edema and sarcoptic mange [2]. The plant has proven to be a rich source of flavonoids, alkaloids, steroids, lignans and phenolic acids [3,4,5]. Flavonoids, as the main compounds of *S. davidii,* have a broad spectrum of biological activities. Notably, for the prenylated flavonoids, prenylation increased the lipophilicity of flavonoids, which resulted in an increased affinity to biological receptors [6]. To enhance our understanding of the chemical and biological diversity of the *Sophora* species, the chemical investigation of *S. davidii* led to the isolation of one new prenylated flavanone, davidone F (**1**), with a novel seven-membered oxygen ring, and one new prenylated flavanonol, davidone G (**2**), together with one known flavanonol (**3**), two known chalcones (**5**–**6**), four known isoflavones (**7**–**12**) and one known flavonol (**13**) (Figure 1). Additionally, we describe the isolation, structure elucidation and GLUT-4 translocation activities of these isolated compounds.

## 2. Results and Discussion

**Figure 2:**
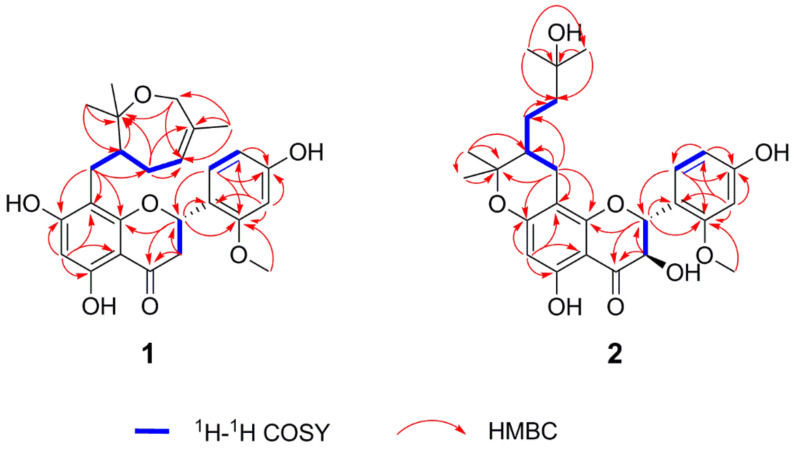
Key ^1^H–^1^H COSY (**─**) and HMBC (→) correlations of **1**–**2**.

**Figure 3:**
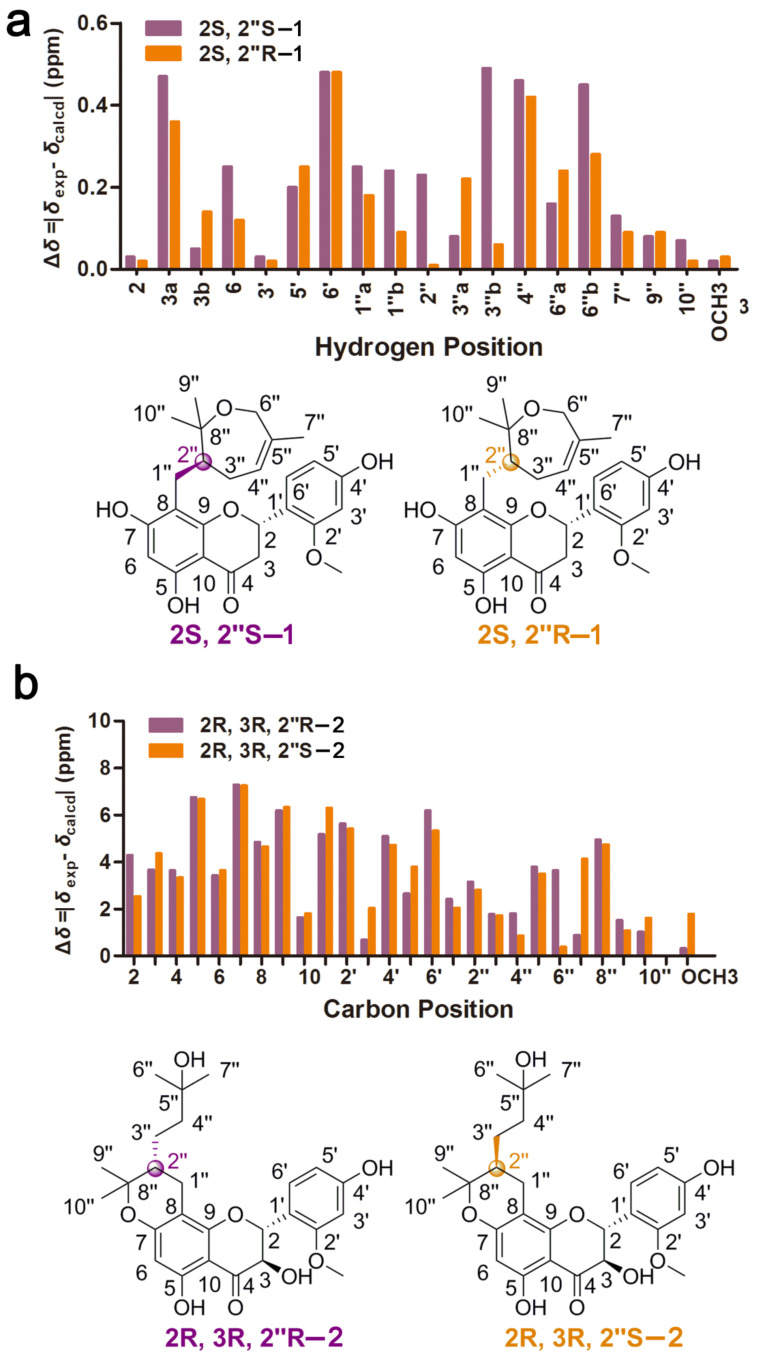
(**a**) Differences between calculated and experimental ^1^H NMR chemical shifts for 2S, 2″S-**1** and 2S, 2″R–**1** (**b**) Differences between calculated and experimental ^13^C NMR chemical shifts for 2R, 3R, 2″R–**2** and 2R, 3R, 2″S–**2**.

**Figure 4:**
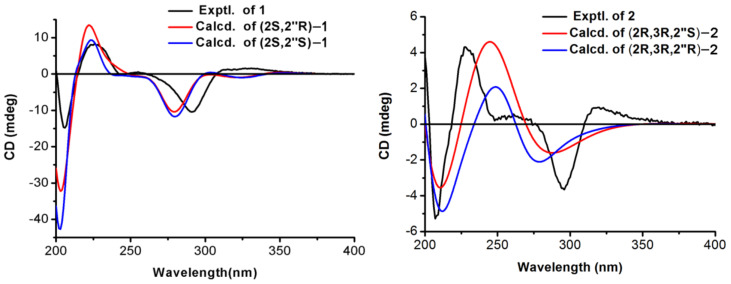
Experimental and calculated ECD spectra of compounds **1** and **2**.

Compound **1** was isolated as a brown oil. The molecular formula was established as C_26_H_30_O_7_ by HRESIMS data (*m*/*z* 455.2061 [M + H]^+^, calculated for 455.2064), indicating 12 indices of hydrogen deficiency. The IR spectrum revealed the presence of characteristic hydroxy (3244 cm^−1^) and carbonyl (1636 cm^−1^) absorptions. Its ^1^H NMR data (Table 1) indicated characteristic resonances for one 1,2,4-trisubstituted phenyl ring system [*δ*_H_ 7.32 (1H, d, *J* = 8.3 Hz, H-6′), 6.48 (1H, d, *J* = 2.2 Hz, H-3′), 6.43 (1H, dd, *J* = 8.3, 2.2 Hz, H-5′)] and one pentasubstituted phenyl ring system [*δ*_H_ 5.94 (1H, s, H-6)]. The presence of three one-proton double doublets [*δ*_H_ 5.61 (1H, dd, *J* = 13.1, 3.0 Hz, H-2), *δ*_H_ 3.13 (1H, dd, *J* = 17.1, 13.1 Hz, H-3), *δ*_H_ 2.66 (1H, dd, *J* = 17.1, 3.0 Hz, H-3)] confirmed the structure of a flavanone skeleton [7]. Several aliphatic resonances emerged in the high-field part: three methyls at *δ*_H_ 1.52 (3H, s, H-7″), 1.18 (3H, s, H-9″) and 1.08 (3H, s, H-10″); one oxygenated methylene at *δ*_H_ 4.26 (1H, d, *J* = 16.7 Hz, H-6″) and *δ*_H_ 3.68 (1H, d, *J* = 16.7 Hz, H-6″); two methylenes at *δ*_H_ 2.40 (1H, dd, *J* = 12.7, 10.7 Hz, H-1″), *δ*_H_ 2.20 (1H, overlap, H-1″), *δ*_H_ 2.18 (1H, m, H-3″) and *δ*_H_ 1.75 (1H, m, H-3″) and one methane at *δ*_H_ 2.20 (1H, m, H-2″). Additionally, the presence of one vinyl group and one methoxy group was confirmed by signals at *δ*_H_ 5.29 (1H, d, *J* = 6.6 Hz, H-4″) and *δ*_H_ 3.81 (3H, s, 2′-OCH_3_). The ^13^C NMR spectrum of **1** displayed 26 carbon signals, including three methyls, one methoxy group, four methylenes, seven methines and 11 non-protonated carbons. The pendant aromatic B-ring suggested a 1,2,4-trisubstituted arrangement, due to the HMBC correlations of H-6′ and H-3′ with C-2 (*δ*_C_ 75.5), C-3′ (*δ*_C_ 99.8), C-2′ (*δ*_C_ 159.3), C-4′ (*δ*_C_ 160.5) and C-5′ (*δ*_C_ 108.0), C-1′ (*δ*_C_ 119.2), respectively. Meanwhile, the pentasubstituted phenyl ring system assigned to the A-ring was determined by HMBC correlations between H-6 and C-10 (*δ*_C_ 103.3), C-8 (*δ*_C_ 108.2), C-9 (*δ*_C_ 162.5), C-5 (*δ*_C_ 163.3), C-7 (*δ*_C_ 166.3) and C-4 (*δ*_C_ 198.7). The methoxy group located on C-2′ was established by HMBC correlation from H_3_-2′-OCH_3_ to C-3′ (*δ*_C_ 99.8) and C-2′ (*δ*_C_ 159.3). Besides 15 skeletal carbon atoms of flavanone and the methoxy moiety, the remaining 10 carbons were connected to form a substituted seven-membered ring from the detailed analysis of the COSY and HMBC correlations. Through interpretation of the COSY correlations between H-1″/H-2″/H-3″/H-4″ (Figure 2), it was possible to establish the presence of a CH_2_-CH-CH_2_-CH=C spin system. Combining with the HMBC cross-peaks (Figure 2) from H_3_-7″ to C-6″ (*δ*_C_ 65.7), C-4″ (*δ*_C_ 126.6) and C-5″ (*δ*_C_ 136.8), from H_2_-6″ to C-7″ (*δ*_C_ 20.9) and C-8″ (*δ*_C_ 80.3) and from H_3_-9″ or H_3_-10″ to C-2″ (*δ*_C_ 48.9) and C-8” (*δ*_C_ 80.3), allowed the construction of a seven-membered ring. The HMBC correlation between H_2_-1″ and C-8 (*δ*_C_ 108.2), C-8 (*δ*_C_ 162.5) and C-9 (*δ*_C_ 166.5) suggested the seven-membered ring containing oxygen in the ring was connected to the A-ring. Thus, the planar structure of compound **1** was determined as shown. In order to further verify the relative configuration of **1**, we use the DP4 analysis to solve the stereochemistry problems [8]. NMR chemical shift (GIAO) was calculated at the mPW1PW91/6-311G(d,p) level of theory as required for DP4 analysis [9]. Importantly, the calculated DP4 probability of isomer 2S*, 2″R*-1 was assigned to 100% (Figure S21). Therefore, the relative configuration of **1** was defined as 2S*, 2″R* (Figure 3). To ascertain the absolute configuration of **1**, its ECD spectrum was determined in MeOH and then comparing the experimental and time-dependent density functional theory (TDDFT)-calculated electronic circular dichroism (ECD) spectra. The experimental ECD data of **1** showed positive CEs at 331 nm due to n → π* transitions of the carbonyl group (Figure 4) [10,11]. Based on the above evidence, the absolute configuration of **1** was elucidated as 2S, 2″R, and named davidone F.

Compound **2**, light yellow powder, was identified as a new natural flavanonol compound. Its molecular formula was assigned to C_26_H_32_O_8_ (11 indices of hydrogen deficiency), on the basis of the HRESIMS sodium adduct ion [M + Na]^+^ at *m*/*z* 495.1990 (calculated for 495.1989). The NMR data (Table 1) showed signals with splitting patterns similar to davidone B [12], a flavanone previously isolated from *S. davidii*, except for an oxygenated aliphatic carbon C-3 (*δ*_C_ 72.5), where an additional hydroxy group moiety was attached. The location of the additional hydroxy group substituent was established by the COSY relationship between H-2 and H-3 as well as the HMBC correlations (Figure 2) from H-3 to C-2 (*δ*_C_ 79.8), C-1′ (*δ*_C_ 116.9) and C-4 (*δ*_C_ 199.3), from H-2 to C-3 (*δ*_C_ 72.5), C-1′ (*δ*_C_ 116.9), C-6′ (*δ*_C_ 130.9), C-9 (*δ*_C_ 161.6) and C-4 (*δ*_C_ 199.3). According to the *J*-based configuration analysis protocol, the large coupling constant (11.6 Hz) observed between H-2 and H-3 indicated that the two hydrogens tended to adopt an “*anti*” arrangement. The ECD spectrum of **2** contained the characteristic CEs induced by C-4 ketone carbonyl in its flavanonol framework, namely, a positive one at λ_max_ 320 nm (Figure 4). The absolute configuration of C-2 and C-3 was thus proposed to be 2R, 3R [11,13]. Subsequently, we performed computational predictions of NMR chemical shifts of both the possible isomers 2R, 3R, 2″R and 2R, 3R, 2″S (Figure 3) using the GIAO method at the mPW1PW91/6-311G(d,p) level with the conductor polarizable calculation model (CPCM) in MeOH [8]. As a result, DP4 probability analysis showed that 2R, 3R, 2″S was predicted to be correct with probabilities of 99.56% for the NMR data (Figure S22). The calculated ECD spectrum of 2R, 3R, 2″S-2 at the B3LYP/6-311+G(d,p) level with the CPCM in MeOH fit well with the experimental spectrum of **2** (Figure 4). Finally, the absolute configuration of **2** was determined as 2R, 3R, 2″S, and named davidone G.

Eleven known compounds were also isolated and identified as 3,4′,7-trihydroxyflavanone (**3**) [14], liquiritigenin (**4**) [15], 4′-methylisoliquiritigenin (**5**) [16], isoliquiritigenin (**6**) [17], genistein (**7**) [18], 6-methyl-genistein (**8**) [19], 7,4′-dihydroxy-3′,5′-dimethoxyisoflavone (**9**) [20], 3′-methoxy-daidzein (**10**) [21], calycosin (**11**) [22], 7-methoxy-daidzein (**12**) [23] and kaempferol (**13**) [24] by comparison between spectroscopic data and physicochemical properties with those reported values in the literature. 

**Figure 5:**
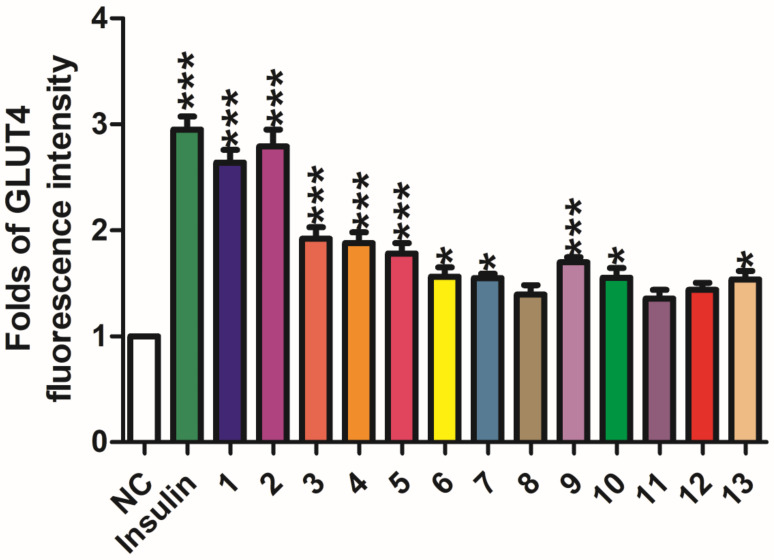
Effects of compounds **1**–**6** on stimulating GLUT4 translocation in L6 cells. (* *p* < 0.05, *** *p* < 0.001, compared with non-treated groups).

The potential GLUT-4 translocation activity of compounds **1**–**13** was tested against pIRAP-mOrange cDNAs transfected L6 cells (Figure 5), and insulin (100 nM) was used as the positive control [25]. The enhancing fluorescence intensity caused by compounds **1**–**13** was increased to the range of 1.56 and 2.79 folds. Compounds **1** and **2** showed moderate GLUT-4 translocation activity with 1.64 and 1.79 folds enhancement, respectively, at a concentration of 20 μg/mL. Compounds **3**–**13** exhibited inactive to weak activities, increasing GLUT-4 translocation by 0.56–0.88 folds, respectively. Initial examination of the structure–activity relationship inferred that the prenylated groups in compounds **1** and **2** might contribute to their enhanced GLUT-4 translocation activity [6,12,25,26,27].

## 3. Materials and Methods

### 3.1. General Information

Semi-preparative HPLC was carried out on a Waters 2535 HPLC fitted with a 2998 photodiode array Detector and a 2707 autosampler (Waters). Separations were performed on two Thermo C18 columns (5 μm, 10 × 150 mm; 5 μm, 20 × 150 mm) (Thermo, Waltham, MA, USA) and one Phenomenex column (5 μm, 10 × 150 mm) (Phenomenex, Torrance, CA, USA). Direct injection ESIMS and LC-PDA-ESIMS analyses were recorded on a Waters ACQUITY SQD MS system (Waters, Milford, MA, USA) connected to a Waters 1525 HPLC with a 2998 photodiode array Detector (Waters, Milford, MA, USA). The NMR spectra were recorded on an AVANCE III 600 MHz spectrometer (Bruker BioSpin, Ettlingen, Germany). Optical rotations were recorded on a Rudolph Research Analytical Autopol IV Automatic Polarimeter. UV and IR spectra were recorded on a UH5300 spectrophotometer (Hitachi, Tokyo, Japan) and a Nicolet Magna FT-IR 750 spectrometer (Nicolet, Madison, WI, USA), respectively.

### 3.2. Materials

The roots of *S. davidii* (Franch.) Skeels (age 12–15 years) were collected from Xiuwen county, Guizhou province, China (at altitudes of 1200 to 1300 m) in June 2014. The roots were dried at room temperature, macerated into a fine powder and stored at room temperature. The identification was performed by Professor Dingrong Wan of School of Pharmaceutical Sciences, South-Central University for Nationalities (SCUN), Wuhan, China. A voucher specimen (SC0801) is deposited at the School of Pharmaceutical Sciences, SCUN, Wuhan, China.

### 3.3. Extraction and Isolation

The dried roots of the plant (18 kg) were milled and then extracted with 80% EtOH (4 × 20 L, 3 days each) at room temperature yield 850 g of crude EtOH extract. Subsequently, the EtOH fraction was suspended in H_2_O and partitioned with petroleum ether (PE) (4 × 10 L), ethyl acetate (EtOAc) (4 × 10 L) and n-butyl alcohol (n-BuOH) (4 × 10 L) to give a PE extract (90 g), EtOAc extract (215 g) and n-BuOH extract (110 g), respectively. The EtOAc part (200 g) was subjected to a silica-gel column chromatography (300–400 mesh) eluting with a gradient solvent system of CH_2_Cl_2_/MeOH (200:1 to 0:1, *v*/*v*) to yield 16 fractions (FB1–FB16). Fraction FB8 (5.6 g) was separated on a silica-gel column chromatography (silicone H), using CH_2_Cl_2_/MeOH (100:1 to 0:1, *v*/*v*) as a mobile phase, to obtain seven fractions (FB8-1–FB8-7). Fraction FB8-3 (165.0 mg) was further separated on semi-preparative HPLC (MeCN/H_2_O, 35:65–70:30, 20 min) to afford compound **1** (5.3 mg). Purification of fraction FB10 (7.0 g) afforded eight subfractions, FB10-1–FB10-8, using silica-gel column chromatography (CH_2_Cl_2_/MeOH, 50:1 to 0:1, *v*/*v*). Fraction FB10-2 was purified by Sephadex LH-20 eluted with a mixture of MeOH–H_2_O (95%:5%) and was subjected to further purification via semi-preparative HPLC (MeCN/H_2_O, 10:90–100:0, 20 min) to yield compound **5** (8.0 mg) and compound **6** (8.1 mg). FB10-6 (900 mg) was fractionated by the Sephadex LH-20 with elution MeOH to give five subfractions, FB10-6-1–FB10-6-5. Compound **4** (2.1 mg) was obtained from FB10-6-1 (53.6 mg) by preparative HPLC (MeCN/H_2_O, 29:71–33:67, 20 min). Fraction FB11 (2.0 g) was separated by Sephadex LH-20 and eluted with 90% MeOH to give nine subfractions (FB11-1–FB11-9). FB11-1 (13.8 mg) was purified by semi-preparative HPLC (MeCN/H_2_O, 30:70–100:0, 20 min) to obtain **2** (1.6 mg). Fraction FB12 was purified by Sephadex LH-20 to yield subfraction FB12-2, which was separated using semi-preparative HPLC (MeCN/H_2_O, 10:90–45:55, 25 min) to obtain **3** (12.5 mg).

#### 3.3.1. Davidone F (**1**)

Brown oil; [α]^20^_D_ −86.4 (*c* 0.50, MeOH); UV λ_max_ (MeOH, nm) (log *ε*) 225 (3.04), 290 (2.36); IR *ν*_max_ (cm^−1^): 3244, 2972, 1636, 1458, 1166 and 1070; ECD (c 0.5, MeOH) λ_max_(Δ*ε*) 206 (−4.07) nm, 226 (2.30) nm, 291 (−2.86) nm, 331 (0.44) nm; ^1^H and ^13^C-NMR spectroscopic data can be found in Table 1; HRESIMS *m*/*z* 455.2061 [M + H]^+^ (calculated for C_26_H_31_O_7_: 455.2064).

#### 3.3.2. Davidone G (**2**)

Light yellow powder; [α]^20^_D_ +24.2 (*c* 0.50, MeOH); UV λ_max_ (MeOH, nm) (log *ε*) 215 (2.40), 295 (1.75); IR *ν*_max_ (cm^−1^): 3366, 2934, 1643, 1616, 1589, 1163 and 1123; ECD (c 0.5, MeOH) λ_max_(Δ*ε*) 207 (−1.51) nm, 227 (1.23) nm, 296 (−1.04) nm, 320 (0.27) nm; ^1^H and ^13^C-NMR spectroscopic data can be found in Table 1; HRESIMS *m*/*z* 495.1990 [M + Na]^+^ (calculated for C_26_H_32_O_8_Na: 495.1989).

### 3.4. Computation Details

Conformational searches were carried out via random searching in Sybyl-X 1.1.1 using the MMFF94S forcefield [28]. The conformers obtained in an energy window of 10 kcal·mol^−1^ were further applied to geometrical optimization and minimization using the B3LYP/6-31G(d,p) level in the gas phase in Gaussian 09. Subsequently, NMR chemical shift calculations were conducted using gauge-independent atomic orbitals (GIAO) method at the mPW1PW91/6-311G(d,p)/CPCM level in MeOH (**1** and **2**) [9]. The shielding constants were converted into unscaled chemical shifts (*δ*_u_) by referencing TMS (*δ*_u_ = *σ*_TMS_ − *σ*_Cal_), where the *σ*_TMS_ was the shielding constant of TMS calculated at the same level. The NMR chemical shifts of the isomers were obtained by Boltzmann averaging the ^1^H and ^13^C NMR chemical shifts of the stable conformers. The calculated NMR properties of optimized structures were averaged based upon their respective Boltzmann populations, and calculations of DP4+ probability analysis were facilitated by the Excel sheet (DP4+) provided by Grimblat et al. [8]. In the part of ECD calculation, the optimized conformers were calculated using the time-dependent density functional theory (TD-DFT) method at the B3LYP/6-311+G(d,p) level in MeOH. The ECD curves were simulated on the basis of rotatory strengths using SpecDis v1.71 with a half-band of 0.3 eV and averaged according to the Boltzmann distribution [29].

### 3.5. GLUT-4 Translocation Assay

Construction of myc-GLUT4-mOrange plasmid and cell line were performed as described previously. Myc-GLUT4-mOrange-L6 cells were cultured on glass coverslips for 12 h, and then L6 myoblasts were differentiated to L6 myotubes. Cells were starved in a PSS solution for 2 h. After starvation, mOrange fluorescence was detected by laser-scanning confocal microscopy at an excitation wavelength of 555 nm. Images were taken after addition of tested samples (10 μg/mL), insulin (10 nM) or normal control (NC) (0.1% DMSO) for 30 min using 555 nm excitation laser. Zen 2010 Software (Carl Zeiss, Jena, Germany) was used to analyze the fluorescence intensity of mOrange [30].

## 4. Conclusions

In conclusion, a phytochemical investigation of the dry roots of *S. davidii* yielded one unprecedented new prenylated flavanone, davidone F (**1**), with a novel seven-membered oxygen ring, one new prenylated flavanonol, davidone G (**2**), along with 11 known flavonoids: 3,4′,7-trihydroxyflavanone (**3**), liquiritigenin (**4**), 4′-methylisoliquiritigenin (**5**), isoliquiritigenin (**6**), genistein (**7**), 6-methyl-genistein (**8**), 7,4′-dihydroxy-3′,5′-dimethoxyisoflavone (**9**), 3′-methoxy-daidzein (**10**), calycosin (**11**), 7-methoxy-daidzein (**12**) and kaempferol (**13**). Their structures and absolute configurations were determined by HRESIMS, 1D/2D NMR, NMR and ECD calculations. Davidone F (**1**) and davidone G (**2**) showed moderate GLUT-4 translocation activity with 1.64 and 1.79 folds enhancement, respectively. This study suggests that prenylated flavonoids could serve as a lead structure for the development of novel anti-diabetic drugs.

## Figures and Tables

**Table 1:** ^1^H and ^13^C NMR data (*δ*) for **1** and **2** (600, 150 MHz) in MeOD-*d*_4_.

No.	1		2	
	*δ*_H_ (*J* in Hz)	*δ*_C_	*δ*_H_ (*J* in Hz)	*δ*_C_
2	5.61, dd, (13.1, 3.0)	75.5	5.42, d, (11.6)	79.8
3	3.13, dd, (17.1, 13.1)	42.7	4.77, d, (11.6)	72.5
	2.66, dd, (17.1, 3.0)			
4		198.7		199.3
5		163.3		162.5
6	5.94, s	96.3	5.87, s	97.8
7		166.3		164.0
8		108.2		102.7
9		162.5		161.6
10		103.3		102.1
1′		119.2		116.9
2′		159.3		160.8
3′	6.48, d, (2.2)	99.8	6.50, d, (2.2)	100.1
4′		160.5		161.0
5′	6.43, dd, (8.3, 2.2)	108.0	6.46, dd, (8.3, 2.2)	108.2
6′	7.32, d, (8.3)	129.1	7.32, d, (8.3)	130.9
1″	2.40, dd, (12.7, 10.7)	26.0	2.74, dd, (16.8, 5.4)	22.9
	2.20, overlapped		2.00, dd, (16.8, 11.2)	
2″	2.20, overlapped	48.9	1.58, m	42.3
3″	2.18, m	28.5	1.71, m	26.6
	1.75, m		1.10, m	
4″	5.29, d, (6.6)	126.6	1.64, m	42.6
			1.36, m	
5″		136.8		71.3
6″	4.26, d, (16.7)	65.7	1.16, s	29.2
	3.68, d, (16.7)			
7″	1.52, s	20.9	1.16, s	29.0
8″		80.3		81.0
9″	1.18, s	24.3	1.42, s	28.0
10″	1.08, s	22.5	1.16, s	20.5
2″-OCH_3_	3.81, s	55.9	3.82, s	56.0

## Data Availability

Not available.

## Supplementary Materials

Supplementary Materials are available online. Figures S1–S10: HRESIMS, 1D and 2D NMR spectra, UV and IR of compound **1**; Figures S11–S20: HRESIMS, 1D and 2D NMR spectra, UV and IR of compound **2**; last part was the calculation detail; Figure S21: DP4+ analysis of compound **1** with isomers **2S**, **2″S-1** and **2**, **2″R-1**; Figure S22: DP4+ analysis of compound **2** with isomers **2R**, **3R**, **2″R-2** and **2R**, **3R**, **2″S-2**.
