# Supplementary material for: Davidones F and G, Two Novel Flavonoids from Sophora davidii (Franch.) Skeels
Source: Molecules. 2021 Jul 9;26(14):4182. doi: 10.3390/molecules26144182 (PMC8306354; doi:10.3390/molecules26144182)
Supplement: Supplementary file 1 [file molecules-26-04182-s001.zip › molecules-1265875-supplementary.pdf]

Supporting Information

Davidones F and G, two novel flavonoids from *Sophora davidii* (Franch.) Skeels

Ping Song [1], Xuecui Li [1], Tongxi Zhou [2], Yu Peng [2], Ho-Young Choi [3], Yuanren Ma [2,*] and Xinzhou Yang [2,*]

[1] *School of Chemistry and Chemical Engineering, Qinghai Nationalities University, Xining 810007, China;*

[2] *School of Pharmaceutical Sciences, South-Central University for Nationalities, Wuhan 430074, China;*

[3] *College of Korean Medicine, Kyung Hee University, Seoul 02447, Republic of Korea*

*Corresponding authors E-mail address: 13007136998@163.com (Y.R. Ma); xzyang@mail.scuec.edu.cn (X.Z. Yang).

CONTENT

**Figure S1** HRESIMS spectrum of **1**
**Figure S2** ^{1}H NMR spectrum (600 MHz, CD_3OD) of **1**
**Figure S3** ^{13}C NMR spectrum (150 MHz, CD_3OD) of **1**
**Figure S4** DEPT 135° spectrum (150 MHz, CD_3OD) of **1**
**Figure S5** HSQC spectrum of **1**
**Figure S6** COSY spectrum of **1**
**Figure S7** HMBC spectrum of **1**
**Figure S8** ROESY spectrum of **1**
**Figure S9** UV spectrum of **1**
**Figure S10** IR spectrum of **1**
**Figure S11** HRESIMS spectrum of **2**
**Figure S12** ^{1}H NMR spectrum (600 MHz,CD_3OD) of **2**
**Fingue S13** ^{13}C NMR spectrum (150 MHz, CD_3OD) of **2**
**Figure S14** DEPT 135° spectrum (150 MHz, CD_3OD) of **2**
**Figure S15** HSQC spectrum of **2**
**Figure S16** COSY spectrum of **2**
**Figure S17** HMBC spectrum of **2**
**Figure S18** ROESY spectrum of **2**
**Figure S19** UV spectrum of **2**
**Figure S20** IR spectrum of **2**
**Calculation detail**
**Figure S21 DP4+ analysis of compound 1 with isomers 2S, 2"S-1 and 2S, 2"R-1**
**Figure S22 DP4+ analysis of compound 2 with isomers 2R, 3R, 2"R-2 and 2R, 3R, 2"S-2**
**Figure S23 Caculated and experimental optical rotation values of Compounds 1 and 2**

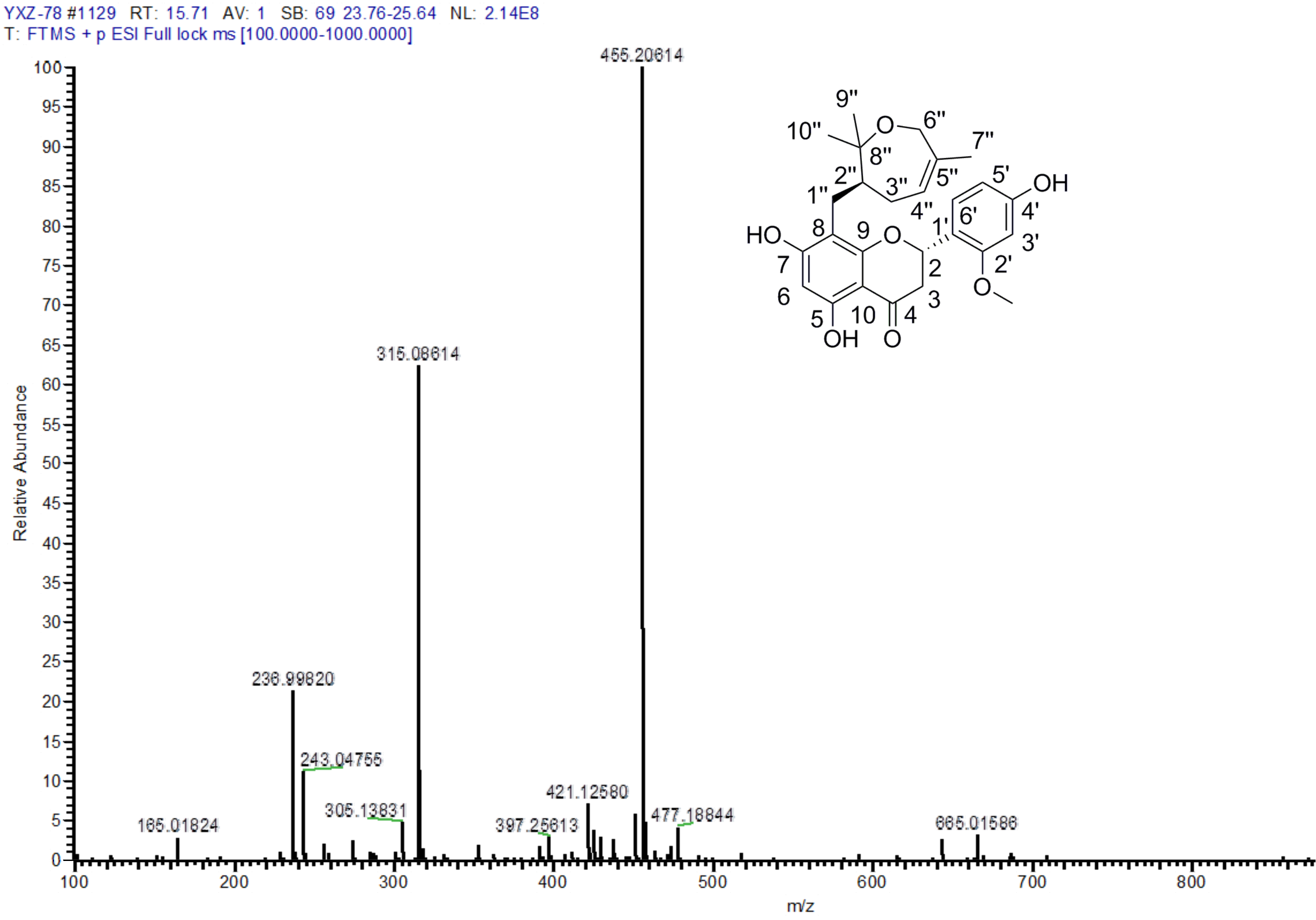

Figure S1 HRESIMS spectrum of **1**

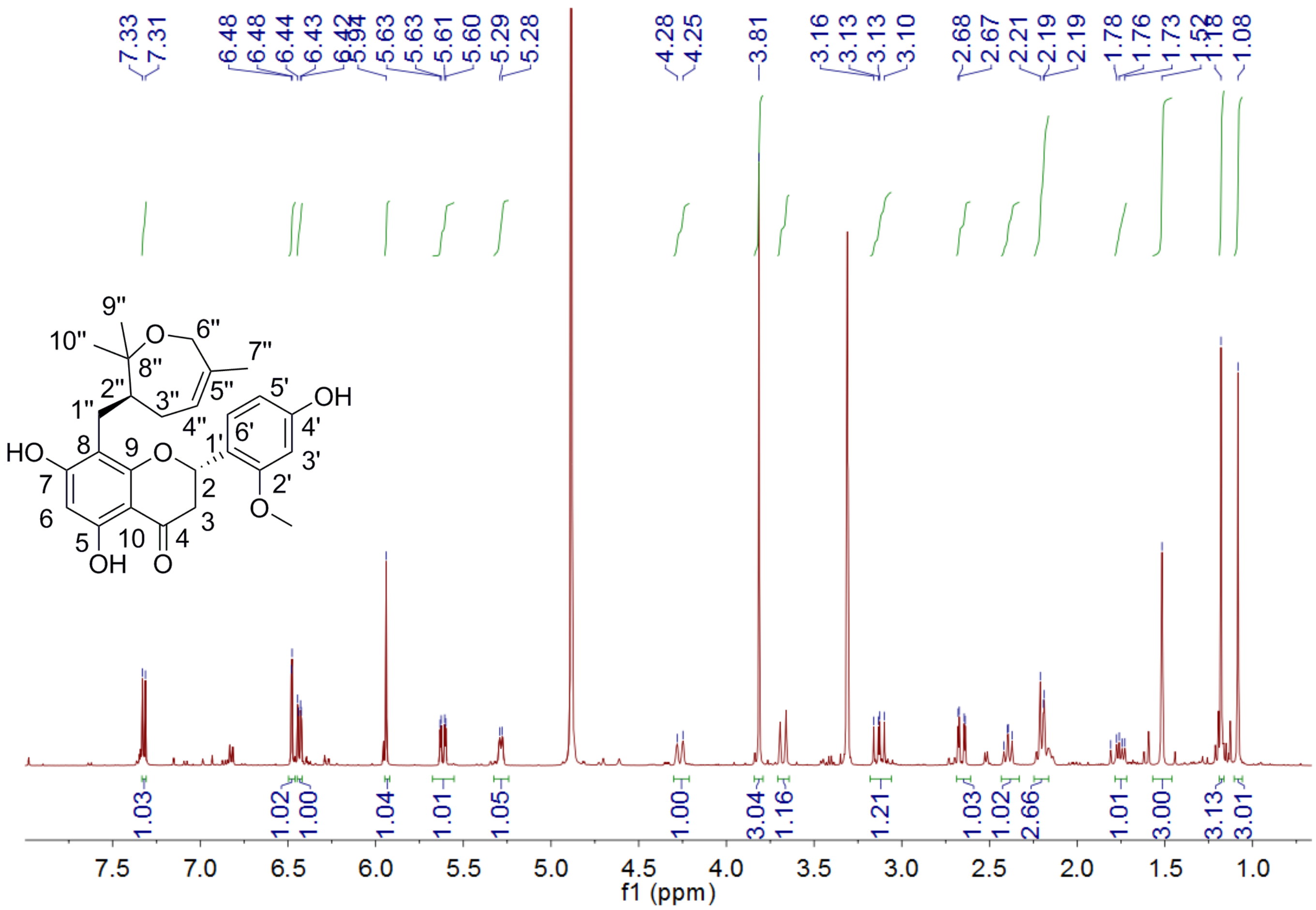

Figure S2 ^{1}H NMR spectrum (600 MHz, CD_3OD) of **1**

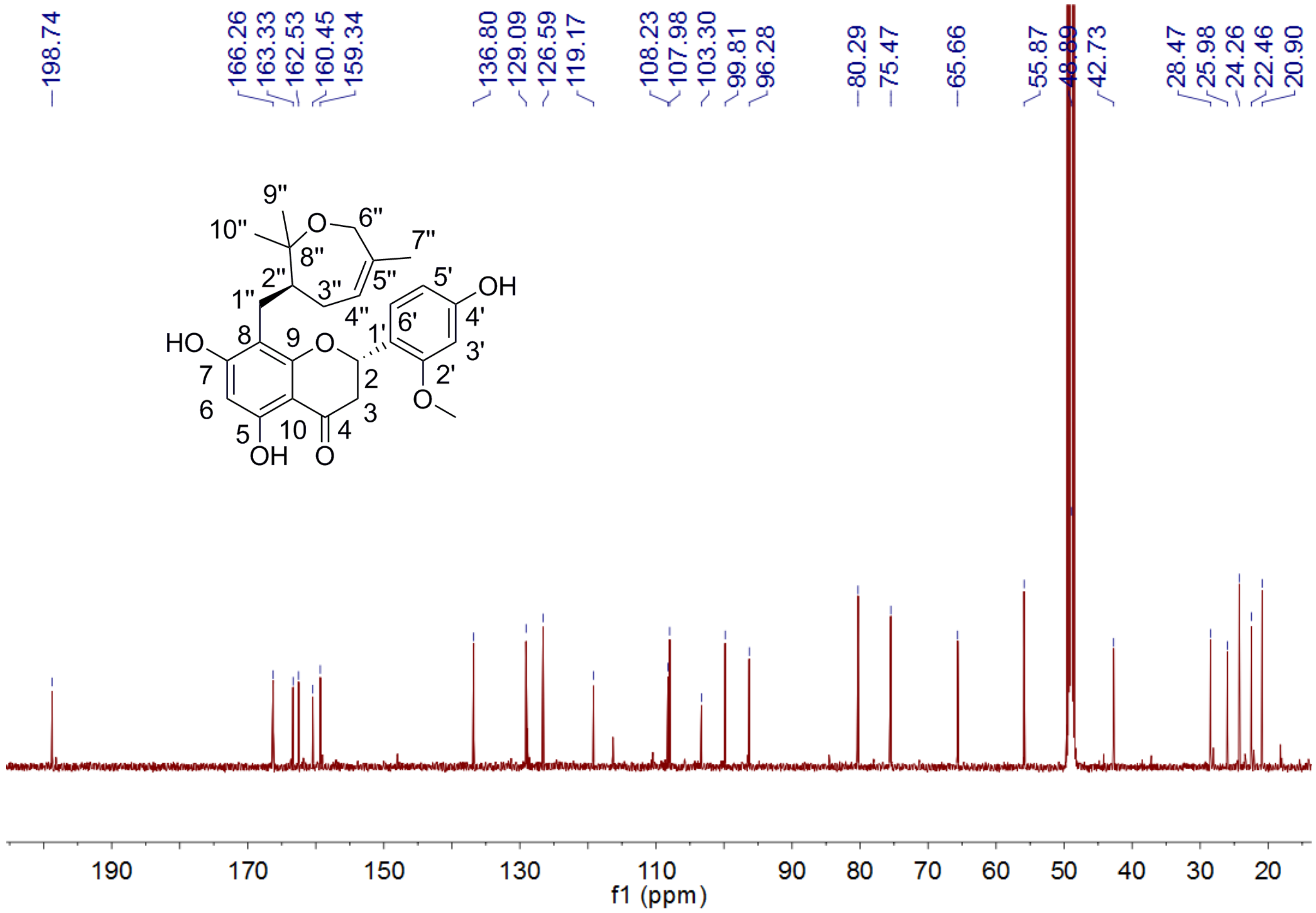

Figure S3 ^{13}C NMR spectrum (150 MHz, CD_3OD) of **1**

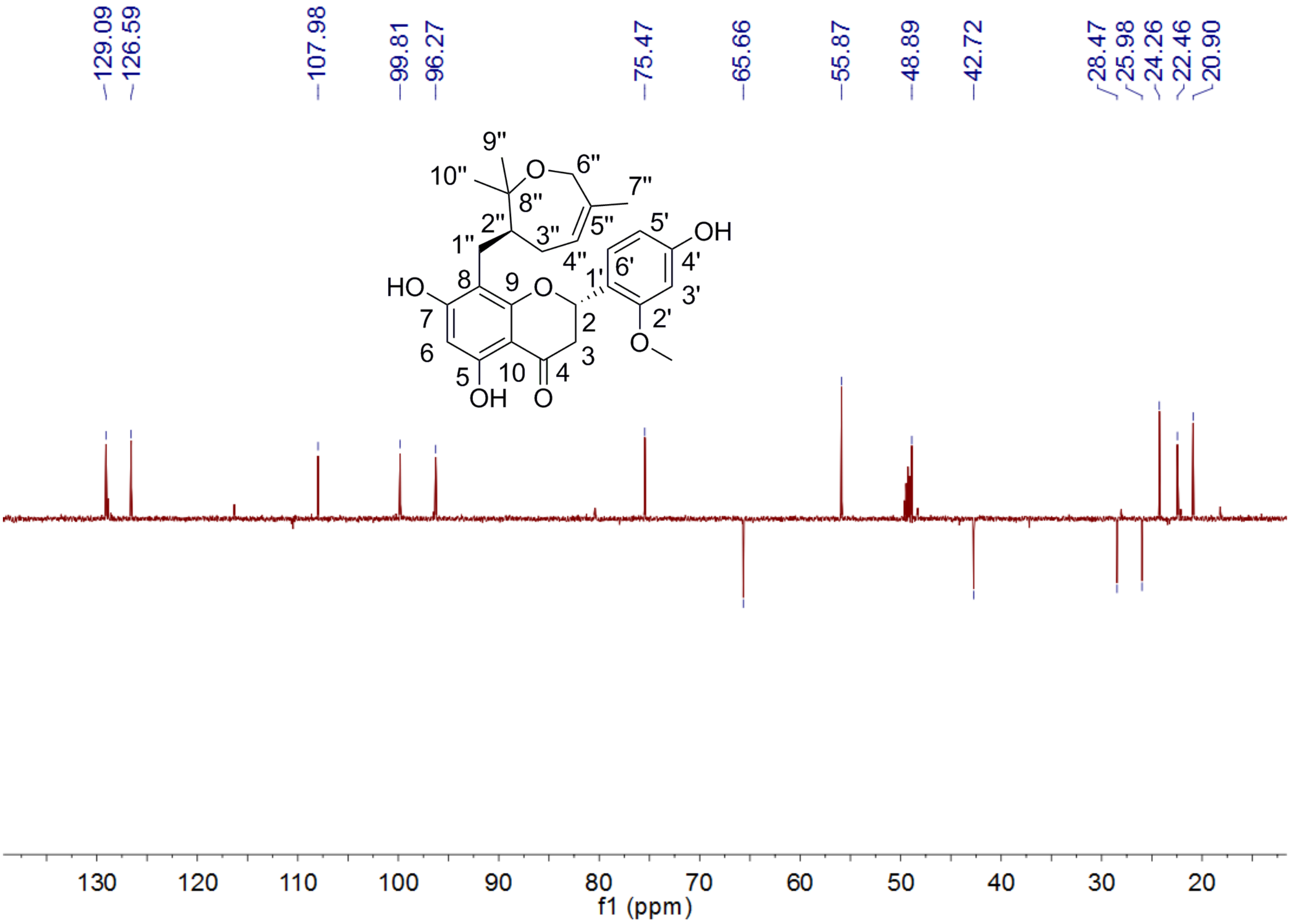

Figure S4 DEPT 135° spectrum (150 MHz, CD_3OD) of **1**

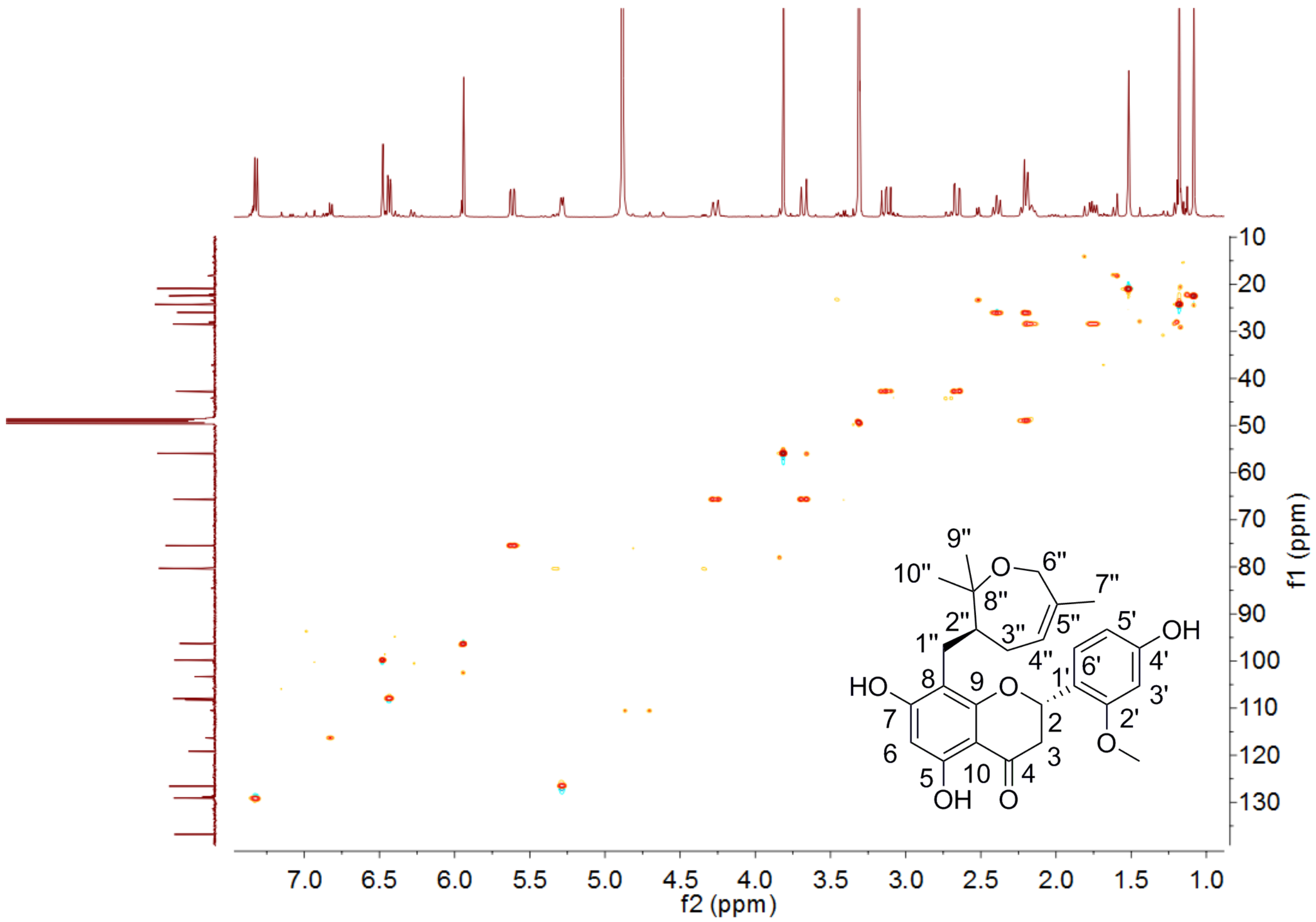

Figure S5 HSQC spectrum of **1**

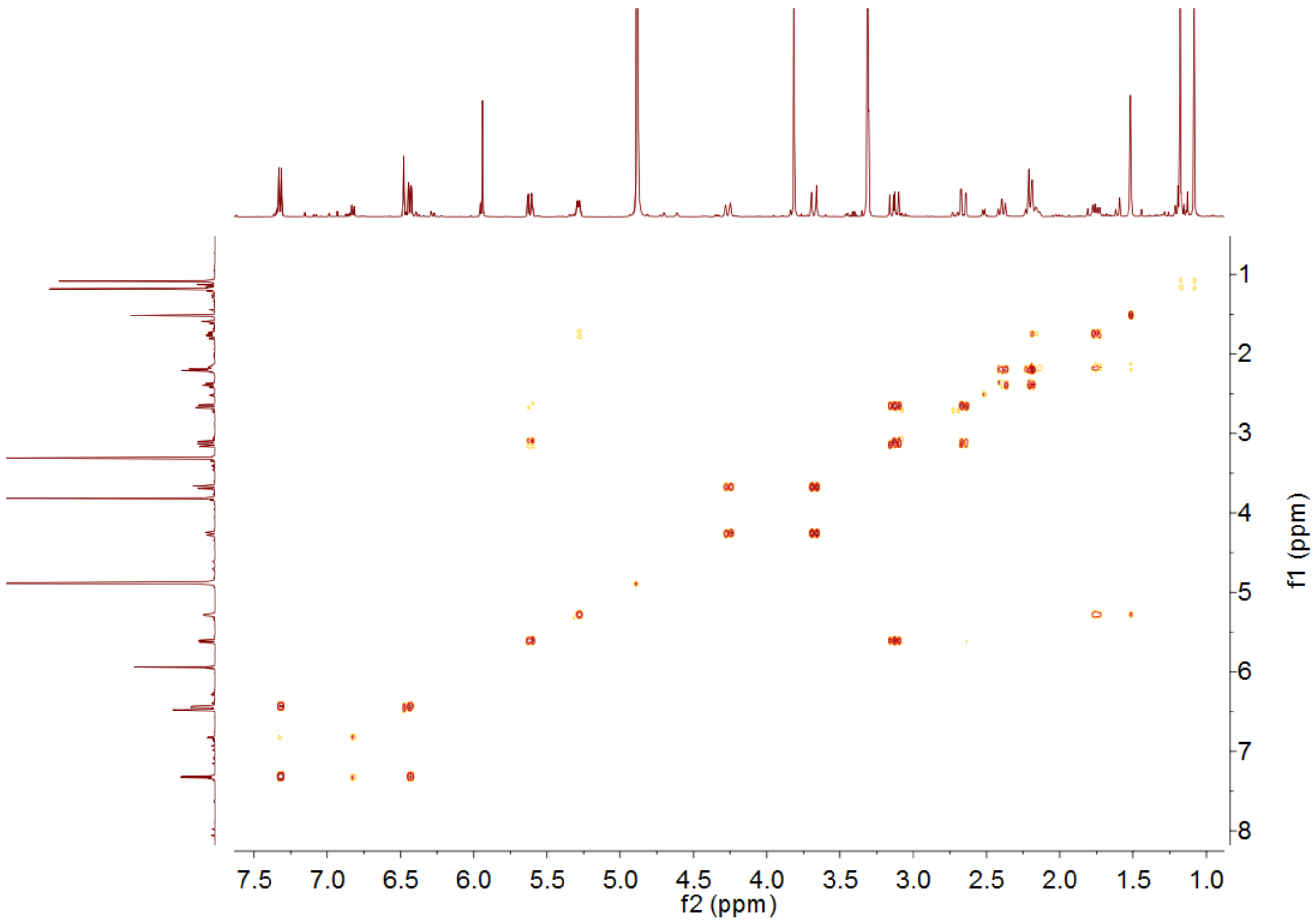

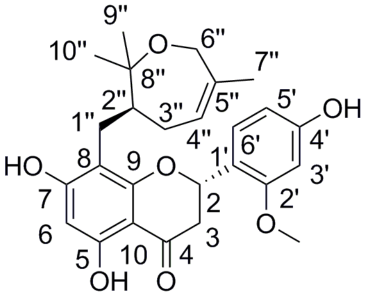

Figure S6 COSY spectrum of **1**

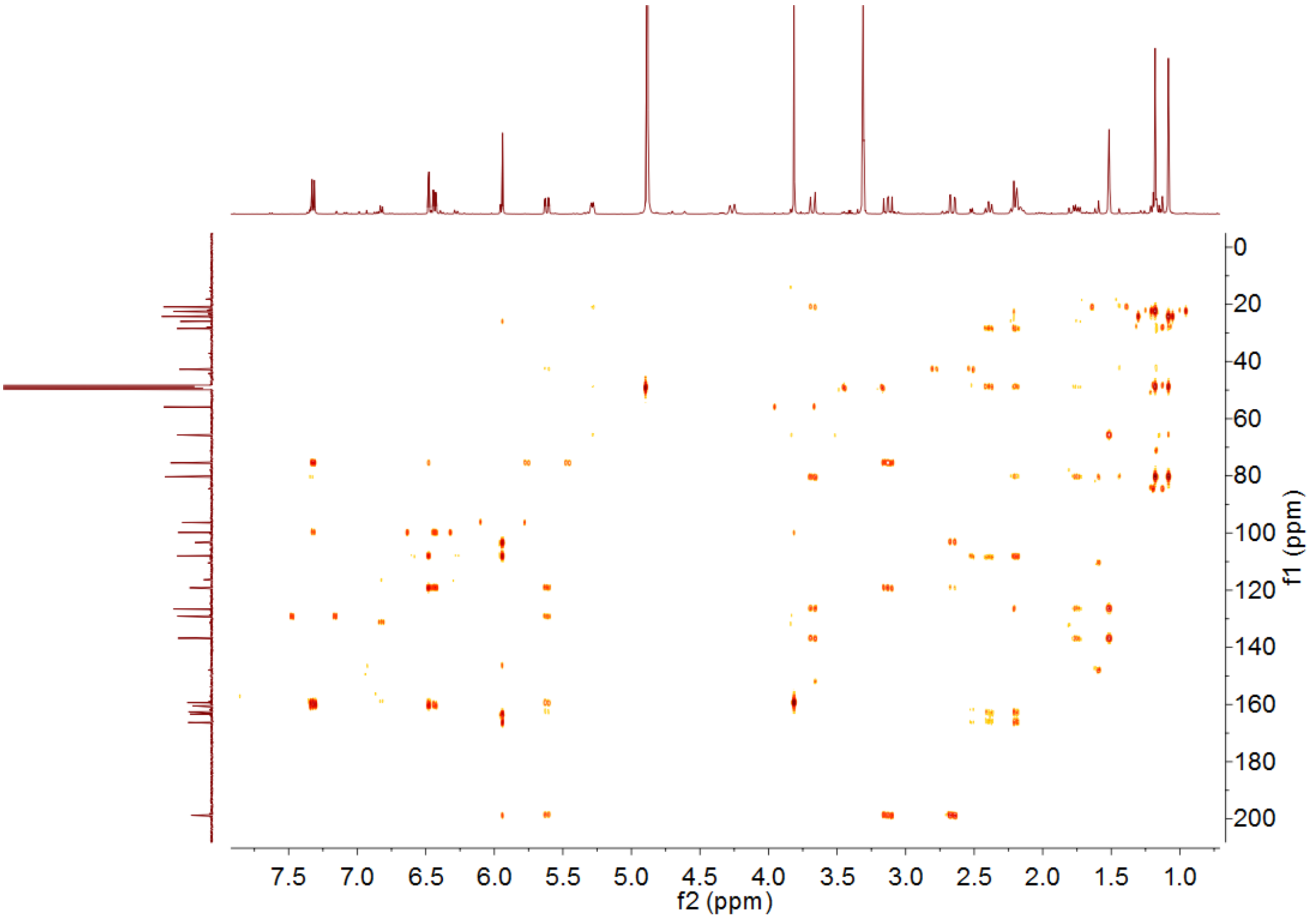

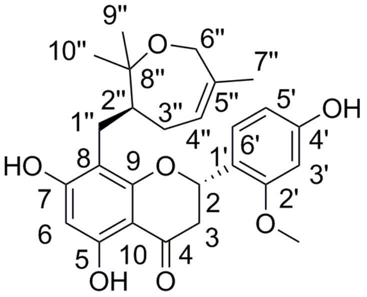

Figure S7 HMBC spectrum of **1**

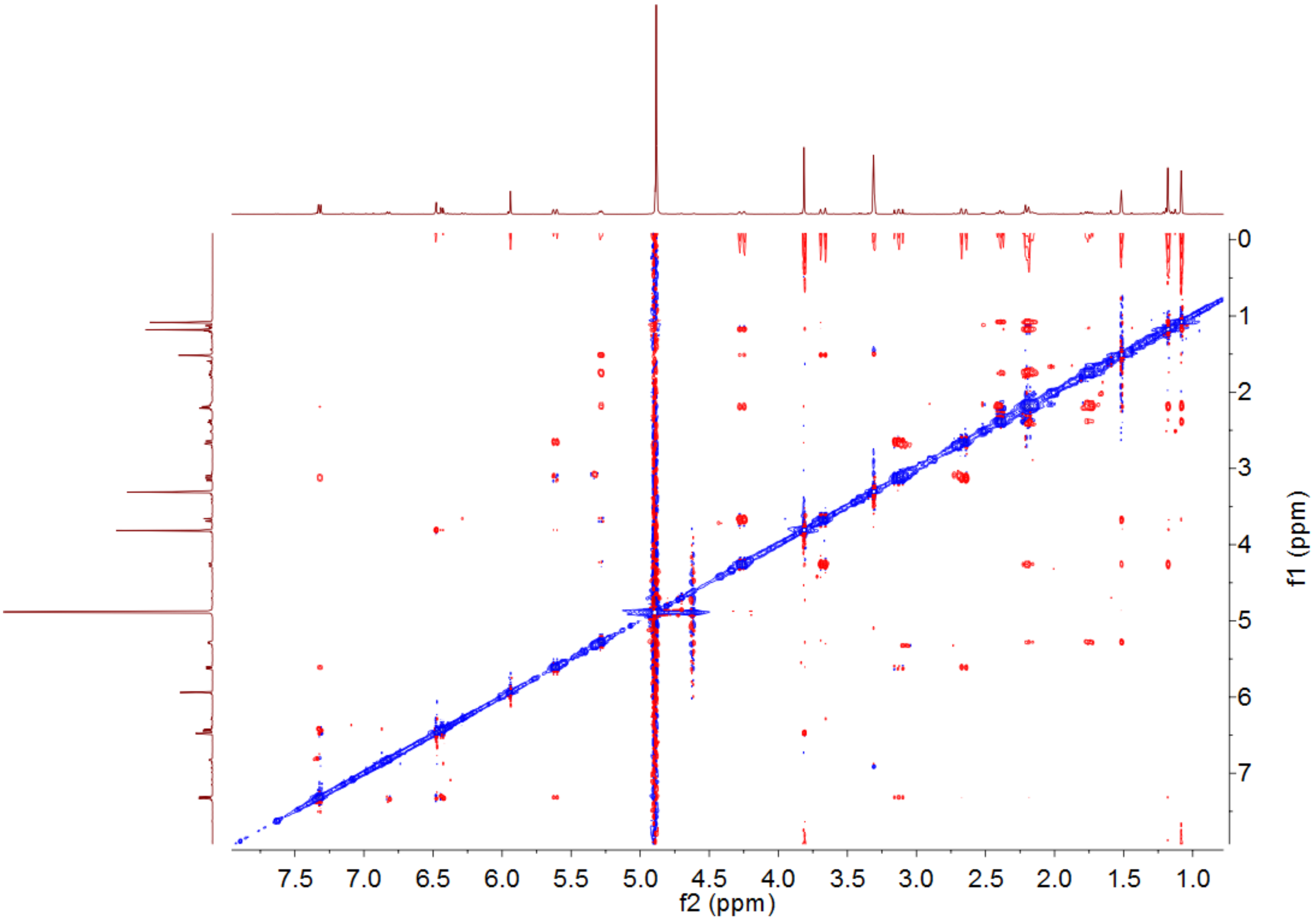

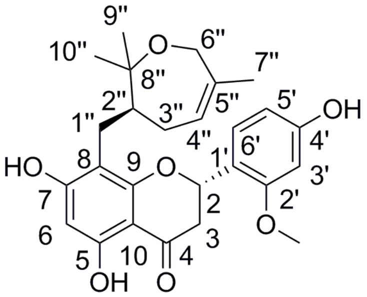

Figure S8 ROESY spectrum of **1**

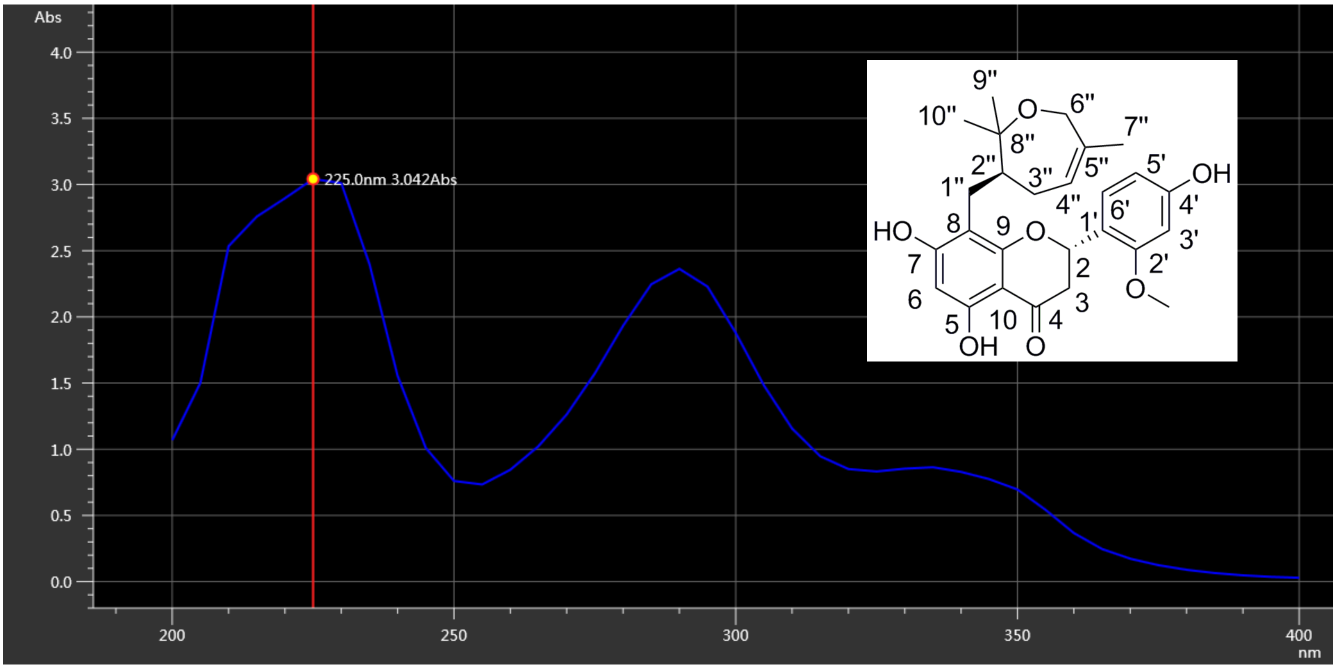

Figure S9 UV spectrum of **1**

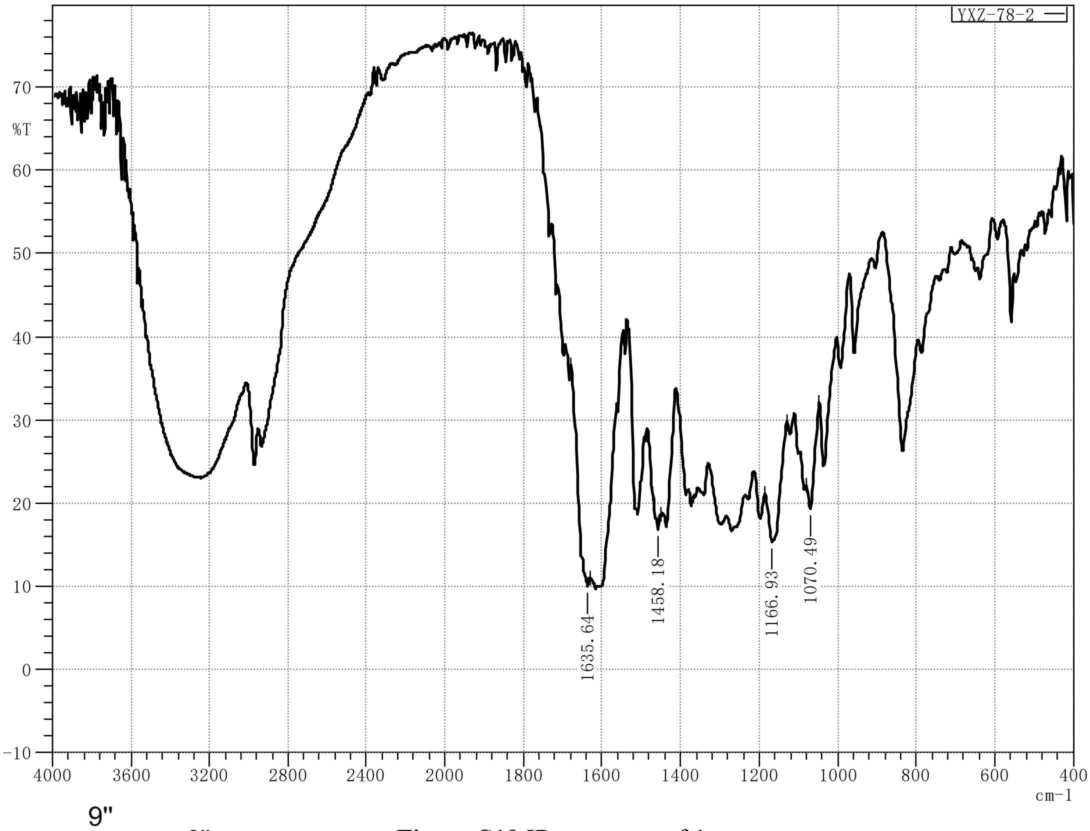

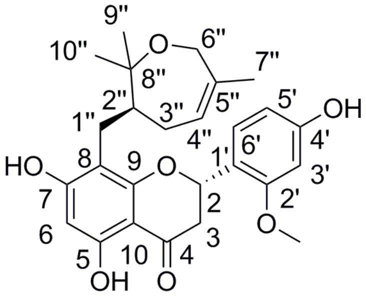

Figure S10 IR spectrum of **1**

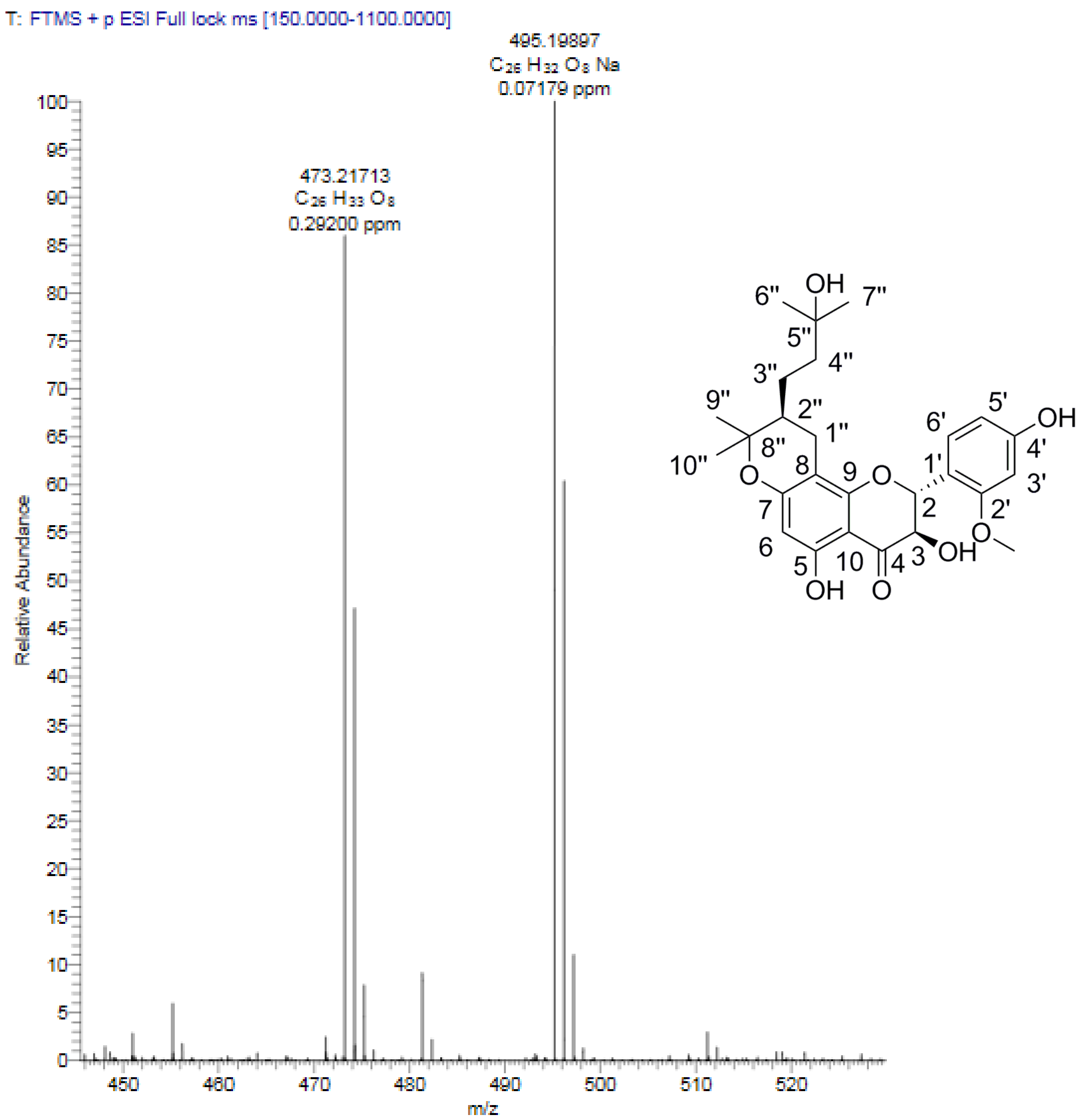

Figure S11 HRESIMS spectrum of **2**

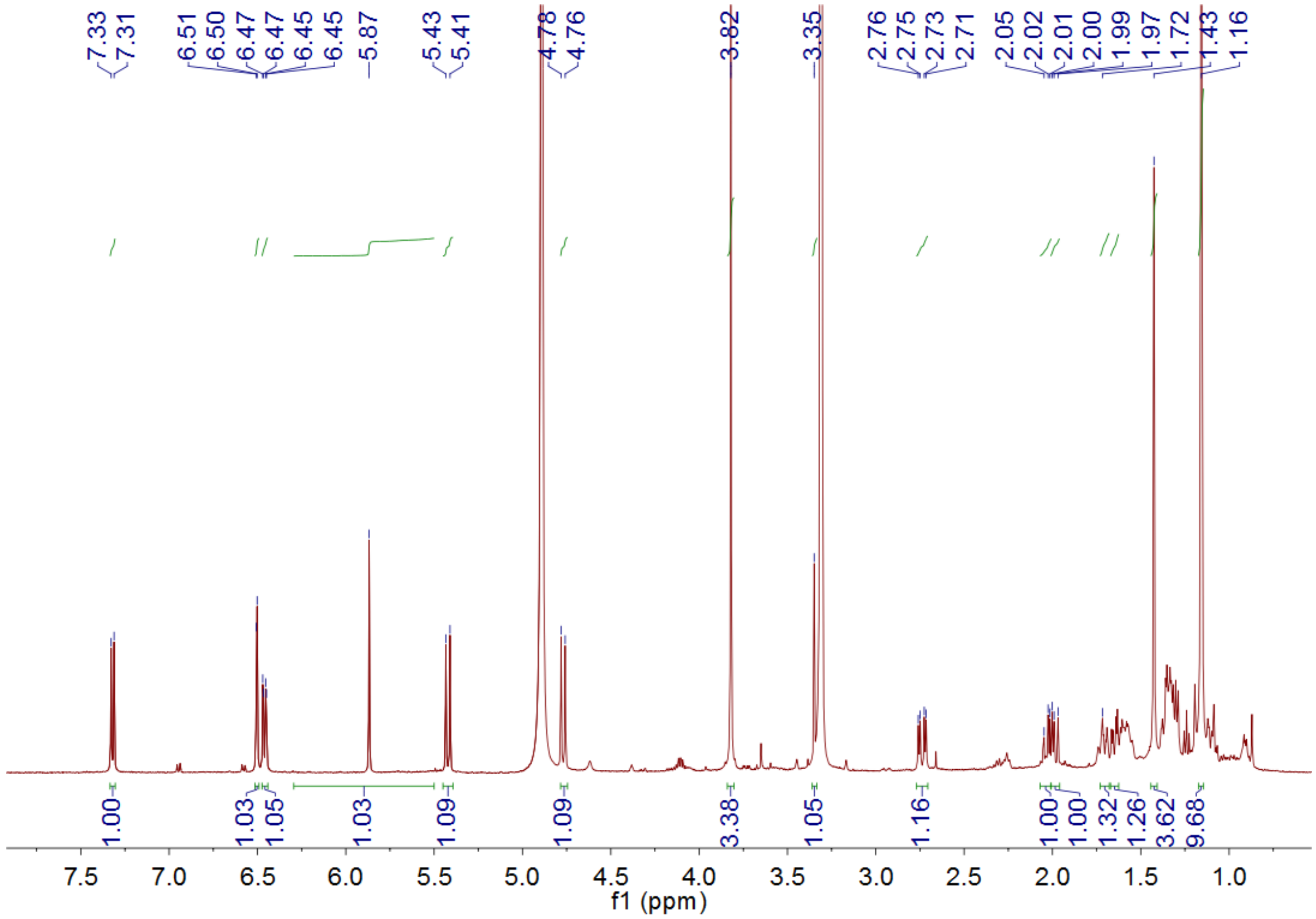

Figure S12 ^{1}H NMR spectrum (600 MHz,CD_3OD) of **2**

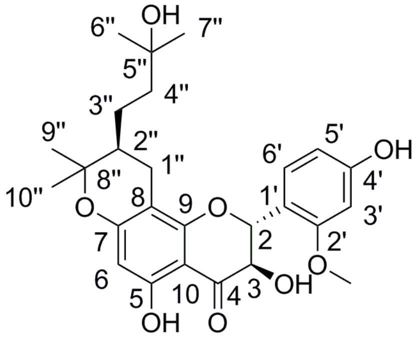

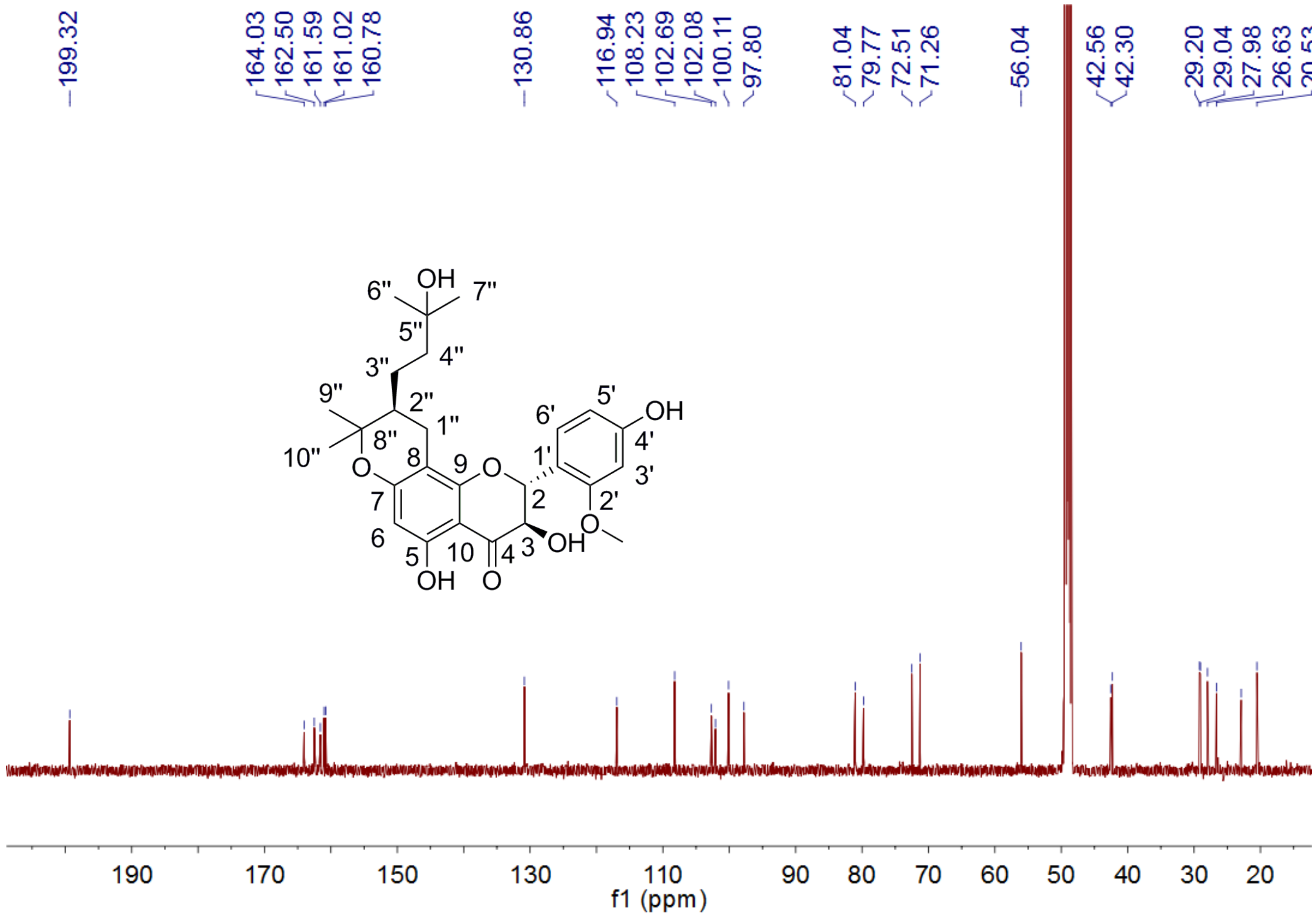

Fingue S13 ^{13}C NMR spectrum (150 MHz, CD_3OD) of **2**

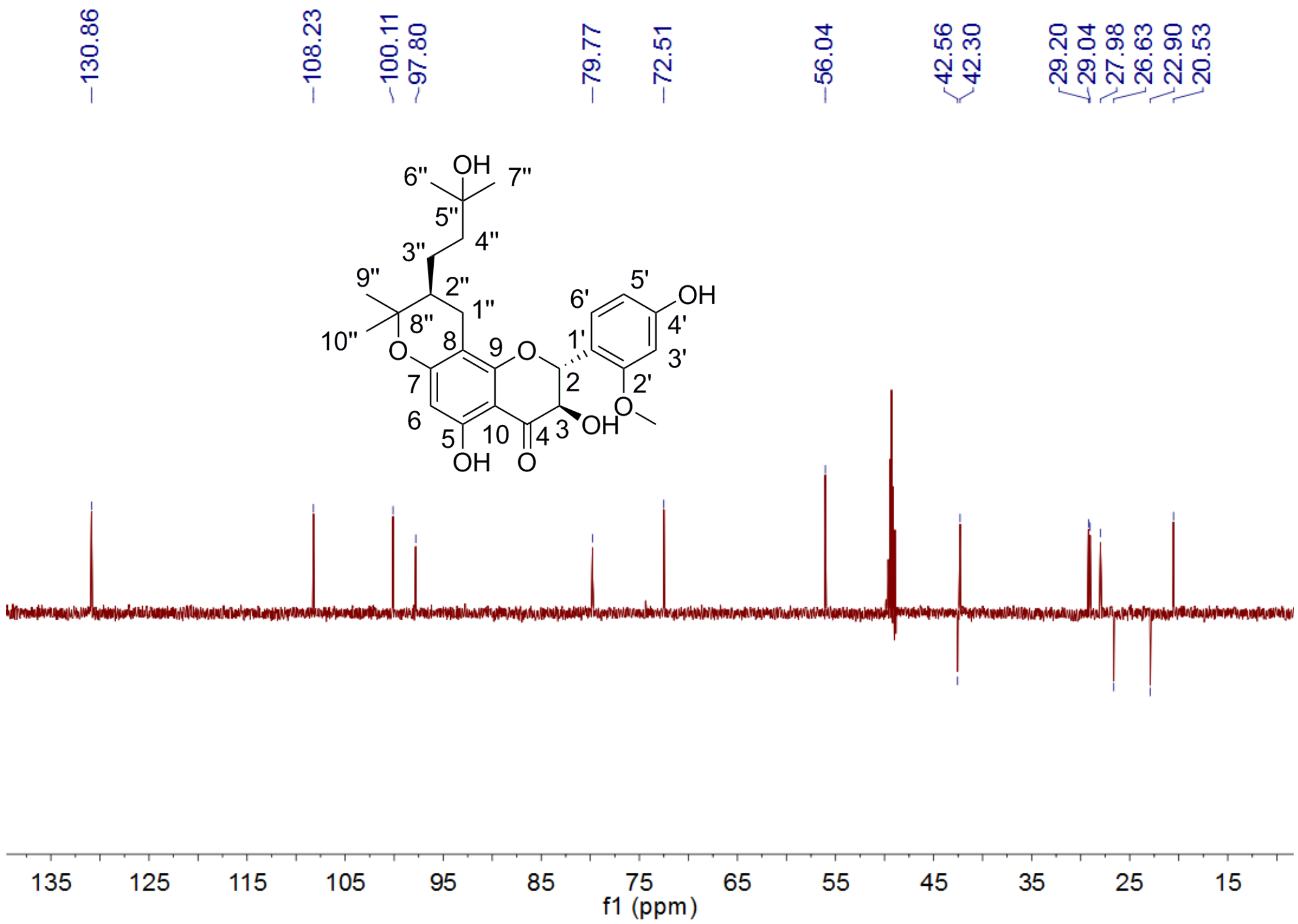

Figure S14 DEPT 135° spectrum (150 MHz, CD_3OD) of **2**

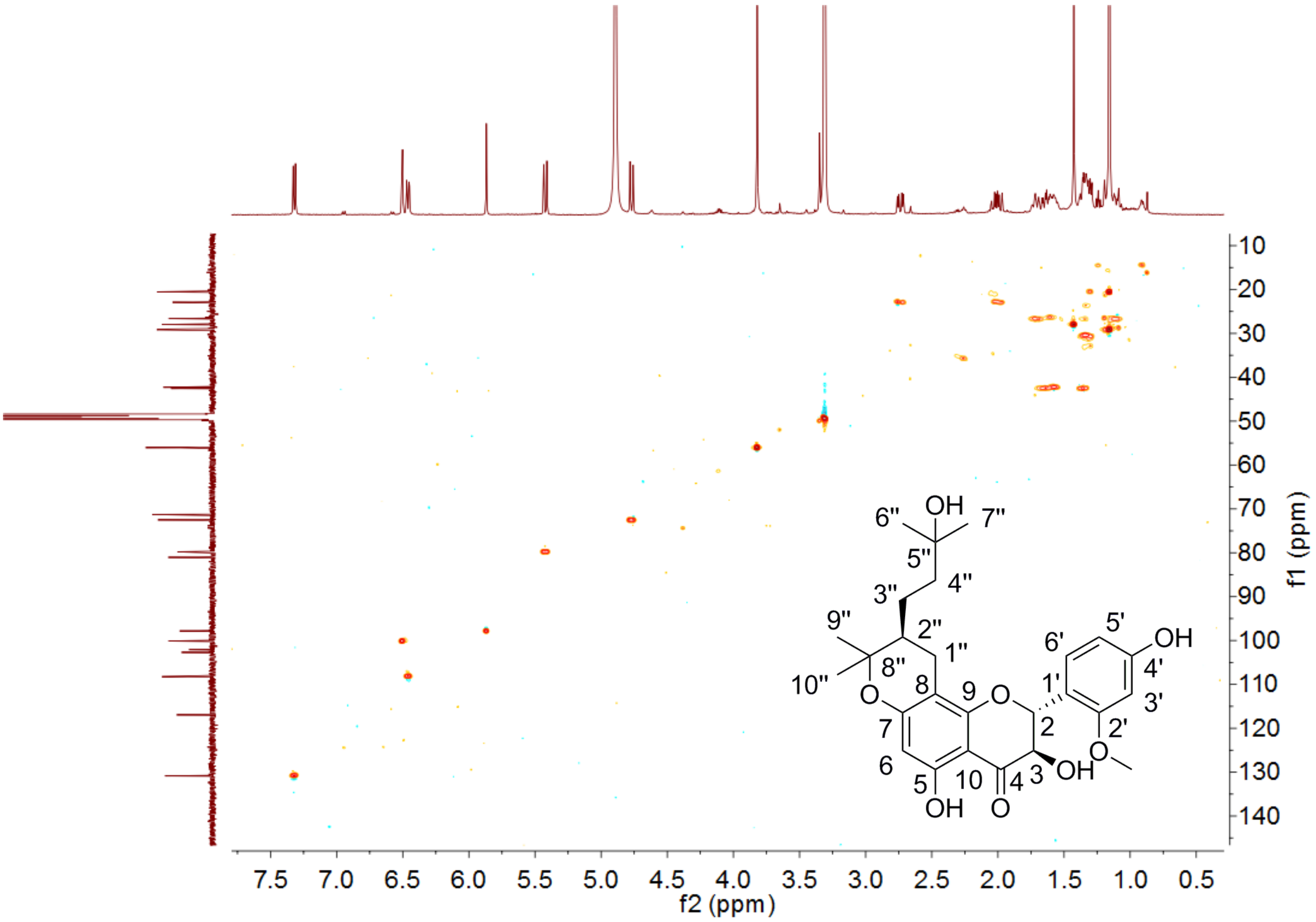

Figure S15 HSQC spectrum of **2**

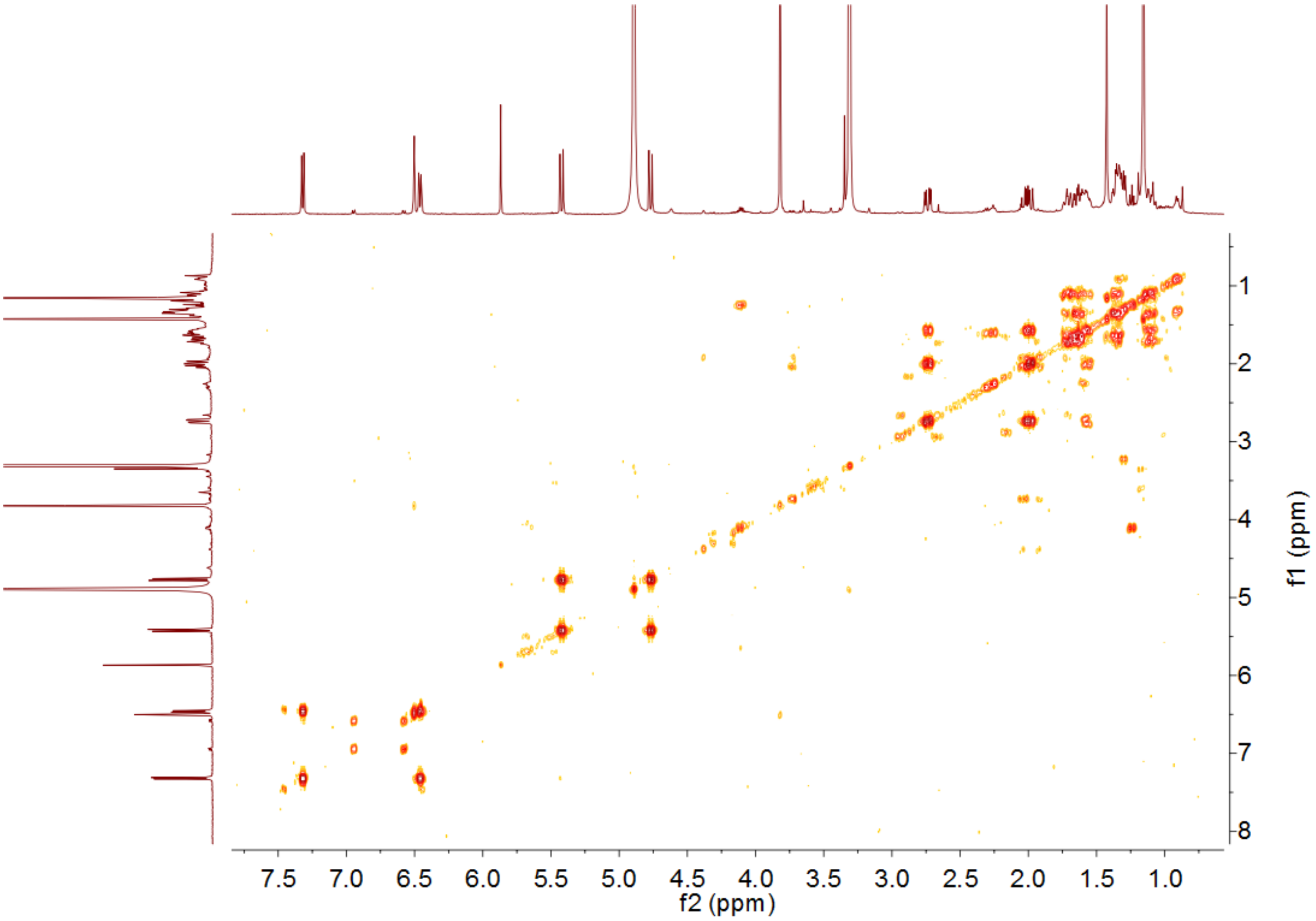

Figure S16 COSY spectrum of **2**

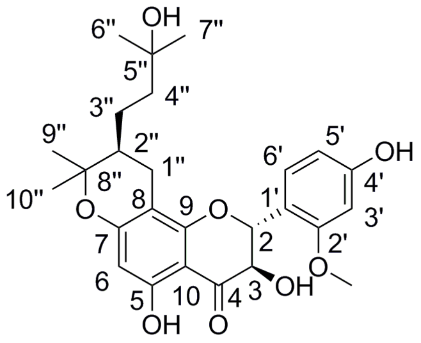

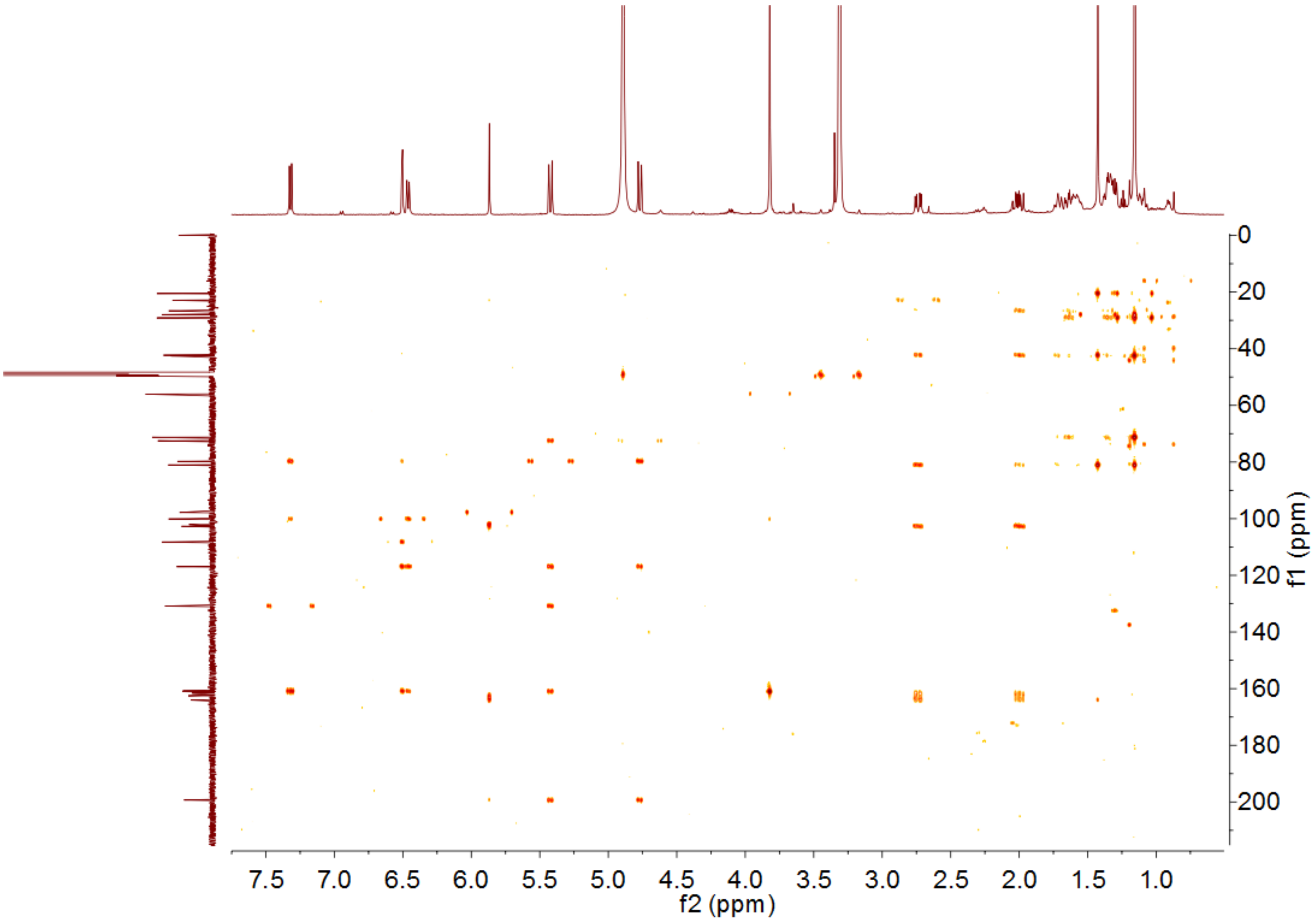

Figure S17 HMBC spectrum of **2**

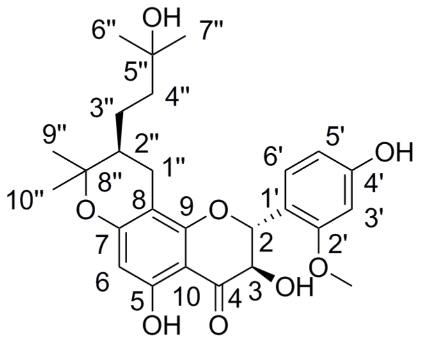

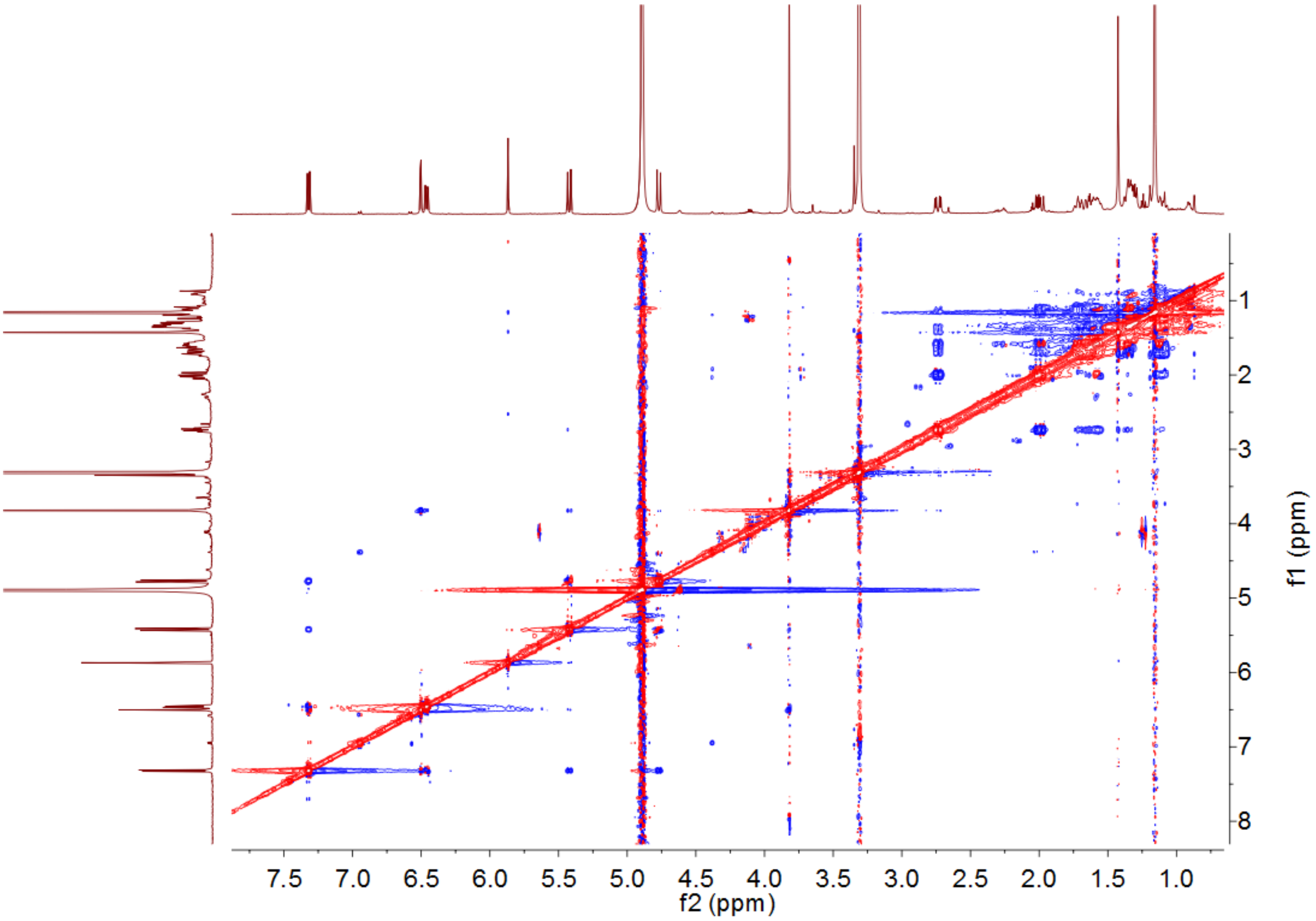

Figure S18 ROESY spectrum of **2**

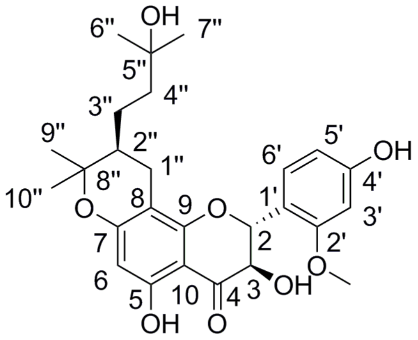

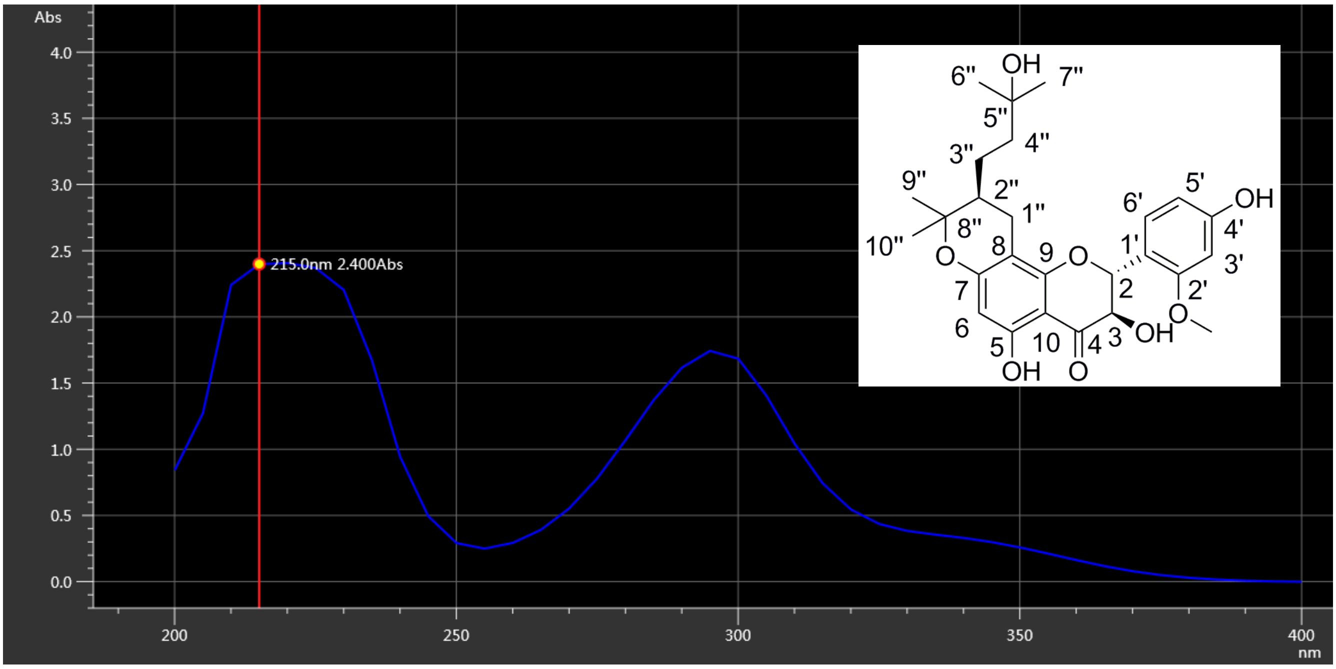

Figure S19 UV spectrum of **2**

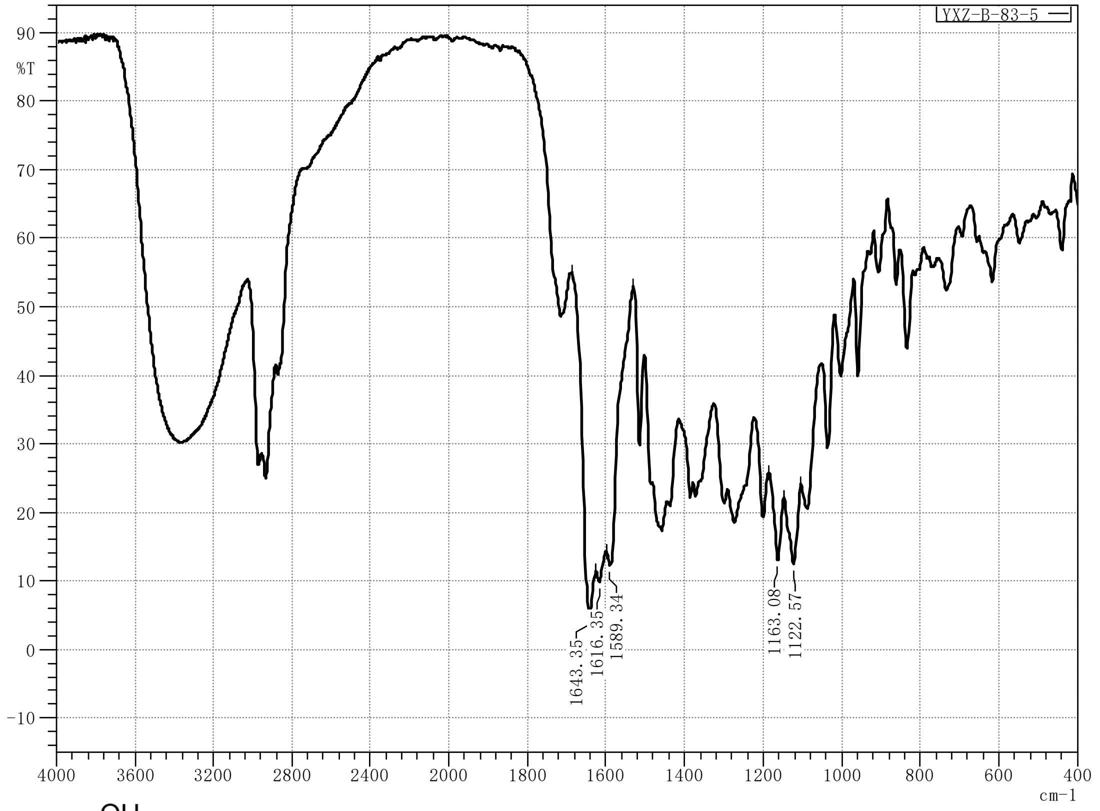

Figure S20 IR spectrum of **2**

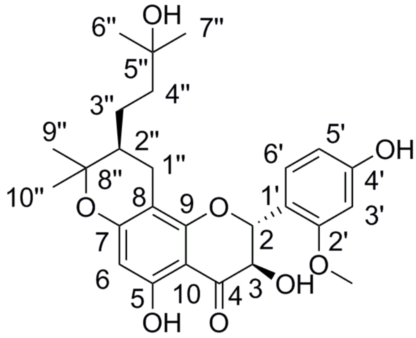

Calculation detail

Table S1. Experimental chemical shifts and Boltzmann-averaged calculated ^{13}C NMR shifts of two diastereomers of 1 (δ in ppm).

no.	2S, 2"S-1			2S, 2"R-1		
	δ_C, exptl[a]	δ_C, (adj_calcd)[b]	δ_C, $\Delta\delta$[c]	δ_C, exptl[a]	δ_C, (adj_calcd)[b]	δ_C, $\Delta\delta$[c]
1	166.3	172.2	5.9	166.3	171.3	5
2	96.3	100.65	4.35	96.3	99.34	3.04
3	163.3	170.65	7.35	163.3	170.4	7.1
4	103.3	106.61	3.31	103.3	106.74	3.44
5	162.5	168.63	6.13	162.5	168.96	6.46
6	108.2	111.72	3.52	108.2	111.65	3.45
7	198.7	202.9	4.2	198.7	203.04	4.34
8	42.7	46.55	3.85	42.7	46.58	3.88
9	75.5	80.32	4.82	75.5	80.25	4.75
10	119.2	125.55	6.35	119.2	125.4	6.2
11	159.3	164.28	4.98	159.3	164.53	5.23
12	99.8	100.88	1.08	99.8	100.88	1.08
13	160.5	165.38	4.88	160.5	165.45	4.95
14	108	109.81	1.81	108	110.1	2.1
15	129.1	133.64	4.54	129.1	134.62	5.52
16	55.9	56.61	0.71	55.9	56.58	0.68
17	26	28.2	2.2	26	27.85	1.85
18	48.9	51.7	2.8	48.9	52.33	3.43
19	28.5	34.63	6.13	28.5	31.32	2.82
20	126.6	131.52	4.92	126.6	132.86	6.26
21	136.8	147.66	10.86	136.8	147.82	11.02
22	65.7	70.71	5.01	65.7	69.7	4
23	80.3	83.55	3.25	80.3	83.21	2.91
24	24.3	28.39	4.09	24.3	25.83	1.53
25	22.5	24.61	2.11	22.5	24.13	1.63
26	20.9	24.83	3.93	20.9	24.19	3.29

[a]Recorded in CD_3OD at 150 MHz. [b]Calculated in CD_3OD. [c]$\Delta\delta = |\delta_{adj\_calcd}-\delta_{exptl}|$

Table S2. Experimental chemical shifts and Boltzmann-averaged calculated ^{1}H NMR shifts of two diastereomers of 1 (δ in ppm).

no.	2S, 2"S-1			2S, 2"R-1		
	δ_H, exptl[a]	δ_H, (adj_calcd)[b]	δ_H, $\Delta\delta$[c]	δ_H, exptl[a]	δ_H, (adj_calcd)[b]	δ_H, $\Delta\delta$[c]
1	5.94	6.19	0.25	5.94	6.06	0.12
2	2.66	2.61	0.05	2.66	2.52	0.14
3	3.13	2.66	0.47	3.13	2.77	0.36
4	5.61	5.49	0.12	5.61	5.53	0.08
5	6.48	6.51	0.03	6.48	6.50	0.02
6	6.43	6.63	0.2	6.43	6.68	0.25
7	7.32	7.80	0.48	7.32	7.80	0.48

8	3.81	3.83	0.02	3.81	3.84	0.03
9	2.20	2.45	0.25	2.20	2.38	0.18
10	2.40	2.64	0.24	2.40	2.49	0.09
11	2.20	1.97	0.23	2.20	2.19	0.01
12	1.75	2.24	0.49	1.75	1.81	0.06
13	2.18	2.26	0.08	2.18	2.40	0.22
14	5.29	5.75	0.46	5.29	5.71	0.42
15	3.68	4.13	0.45	3.68	3.96	0.28
16	4.26	4.42	0.16	4.26	4.50	0.24
17	1.08	1.15	0.07	1.08	1.10	0.02
18	1.18	1.26	0.08	1.18	1.27	0.09
19	1.52	1.65	0.13	1.52	1.61	0.09

[a]Recorded in CD_3OD at 600 MHz. [b]Calculated in CD_3OD. [c]$\Delta\delta = |\delta_{adj\_calcd}-\delta_{exptl}|$

Table S3. Conformers and Bolzmann distributions of the optimized 2S, 2"S-1

species	*E'=E+ZPE*	*E*	*H*	*G*	*ΔG*	*ΔE(kcal/mol)*	*p%*
1	-1534.851395	-1534.821087	-1534.820143	-1534.911846	0	0	40.18%
2	-1534.849326	-1534.818599	-1534.817654	-1534.911119	0.000727	0.456199406	18.59%
3	-1534.848375	-1534.817372	-1534.816428	-1534.911071	0.000775	0.486319862	17.67%
4	-1534.848703	-1534.817765	-1534.816821	-1534.911037	0.000809	0.507655185	17.05%
5	-1534.848914	-1534.818683	-1534.817738	-1534.909441	0.002405	1.509160347	3.14%
6	-1534.84701	-1534.816028	-1534.815084	-1534.909181	0.002665	1.672312817	2.38%
7	-1534.8464	-1534.815534	-1534.814589	-1534.908029	0.003817	2.395203762	0.70%
8	-1534.846673	-1534.81661	-1534.815666	-1534.906515	0.005331	3.345253145	0.14%
9	-1534.845051	-1534.814287	-1534.813343	-1534.906343	0.005503	3.453184778	0.12%
10	-1534.842947	-1534.812101	-1534.811156	-1534.904645	0.007201	4.518695909	0.02%
11	-1534.841553	-1534.810634	-1534.80969	-1534.903686	0.00816	5.12047752	0.01%

***E*, *E'*, *H, G*: total energy, total energy with zero point energy (ZPE), enthalpy, and Gibbs free energy**

Table S4. Conformers and Bolzmann distributions of the optimized 2S, 2"R-1

species	*E'=E+ZPE*	*E*	*H*	*G*	*ΔG*	*ΔE(kcal/mol)*	*p%*
1	-1534.849287	-1534.818222	-1534.817278	-1534.911961	0	0	44.98%
2	-1534.848519	-1534.817463	-1534.816518	-1534.911104	0.000857	0.537775641	18.14%
3	-1534.849262	-1534.81859	-1534.817646	-1534.910864	0.001097	0.688377922	14.06%
4	-1534.849821	-1534.819553	-1534.818609	-1534.910347	0.001614	1.012800333	8.13%
5	-1534.847273	-1534.816364	-1534.81542	-1534.909427	0.002534	1.590109073	3.07%
6	-1534.847273	-1534.816364	-1534.81542	-1534.909425	0.002536	1.591364092	3.06%
7	-1534.847602	-1534.816691	-1534.815747	-1534.909299	0.002662	1.670430289	2.68%
8	-1534.848321	-1534.818062	-1534.817118	-1534.909187	0.002774	1.740711353	2.38%
9	-1534.846747	-1534.815792	-1534.814848	-1534.908624	0.003337	2.093999202	1.31%
10	-1534.846315	-1534.815403	-1534.814459	-1534.908515	0.003446	2.162397737	1.17%
11	-1534.84518	-1534.814123	-1534.813179	-1534.907547	0.004414	2.769826933	0.42%
12	-1534.845115	-1534.814271	-1534.813327	-1534.907289	0.004672	2.931724384	0.32%

13	-1534.844862	-1534.813987	-1534.813043	-1534.907016	0.004945	3.103034477	0.24%
14	-1534.844757	-1534.813961	-1534.813017	-1534.905546	0.006415	4.025473443	0.05%

Table S5. Stable conformers of compound 1 with 2S, 2"S-1 configurations.

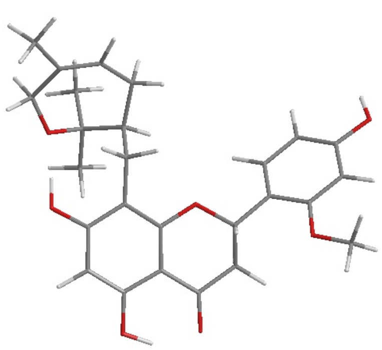

Conformer 1 40.18%

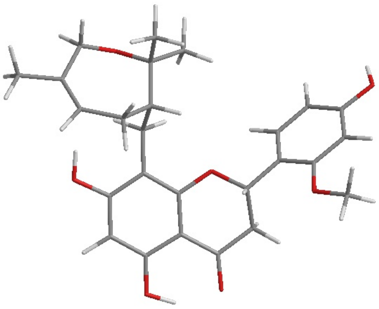

Conformer 2 18.59%

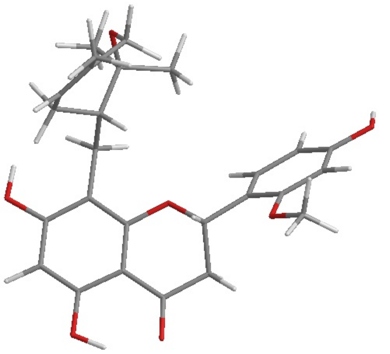

Conformer 3 17.67%

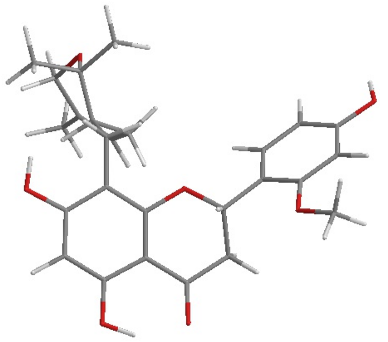

Conformer 4 17.05%

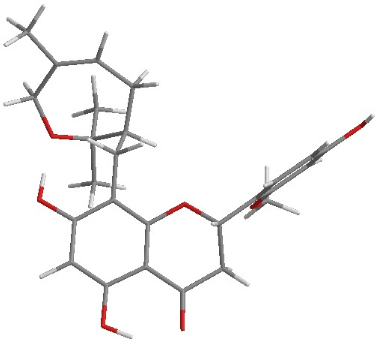

Conformer 5 3.14%

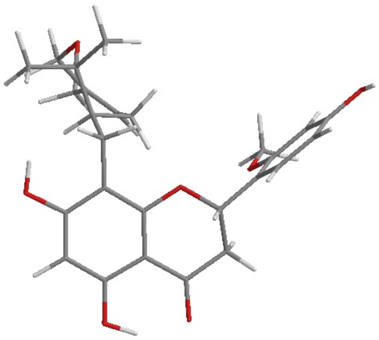

Conformer 6 2.38%

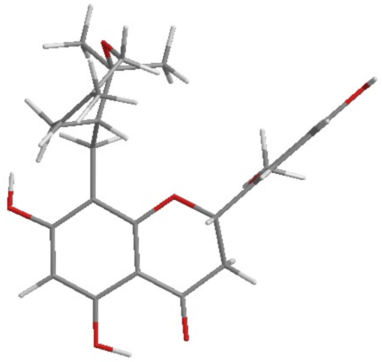

Conformer 7 0.70%

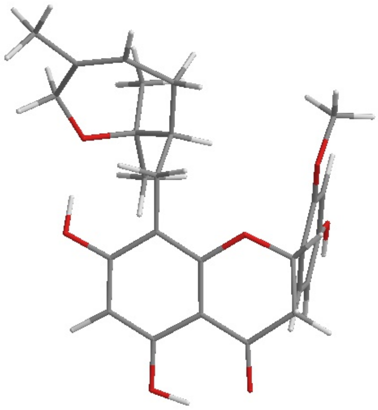

Conformer 8 0.14%

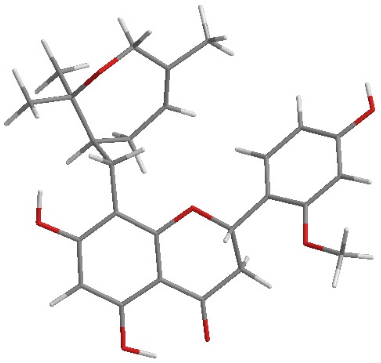

Conformer 9 0.12%

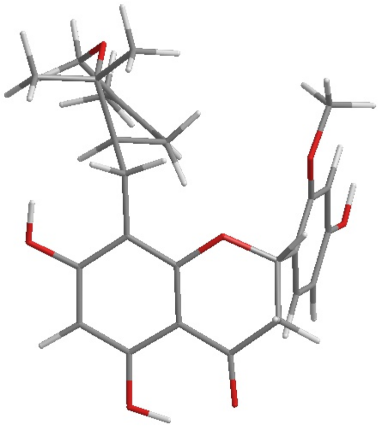

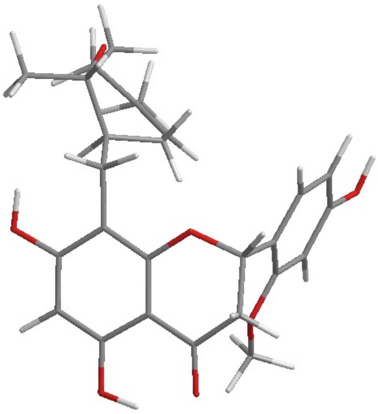

Conformer 10 0.02% **Conformer 11 0.01%**

Table S6. Stable conformers of compound 1 with 2S, 2''R-1 configurations.

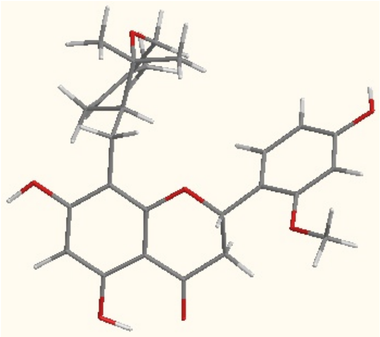

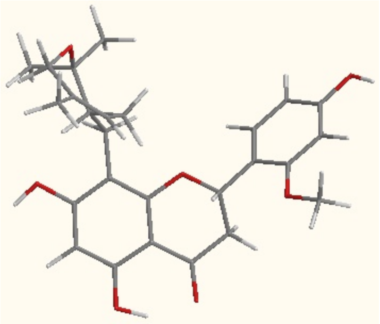

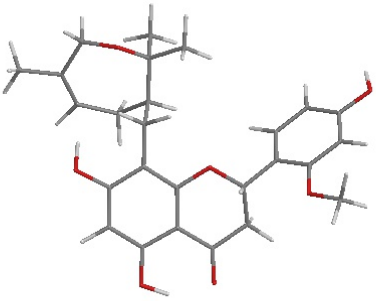

Conformer 1 44.98% **Conformer 2 18.14%** **Conformer 3 14.06%**

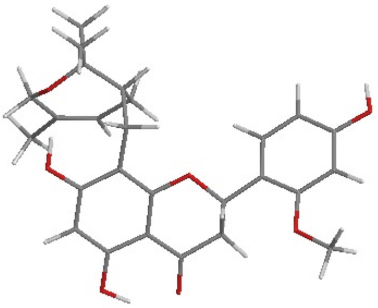

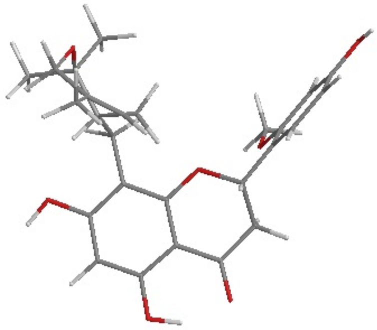

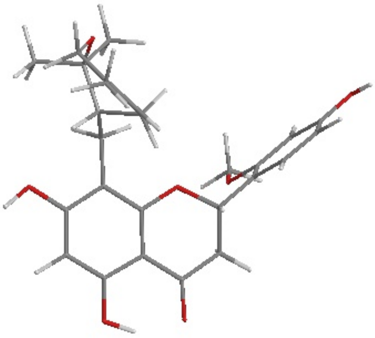

Conformer 4 8.13% **Conformer 5 3.07%** **Conformer 6 3.06%**

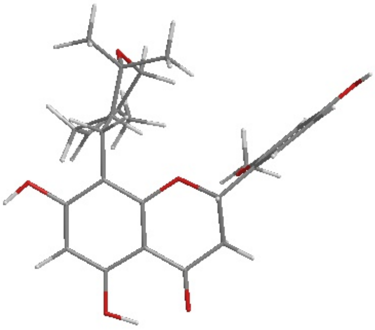

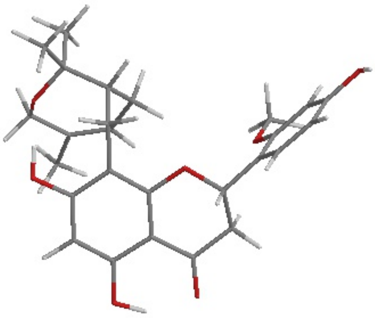

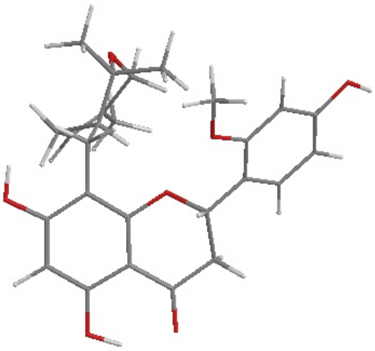

Conformer 7 2.68% **Conformer 8 2.38%** **Conformer 9 1.31%**

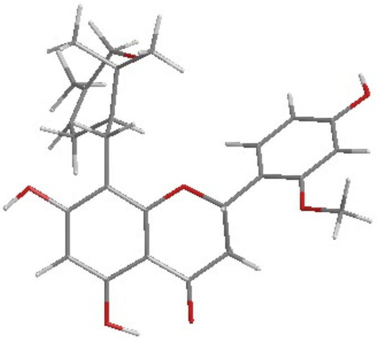

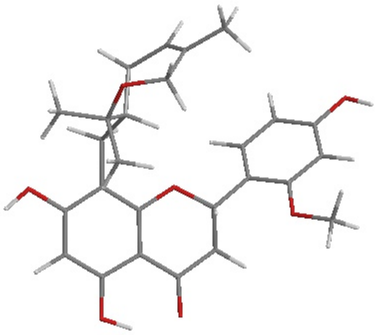

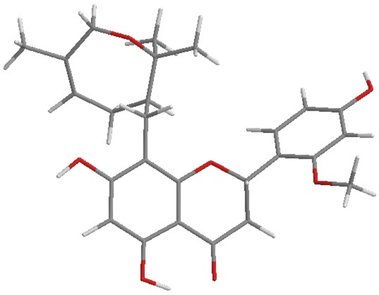

Conformer 10 1.17% **Conformer 11 0.42%** **Conformer 12 0.32%**

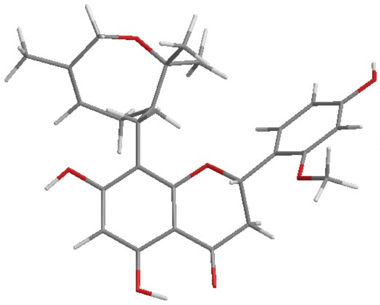

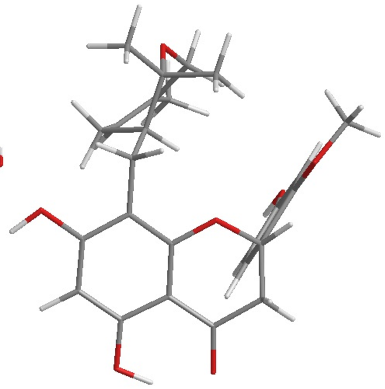

Conformer 13 0.24% **Conformer 14 0.05%**

Table S7. Experimental chemical shifts and calculated ^{13}C NMR shifts of conformers of 2S, 2"S-1 (δ in ppm).

no.	2S, 2"S-1 Conf. 1			2S, 2"S-1 Conf. 2		
	δ_C, exptl[a]	δ_C, (adj_calcd)[b]	δ_C, $\Delta\delta$[c]	δ_C, exptl[a]	δ_C, (adj_calcd)[b]	δ_C, $\Delta\delta$[c]
1	166.3	173.681025	7.381025	166.3	171.851925	5.551925
2	96.3	101.723725	5.423725	96.3	99.899625	3.599625
3	163.3	170.719625	7.419625	163.3	170.338125	7.038125
4	103.3	106.439425	3.139425	103.3	106.742525	3.442525
5	162.5	168.492425	5.992425	162.5	168.778225	6.278225
6	108.2	114.209225	6.009225	108.2	110.501225	2.301225
7	198.7	202.665425	3.965425	198.7	202.847825	4.147825
8	42.7	46.788025	4.088025	42.7	46.615325	3.915325
9	75.5	79.845725	4.345725	75.5	79.942125	4.442125
10	119.2	125.884625	6.684625	119.2	125.522825	6.322825
11	159.3	164.022625	4.722625	159.3	163.812825	4.512825
12	99.8	100.742025	0.942025	99.8	100.848925	1.048925
13	160.5	165.195925	4.695925	160.5	165.390525	4.890525
14	108	109.873025	1.873025	108	109.856225	1.856225
15	129.1	132.913725	3.813725	129.1	133.345325	4.245325
16	55.9	56.656425	0.756425	55.9	56.596025	0.696025
17	26	30.390925	4.390925	26	23.595225	2.404775
18	48.9	51.105825	2.205825	48.9	49.829425	0.929425

19	28.5	38.634225	10.134225	28.5	32.155025	3.655025
20	126.6	131.467425	4.867425	126.6	129.468325	2.868325
21	136.8	144.902725	8.102725	136.8	154.236225	17.436225
22	65.7	71.933625	6.233625	65.7	71.349825	5.649825
23	80.3	84.998825	4.698825	80.3	82.405825	2.105825
24	24.3	30.722825	6.422825	24.3	31.229925	6.929925
25	22.5	25.541625	3.041625	22.5	23.676725	1.176725
26	20.9	25.368625	4.468625	20.9	26.045025	5.145025

no.		**2S, 2"S-1 Conf. 3**			**2S, 2"S-1 Conf. 4**	
	δ_C, exptl[a]	δ_C, (adj_calcd)[b]	δ_C, $\Delta\delta$[c]	δ_C, exptl[a]	δ_C, (adj_calcd)[b]	δ_C, $\Delta\delta$[c]
1	166.3	170.475625	4.175625	166.3	170.965325	4.665325
2	96.3	99.715025	3.415025	96.3	100.096025	3.796025
3	163.3	170.437625	7.137625	163.3	171.032725	7.732725
4	103.3	106.913725	3.613725	103.3	106.687225	3.387225
5	162.5	168.898325	6.398325	162.5	168.541925	6.041925
6	108.2	109.601825	1.401825	108.2	109.541925	1.341925
7	198.7	202.844725	4.144725	198.7	203.281625	4.581625
8	42.7	46.803225	4.103225	42.7	46.780725	4.080725
9	75.5	79.939925	4.439925	75.5	80.348625	4.848625
10	119.2	125.513125	6.313125	119.2	125.748025	6.548025
11	159.3	164.055725	4.755725	159.3	164.106425	4.806425
12	99.8	100.729625	0.929625	99.8	100.898925	1.098925
13	160.5	165.384625	4.884625	160.5	165.291325	4.791325
14	108	109.733125	1.733125	108	109.900625	1.900625
15	129.1	133.927025	4.827025	129.1	133.420925	4.320925
16	55.9	56.605425	0.705425	55.9	56.597725	0.697725
17	26	28.565925	2.565925	26	27.459125	1.459125
18	48.9	53.243825	4.343825	48.9	53.370025	4.470025
19	28.5	31.132625	2.632625	28.5	31.451225	2.951225
20	126.6	132.239525	5.639525	126.6	132.923025	6.323025
21	136.8	147.539125	10.739125	136.8	147.663725	10.863725
22	65.7	68.947825	3.247825	65.7	69.042925	3.342925
23	80.3	82.607825	2.307825	80.3	82.307825	2.007825
24	24.3	24.749025	0.449025	24.3	23.959925	0.340075
25	22.5	23.971025	1.471025	22.5	24.123225	1.623225
26	20.9	23.555025	2.655025	20.9	23.635125	2.735125

no.		**2S, 2"S-1 Conf. 5**			**2S, 2"S-1 Conf. 6**	
	δ_C, exptl[a]	δ_C, (adj_calcd)[b]	δ_C, $\Delta\delta$[c]	δ_C, exptl[a]	δ_C, (adj_calcd)[b]	δ_C, $\Delta\delta$[c]
1	166.3	173.373925	7.073925	166.3	170.776425	4.476425
2	96.3	101.082825	4.782825	96.3	99.368925	3.068925
3	163.3	170.664925	7.364925	163.3	170.925025	7.625025
4	103.3	106.114425	2.814425	103.3	106.299325	2.999325
5	162.5	168.625725	6.125725	162.5	168.537125	6.037125
6	108.2	113.511725	5.311725	108.2	109.072925	0.872925

7	198.7	203.414825	4.714825	198.7	203.935925	5.235925
8	42.7	43.783225	1.083225	42.7	43.530325	0.830325
9	75.5	85.425025	9.925025	75.5	85.654925	10.154925
10	119.2	123.143725	3.943725	119.2	122.849525	3.649525
11	159.3	168.296425	8.996425	159.3	168.254425	8.954425
12	99.8	102.181825	2.381825	99.8	102.353025	2.553025
13	160.5	166.772425	6.272425	160.5	166.815825	6.315825
14	108	109.315025	1.315025	108	109.326325	1.326325
15	129.1	138.984425	9.884425	129.1	138.910925	9.810925
16	55.9	56.342925	0.442925	55.9	56.437725	0.537725
17	26	29.936125	3.936125	26	27.361625	1.361625
18	48.9	50.404125	1.504125	48.9	54.155125	5.255125
19	28.5	38.333825	9.833825	28.5	31.121425	2.621425
20	126.6	131.470625	4.870625	126.6	133.106625	6.506625
21	136.8	144.776525	7.976525	136.8	147.582525	10.782525
22	65.7	71.901125	6.201125	65.7	69.028025	3.328025
23	80.3	84.973525	4.673525	80.3	82.262525	1.962525
24	24.3	30.800725	6.500725	24.3	23.807125	0.492875
25	22.5	25.379725	2.879725	22.5	23.719525	1.219525
26	20.9	25.554525	4.654525	20.9	23.594725	2.694725

no.		**2S, 2"S-1 Conf. 7**			**2S, 2"S-1 Conf. 8**	
	δ_C, exptl[a]	δ_C, (adj_calcd)[b]	δ_C, $\Delta\delta$[c]	δ_C, exptl[a]	δ_C, (adj_calcd)[b]	δ_C, $\Delta\delta$[c]
1	166.3	170.210825	3.910825	166.3	173.592525	7.292525
2	96.3	99.021825	2.721825	96.3	101.164325	4.864325
3	163.3	170.331125	7.031125	163.3	170.145425	6.845425
4	103.3	106.561925	3.261925	103.3	107.193925	3.893925
5	162.5	168.938525	6.438525	162.5	166.090225	3.590225
6	108.2	108.762625	0.562625	108.2	114.208825	6.008825
7	198.7	203.768625	5.068625	198.7	202.685425	3.985425
8	42.7	43.423025	0.723025	42.7	42.460325	0.239675
9	75.5	85.744525	10.244525	75.5	77.148025	1.648025
10	119.2	123.008925	3.808925	119.2	124.292425	5.092425
11	159.3	168.378825	9.078825	159.3	167.669325	8.369325
12	99.8	102.304625	2.504625	99.8	101.537725	1.737725
13	160.5	166.921325	6.421325	160.5	166.252225	5.752225
14	108	109.398325	1.398325	108	108.744025	0.744025
15	129.1	139.173125	10.073125	129.1	136.120925	7.020925
16	55.9	56.353925	0.453925	55.9	56.706225	0.806225
17	26	28.734025	2.734025	26	29.320325	3.320325
18	48.9	53.300925	4.400925	48.9	49.799125	0.899125
19	28.5	31.468725	2.968725	28.5	38.360525	9.860525
20	126.6	132.138225	5.538225	126.6	131.853525	5.253525
21	136.8	147.845725	11.045725	136.8	145.187225	8.387225
22	65.7	68.864825	3.164825	65.7	71.868125	6.168125
23	80.3	82.664025	2.364025	80.3	84.647225	4.347225

24	24.3	23.342025	0.957975	24.3	30.375625	6.075625
25	22.5	24.013025	1.513025	22.5	25.126025	2.626025
26	20.9	23.545825	2.645825	20.9	25.468125	4.568125

no.		**2S, 2"S-1 Conf. 9**			**2S, 2"S-1 Conf. 10**	
	δ_C, exptl[a]	δ_C, (adj_calcd)[b]	δ_C, $\Delta\delta$[c]	δ_C, exptl[a]	δ_C, (adj_calcd)[b]	δ_C, $\Delta\delta$[c]
1	166.3	171.043825	4.743825	166.3	171.483125	5.183125
2	96.3	100.199725	3.899725	96.3	99.128625	2.828625
3	163.3	170.913625	7.613625	163.3	170.542225	7.242225
4	103.3	106.859625	3.559625	103.3	106.926425	3.626425
5	162.5	168.844825	6.344825	162.5	166.049925	3.549925
6	108.2	111.398525	3.198525	108.2	108.898525	0.698525
7	198.7	203.763725	5.063725	198.7	203.106525	4.406525
8	42.7	46.449125	3.749125	42.7	42.198225	0.501775
9	75.5	80.828325	5.328325	75.5	77.010825	1.510825
10	119.2	125.355125	6.155125	119.2	123.709825	4.509825
11	159.3	164.003925	4.703925	159.3	167.384725	8.084725
12	99.8	100.914825	1.114825	99.8	100.925325	1.125325
13	160.5	165.102825	4.602825	160.5	166.382525	5.882525
14	108	110.161425	2.161425	108	110.020125	2.020125
15	129.1	133.390725	4.290725	129.1	136.618825	7.518825
16	55.9	56.531725	0.631725	55.9	56.794625	0.894625
17	26	24.211525	1.788475	26	26.493925	0.493925
18	48.9	52.711225	3.811225	48.9	51.699825	2.799825
19	28.5	33.657225	5.157225	28.5	30.476525	1.976525
20	126.6	131.240725	4.640725	126.6	132.480325	5.880325
21	136.8	148.332625	11.532625	136.8	147.145525	10.345525
22	65.7	71.988225	6.288225	65.7	68.794725	3.094725
23	80.3	81.320825	1.020825	80.3	82.672825	2.372825
24	24.3	30.385925	6.085925	24.3	23.887525	0.412475
25	22.5	23.034125	0.534125	22.5	24.126725	1.626725
26	20.9	26.034925	5.134925	20.9	23.447425	2.547425

no.		**2S, 2"S-1 Conf. 11**	
	δ_C, exptl[a]	δ_C, (adj_calcd)[b]	δ_C, $\Delta\delta$[c]
1	166.3	170.180225	3.880225
2	96.3	99.058825	2.758825
3	163.3	170.639125	7.339125
4	103.3	106.957525	3.657525
5	162.5	166.415025	3.915025
6	108.2	108.812325	0.612325
7	198.7	202.160325	3.460325
8	42.7	42.639225	0.060775
9	75.5	83.954325	8.454325
10	119.2	125.201225	6.001225
11	159.3	166.589825	7.289825

12	99.8	101.331525	1.531525
13	160.5	166.714825	6.214825
14	108	109.837425	1.837425
15	129.1	142.706025	13.606025
16	55.9	54.653925	1.246075
17	26	26.798525	0.798525
18	48.9	52.341625	3.441625
19	28.5	30.808225	2.308225
20	126.6	132.779725	6.179725
21	136.8	146.873025	10.073025
22	65.7	68.749225	3.049225
23	80.3	81.953025	1.653025
24	24.3	24.126925	0.173075
25	22.5	23.651725	1.151725
26	20.9	23.490025	2.590025

[a]Recorded in CD_3OD at 150 MHz. [b]Calculated in CD_3OD. [c]$\Delta\delta = |\delta_{adj\_calcd}-\delta_{exptl}|$

Table S8. Experimental chemical shifts and calculated ^{1}H NMR shifts of conformers of 2S, 2"S-1 (δ in ppm).

no.	2S, 2"S-1 Conf. 1			2S, 2"S-1 Conf. 2		
	δ_H, exptl[a]	δ_H, (adj_calcd)[b]	δ_H, $\Delta\delta$[c]	δ_H, exptl[a]	δ_H, (adj_calcd)[b]	δ_H, $\Delta\delta$[c]
1	5.94	6.173292	0.233292	5.94	6.173792	0.233792
2	2.66	2.629292	0.020708	2.66	2.649892	0.000108
3	3.13	2.560992	0.569008	3.13	2.562592	0.567408
4	5.61	5.487792	0.122208	5.61	5.515092	0.094908
5	6.48	6.506992	0.026992	6.48	6.470092	0.009908
6	6.43	6.644692	0.214692	6.43	6.616892	0.186892
7	7.32	7.778592	0.458592	7.32	7.889592	0.569592
8	3.81	4.124392	0.314392	3.81	3.685492	0.124508
9	3.81	3.722892	0.087108	3.81	3.647192	0.162808
10	3.81	3.648092	0.161908	3.81	4.113692	0.303692
11	2.2	2.774892	0.574892	2.2	3.122592	0.922592
12	2.40	2.528192	0.138192	2.40	2.654592	0.264592
13	2.2	2.213992	0.013992	2.2	1.737292	0.462708
14	1.75	2.134492	0.384492	1.75	1.838292	0.088292
15	2.18	2.705792	0.525792	2.18	2.358492	0.178492
16	5.29	5.784592	0.494592	5.29	5.694792	0.404792
17	3.68	4.394492	0.714492	3.68	4.139792	0.459792
18	4.27	4.527892	0.257892	4.27	4.422792	0.152792
19	1.08	0.787692	0.292308	1.08	1.768492	0.688492
20	1.08	1.336992	0.256992	1.08	1.113192	0.033192
21	1.08	1.044192	0.035808	1.08	0.865392	0.214608
22	1.18	1.246392	0.066392	1.18	0.999092	0.180908
23	1.18	1.767092	0.587092	1.18	1.689792	0.509792
24	1.18	1.327292	0.147292	1.18	0.993092	0.186908
25	1.52	1.678292	0.158292	1.52	1.868792	0.348792

26	1.52	1.775092	0.255092	1.52	1.662092	0.142092
27	1.52	1.625492	0.105492	1.52	1.722692	0.202692
no.	**2S, 2"S-1 Conf. 3**			**2S, 2"S-1 Conf. 4**		
	δ_H, exptl[a]	δ_H, (adj_calcd)[b]	δ_H, $\Delta\delta$[c]	δ_H, exptl[a]	δ_H, (adj_calcd)[b]	δ_H, $\Delta\delta$[c]
1	5.94	6.231992	0.291992	5.94	6.205692	0.265692
2	2.66	2.587792	0.062208	2.66	2.657992	0.007992
3	3.13	2.597392	0.532608	3.13	2.622992	0.507008
4	5.61	5.620392	0.010392	5.61	5.515292	0.094708
5	6.48	6.511492	0.031492	6.48	6.546192	0.066192
6	6.43	6.621492	0.191492	6.43	6.687092	0.257092
7	7.32	7.869192	0.549192	7.32	7.877692	0.557692
8	3.81	4.110292	0.300292	3.81	4.114692	0.304692
9	3.81	3.721692	0.088308	3.81	3.750592	0.059408
10	3.81	3.660192	0.149808	3.81	3.649492	0.160508
11	2.2	2.679092	0.479092	2.2	1.902392	0.297608
12	2.40	1.700692	0.689308	2.40	2.862692	0.472692
13	2.2	1.858892	0.341108	2.2	1.812592	0.387408
14	1.75	2.477492	0.727492	1.75	2.740192	0.990192
15	2.18	1.449992	0.730008	2.18	1.984792	0.195208
16	5.29	5.659892	0.369892	5.29	5.800192	0.510192
17	3.68	3.701892	0.021892	3.68	3.928292	0.248292
18	4.27	4.133892	0.136108	4.27	4.450692	0.180692
19	1.08	0.653692	0.426308	1.08	1.486392	0.406392
20	1.08	1.089392	0.009392	1.08	0.947492	0.132508
21	1.08	1.448492	0.368492	1.08	1.679792	0.599792
22	1.18	0.938492	0.241508	1.18	0.907192	0.272808
23	1.18	1.323992	0.143992	1.18	1.411992	0.231992
24	1.18	0.914992	0.265008	1.18	0.960192	0.219808
25	1.52	1.434492	0.085508	1.52	1.570792	0.050792
26	1.52	1.519292	0.000708	1.52	1.633392	0.113392
27	1.52	1.518392	0.001608	1.52	1.614892	0.094892
no.	**2S, 2"S-1 Conf. 5**			**2S, 2"S-1 Conf. 6**		
	δ_H, exptl[a]	δ_H, (adj_calcd)[b]	δ_H, $\Delta\delta$[c]	δ_H, exptl[a]	δ_H, (adj_calcd)[b]	δ_H, $\Delta\delta$[c]
1	5.94	6.132492	0.192492	5.94	6.158892	0.218892
2	2.66	2.234892	0.415108	2.66	2.274792	0.375208
3	3.13	3.914592	0.784592	3.13	3.969492	0.839492
4	5.61	5.041492	0.568508	5.61	5.046492	0.563508
5	6.48	6.571092	0.091092	6.48	6.603692	0.123692
6	6.43	6.508492	0.078492	6.43	6.505592	0.075592
7	7.32	7.338192	0.018192	7.32	7.325592	0.005592
8	3.81	4.148492	0.338492	3.81	4.197992	0.387992
9	3.81	3.737892	0.072108	3.81	3.761192	0.048808
10	3.81	3.690392	0.119608	3.81	3.723492	0.086508
11	2.2	2.729192	0.529192	2.2	1.749592	0.450408

12	2.40	2.394892	0.004892	2.40	2.616792	0.226792
13	2.2	2.006392	0.193608	2.2	1.662592	0.537408
14	1.75	1.933792	0.183792	1.75	2.352592	0.602592
15	2.18	2.520392	0.340392	2.18	1.950992	0.229008
16	5.29	5.709992	0.419992	5.29	5.832092	0.542092
17	3.68	4.306492	0.626492	3.68	3.910792	0.230792
18	4.27	4.462492	0.192492	4.27	4.442092	0.172092
19	1.08	0.770392	0.309608	1.08	0.945592	0.134408
20	1.08	1.346792	0.266792	1.08	1.662892	0.582892
21	1.08	1.004992	0.075008	1.08	1.466492	0.386492
22	1.18	1.177492	0.002508	1.18	0.795992	0.384008
23	1.18	1.652592	0.472592	1.18	1.193992	0.013992
24	1.18	1.233192	0.053192	1.18	0.886192	0.293808
25	1.52	1.595792	0.075792	1.52	1.557792	0.037792
26	1.52	1.638092	0.118092	1.52	1.606192	0.086192
27	1.52	1.743392	0.223392	1.52	1.599792	0.079792

no.	**2S, 2"S-1 Conf. 7**			**2S, 2"S-1 Conf. 8**		
	δ_H, exptl[a]	δ_H, (adj_calcd)[b]	δ_H, $\Delta\delta$[c]	δ_H, exptl[a]	δ_H, (adj_calcd)[b]	δ_H, $\Delta\delta$[c]
1	5.94	6.187692	0.247692	5.94	6.018292	0.078292
2	2.66	2.268592	0.381408	2.66	3.382692	0.732692
3	3.13	3.922292	0.792292	3.13	2.682592	0.447408
4	5.61	5.057092	0.552908	5.61	6.080092	0.470092
5	6.48	6.587192	0.107192	6.48	6.518392	0.038392
6	6.43	6.529992	0.099992	6.43	6.343192	0.086808
7	7.32	7.367792	0.047792	7.32	7.230392	0.089608
8	3.81	3.724092	0.085908	3.81	4.241592	0.431592
9	3.81	3.717992	0.092008	3.81	3.621892	0.188108
10	3.81	4.134792	0.324792	3.81	3.836092	0.026092
11	2.2	2.415692	0.215692	2.2	2.654192	0.454192
12	2.40	1.613392	0.776608	2.40	2.488492	0.098492
13	2.2	1.750792	0.449208	2.2	1.920892	0.279108
14	1.75	2.490192	0.740192	1.75	2.231292	0.481292
15	2.18	1.439092	0.740908	2.18	2.665092	0.485092
16	5.29	5.683492	0.393492	5.29	5.834392	0.544392
17	3.68	3.768092	0.088092	3.68	4.252092	0.572092
18	4.27	4.204192	0.065808	4.27	4.381692	0.111692
19	1.08	0.407592	0.672408	1.08	0.713892	0.366108
20	1.08	0.902292	0.177708	1.08	0.608692	0.471308
21	1.08	0.991392	0.088608	1.08	0.13921	1.219208
22	1.18	1.291092	0.111092	1.18	1.032492	0.147508
23	1.18	0.828892	0.351108	1.18	1.595192	0.415192
24	1.18	0.853092	0.326908	1.18	1.106392	0.073608
25	1.52	1.512392	0.007608	1.52	1.616692	0.096692
26	1.52	1.563092	0.043092	1.52	1.639392	0.119392

27	1.52	1.558992	0.038992	1.52	1.768092	0.248092

no.	2S, 2"S-1 Conf. 9			2S, 2"S-1 Conf. 10		
	δ_H, exptl[a]	δ_H, (adj_calcd)[b]	δ_H, $\Delta\delta$[c]	δ_H, exptl[a]	δ_H, (adj_calcd)[b]	δ_H, $\Delta\delta$[c]
1	5.94	6.230092	0.290092	5.94	6.057792	0.117792
2	2.66	2.776792	0.126792	2.66	3.387092	0.737092
3	3.13	2.558392	0.571608	3.13	2.684492	0.445508
4	5.61	5.423092	0.186908	5.61	6.130392	0.520392
5	6.48	6.514392	0.034392	6.48	6.357992	0.122008
6	6.43	6.681792	0.251792	6.43	6.571392	0.141392
7	7.32	7.997992	0.677992	7.32	7.342692	0.022692
8	3.81	3.727492	0.082508	3.81	4.324292	0.514292
9	3.81	3.633792	0.176208	3.81	3.665592	0.144408
10	3.81	4.112992	0.302992	3.81	3.888192	0.078192
11	2.2	2.410292	0.210292	2.2	1.748492	0.451508
12	2.40	3.328692	0.938692	2.40	2.836892	0.446892
13	2.2	1.640992	0.559008	2.2	1.580692	0.619308
14	1.75	2.258692	0.508692	1.75	2.157192	0.407192
15	2.18	2.431492	0.251492	2.18	0.844292	1.335708
16	5.29	5.945092	0.655092	5.29	5.108992	0.181008
17	3.68	4.140692	0.460692	3.68	3.801592	0.121592
18	4.27	4.373792	0.103792	4.27	4.332892	0.062892
19	1.08	1.095992	0.015992	1.08	0.941892	0.138108
20	1.08	1.916392	0.836392	1.08	1.608892	0.528892
21	1.08	1.307092	0.227092	1.08	1.428392	0.348392
22	1.18	1.206292	0.026292	1.18	0.970892	0.209108
23	1.18	1.810192	0.630192	1.18	1.441192	0.261192
24	1.18	1.260392	0.080392	1.18	0.987792	0.192208
25	1.52	1.946592	0.426592	1.52	1.438392	0.081608
26	1.52	1.854592	0.334592	1.52	1.505092	0.014908
27	1.52	1.735592	0.215592	1.52	1.422092	0.097908

no.	2S, 2"S-1 Conf. 11		
	δ_H, exptl[a]	δ_H, (adj_calcd)[b]	δ_H, $\Delta\delta$[c]
1	5.94	6.092492	0.152492
2	2.66	3.362892	0.712892
3	3.13	2.894092	0.235908
4	5.61	5.699992	0.089992
5	6.48	6.408492	0.071508
6	6.43	6.567692	0.137692
7	7.32	7.592192	0.272192
8	3.81	3.868892	0.058892
9	3.81	3.360792	0.449208
10	3.81	3.475892	0.334108
11	2.2	1.699492	0.500508
12	2.40	2.670692	0.280692

13	2.2	1.544392	0.655608
14	1.75	1.896392	0.146392
15	2.18	0.770892	1.409108
16	5.29	5.021292	0.268708
17	3.68	3.755592	0.075592
18	4.27	4.305892	0.035892
19	1.08	0.900792	0.179208
20	1.08	1.592892	0.512892
21	1.08	1.435792	0.355792
22	1.18	0.906892	0.273108
23	1.18	0.898792	0.281208
24	1.18	1.304992	0.124992
25	1.52	1.487592	0.032408
26	1.52	1.428992	0.091008
27	1.52	1.431892	0.088108

[a]Recorded in CD_3OD at 600 MHz. [b]Calculated in CD_3OD. [c]$\Delta\delta = |\delta_{adj\_calcd}-\delta_{exptl}|$

Table S9. Experimental chemical shifts and calculated ^{13}C NMR shifts of conformers of 2S, 2"R-1 (δ in ppm).

no.	2S, 2"R-1 Conf. 1			2S, 2"S-1 Conf. 2		
	δ_C, exptl[a]	δ_C, (adj_calcd)[b]	δ_C, $\Delta\delta$[c]	δ_C, exptl[a]	δ_C, (adj_calcd)[b]	δ_C, $\Delta\delta$[c]
1	166.3	170.775025	4.475025	166.3	170.695125	4.395125
2	96.3	98.823425	2.523425	96.3	99.452925	3.152925
3	163.3	170.259925	6.959925	163.3	170.494925	7.194925
4	103.3	106.967525	3.667525	103.3	106.591725	3.291725
5	162.5	169.276125	6.776125	162.5	168.348925	5.848925
6	108.2	111.748125	3.548125	108.2	111.819425	3.619425
7	198.7	202.927825	4.227825	198.7	203.130925	4.430925
8	42.7	47.347525	4.647525	42.7	46.551425	3.851425
9	75.5	79.475425	3.975425	75.5	79.567225	4.067225
10	119.2	125.892225	6.692225	119.2	125.424625	6.224625
11	159.3	164.147925	4.847925	159.3	163.545925	4.245925
12	99.8	100.854425	1.054425	99.8	100.191425	0.391425
13	160.5	165.407025	4.907025	160.5	164.962225	4.462225
14	108	110.097825	2.097825	108	111.027925	3.027925
15	129.1	134.726825	5.626825	129.1	133.590625	4.490625
16	55.9	56.653725	0.753725	55.9	56.421825	0.521825
17	26	28.962025	2.962025	26	28.919525	2.919525
18	48.9	52.815725	3.915725	48.9	52.508525	3.608525
19	28.5	30.713225	2.213225	28.5	30.708525	2.208525
20	126.6	133.474525	6.874525	126.6	133.268525	6.668525
21	136.8	147.220525	10.420525	136.8	147.264425	10.464425
22	65.7	69.081625	3.381625	65.7	68.991125	3.291125
23	80.3	83.279825	2.979825	80.3	83.071725	2.771725
24	24.3	24.419725	0.119725	24.3	24.115625	0.184375
25	22.5	24.378125	1.878125	22.5	23.470425	0.970425

26	20.9	23.771025	2.871025	20.9	23.590525	2.690525

no.		**2S, 2"R-1 Conf. 3**			**2S, 2"R-1 Conf. 4**	
	δ_C, exptl[a]	δ_C, (adj_calcd)[b]	δ_C, $\Delta\delta$[c]	δ_C, exptl[a]	δ_C, (adj_calcd)[b]	δ_C, $\Delta\delta$[c]
1	166.3	172.268325	5.968325	166.3	174.294325	7.994325
2	96.3	100.039325	3.739325	96.3	101.141525	4.841525
3	163.3	170.475125	7.175125	163.3	170.836525	7.536525
4	103.3	106.594125	3.294125	103.3	106.375725	3.075725
5	162.5	168.816525	6.316525	162.5	168.914925	6.414925
6	108.2	110.857625	2.657625	108.2	112.672225	4.472225
7	198.7	202.771425	4.071425	198.7	202.716825	4.016825
8	42.7	46.837125	4.137125	42.7	46.865825	4.165825
9	75.5	79.880425	4.380425	75.5	80.155525	4.655525
10	119.2	125.680425	6.480425	119.2	125.759525	6.559525
11	159.3	163.999325	4.699325	159.3	163.936725	4.636725
12	99.8	100.798125	0.998125	99.8	100.797225	0.997225
13	160.5	165.293425	4.793425	160.5	165.221025	4.721025
14	108	109.942525	1.942525	108	109.680025	1.680025
15	129.1	133.291525	4.191525	129.1	132.895325	3.795325
16	55.9	56.688125	0.788125	55.9	56.587125	0.687125
17	26	24.311725	1.688275	26	24.882525	1.117475
18	48.9	50.615525	1.715525	48.9	52.325525	3.425525
19	28.5	32.373325	3.873325	28.5	33.484325	4.984325
20	126.6	129.317625	2.717625	126.6	133.863325	7.263325
21	136.8	154.339225	17.539225	136.8	143.689025	6.889025
22	65.7	71.377225	5.677225	65.7	71.998625	6.298625
23	80.3	82.195125	1.895125	80.3	84.745125	4.445125
24	24.3	31.043325	6.743325	24.3	30.386825	6.086825
25	22.5	23.859325	1.359325	22.5	23.925125	1.425125
26	20.9	26.009825	5.109825	20.9	25.009025	4.109025

no.		**2S, 2"R-1 Conf. 5**			**2S, 2"R-1 Conf. 6**	
	δ_C, exptl[a]	δ_C, (adj_calcd)[b]	δ_C, $\Delta\delta$[c]	δ_C, exptl[a]	δ_C, (adj_calcd)[b]	δ_C, $\Delta\delta$[c]
1	166.3	170.528525	4.228525	166.3	170.529325	4.229325
2	96.3	98.798625	2.498625	96.3	98.799925	2.499925
3	163.3	170.515025	7.215025	163.3	170.515925	7.215925
4	103.3	106.425025	3.125025	103.3	106.426025	3.126025
5	162.5	168.762425	6.262425	162.5	168.767125	6.267125
6	108.2	111.159125	2.959125	108.2	111.159525	2.959525
7	198.7	203.998525	5.298525	198.7	203.997525	5.297525
8	42.7	43.492925	0.792925	42.7	43.494325	0.794325
9	75.5	85.491025	9.991025	75.5	85.500825	10.000825
10	119.2	123.040525	3.840525	119.2	123.037925	3.837925
11	159.3	168.342225	9.042225	159.3	168.342825	9.042825
12	99.8	102.149525	2.349525	99.8	102.150725	2.350725
13	160.5	166.609925	6.109925	160.5	166.611125	6.111125

14	108	109.193425	1.193425	108	109.194925	1.194925
15	129.1	138.966625	9.866625	129.1	138.966425	9.866425
16	55.9	56.326625	0.426625	55.9	56.328625	0.428625
17	26	28.424825	2.424825	26	28.423225	2.423225
18	48.9	51.950725	3.050725	48.9	51.949725	3.049725
19	28.5	30.910525	2.410525	28.5	30.909625	2.409625
20	126.6	133.474825	6.874825	126.6	133.474225	6.874225
21	136.8	147.259225	10.459225	136.8	147.259825	10.459825
22	65.7	68.767525	3.067525	65.7	68.766125	3.066125
23	80.3	83.020625	2.720625	80.3	83.022525	2.722525
24	24.3	24.009925	0.290075	24.3	24.007625	0.292375
25	22.5	23.850025	1.350025	22.5	23.850925	1.350925
26	20.9	23.638325	2.738325	20.9	23.639125	2.739125
no.		**2S, 2"R-1 Conf. 7**			**2S, 2"R-1 Conf. 8**	
	δ_C, exptl[a]	δ_C, (adj_calcd)[b]	δ_C, $\Delta\delta$[c]	δ_C, exptl[a]	δ_C, (adj_calcd)[b]	δ_C, $\Delta\delta$[c]
1	166.3	170.514825	4.214825	166.3	174.042825	7.742825
2	96.3	98.149825	1.849825	96.3	100.874925	4.574925
3	163.3	170.116025	6.816025	163.3	170.700525	7.400525
4	103.3	106.622725	3.322725	103.3	106.240325	2.940325
5	162.5	169.199225	6.699225	162.5	168.893225	6.393225
6	108.2	111.126725	2.926725	108.2	112.350325	4.150325
7	198.7	203.722825	5.022825	198.7	203.436325	4.736325
8	42.7	43.406225	0.706225	42.7	43.798825	1.098825
9	75.5	85.248925	9.748925	75.5	85.417325	9.917325
10	119.2	122.858625	3.658625	119.2	123.377425	4.177425
11	159.3	168.643125	9.343125	159.3	168.551525	9.251525
12	99.8	102.407225	2.607225	99.8	102.358025	2.558025
13	160.5	166.637225	6.137225	160.5	166.555625	6.055625
14	108	109.207225	1.207225	108	109.111625	1.111625
15	129.1	138.583825	9.483825	129.1	138.941325	9.841325
16	55.9	56.429025	0.529025	55.9	56.365825	0.465825
17	26	28.932125	2.932125	26	25.173825	0.826175
18	48.9	51.755825	2.855825	48.9	52.364225	3.464225
19	28.5	30.915125	2.415125	28.5	32.430025	3.930025
20	126.6	133.490025	6.890025	126.6	134.420525	7.820525
21	136.8	146.924425	10.124425	136.8	143.040025	6.240025
22	65.7	68.941425	3.241425	65.7	71.826225	6.126225
23	80.3	83.122825	2.822825	80.3	85.004425	4.704425
24	24.3	24.099325	0.200675	24.3	30.628125	6.328125
25	22.5	23.259025	0.759025	22.5	24.039025	1.539025
26	20.9	23.831625	2.931625	20.9	24.946825	4.046825
no.		**2S, 2"R-1 Conf. 9**			**2S, 2"R-1 Conf. 10**	
	δ_C, exptl[a]	δ_C, (adj_calcd)[b]	δ_C, $\Delta\delta$[c]	δ_C, exptl[a]	δ_C, (adj_calcd)[b]	δ_C, $\Delta\delta$[c]
1	166.3	170.277825	3.977825	166.3	170.625625	4.325625

2	96.3	99.378125	3.078125	96.3	98.829125	2.529125
3	163.3	170.383225	7.083225	163.3	170.134725	6.834725
4	103.3	106.956725	3.656725	103.3	106.914225	3.614225
5	162.5	169.170025	6.670025	162.5	168.784425	6.284425
6	108.2	109.578025	1.378025	108.2	111.790125	3.590125
7	198.7	203.587125	4.887125	198.7	203.121525	4.421525
8	42.7	42.222025	0.477975	42.7	46.578325	3.878325
9	75.5	77.091225	1.591225	75.5	80.053125	4.553125
10	119.2	122.372625	3.172625	119.2	125.619525	6.419525
11	159.3	167.942025	8.642025	159.3	164.094825	4.794825
12	99.8	101.529125	1.729125	99.8	100.848025	1.048025
13	160.5	166.868925	6.368925	160.5	165.343925	4.843925
14	108	109.494025	1.494025	108	109.960325	1.960325
15	129.1	135.228625	6.128625	129.1	133.766825	4.666825
16	55.9	56.880225	0.980225	55.9	56.579925	0.679925
17	26	29.245725	3.245725	26	29.487925	3.487925
18	48.9	52.770325	3.870325	48.9	54.686425	5.786425
19	28.5	31.742425	3.242425	28.5	35.301225	6.801225
20	126.6	132.057525	5.457525	126.6	131.683325	5.083325
21	136.8	147.518525	10.718525	136.8	146.308725	9.508725
22	65.7	68.651225	2.951225	65.7	71.370525	5.670525
23	80.3	82.582525	2.282525	80.3	83.256425	2.956425
24	24.3	23.973725	0.326275	24.3	17.794125	6.505875
25	22.5	23.260425	0.760425	22.5	32.044625	9.544625
26	20.9	23.528925	2.628925	20.9	24.984525	4.084525
no.	**2S, 2"R-1 Conf. 11**			**2S, 2"R-1 Conf. 12**		
	δ_C, exptl[a]	δ_C, (adj_calcd)[b]	δ_C, $\Delta\delta$[c]	δ_C, exptl[a]	δ_C, (adj_calcd)[b]	δ_C, $\Delta\delta$[c]
1	166.3	169.637025	3.337025	166.3	171.246625	4.946625
2	96.3	99.469825	3.169825	96.3	98.785225	2.485225
3	163.3	170.109125	6.809125	163.3	170.135025	6.835025
4	103.3	107.273725	3.973725	103.3	106.799225	3.499225
5	162.5	169.580525	7.080525	162.5	169.079025	6.579025
6	108.2	112.075425	3.875425	108.2	113.106825	4.906825
7	198.7	203.124725	4.424725	198.7	202.932725	4.232725
8	42.7	46.321325	3.621325	42.7	47.033725	4.333725
9	75.5	79.725725	4.225725	75.5	79.726925	4.226925
10	119.2	125.446825	6.246825	119.2	125.791425	6.591425
11	159.3	163.590125	4.290125	159.3	164.032125	4.732125
12	99.8	100.211925	0.411925	99.8	100.784425	0.984425
13	160.5	164.997425	4.497425	160.5	165.277225	4.777225
14	108	111.030025	3.030025	108	110.012525	2.012525
15	129.1	133.508025	4.408025	129.1	133.873725	4.773725
16	55.9	56.337525	0.437525	55.9	56.707125	0.807125
17	26	32.196925	6.196925	26	25.149925	0.850075

18	48.9	50.718925	1.818925	48.9	51.995925	3.095925
19	28.5	36.101725	7.601725	28.5	32.851325	4.351325
20	126.6	133.104025	6.504025	126.6	132.000625	5.400625
21	136.8	146.816225	10.016225	136.8	145.556525	8.756525
22	65.7	68.711525	3.011525	65.7	71.563625	5.863625
23	80.3	82.894925	2.594925	80.3	82.119625	1.819625
24	24.3	24.695425	0.395425	24.3	31.285625	6.985625
25	22.5	25.978725	3.478725	22.5	23.940625	1.440625
26	20.9	23.237025	2.337025	20.9	25.608825	4.708825

no.	2S, 2"R-1 Conf. 13			2S, 2"R-1 Conf. 14		
	δ_C, exptl[a]	δ_C, (adj_calcd)[b]	δ_C, $\Delta\delta$[c]	δ_C, exptl[a]	δ_C, (adj_calcd)[b]	δ_C, $\Delta\delta$[c]
1	166.3	170.707425	4.407425	166.3	171.3097	5.0097
2	96.3	98.800825	2.500825	96.3	99.38135	3.08135
3	163.3	170.053525	6.753525	163.3	170.4099	7.1099
4	103.3	106.816025	3.516025	103.3	106.7796	3.4796
5	162.5	168.858125	6.358125	162.5	168.9737	6.4737
6	108.2	113.443725	5.243725	108.2	111.693	3.493
7	198.7	202.942525	4.242525	198.7	203.0291	4.3291
8	42.7	46.829425	4.129425	42.7	46.65441	3.95441
9	75.5	79.893725	4.393725	75.5	80.30234	4.80234
10	119.2	125.614025	6.414025	119.2	125.4304	6.2304
11	159.3	163.956525	4.656525	159.3	164.541	5.241
12	99.8	100.841125	1.041125	99.8	100.9283	1.1283
13	160.5	165.311925	4.811925	160.5	165.457	4.957
14	108	110.006025	2.006025	108	110.1414	2.1414
15	129.1	133.624225	4.524225	129.1	134.6453	5.5453
16	55.9	56.694125	0.794125	55.9	56.64556	0.74556
17	26	26.189225	0.189225	26	27.92951	1.92951
18	48.9	53.610425	4.710425	48.9	52.39849	3.49849
19	28.5	30.044125	1.544125	28.5	31.40351	2.90351
20	126.6	132.242125	5.642125	126.6	132.8861	6.2861
21	136.8	144.910425	8.110425	136.8	147.8392	11.0392
22	65.7	70.129725	4.429725	65.7	69.75744	4.05744
23	80.3	83.165825	2.865825	80.3	83.26759	2.96759
24	24.3	22.929925	1.370075	24.3	25.91398	1.61398
25	22.5	33.306425	10.806425	22.5	24.21716	1.71716
26	20.9	23.768125	2.868125	20.9	24.27266	3.37266

[a]Recorded in CD_3OD at 150 MHz. [b]Calculated in CD_3OD. [c]$\Delta\delta = |\delta_{adj\_calcd}-\delta_{exptl}|$

Table S10. Experimental chemical shifts and calculated ^{1}H NMR shifts of conformers of 2S, 2"R-1 (δ in ppm).

no.	2S, 2"R-1 Conf. 1			2S, 2"R-1 Conf. 2		
	δ_H, exptl[a]	δ_H, (adj_calcd)[b]	δ_H, $\Delta\delta$[c]	δ_H, exptl[a]	δ_H, (adj_calcd)[b]	δ_H, $\Delta\delta$[c]
1	5.94	6.031192	0.091192	5.94	6.027792	0.087792
2	2.66	2.638292	0.011708	2.66	2.569692	0.080308

3	3.13	2.465292	0.664708	3.13	2.650192	0.479808
4	5.61	5.627892	0.017892	5.61	5.559592	0.050408
5	6.48	6.534592	0.054592	6.48	6.351792	0.128208
6	6.43	6.683792	0.253792	6.43	6.824992	0.394992
7	7.32	7.888492	0.568492	7.32	7.875692	0.555692
8	3.81	4.104792	0.294792	3.81	4.142392	0.332392
9	3.81	3.737392	0.072608	3.81	3.737592	0.072408
10	3.81	3.648892	0.161108	3.81	3.654292	0.155708
11	2.2	2.438092	0.238092	2.2	2.609292	0.409292
12	2.40	2.186892	0.203108	2.40	2.158192	0.231808
13	2.2	2.301092	0.101092	2.2	2.056892	0.143108
14	1.75	2.477992	0.727992	1.75	2.180392	0.430392
15	2.18	1.700392	0.479608	2.18	1.506592	0.673408
16	5.29	5.779292	0.489292	5.29	5.619892	0.329892
17	3.68	3.929692	0.249692	3.68	3.855292	0.175292
18	4.27	4.600392	0.330392	4.27	4.445192	0.175192
19	1.08	1.375792	0.295792	1.08	1.269792	0.189792
20	1.08	0.897792	0.182208	1.08	0.978792	0.101208
21	1.08	0.892292	0.187708	1.08	0.887692	0.192308
22	1.18	1.205092	0.025092	1.18	1.547992	0.367992
23	1.18	1.674992	0.494992	1.18	1.673492	0.493492
24	1.18	0.698492	0.481508	1.18	0.784292	0.395708
25	1.52	1.632092	0.112092	1.52	1.510092	0.009908
26	1.52	1.629292	0.109292	1.52	1.525792	0.005792
27	1.52	1.654092	0.134092	1.52	1.547492	0.027492

no.	**2S, 2"R-1 Conf. 3**			**2S, 2"R-1 Conf. 4**		
	δ_H, exptl[a]	δ_H, (adj_calcd)[b]	δ_H, $\Delta\delta$[c]	δ_H, exptl[a]	δ_H, (adj_calcd)[b]	δ_H, $\Delta\delta$[c]
1	5.94	6.181792	0.241792	5.94	6.102492	0.162492
2	2.66	2.570492	0.079508	2.66	2.614992	0.035008
3	3.13	2.603792	0.526208	3.13	2.658692	0.471308
4	5.61	5.544292	0.065708	5.61	5.481592	0.128408
5	6.48	6.491892	0.011892	6.48	6.505492	0.025492
6	6.43	6.646392	0.216392	6.43	6.645192	0.215192
7	7.32	7.778792	0.458792	7.32	7.839692	0.519692
8	3.81	3.642792	0.167208	3.81	3.633092	0.176908
9	3.81	4.123692	0.313692	3.81	4.115892	0.305892
10	3.81	3.720092	0.089908	3.81	3.739992	0.070008
11	2.2	2.651192	0.451192	2.2	2.539792	0.339792
12	2.40	2.930892	0.540892	2.40	3.089492	0.699492
13	2.2	2.071492	0.128508	2.2	2.300292	0.100292
14	1.75	2.525392	0.775392	1.75	2.618292	0.868292
15	2.18	1.967392	0.212608	2.18	2.608492	0.428492
16	5.29	5.744092	0.454092	5.29	5.463692	0.173692
17	3.68	4.183692	0.503692	3.68	4.080192	0.400192

no.						
18	4.27	4.456892	0.186892	4.27	4.403292	0.133292
19	1.08	1.555592	0.475592	1.08	1.840292	0.760292
20	1.08	1.050792	0.029208	1.08	1.032892	0.047108
21	1.08	1.116292	0.036292	1.08	1.246292	0.166292
22	1.18	1.293192	0.113192	1.18	1.330792	0.150792
23	1.18	1.836192	0.656192	1.18	1.268592	0.088592
24	1.18	1.132892	0.047108	1.18	1.819492	0.639492
25	1.52	1.682392	0.162392	1.52	1.314292	0.205708
26	1.52	1.875592	0.355592	1.52	1.535492	0.015492
27	1.52	1.741692	0.221692	1.52	1.492292	0.027708

no.	**2S, 2"R-1 Conf. 5**			**2S, 2"R-1 Conf. 6**		
	δ_H, exptl[a]	δ_H, (adj_calcd)[b]	δ_H, $\Delta\delta$[c]	δ_H, exptl[a]	δ_H, (adj_calcd)[b]	δ_H, $\Delta\delta$[c]
1	5.94	5.974492	0.034492	5.94	5.974592	0.034592
2	2.66	3.943192	1.293192	2.66	3.942592	1.292592
3	3.13	2.293992	0.836008	3.13	2.294292	0.835708
4	5.61	5.069692	0.540308	5.61	5.069592	0.540408
5	6.48	6.602092	0.122092	6.48	6.602192	0.122192
6	6.43	6.530392	0.100392	6.43	6.530392	0.100392
7	7.32	7.369192	0.049192	7.32	7.369092	0.049092
8	3.81	4.117392	0.307392	3.81	4.117492	0.307492
9	3.81	3.680292	0.129708	3.81	3.680292	0.129708
10	3.81	3.748192	0.061808	3.81	3.748292	0.061708
11	2.2	2.384592	0.184592	2.2	2.384692	0.184692
12	2.40	2.069292	0.320708	2.40	2.069292	0.320708
13	2.2	2.054992	0.145008	2.2	2.055092	0.144908
14	1.75	2.178492	0.428492	1.75	2.178092	0.428092
15	2.18	1.545492	0.634508	2.18	1.545092	0.634908
16	5.29	5.692192	0.402192	5.29	5.692292	0.402292
17	3.68	3.821492	0.141492	3.68	3.821292	0.141292
18	4.27	4.410992	0.140992	4.27	4.411092	0.141092
19	1.08	0.816092	0.263908	1.08	0.815992	0.264008
20	1.08	0.778392	0.301608	1.08	0.778592	0.301408
21	1.08	1.138492	0.058492	1.08	1.138392	0.058392
22	1.18	1.469392	0.289392	1.18	1.469592	0.289592
23	1.18	1.605092	0.425092	1.18	1.605292	0.425292
24	1.18	0.733292	0.446708	1.18	0.733392	0.446608
25	1.52	1.561592	0.041592	1.52	1.561692	0.041692
26	1.52	1.581892	0.061892	1.52	1.581892	0.061892
27	1.52	1.532292	0.012292	1.52	1.532292	0.012292

no.	**2S, 2"R-1 Conf. 7**			**2S, 2"R-1 Conf. 8**		
	δ_H, exptl[a]	δ_H, (adj_calcd)[b]	δ_H, $\Delta\delta$[c]	δ_H, exptl[a]	δ_H, (adj_calcd)[b]	δ_H, $\Delta\delta$[c]
1	5.94	5.987492	0.047492	5.94	6.070692	0.130692
2	2.66	3.911592	1.261592	2.66	3.982192	1.332192
3	3.13	2.237092	0.892908	3.13	2.253692	0.876308

4	5.61	5.105192	0.504808	5.61	5.030892	0.579108
5	6.48	6.615492	0.135492	6.48	6.642092	0.162092
6	6.43	6.512692	0.082692	6.43	6.525292	0.095292
7	7.32	7.338092	0.018092	7.32	7.342392	0.022392
8	3.81	4.065792	0.255792	3.81	3.724092	0.085908
9	3.81	3.781392	0.028608	3.81	4.189592	0.379592
10	3.81	3.675992	0.134008	3.81	3.757592	0.052408
11	2.2	2.328092	0.128092	2.2	2.500092	0.300092
12	2.40	2.056492	0.333508	2.40	2.934992	0.544992
13	2.2	2.162092	0.037908	2.2	2.021092	0.178908
14	1.75	2.408092	0.658092	1.75	2.379792	0.629792
15	2.18	1.659692	0.520308	2.18	2.681992	0.501992
16	5.29	5.774792	0.484792	5.29	5.558292	0.268292
17	3.68	3.849892	0.169892	3.68	4.008792	0.328792
18	4.27	4.403992	0.133992	4.27	4.344092	0.074092
19	1.08	0.798992	0.281008	1.08	1.802992	0.722992
20	1.08	0.813392	0.266608	1.08	0.946892	0.133108
21	1.08	1.295692	0.215692	1.08	1.230992	0.150992
22	1.18	1.015892	0.164108	1.18	1.284192	0.104192
23	1.18	1.107192	0.072808	1.18	1.163692	0.016308
24	1.18	0.489892	0.690108	1.18	1.677292	0.497292
25	1.52	1.622392	0.102392	1.52	1.467492	0.052508
26	1.52	1.628192	0.108192	1.52	1.447992	0.072008
27	1.52	1.616292	0.096292	1.52	1.293092	0.226908

no.		**2S, 2"R-1 Conf. 9**			**2S, 2"R-1 Conf. 10**	
	δ_H, exptl[a]	δ_H, (adj_calcd)[b]	δ_H, $\Delta\delta$[c]	δ_H, exptl[a]	δ_H, (adj_calcd)[b]	δ_H, $\Delta\delta$[c]
1	5.94	6.202492	0.262492	5.94	6.033492	0.093492
2	2.66	3.338992	0.688992	2.66	2.608392	0.041608
3	3.13	2.406792	0.723208	3.13	2.643392	0.486608
4	5.61	5.531892	0.078108	5.61	5.586192	0.023808
5	6.48	6.585792	0.105792	6.48	6.555492	0.075492
6	6.43	6.609592	0.179592	6.43	6.686192	0.256192
7	7.32	7.537192	0.217192	7.32	7.982092	0.662092
8	3.81	3.856092	0.046092	3.81	4.123492	0.313492
9	3.81	4.156192	0.346192	3.81	3.759092	0.050908
10	3.81	3.639492	0.170508	3.81	3.668892	0.141108
11	2.2	1.664992	0.535008	2.2	2.372992	0.172992
12	2.40	2.542692	0.152692	2.40	2.595192	0.205192
13	2.2	1.764892	0.435108	2.2	2.292492	0.092492
14	1.75	1.415492	0.334508	1.75	2.221992	0.471992
15	2.18	2.484292	0.304292	2.18	2.083692	0.096308
16	5.29	5.650392	0.360392	5.29	5.767492	0.477492
17	3.68	3.717792	0.037792	3.68	4.115192	0.435192
18	4.27	4.137092	0.132908	4.27	4.355592	0.085592

19	1.08	1.292692	0.212692	1.08	1.439992	0.359992
20	1.08	0.898592	0.181408	1.08	1.132992	0.052992
21	1.08	0.853792	0.226208	1.08	1.017792	0.062208
22	1.18	0.882392	0.297608	1.18	1.235292	0.055292
23	1.18	0.462892	0.717108	1.18	1.345392	0.165392
24	1.18	1.152992	0.027008	1.18	1.073392	0.106608
25	1.52	1.519792	0.000208	1.52	1.549092	0.029092
26	1.52	1.519992	0.0000083	1.52	1.606492	0.086492
27	1.52	1.449492	0.070508	1.52	1.697892	0.177892

no.		**2S, 2"R-1 Conf. 11**			**2S, 2"R-1 Conf. 12**	
	δ_H, exptl[a]	δ_H, (adj_calcd)[b]	δ_H, $\Delta\delta$[c]	δ_H, exptl[a]	δ_H, (adj_calcd)[b]	δ_H, $\Delta\delta$[c]
1	5.94	6.036292	0.096292	5.94	6.024892	0.084892
2	2.66	2.558392	0.091608	2.66	2.591292	0.058708
3	3.13	2.658592	0.471408	3.13	2.543692	0.586308
4	5.61	5.596492	0.013508	5.61	5.572492	0.037508
5	6.48	6.333892	0.146108	6.48	6.507192	0.027192
6	6.43	6.817192	0.387192	6.43	6.645192	0.215192
7	7.32	7.892992	0.572992	7.32	7.794292	0.474292
8	3.81	3.726192	0.083808	3.81	3.643692	0.166308
9	3.81	3.634492	0.175508	3.81	4.119892	0.309892
10	3.81	4.118292	0.308292	3.81	3.729992	0.080008
11	2.2	2.477792	0.277792	2.2	3.021592	0.821592
12	2.40	2.890092	0.500092	2.40	2.603392	0.213392
13	2.2	2.452792	0.252792	2.2	2.041492	0.158508
14	1.75	2.656492	0.906492	1.75	2.393692	0.643692
15	2.18	1.617792	0.562208	2.18	2.038792	0.141208
16	5.29	5.770492	0.480492	5.29	5.747192	0.457192
17	3.68	4.134992	0.454992	3.68	4.071792	0.391792
18	4.27	3.680492	0.589508	4.27	4.373492	0.103492
19	1.08	0.785292	0.294708	1.08	1.559692	0.479692
20	1.08	1.350492	0.270492	1.08	1.076492	0.003508
21	1.08	1.607792	0.527792	1.08	1.042492	0.037508
22	1.18	0.517992	0.662008	1.18	1.296892	0.116892
23	1.18	0.554792	0.625208	1.18	1.789592	0.609592
24	1.18	1.151192	0.028808	1.18	1.145092	0.034908
25	1.52	1.410092	0.109908	1.52	1.576692	0.056692
26	1.52	1.446492	0.073508	1.52	1.774892	0.254892
27	1.52	1.241392	0.278608	1.52	1.621192	0.101192

no.		**2S, 2"R-1 Conf. 13**			**2S, 2"R-1 Conf. 13**	
	δ_H, exptl[a]	δ_H, (adj_calcd)[b]	δ_H, $\Delta\delta$[c]	δ_H, exptl[a]	δ_H, (adj_calcd)[b]	δ_H, $\Delta\delta$[c]
1	5.94	5.973692	0.033692	5.94	5.935392	0.004608
2	2.66	2.569492	0.080508	2.66	2.559092	0.090908
3	3.13	2.564692	0.565308	3.13	3.448992	0.318992
4	5.61	5.542092	0.067908	5.61	6.361392	0.751392

5	6.48	6.494492	0.014492	6.48	6.571592	0.091592
6	6.43	6.674492	0.244492	6.43	6.325792	0.104208
7	7.32	7.814892	0.494892	7.32	7.263892	0.056108
8	3.81	3.641192	0.168808	3.81	4.247292	0.437292
9	3.81	4.115892	0.305892	3.81	3.628492	0.181508
10	3.81	3.718192	0.091808	3.81	3.953592	0.143592
11	2.2	2.783492	0.583492	2.2	2.216992	0.016992
12	2.40	2.649992	0.259992	2.40	2.107592	0.282408
13	2.2	1.994992	0.205008	2.2	1.316192	0.883808
14	1.75	2.736892	0.986892	1.75	2.215092	0.465092
15	2.18	1.830292	0.349708	2.18	1.400392	0.779608
16	5.29	5.732592	0.442592	5.29	5.373792	0.083792
17	3.68	3.924792	0.244792	3.68	3.668192	0.011808
18	4.27	4.337092	0.067092	4.27	4.022792	0.247208
19	1.08	1.812092	0.732092	1.08	1.234892	0.154892
20	1.08	1.063792	0.016208	1.08	0.843192	0.236808
21	1.08	0.712592	0.367408	1.08	0.886992	0.193008
22	1.18	1.144392	0.035608	1.18	1.487792	0.307792
23	1.18	1.197292	0.017292	1.18	1.657192	0.477192
24	1.18	1.743092	0.563092	1.18	0.845792	0.334208
25	1.52	1.713992	0.193992	1.52	1.336992	0.183008
26	1.52	1.675792	0.155792	1.52	1.386092	0.133908
27	1.52	1.704992	0.184992	1.52	1.494392	0.025608

[a]Recorded in CD_3OD at 600 MHz. [b]Calculated in CD_3OD. [c]$\Delta\delta = |\delta_{adj\_calcd}-\delta_{exptl}|$

Table S11. The coordinate for the low-energy conformer 2S, 2"S-1 in NMR and ECD calculations

**2S, 2"S-1** Conf. 1		Standard Orientation (Ångstroms)		
I	atom	X	Y	Z
1	C	-2.06346	2.317608	-0.47145
2	C	-1.58598	3.627659	-0.363
3	C	-0.24274	3.853729	-0.08562
4	C	0.648538	2.751862	0.08657
5	C	0.130155	1.439364	-0.06602
6	C	-1.2121	1.186757	-0.34935
7	C	2.02376	2.96488	0.478905
8	C	2.848986	1.714198	0.717167
9	C	2.354146	0.566916	-0.16899
10	O	0.944947	0.34796	0.057523
11	O	2.520469	4.09372	0.642751
12	C	3.077167	-0.73737	0.076709
13	O	-3.38395	2.178986	-0.71981
14	O	0.193568	5.114732	0.048105
15	C	4.345816	-0.94818	-0.5065

16	C	5.049527	-2.13173	-0.28649
17	C	4.491651	-3.12308	0.52943
18	C	3.242525	-2.93373	1.119802
19	C	2.553159	-1.74145	0.88642
20	O	4.815391	0.078292	-1.2779
21	C	6.072636	-0.0733	-1.92238
22	O	5.232989	-4.25908	0.704383
23	C	-1.68286	-0.21248	-0.68368
24	C	-2.12065	-1.20441	0.437317
25	C	-2.35137	-2.57685	-0.23024
26	C	-3.54001	-2.73248	-1.15167
27	C	-4.72308	-2.09961	-1.19983
28	C	-5.24047	-0.99227	-0.29948
29	O	-4.3255	-0.15911	0.403865
30	C	-3.33841	-0.75105	1.295224
31	C	-2.97761	0.368434	2.283622
32	C	-3.95647	-1.89878	2.113243
33	C	-5.77314	-2.52214	-2.20659
34	H	-2.27115	4.460797	-0.46427
35	H	3.901426	1.930369	0.517674
36	H	2.758219	1.425973	1.773852
37	H	2.479073	0.861639	-1.21937
38	H	-3.70391	1.292916	-0.42686
39	H	1.158017	5.058067	0.287224
40	H	6.022243	-2.31462	-0.72526
41	H	2.808999	-3.70538	1.752323
42	H	1.576779	-1.59115	1.334009
43	H	6.232108	0.848819	-2.48364
44	H	6.882743	-0.20127	-1.19343
45	H	6.067979	-0.92587	-2.61316
46	H	4.747328	-4.86235	1.288045
47	H	-2.49209	-0.14481	-1.41723
48	H	-0.85154	-0.70673	-1.19693
49	H	-1.27251	-1.32389	1.125255
50	H	-1.4502	-2.82453	-0.80652
51	H	-2.40962	-3.36011	0.540137
52	H	-3.40954	-3.53462	-1.87979
53	H	-5.81734	-0.28669	-0.90947
54	H	-5.9582	-1.43222	0.412185
55	H	-2.23466	0.006256	3.002243
56	H	-2.56331	1.247166	1.791168
57	H	-3.87196	0.674139	2.836043
58	H	-4.783	-1.51772	2.722652

59	H	-4.33403	-2.71804	1.497535
60	H	-3.20335	-2.31133	2.793392
61	H	-6.72235	-2.77541	-1.71311
62	H	-5.99591	-1.7123	-2.91489
63	H	-5.4518	-3.39616	-2.77999
**2S, 2"S-1** Conf. 2		Standard Orientation (Ångstroms)		
I	atom	X	Y	Z
1	C	2.311205	2.331182	-0.46382
2	C	1.909522	3.658603	-0.286519
3	C	0.556391	3.963985	-0.202679
4	C	-0.417394	2.923345	-0.283793
5	C	0.037363	1.591621	-0.458419
6	C	1.389267	1.261265	-0.570716
7	C	-1.831934	3.230111	-0.291299
8	C	-2.768673	2.055691	-0.503992
9	C	-2.149038	0.765594	0.041671
10	O	-0.849535	0.556273	-0.551714
11	O	-2.27875	4.383691	-0.165589
12	C	-2.98522	-0.466012	-0.217381
13	O	3.652723	2.131949	-0.526306
14	O	0.183521	5.242253	-0.045639
15	C	-4.07847	-0.753267	0.628595
16	C	-4.878294	-1.874903	0.412609
17	C	-4.595324	-2.725416	-0.662938
18	C	-3.52397	-2.457082	-1.514754
19	C	-2.734533	-1.328521	-1.281636
20	O	-4.283428	0.137947	1.645331
21	C	-5.34788	-0.099098	2.556256
22	O	-5.416699	-3.807387	-0.818561
23	C	1.813942	-0.167599	-0.839931
24	C	1.885313	-1.098476	0.402207
25	C	2.778143	-0.50611	1.510008
26	C	4.263765	-0.382094	1.252535
27	C	5.096959	-1.159311	0.538106
28	C	4.739601	-2.413109	-0.243647
29	O	3.459087	-2.521917	-0.829712
30	C	2.275428	-2.552639	-0.002986
31	C	1.214173	-3.17863	-0.923114
32	C	2.452398	-3.468163	1.220298
33	C	6.584892	-0.882076	0.530481
34	H	2.653173	4.442814	-0.210794
35	H	-3.725032	2.261288	-0.016629

36	H	-2.956482	1.946273	-1.581306
37	H	-2.001573	0.884014	1.123532
38	H	3.864118	1.182738	-0.466822
39	H	-0.811529	5.249336	-0.036775
40	H	-5.719614	-2.114133	1.050535
41	H	-3.304089	-3.118018	-2.350202
42	H	-1.895102	-1.118282	-1.93515
43	H	-6.319333	-0.094555	2.046166
44	H	-5.219029	-1.053126	3.082885
45	H	-5.312149	0.720984	3.275281
46	H	-5.128185	-4.309934	-1.596239
47	H	1.102838	-0.594155	-1.550949
48	H	2.780477	-0.189644	-1.354343
49	H	0.871634	-1.149094	0.821701
50	H	2.393891	0.493004	1.747294
51	H	2.636917	-1.080757	2.436874
52	H	4.730893	0.44158	1.794717
53	H	5.424403	-2.493969	-1.096743
54	H	4.957553	-3.284794	0.398473
55	H	1.187958	-2.674525	-1.892236
56	H	1.455991	-4.230439	-1.10674
57	H	0.217612	-3.121409	-0.472458
58	H	2.70357	-4.483444	0.894254
59	H	3.234695	-3.129822	1.904712
60	H	1.515452	-3.517914	1.786337
61	H	6.944699	-0.656726	-0.482596
62	H	6.845058	-0.042344	1.181151
63	H	7.150029	-1.760763	0.872739
**2S, 2"S-1** Conf. 3		Standard Orientation (Ångstroms)		
I	atom	X	Y	Z
1	C	3.0417	2.142442	-0.750989
2	C	2.910508	3.429105	-0.224851
3	C	1.654855	3.892778	0.148758
4	C	0.509649	3.054687	-0.004071
5	C	0.693911	1.751413	-0.531555
6	C	1.945528	1.262747	-0.917309
7	C	-0.817725	3.559779	0.27817
8	C	-1.967773	2.627727	-0.04761
9	C	-1.540961	1.164068	0.096079
10	O	-0.366246	0.908876	-0.708253
11	O	-1.030359	4.699063	0.727469
12	C	-2.617682	0.189072	-0.31899
13	O	4.310655	1.779326	-1.083683

14	O	1.538068	5.134049	0.639002
15	C	-3.625578	-0.166474	0.60362
16	C	-4.648683	-1.044116	0.246177
17	C	-4.680065	-1.574049	-1.049272
18	C	-3.699428	-1.22891	-1.979524
19	C	-2.683008	-0.348785	-1.602132
20	O	-3.521283	0.411156	1.838319
21	C	-4.477575	0.07234	2.833457
22	O	-5.708036	-2.428183	-1.335194
23	C	2.077985	-0.138166	-1.484125
24	C	2.097343	-1.271863	-0.420258
25	C	3.38813	-1.241327	0.431769
26	C	3.241931	-1.820787	1.820798
27	C	2.481298	-2.860081	2.183316
28	C	1.694262	-3.697566	1.194486
29	O	2.225481	-3.716613	-0.120144
30	C	1.841695	-2.679775	-1.045545
31	C	0.366133	-2.859689	-1.443584
32	C	2.738667	-2.965353	-2.258294
33	C	2.347874	-3.29442	3.621931
34	H	3.783451	4.061001	-0.114344
35	H	-2.812154	2.847719	0.61074
36	H	-2.290341	2.817185	-1.081051
37	H	-1.266147	0.982039	1.142817
38	H	4.310752	0.870511	-1.421212
39	H	0.569071	5.283502	0.810597
40	H	-5.429705	-1.331638	0.938552
41	H	-3.724138	-1.640997	-2.985857
42	H	-1.912921	-0.082991	-2.318298
43	H	-4.187236	0.628496	3.726278
44	H	-5.490282	0.368846	2.532782
45	H	-4.463815	-1.002907	3.05165
46	H	-5.625043	-2.721158	-2.25599
47	H	1.242218	-0.30448	-2.169933
48	H	2.976175	-0.213924	-2.114775
49	H	1.260369	-1.064565	0.257692
50	H	4.206318	-1.742591	-0.107468
51	H	3.7054	-0.199319	0.551929
52	H	3.800481	-1.305728	2.602469
53	H	1.715933	-4.744325	1.526708
54	H	0.632184	-3.40206	1.201039
55	H	0.2052	-3.881875	-1.802093

56	H	-0.311824	-2.675994	-0.60403
57	H	0.078553	-2.168866	-2.242376
58	H	2.483784	-2.326504	-3.109256
59	H	3.796589	-2.823628	-2.01488
60	H	2.606764	-4.008497	-2.561127
61	H	1.296651	-3.293192	3.944957
62	H	2.71625	-4.319884	3.766555
63	H	2.903283	-2.634349	4.295091
**2S, 2"S-1** Conf. 4		Standard Orientation (Ångstroms)		
I	atom	X	Y	Z
1	C	-1.846994	2.66788	-0.887377
2	C	-1.382711	3.894647	-0.414013
3	C	-0.125568	3.965353	0.177939
4	C	0.679812	2.792793	0.289355
5	C	0.169336	1.569874	-0.219264
6	C	-1.094169	1.471501	-0.805338
7	C	1.951367	2.833169	0.98205
8	C	2.674621	1.508549	1.129215
9	C	2.337381	0.577011	-0.038666
10	O	0.903843	0.421543	-0.136832
11	O	2.430048	3.875097	1.462362
12	C	2.949999	-0.798251	0.0869
13	O	-3.080112	2.684999	-1.46351
14	O	0.305692	5.143041	0.648017
15	C	4.298489	-0.991617	-0.284058
16	C	4.901279	-2.243805	-0.171533
17	C	4.158719	-3.323332	0.321857
18	C	2.827068	-3.152936	0.699812
19	C	2.240785	-1.891182	0.578817
20	O	4.945605	0.121463	-0.744773
21	C	6.292948	-0.003988	-1.178827
22	O	4.807863	-4.523528	0.406613
23	C	-1.621119	0.168581	-1.370027
24	C	-2.73035	-0.51178	-0.511947
25	C	-2.150096	-1.068089	0.808209
26	C	-3.145009	-1.171071	1.941805
27	C	-4.443422	-1.481321	1.849654
28	C	-5.113994	-1.876865	0.548618
29	O	-4.249488	-2.453806	-0.417657
30	C	-3.521224	-1.592555	-1.312996
31	C	-4.498503	-0.94576	-2.313636
32	C	-2.601878	-2.572903	-2.053868
33	C	-5.360452	-1.46798	3.047739

34	H	-1.998076	4.781751	-0.503202
35	H	3.751494	1.686729	1.183179
36	H	2.363125	1.040254	2.073448
37	H	2.682305	1.046991	-0.969088
38	H	-3.313596	1.793638	-1.763879
39	H	1.199407	4.978032	1.054931
40	H	5.932766	-2.415085	-0.452417
41	H	2.249929	-3.992624	1.080683
42	H	1.201997	-1.75587	0.859546
43	H	6.590086	0.98994	-1.517331
44	H	6.949575	-0.321211	-0.358987
45	H	6.380662	-0.714987	-2.009931
46	H	4.195333	-5.185797	0.762703
47	H	-1.998498	0.346665	-2.388668
48	H	-0.781957	-0.521005	-1.486104
49	H	-3.453	0.266206	-0.225279
50	H	-1.673944	-2.042223	0.622564
51	H	-1.342342	-0.403933	1.134083
52	H	-2.751751	-0.936687	2.931231
53	H	-5.860694	-2.653683	0.760769
54	H	-5.677931	-1.024588	0.134566
55	H	-3.969795	-0.374132	-3.085314
56	H	-5.079785	-1.723441	-2.819416
57	H	-5.202957	-0.267491	-1.817879
58	H	-2.061716	-2.084204	-2.869926
59	H	-1.873103	-3.023334	-1.37329
60	H	-3.208281	-3.380185	-2.475451
61	H	-6.214848	-0.793809	2.890718
62	H	-5.78175	-2.464648	3.240944
63	H	-4.838147	-1.137286	3.950526
**2S, 2"S-1** Conf. 5		Standard Orientation (Ångstroms)		
I	atom	X	Y	Z
1	C	-2.082106	2.306244	-0.483234
2	C	-1.679359	3.643394	-0.396893
3	C	-0.328953	3.951816	-0.280816
4	C	0.64552	2.907928	-0.248538
5	C	0.196797	1.567183	-0.376922
6	C	-1.152307	1.234536	-0.504623
7	C	2.039609	3.202289	-0.007753
8	C	2.963067	2.005703	0.121578
9	C	2.453888	0.838454	-0.729058
10	O	1.07975	0.527515	-0.388493

11	O	2.481925	4.358625	0.121235
12	C	3.26244	-0.43204	-0.635757
13	O	-3.412628	2.081943	-0.564481
14	O	0.040865	5.236132	-0.165361
15	C	3.433577	-1.146259	0.575353
16	C	4.20172	-2.310378	0.612592
17	C	4.813671	-2.781084	-0.555324
18	C	4.659074	-2.097054	-1.760216
19	C	3.881256	-0.938459	-1.777699
20	O	2.812045	-0.632726	1.675271
21	C	2.955934	-1.305424	2.918057
22	O	5.550483	-3.925518	-0.438778
23	C	-1.567966	-0.185699	-0.819598
24	C	-1.761571	-1.231483	0.319312
25	C	-1.978756	-2.605515	-0.348488
26	C	-3.267233	-2.833205	-1.10555
27	C	-4.486943	-2.287094	-0.977282
28	C	-4.956923	-1.23613	0.012484
29	O	-4.020076	-0.353692	0.620668
30	C	-2.879632	-0.891327	1.349236
31	C	-2.467122	0.230469	2.314421
32	C	-3.296172	-2.099765	2.20585
33	C	-5.631695	-2.767487	-1.845715
34	H	-2.423058	4.431194	-0.386209
35	H	3.971009	2.297748	-0.188168
36	H	3.007526	1.707228	1.174867
37	H	2.453324	1.161701	-1.780488
38	H	-3.628306	1.169925	-0.257927
39	H	1.029047	5.237215	-0.046483
40	H	4.347142	-2.873637	1.525533
41	H	5.129445	-2.461735	-2.670362
42	H	3.75247	-0.407783	-2.717895
43	H	2.383376	-0.719965	3.639339
44	H	2.550353	-2.323951	2.87095
45	H	4.005922	-1.348201	3.234578
46	H	5.924653	-4.147658	-1.305567
47	H	-2.477953	-0.161248	-1.426515
48	H	-0.787058	-0.602828	-1.464234
49	H	-0.816444	-1.292508	0.875257
50	H	-1.148182	-2.77468	-1.046403
51	H	-1.875145	-3.404153	0.401225
52	H	-3.181738	-3.611386	-1.865682
53	H	-5.653467	-0.561523	-0.500062

54	H	-5.548695	-1.740268	0.794361
55	H	-1.607263	-0.089792	2.912434
56	H	-2.188466	1.147843	1.797429
57	H	-3.295988	0.456072	2.993208
58	H	-4.055978	-1.79558	2.933818
59	H	-3.695039	-2.931063	1.620539
60	H	-2.428475	-2.469393	2.762866
61	H	-5.330217	-3.603836	-2.48269
62	H	-6.484448	-3.100469	-1.236845
63	H	-6.006152	-1.96318	-2.49385
**2S, 2"S-1** Conf. 6		Standard Orientation (Ångstroms)		
I	atom	X	Y	Z
1	C	-1.948153	2.678462	-0.817285
2	C	-1.570268	3.919359	-0.303359
3	C	-0.278636	4.090105	0.183122
4	C	0.649874	3.006424	0.146669
5	C	0.225327	1.76884	-0.405234
6	C	-1.070603	1.571033	-0.888432
7	C	1.95867	3.136518	0.751465
8	C	2.811162	1.883897	0.781828
9	C	2.495212	0.986926	-0.417812
10	O	1.073281	0.705127	-0.474499
11	O	2.371551	4.195159	1.257554
12	C	3.235905	-0.325718	-0.459252
13	O	-3.227517	2.59336	-1.274816
14	O	0.070965	5.277111	0.69689
15	C	3.108641	-1.310092	0.551552
16	C	3.823566	-2.505467	0.47327
17	C	4.678347	-2.738502	-0.610777
18	C	4.819443	-1.785543	-1.618993
19	C	4.090306	-0.599588	-1.526301
20	O	2.262406	-1.017779	1.58022
21	C	2.093705	-1.973084	2.619532
22	O	5.345195	-3.929979	-0.615455
23	C	-1.511731	0.252948	-1.490577
24	C	-2.423757	-0.605034	-0.562809
25	C	-1.611306	-1.220341	0.6005
26	C	-2.419144	-1.50362	1.846308
27	C	-3.690663	-1.916595	1.906679
28	C	-4.513285	-2.25773	0.679109
29	O	-3.759334	-2.675216	-0.447981
30	C	-3.233872	-1.681747	-1.348619

31	C	-4.390697	-1.043793	-2.142071
32	C	-2.35727	-2.511753	-2.296307
33	C	-4.421736	-2.083747	3.215924
34	H	-2.279981	4.737353	-0.27564
35	H	3.867256	2.16966	0.782505
36	H	2.607875	1.343375	1.713006
37	H	2.741084	1.544614	-1.333425
38	H	-3.396485	1.699082	-1.607904
39	H	1.00934	5.183396	1.016904
40	H	3.742948	-3.273854	1.231387
41	H	5.479089	-1.965037	-2.464651
42	H	4.192001	0.141484	-2.315443
43	H	1.379825	-1.529757	3.315458
44	H	1.687522	-2.915775	2.23308
45	H	3.038713	-2.170023	3.14143
46	H	5.899728	-3.977182	-1.409795
47	H	-2.02698	0.448129	-2.443619
48	H	-0.621608	-0.323746	-1.753013
49	H	-3.158278	0.072381	-0.102989
50	H	-1.104039	-2.132171	0.25346
51	H	-0.806219	-0.52938	0.869324
52	H	-1.905817	-1.317523	2.790409
53	H	-5.162662	-3.110604	0.918095
54	H	-5.192011	-1.425027	0.429953
55	H	-4.982693	-1.825277	-2.629218
56	H	-5.061867	-0.466921	-1.494774
57	H	-4.022198	-0.372586	-2.927084
58	H	-1.972249	-1.908825	-3.124063
59	H	-1.510591	-2.956009	-1.764416
60	H	-2.955866	-3.3276	-2.7128
61	H	-5.336096	-1.473538	3.245802
62	H	-4.737994	-3.125406	3.368478
63	H	-3.798351	-1.788436	4.065435
**2S, 2"S-1** Conf. 7		Standard Orientation (Ångstroms)		
I	atom	X	Y	Z
1	C	2.63669	-2.38396	0.901671
2	C	2.540349	-3.630554	0.278163
3	C	1.374615	-3.971474	-0.396799
4	C	0.287463	-3.047878	-0.457277
5	C	0.438082	-1.788308	0.176236
6	C	1.597457	-1.425924	0.869651
7	C	-0.975226	-3.430304	-1.052954
8	C	-2.10566	-2.428488	-0.926738

9	C	-1.559708	-0.998666	-0.892107
10	O	-0.560738	-0.862671	0.151698
11	O	-1.160308	-4.527451	-1.608295
12	C	-2.591993	0.084033	-0.701717
13	O	3.816113	-2.143189	1.538246
14	O	1.284454	-5.177317	-0.974755
15	C	-3.41343	0.165539	0.449652
16	C	-4.363253	1.179487	0.575082
17	C	-4.511474	2.127596	-0.444461
18	C	-3.713453	2.06996	-1.586702
19	C	-2.764684	1.051788	-1.690579
20	O	-3.217574	-0.79019	1.401425
21	C	-3.999967	-0.74708	2.587094
22	O	-5.463069	3.086945	-0.250134
23	C	1.678613	-0.071971	1.548208
24	C	2.013303	1.114153	0.600401
25	C	3.475473	1.053304	0.100192
26	C	3.716659	1.720938	-1.235236
27	C	3.126048	2.828143	-1.699289
28	C	2.153682	3.656225	-0.882038
29	O	2.324886	3.560865	0.521703
30	C	1.665519	2.496437	1.237623
31	C	0.14631	2.740791	1.251571
32	C	2.225227	2.654536	2.658332
33	C	3.387749	3.355757	-3.088401
34	H	3.367925	-4.327688	0.329862
35	H	-2.793959	-2.557558	-1.767391
36	H	-2.658113	-2.638354	-0.004149
37	H	-1.042399	-0.809859	-1.844125
38	H	3.798502	-1.255145	1.926909
39	H	0.37198	-5.237472	-1.367694
40	H	-5.002393	1.263039	1.444719
41	H	-3.823965	2.806453	-2.379093
42	H	-2.137449	1.007397	-2.577707
43	H	-3.837786	0.187556	3.138226
44	H	-5.069423	-0.859037	2.368141
45	H	-3.663513	-1.589088	3.194033
46	H	-5.473159	3.68075	-1.016967
47	H	0.715178	0.115688	2.031277
48	H	2.407077	-0.096337	2.372449
49	H	1.367398	0.993534	-0.277523
50	H	4.150669	1.471114	0.862499

51	H	3.763799	0.00152	-0.010041
52	H	4.430673	1.217533	-1.887257
53	H	2.313585	4.717131	-1.118155
54	H	1.116272	3.434829	-1.183022
55	H	-0.063075	3.73452	1.661592
56	H	-0.288151	2.682247	0.248422
57	H	-0.373799	1.999325	1.865961
58	H	3.302118	2.459077	2.687024
59	H	2.067363	3.68515	2.990105
60	H	1.726827	1.985822	3.366569
61	H	2.455924	3.44636	-3.665594
62	H	3.832816	4.360364	-3.057968
63	H	4.062618	2.700692	-3.647678
**2S, 2"S-1** Conf. 8		Standard Orientation (Ångstroms)		
I	atom	X	Y	Z
1	C	-0.868749	2.613439	0.561263
2	C	0.101104	3.518529	1.006887
3	C	1.412372	3.396596	0.564604
4	C	1.769956	2.346617	-0.335231
5	C	0.755131	1.451733	-0.76709
6	C	-0.572375	1.572963	-0.357225
7	C	3.111978	2.236772	-0.848551
8	C	3.34378	1.167569	-1.901841
9	C	2.404876	-0.027693	-1.735852
10	O	1.028209	0.447817	-1.648579
11	O	4.047326	2.981446	-0.496599
12	C	2.725429	-0.964516	-0.585715
13	O	-2.114318	2.770897	1.062128
14	O	2.33515	4.267088	1.002959
15	C	2.141519	-2.255377	-0.573887
16	C	2.395579	-3.154618	0.461252
17	C	3.245862	-2.779283	1.508642
18	C	3.843715	-1.520109	1.515833
19	C	3.575593	-0.634351	0.470555
20	O	1.332194	-2.55038	-1.634053
21	C	0.814359	-3.867433	-1.750336
22	O	3.45025	-3.705445	2.491166
23	C	-1.654972	0.716576	-0.979812
24	C	-1.943396	-0.71993	-0.449601
25	C	-2.975631	-1.362416	-1.400811
26	C	-4.389195	-0.82626	-1.392187
27	C	-5.105884	-0.212425	-0.437261
28	C	-4.68988	0.138079	0.980177

29	O	-3.312061	0.287405	1.303367
30	C	-2.380476	-0.802029	1.042023
31	C	-1.211966	-0.564165	2.01072
32	C	-3.006508	-2.158782	1.409919
33	C	-6.552547	0.159098	-0.690922
34	H	-0.169015	4.292945	1.714728
35	H	3.147447	1.629017	-2.879487
36	H	4.394682	0.865999	-1.891294
37	H	2.389111	-0.611072	-2.658237
38	H	-2.601911	1.913644	1.040525
39	H	3.194622	4.01141	0.573152
40	H	1.960068	-4.145469	0.48498
41	H	4.511605	-1.228787	2.323096
42	H	4.060283	0.335917	0.489811
43	H	0.253869	-3.884653	-2.68657
44	H	1.618615	-4.612893	-1.790535
45	H	0.139176	-4.109474	-0.919482
46	H	4.04516	-3.328701	3.158327
47	H	-2.592101	1.28117	-0.976118
48	H	-1.388308	0.588146	-2.034149
49	H	-1.016323	-1.298472	-0.549769
50	H	-2.590887	-1.268231	-2.424976
51	H	-3.0206	-2.447322	-1.221454
52	H	-4.920333	-1.013485	-2.326841
53	H	-5.124657	1.110808	1.240369
54	H	-5.152715	-0.5905	1.666021
55	H	-0.733343	0.402676	1.860831
56	H	-1.573849	-0.612498	3.042983
57	H	-0.448847	-1.337552	1.87319
58	H	-3.260472	-2.175186	2.475239
59	H	-3.907273	-2.394057	0.838477
60	H	-2.278176	-2.957035	1.229606
61	H	-6.893083	-0.193405	-1.668625
62	H	-7.216956	-0.272517	0.071304
63	H	-6.697834	1.247431	-0.65153
**2S, 2"S-1** Conf. 9		Standard Orientation (Ångstroms)		
I	atom	X	Y	Z
1	C	2.316129	2.769013	0.522903
2	C	1.82798	4.029344	0.180814
3	C	0.513515	4.153483	-0.256922
4	C	-0.326386	3.00275	-0.330756
5	C	0.212403	1.74166	0.042641

6	C	1.539681	1.586469	0.44791
7	C	-1.671567	3.105931	-0.858401
8	C	-2.447771	1.808604	-0.963993
9	C	-1.988302	0.829636	0.120377
10	O	-0.564482	0.618267	0.000588
11	O	-2.174997	4.180371	-1.230814
12	C	-2.681577	-0.511872	0.061023
13	O	3.607281	2.733117	0.954714
14	O	0.056996	5.363087	-0.608158
15	C	-4.0102	-0.619562	0.528388
16	C	-4.695253	-1.832549	0.475789
17	C	-4.054673	-2.961371	-0.04888
18	C	-2.742092	-2.878237	-0.511384
19	C	-2.071806	-1.653663	-0.450852
20	O	-4.5545	0.534738	1.020994
21	C	-5.873452	0.49629	1.548077
22	O	-4.781097	-4.120182	-0.074342
23	C	2.133045	0.241946	0.817902
24	C	3.168453	-0.288284	-0.223453
25	C	2.440306	-0.812298	-1.475552
26	C	1.718724	-2.137137	-1.410048
27	C	1.873459	-3.207333	-0.614667
28	C	2.864611	-3.400255	0.517797
29	O	3.425448	-2.275422	1.163053
30	C	4.166328	-1.308659	0.396867
31	C	5.079265	-0.635638	1.437144
32	C	5.063684	-1.961532	-0.669292
33	C	1.04423	-4.453814	-0.844821
34	H	2.466826	4.901034	0.256354
35	H	-3.516219	2.016725	-0.874205
36	H	-2.269943	1.366623	-1.954409
37	H	-2.171882	1.290069	1.101056
38	H	3.829943	1.834804	1.243026
39	H	-0.881711	5.234735	-0.915321
40	H	-5.713603	-1.935595	0.828363
41	H	-2.241812	-3.75754	-0.911199
42	H	-1.043808	-1.591313	-0.789088
43	H	-6.603865	0.214594	0.77918
44	H	-5.946798	-0.201891	2.391367
45	H	-6.084742	1.509173	1.894822
46	H	-4.234598	-4.822059	-0.460514
47	H	2.591963	0.305236	1.813834
48	H	1.331227	-0.489499	0.92586

49	H	3.77317	0.562725	-0.569027
50	H	1.711188	-0.049349	-1.777294
51	H	3.151677	-0.85966	-2.31426
52	H	0.972313	-2.242957	-2.199887
53	H	2.361493	-3.943613	1.327775
54	H	3.659875	-4.076021	0.154237
55	H	5.644468	0.190858	0.990066
56	H	4.509116	-0.260991	2.292655
57	H	5.796071	-1.363743	1.829061
58	H	5.768366	-2.653855	-0.195791
59	H	4.49469	-2.512638	-1.421741
60	H	5.646154	-1.191946	-1.188026
61	H	0.376914	-4.650138	0.00591
62	H	0.432702	-4.370038	-1.748418
63	H	1.681228	-5.343078	-0.954321

**2S, 2"S-1** Conf. 10		Standard Orientation (Ångstroms)		
I	atom	X	Y	Z
1	C	-0.105108	-3.124795	-0.574478
2	C	0.998847	-3.571352	-1.301264
3	C	2.244329	-3.002602	-1.060882
4	C	2.385846	-1.978723	-0.076093
5	C	1.228976	-1.533835	0.618318
6	C	-0.033982	-2.087266	0.384937
7	C	3.689599	-1.46168	0.26395
8	C	3.746342	-0.505602	1.439962
9	C	2.469119	0.322177	1.582688
10	O	1.307509	-0.566224	1.571334
11	O	4.734328	-1.792896	-0.327089
12	C	2.300066	1.453336	0.587906
13	O	-1.283289	-3.749692	-0.849106
14	O	3.304779	-3.444779	-1.75059
15	C	1.389061	2.491977	0.889566
16	C	1.198132	3.559581	0.007821
17	C	1.918973	3.605709	-1.192379
18	C	2.831718	2.599609	-1.507321
19	C	3.007693	1.544375	-0.615431
20	O	0.733505	2.383201	2.081407
21	C	-0.129315	3.434602	2.489161
22	O	1.766115	4.625237	-2.086835
23	C	-1.26432	-1.623319	1.139049
24	C	-2.286133	-0.808524	0.289692
25	C	-1.704667	0.571474	-0.087031

26	C	-2.289317	1.194271	-1.333405
27	C	-3.548663	1.095368	-1.775013
28	C	-4.645812	0.387551	-1.004643
29	O	-4.46078	0.357377	0.40192
30	C	-3.679284	-0.702767	0.985194
31	C	-4.457278	-2.029141	0.885969
32	C	-3.579178	-0.276878	2.45648
33	C	-3.988882	1.689827	-3.090105
34	H	0.887291	-4.362148	-2.033137
35	H	3.868333	-1.116157	2.34539
36	H	4.632617	0.128388	1.353719
37	H	2.419842	0.743016	2.588118
38	H	-1.980043	-3.397736	-0.274818
39	H	4.094228	-2.938974	-1.417471
40	H	0.497272	4.354671	0.240879
41	H	3.38913	2.655038	-2.435717
42	H	3.733618	0.779758	-0.871125
43	H	-0.501684	3.148126	3.474164
44	H	0.408237	4.388636	2.566422
45	H	-0.977091	3.548088	1.801666
46	H	1.109342	5.253176	-1.747957
47	H	-1.764559	-2.497013	1.585032
48	H	-0.937463	-1.012597	1.983112
49	H	-2.441986	-1.344493	-0.657718
50	H	-1.789837	1.255692	0.769585
51	H	-0.629599	0.451852	-0.253608
52	H	-1.5826	1.756366	-1.944505
53	H	-5.587952	0.931601	-1.15543
54	H	-4.814187	-0.621387	-1.416386
55	H	-5.450794	-1.911099	1.330565
56	H	-4.589624	-2.349424	-0.154157
57	H	-3.94762	-2.837621	1.423065
58	H	-3.119013	-1.053953	3.073619
59	H	-2.996611	0.643316	2.562057
60	H	-4.585483	-0.082198	2.839553
61	H	-4.418445	0.924128	-3.752237
62	H	-4.770307	2.449577	-2.945919
63	H	-3.15292	2.155347	-3.620982
**2S, 2"S-1** Conf. 11		Standard Orientation (Ångstroms)		
I	atom	X	Y	Z
1	C	0.297022	-3.008426	0.638829
2	C	-0.826221	-3.425403	1.352227
3	C	-2.072711	-2.905943	1.019414

4	C	-2.195687	-1.961651	-0.041388
5	C	-1.024351	-1.550703	-0.72591
6	C	0.240414	-2.055834	-0.405759
7	C	-3.49175	-1.452374	-0.426849
8	C	-3.538959	-0.540357	-1.636015
9	C	-2.233122	0.217054	-1.910626
10	O	-1.083508	-0.672025	-1.766081
11	O	-4.548355	-1.784941	0.140311
12	C	-1.987666	1.537388	-1.196378
13	O	1.478062	-3.575282	1.013293
14	O	-3.150952	-3.31496	1.704937
15	C	-2.394957	1.847218	0.123794
16	C	-2.082319	3.078802	0.703039
17	C	-1.352788	4.026172	-0.022263
18	C	-0.942222	3.751696	-1.328063
19	C	-1.268292	2.517369	-1.886266
20	O	-3.10919	0.892188	0.77348
21	C	-3.585453	1.125125	2.092214
22	O	-1.083	5.2059	0.608312
23	C	1.493505	-1.632601	-1.146201
24	C	2.441838	-0.696897	-0.335529
25	C	1.83444	0.716727	-0.206612
26	C	2.303447	1.506301	0.993231
27	C	3.514438	1.471946	1.561642
28	C	4.671692	0.665942	1.003741
29	O	4.626121	0.451565	-0.398435
30	C	3.89126	-0.675168	-0.912538
31	C	4.646477	-1.975877	-0.57708
32	C	3.925062	-0.438823	-2.428596
33	C	3.836374	2.245501	2.816552
34	H	-0.728639	-4.153457	2.148462
35	H	-3.751635	-1.18381	-2.501461
36	H	-4.381715	0.146226	-1.528285
37	H	-2.200361	0.446153	-2.978769
38	H	2.191867	-3.248346	0.445284
39	H	-3.935154	-2.858559	1.293455
40	H	-2.391351	3.329048	1.7099
41	H	-0.381505	4.487325	-1.899872
42	H	-0.946032	2.303501	-2.902703
43	H	-4.135543	0.222342	2.360428
44	H	-2.756194	1.276965	2.79376
45	H	-4.258476	1.990797	2.125556

46	H	-0.566945	5.770963	0.012313
47	H	2.04097	-2.532284	-1.467274
48	H	1.199015	-1.129669	-2.069932
49	H	2.51146	-1.098012	0.686048
50	H	2.005996	1.283236	-1.133339
51	H	0.747357	0.615089	-0.12947
52	H	1.549184	2.149076	1.447811
53	H	5.602431	1.223213	1.175157
54	H	4.783225	-0.280851	1.557824
55	H	5.678699	-1.909056	-0.935505
56	H	4.676175	-2.164447	0.502418
57	H	4.186568	-2.847275	-1.05809
58	H	4.963922	-0.306218	-2.745873
59	H	3.500924	-1.282898	-2.980279
60	H	3.375838	0.467886	-2.699758
61	H	4.635883	2.979438	2.641457
62	H	2.959745	2.779573	3.195671
63	H	4.194022	1.581394	3.61687

Table S12. The coordinate for the low-energy conformer 2S, 2"R-1 in NMR and ECD calculations

**2S, 2"R-1** Conf. 1		Standard Orientation (Ångstroms)		
I	atom	X	Y	Z
1	C	-2.1988	2.863775	-0.87913
2	C	-1.78591	4.073043	-0.31364
3	C	-0.55325	4.144679	0.328831
4	C	0.275686	2.988547	0.400851
5	C	-0.18419	1.785055	-0.19872
6	C	-1.41943	1.687312	-0.84363
7	C	1.520017	3.023003	1.140194
8	C	2.270203	1.710466	1.240829
9	C	2.002099	0.832371	0.014582
10	O	0.576429	0.65338	-0.16262
11	O	1.953136	4.048722	1.693915
12	C	2.636756	-0.53411	0.114246
13	O	-3.3995	2.779609	-1.51965
14	O	-0.17455	5.304449	0.881489
15	C	3.95764	-0.72951	-0.3433
16	C	4.572958	-1.97747	-0.24349
17	C	3.873851	-3.04727	0.327248
18	C	2.572084	-2.87251	0.798175
19	C	1.972989	-1.61649	0.687246
20	O	4.569767	0.374135	-0.86849
21	C	5.885431	0.241082	-1.38853

22	O	4.533826	-4.24229	0.394339
23	C	-1.86495	0.412515	-1.52882
24	C	-2.38126	-0.70159	-0.57559
25	C	-3.67646	-0.27918	0.156441
26	C	-3.89903	-0.96051	1.487478
27	C	-3.58092	-2.21867	1.813605
28	C	-3.00634	-3.21185	0.822091
29	O	-3.35485	-2.97137	-0.52959
30	C	-2.54529	-2.06833	-1.31313
31	C	-3.34546	-1.95019	-2.61815
32	C	-1.18415	-2.72389	-1.60627
33	C	-3.77966	-2.76364	3.206553
34	H	-2.41029	4.961032	-0.3591
35	H	1.938967	1.186335	2.148333
36	H	3.340151	1.910415	1.344983
37	H	2.381204	1.348401	-0.87583
38	H	-3.82667	3.650563	-1.50109
39	H	0.709905	5.138474	1.310146
40	H	5.583182	-2.15187	-0.59161
41	H	2.030149	-3.70389	1.24341
42	H	0.957148	-1.47601	1.041296
43	H	6.156364	1.227774	-1.76781
44	H	6.596296	-0.05673	-0.60752
45	H	5.919951	-0.48788	-2.20787
46	H	3.950519	-4.89974	0.804313
47	H	-2.64675	0.663174	-2.24999
48	H	-1.01325	0.022396	-2.09644
49	H	-1.60952	-0.8406	0.19247
50	H	-4.54576	-0.42589	-0.50031
51	H	-3.63374	0.799413	0.34145
52	H	-4.34581	-0.33752	2.26328
53	H	-3.41164	-4.20747	1.049702
54	H	-1.91415	-3.29615	0.953532
55	H	-4.30712	-1.45438	-2.45358
56	H	-2.79223	-1.39582	-3.38173
57	H	-3.54923	-2.95476	-3.00157
58	H	-0.58276	-2.11445	-2.28805
59	H	-0.59149	-2.86564	-0.69661
60	H	-1.34084	-3.70302	-2.07123
61	H	-2.83792	-3.14557	3.627607
62	H	-4.16099	-1.99709	3.888237
63	H	-4.48552	-3.60643	3.210581

**2S, 2"R-1** Conf. 2		Standard Orientation (Ångstroms)		
I	atom	X	Y	Z
1	C	2.15396	2.588533	-0.741556
2	C	1.735492	3.828178	-0.255019
3	C	0.397033	4.013021	0.083641
4	C	-0.526211	2.938876	-0.063615
5	C	-0.045644	1.689928	-0.54472
6	C	1.290624	1.481275	-0.889455
7	C	-1.938932	3.148102	0.178843
8	C	-2.848538	1.975925	-0.128965
9	C	-2.109618	0.649951	0.076745
10	O	-0.897097	0.636584	-0.707189
11	O	-2.404785	4.227291	0.585011
12	C	-2.928766	-0.559492	-0.309912
13	O	3.453795	2.384789	-1.098901
14	O	0.000273	5.211973	0.529433
15	C	-3.878898	-1.069997	0.59523
16	C	-4.662869	-2.177897	0.260825
17	C	-4.504452	-2.783346	-0.992381
18	C	-3.572697	-2.288391	-1.904645
19	C	-2.800437	-1.183748	-1.551558
20	O	-3.973392	-0.407809	1.789428
21	C	-4.870514	-0.897205	2.774773
22	O	-5.246245	-3.867823	-1.369559
23	C	1.809432	0.159575	-1.414532
24	C	2.644067	-0.655077	-0.384668
25	C	1.745544	-1.22797	0.735549
26	C	2.453524	-1.478377	2.047255
27	C	3.711021	-1.903496	2.216041
28	C	4.61816	-2.29357	1.065092
29	O	3.946132	-2.735239	-0.101761
30	C	3.505776	-1.760281	-1.070985
31	C	2.698738	-2.612053	-2.060222
32	C	4.727275	-1.155223	-1.78552
33	C	4.338045	-2.037859	3.581903
34	H	2.427868	4.658466	-0.147232
35	H	-3.179195	2.052998	-1.174252
36	H	-3.735743	2.02631	0.507251
37	H	-1.820032	0.573325	1.133133
38	H	3.952558	3.204439	-0.954235
39	H	-0.983635	5.147484	0.676896
40	H	-5.394223	-2.573897	0.95837
41	H	-3.462384	-2.772967	-2.868605

42	H	-2.064919	-0.800513	-2.250402
43	H	-4.756847	-0.236887	3.636126
44	H	-5.910403	-0.860273	2.425048
45	H	-4.62169	-1.925019	3.068843
46	H	-5.844748	-4.112165	-0.646951
47	H	0.961421	-0.442396	-1.749964
48	H	2.425579	0.364352	-2.296741
49	H	3.341668	0.047416	0.087684
50	H	1.246522	-2.143385	0.386114
51	H	0.937528	-0.513096	0.925939
52	H	1.871652	-1.260006	2.94361
53	H	5.234625	-3.14689	1.379872
54	H	5.326569	-1.477772	0.844397
55	H	1.801705	-3.025707	-1.588979
56	H	2.396853	-2.031949	-2.937185
57	H	3.316529	-3.450406	-2.39662
58	H	4.425831	-0.472258	-2.585921
59	H	5.358499	-0.583951	-1.096556
60	H	5.3326	-1.954893	-2.225287
61	H	5.256763	-1.438543	3.662737
62	H	3.656222	-1.708292	4.371989
63	H	4.626691	-3.077938	3.790368
**2S, 2"R-1** Conf. 3		Standard Orientation (Ångstroms)		
I	atom	X	Y	Z
1	C	2.16979	2.361577	0.456564
2	C	1.749185	3.675958	0.235072
3	C	0.42514	3.927278	-0.104559
4	C	-0.499484	2.84657	-0.224551
5	C	-0.03324	1.531699	0.032664
6	C	1.289479	1.253294	0.383555
7	C	-1.854282	3.077554	-0.676965
8	C	-2.713811	1.841932	-0.865208
9	C	-2.287207	0.73225	0.10066
10	O	-0.876867	0.460697	-0.056385
11	O	-2.304239	4.208615	-0.93144
12	C	-3.038577	-0.562552	-0.105029
13	O	3.480535	2.214811	0.776891
14	O	0.03988	5.189723	-0.339557
15	C	-4.319308	-0.721851	0.467311
16	C	-5.046861	-1.897232	0.282762
17	C	-4.501514	-2.931142	-0.487368
18	C	-3.241574	-2.791631	-1.069368

19	C	-2.528441	-1.6071	-0.871711
20	O	-4.776214	0.344553	1.190353
21	C	-6.04314	0.244327	1.826191
22	O	-5.265873	-4.055971	-0.629105
23	C	1.712973	-0.152455	0.753445
24	C	2.091162	-1.090068	-0.427911
25	C	3.138492	-0.452547	-1.362006
26	C	4.541264	-0.233428	-0.839921
27	C	5.271469	-0.94044	0.040868
28	C	4.850259	-2.195608	0.786874
29	O	3.49048	-2.378567	1.121102
30	C	2.491577	-2.507071	0.086487
31	C	1.318314	-3.190335	0.808588
32	C	2.960987	-3.426246	-1.054154
33	C	6.713626	-0.569198	0.311925
34	H	2.459375	4.49076	0.308867
35	H	-2.602214	1.490283	-1.900497
36	H	-3.763966	2.103369	-0.712326
37	H	-2.442966	1.086252	1.128046
38	H	3.74379	1.277332	0.733935
39	H	-0.919188	5.150361	-0.602392
40	H	-6.029039	-2.041184	0.714745
41	H	-2.819158	-3.595125	-1.668804
42	H	-1.544797	-1.494597	-1.31466
43	H	-6.063136	-0.580142	2.549909
44	H	-6.189273	1.191037	2.348784
45	H	-6.848019	0.104098	1.093729
46	H	-4.786168	-4.692938	-1.181045
47	H	2.548341	-0.124808	1.461205
48	H	0.882423	-0.608484	1.297124
49	H	1.185541	-1.21992	-1.0364
50	H	3.206285	-1.04156	-2.288294
51	H	2.745635	0.518853	-1.685559
52	H	5.053271	0.604996	-1.314814
53	H	5.361426	-2.207005	1.757358
54	H	5.243552	-3.065922	0.232289
55	H	1.072304	-2.671321	1.7378
56	H	0.423856	-3.213067	0.176795
57	H	1.589977	-4.218732	1.067481
58	H	2.154995	-3.554369	-1.785469
59	H	3.833017	-3.039679	-1.58768
60	H	3.216343	-4.415062	-0.657699
61	H	7.041101	0.269726	-0.308576

62	H	6.865037	-0.297036	1.365242
63	H	7.382369	-1.418222	0.110759
**2S, 2"R-1** Conf. 4		Standard Orientation (Ångstroms)		
I	atom	X	Y	Z
1	C	-2.337085	1.571429	-1.069448
2	C	-2.165508	2.928216	-0.778125
3	C	-0.966141	3.375165	-0.238304
4	C	0.094982	2.452252	0.005361
5	C	-0.110317	1.088539	-0.334347
6	C	-1.309917	0.611526	-0.863892
7	C	1.31883	2.884361	0.642147
8	C	2.35092	1.805101	0.909438
9	C	2.238676	0.689735	-0.134015
10	O	0.890468	0.17175	-0.144797
11	O	1.533008	4.062542	0.981306
12	C	3.187382	-0.461913	0.10589
13	O	-3.526821	1.226201	-1.603807
14	O	-0.82997	4.673745	0.067707
15	C	4.537441	-0.339092	-0.290273
16	C	5.447176	-1.373234	-0.073707
17	C	5.015977	-2.549729	0.550587
18	C	3.688547	-2.691067	0.953662
19	C	2.792467	-1.643673	0.726947
20	O	4.868654	0.847405	-0.883298
21	C	6.199302	1.031491	-1.346338
22	O	5.957115	-3.525034	0.733707
23	C	-1.429208	-0.82224	-1.344109
24	C	-2.012255	-1.924698	-0.382765
25	C	-1.826991	-1.571027	1.107035
26	C	-2.771608	-0.608406	1.789041
27	C	-4.046692	-0.267797	1.550174
28	C	-4.936746	-0.744563	0.422642
29	O	-4.324383	-1.217153	-0.776527
30	C	-3.454621	-2.379939	-0.738124
31	C	-3.541195	-2.958161	-2.159332
32	C	-3.977855	-3.444531	0.240548
33	C	-4.775021	0.681795	2.477153
34	H	-2.978	3.622796	-0.955223
35	H	2.179555	1.388728	1.912041
36	H	3.349574	2.248625	0.897017
37	H	2.434599	1.121809	-1.124319
38	H	-3.70009	0.261853	-1.483949

39	H	0.074261	4.77673	0.471312
40	H	6.485315	-1.299521	-0.371774
41	H	3.353412	-3.606695	1.436142
42	H	1.756389	-1.753574	1.027592
43	H	6.465373	0.285373	-2.1056
44	H	6.224027	2.027721	-1.790951
45	H	6.920819	0.98289	-0.521046
46	H	5.541666	-4.280264	1.178098
47	H	-1.991519	-0.818686	-2.283168
48	H	-0.417753	-1.141971	-1.606525
49	H	-1.390391	-2.815454	-0.550077
50	H	-1.833012	-2.504163	1.692104
51	H	-0.811213	-1.176776	1.22587
52	H	-2.32712	-0.153569	2.67566
53	H	-5.551779	0.095976	0.08228
54	H	-5.636771	-1.504195	0.803571
55	H	-3.284683	-2.217918	-2.921385
56	H	-2.864527	-3.812183	-2.270562
57	H	-4.562853	-3.296049	-2.358915
58	H	-4.987914	-3.758426	-0.043932
59	H	-3.330299	-4.326892	0.198809
60	H	-4.002986	-3.097656	1.276076
61	H	-4.168653	0.934792	3.351422
62	H	-5.034284	1.616061	1.960817
63	H	-5.718945	0.247768	2.837199
**2S, 2"R-1** Conf. 5		Standard Orientation (Ångstroms)		
I	atom	X	Y	Z
1	C	-1.832907	2.623871	0.822466
2	C	-1.463342	3.86144	0.290991
3	C	-0.210572	4.004191	-0.300049
4	C	0.676104	2.890897	-0.36091
5	C	0.243902	1.645875	0.174326
6	C	-1.00854	1.480408	0.771007
7	C	2.024121	3.055556	-0.863373
8	C	2.932699	1.84865	-0.754447
9	C	2.122187	0.552562	-0.844264
10	O	1.055424	0.554261	0.138582
11	O	2.447605	4.128366	-1.330341
12	C	2.914744	-0.719266	-0.677113
13	O	-3.045317	2.462191	1.42621
14	O	0.144473	5.200074	-0.78825
15	C	3.635245	-1.023509	0.503805
16	C	4.359011	-2.2115	0.609045

17	C	4.378467	-3.114195	-0.460881
18	C	3.676288	-2.837773	-1.633475
19	C	2.952755	-1.647685	-1.716357
20	O	3.574956	-0.100008	1.504492
21	C	4.243503	-0.37135	2.728566
22	O	5.110687	-4.253138	-0.283895
23	C	-1.465958	0.162998	1.359526
24	C	-2.530343	-0.585138	0.506856
25	C	-1.919777	-1.128935	-0.805066
26	C	-2.905488	-1.308329	-1.937243
27	C	-4.185624	-1.685888	-1.841088
28	C	-4.834962	-2.089982	-0.531581
29	O	-3.942426	-2.610394	0.438352
30	C	-3.258309	-1.697687	1.323271
31	C	-2.287413	-2.622663	2.069933
32	C	-4.265903	-1.094869	2.318717
33	C	-5.098617	-1.748058	-3.040845
34	H	-2.126131	4.720881	0.340742
35	H	3.458698	1.892234	0.205747
36	H	3.682047	1.891686	-1.5505
37	H	1.63882	0.52182	-1.83177
38	H	-3.523981	3.305765	1.400122
39	H	1.077407	5.100786	-1.126233
40	H	4.916361	-2.467019	1.501095
41	H	3.684756	-3.539133	-2.464402
42	H	2.395826	-1.435262	-2.625642
43	H	4.037248	0.483963	3.373969
44	H	3.858335	-1.284431	3.198989
45	H	5.327295	-0.465975	2.583667
46	H	5.043447	-4.797651	-1.083708
47	H	-0.59267	-0.479029	1.497826
48	H	-1.87977	0.361895	2.354132
49	H	-3.291039	0.153868	0.226848
50	H	-1.382306	-2.068275	-0.609757
51	H	-1.153162	-0.423241	-1.143952
52	H	-2.524068	-1.07503	-2.932216
53	H	-5.54761	-2.90236	-0.729978
54	H	-5.434572	-1.256347	-0.129634
55	H	-1.775677	-2.099465	2.883084
56	H	-2.849672	-3.458625	2.497414
57	H	-1.534031	-3.038333	1.393535
58	H	-3.767406	-0.467334	3.064302

59	H	-5.008848	-0.467473	1.814796
60	H	-4.792644	-1.898902	2.843615
61	H	-4.592657	-1.409606	-3.950363
62	H	-5.465652	-2.769864	-3.213408
63	H	-5.989189	-1.118097	-2.900427
**2S, 2"R-1** Conf. 6		Standard Orientation (Ångstroms)		
I	atom	X	Y	Z
1	C	1.832971	2.623915	-0.82221
2	C	1.463389	3.861489	-0.290741
3	C	0.210547	4.004263	0.300126
4	C	-0.676189	2.891016	0.360841
5	C	-0.243985	1.645991	-0.174415
6	C	1.008538	1.480494	-0.770926
7	C	-2.024248	3.055721	0.863135
8	C	-2.932823	1.848795	0.754298
9	C	-2.122306	0.552731	0.844094
10	O	-1.05566	0.554485	-0.138975
11	O	-2.447788	4.128594	1.329911
12	C	-2.914751	-0.71917	0.677086
13	O	3.045451	2.462232	-1.425807
14	O	-0.144518	5.200129	0.788345
15	C	-3.635195	-1.023626	-0.503787
16	C	-4.358809	-2.21173	-0.608935
17	C	-4.378164	-3.114303	0.461092
18	C	-3.676042	-2.83766	1.633674
19	C	-2.952637	-1.647493	1.716433
20	O	-3.574937	-0.100284	-1.504643
21	C	-4.243269	-0.371982	-2.728762
22	O	-5.110209	-4.253383	0.284228
23	C	1.465931	0.163091	-1.359494
24	C	2.530219	-0.585161	-0.506827
25	C	1.919602	-1.129088	0.805028
26	C	2.905318	-1.308479	1.937227
27	C	4.185472	-1.685977	1.841087
28	C	4.834899	-2.089839	0.531548
29	O	3.942459	-2.610326	-0.438427
30	C	3.258247	-1.697636	-1.323316
31	C	2.287455	-2.622715	-2.069969
32	C	4.265778	-1.094679	-2.31873
33	C	5.09843	-1.748215	3.040868
34	H	2.126216	4.720909	-0.340375
35	H	-3.458897	1.892337	-0.205857
36	H	-3.682133	1.891852	1.55039

37	H	-1.638747	0.522063	1.831502
38	H	3.524146	3.305785	-1.399628
39	H	-1.077512	5.100824	1.126169
40	H	-4.916061	-2.467426	-1.500991
41	H	-3.684465	-3.538936	2.464673
42	H	-2.395721	-1.434915	2.625687
43	H	-4.036902	0.483137	-3.374387
44	H	-3.858014	-1.285191	-3.198859
45	H	-5.32708	-0.466593	-2.583979
46	H	-5.042839	-4.797818	1.084083
47	H	0.592592	-0.478844	-1.497912
48	H	1.879822	0.362035	-2.354062
49	H	3.290907	0.153802	-0.226703
50	H	1.382243	-2.068455	0.609612
51	H	1.152941	-0.42346	1.143918
52	H	2.523845	-1.075224	2.932194
53	H	5.547722	-2.90208	0.729854
54	H	5.434346	-1.256028	0.129692
55	H	1.775508	-2.099579	-2.883019
56	H	2.84982	-3.458544	-2.497575
57	H	1.534251	-3.038567	-1.393487
58	H	3.767204	-0.467275	-3.06438
59	H	5.008609	-0.46711	-1.814847
60	H	4.792646	-1.898662	-2.843584
61	H	4.592388	-1.409932	3.950404
62	H	5.465516	-2.770025	3.213292
63	H	5.988967	-1.118184	2.900576
**2S, 2"R-1** Conf. 7		Standard Orientation (Ångstroms)		
I	atom	X	Y	Z
1	C	-3.635294	0.556573	-0.822209
2	C	-4.189908	1.70813	-0.254644
3	C	-3.350915	2.6907	0.261859
4	C	-1.937476	2.520276	0.20021
5	C	-1.422795	1.343534	-0.407042
6	C	-2.246103	0.33669	-0.918935
7	C	-1.05431	3.473868	0.836537
8	C	0.416857	3.114557	0.846274
9	C	0.784013	2.293541	-0.393601
10	O	-0.083092	1.137364	-0.51733
11	O	-1.459283	4.514015	1.385862
12	C	2.214861	1.819283	-0.441786
13	O	-4.444405	-0.416772	-1.330277

14	O	-3.892178	3.779141	0.825613
15	C	2.765491	0.950613	0.532469
16	C	4.101395	0.555333	0.459306
17	C	4.910997	1.020605	-0.584179
18	C	4.391476	1.874712	-1.55623
19	C	3.051065	2.252793	-1.469348
20	O	1.922313	0.53797	1.521783
21	C	2.423165	-0.325695	2.533549
22	O	6.20751	0.592922	-0.585883
23	C	-1.666178	-0.892344	-1.585738
24	C	-1.047675	-1.928579	-0.604496
25	C	-2.137059	-2.603773	0.26003
26	C	-1.65521	-3.133925	1.591068
27	C	-0.461621	-3.675566	1.860946
28	C	0.595143	-3.922758	0.801564
29	O	0.090069	-4.120934	-0.505879
30	C	-0.154079	-2.971311	-1.346355
31	C	-0.848518	-3.593196	-2.565663
32	C	1.189138	-2.355686	-1.776342
33	C	-0.068062	-4.101467	3.254181
34	H	-5.265735	1.848637	-0.197394
35	H	0.627552	2.533484	1.751044
36	H	1.011166	4.032039	0.888973
37	H	0.602109	2.917215	-1.281051
38	H	-5.368942	-0.142313	-1.224511
39	H	-3.132657	4.332563	1.157655
40	H	4.545007	-0.107084	1.191562
41	H	5.015981	2.234275	-2.37057
42	H	2.642224	2.912465	-2.230881
43	H	1.579115	-0.524717	3.195528
44	H	2.782669	-1.271313	2.109056
45	H	3.233472	0.14871	3.101522
46	H	6.664095	0.97447	-1.351828
47	H	-2.45091	-1.372581	-2.175713
48	H	-0.891615	-0.562249	-2.286637
49	H	-0.391866	-1.369344	0.074228
50	H	-2.632589	-3.401175	-0.312985
51	H	-2.921321	-1.866332	0.463703
52	H	-2.361281	-3.036653	2.416811
53	H	1.127399	-4.852043	1.048042
54	H	1.356635	-3.124821	0.827146
55	H	-0.983615	-2.863045	-3.369004
56	H	-0.232236	-4.413895	-2.945581

57	H	-1.826933	-4.005639	-2.299946
58	H	1.044851	-1.535383	-2.486282
59	H	1.742288	-1.945648	-0.924994
60	H	1.809573	-3.120068	-2.256377
61	H	-0.849254	-3.866232	3.983653
62	H	0.130765	-5.181591	3.302432
63	H	0.856196	-3.602844	3.582726
**2S, 2"R-1** Conf. 8		Standard Orientation (Ångstroms)		
I	atom	X	Y	Z
1	C	-2.484307	1.447094	-1.107601
2	C	-2.478347	2.810184	-0.793016
3	C	-1.306885	3.415576	-0.357093
4	C	-0.10772	2.649447	-0.24033
5	C	-0.149019	1.277568	-0.606869
6	C	-1.315755	0.643838	-1.039192
7	C	1.09253	3.236181	0.31006
8	C	2.276296	2.304487	0.484533
9	C	2.250977	1.204334	-0.57951
10	O	0.979819	0.508962	-0.551054
11	O	1.173051	4.42899	0.658084
12	C	3.353314	0.178533	-0.480849
13	O	-3.663434	0.939644	-1.526262
14	O	-1.32573	4.715282	-0.024947
15	C	3.534901	-0.651738	0.652038
16	C	4.580837	-1.573624	0.699477
17	C	5.465048	-1.68323	-0.38046
18	C	5.306233	-0.879552	-1.508494
19	C	4.250145	0.032104	-1.538318
20	O	2.639346	-0.495559	1.670299
21	C	2.791854	-1.284764	2.842153
22	O	6.464143	-2.606558	-0.258734
23	C	-1.269189	-0.778247	-1.566078
24	C	-1.547788	-1.988025	-0.600745
25	C	-1.141458	-1.687139	0.856007
26	C	-2.053465	-0.872785	1.744239
27	C	-3.379377	-0.670328	1.73856
28	C	-4.396127	-1.198851	0.749257
29	O	-3.960492	-1.5584	-0.560561
30	C	-2.967987	-2.603106	-0.738396
31	C	-3.232348	-3.127274	-2.158873
32	C	-3.19191	-3.763805	0.245341
33	C	-4.035073	0.151598	2.827982

34	H	-3.395491	3.382441	-0.866207
35	H	2.229968	1.857019	1.483515
36	H	3.20102	2.885571	0.4184
37	H	2.325629	1.685515	-1.565715
38	H	-3.692599	-0.035242	-1.372049
39	H	-0.410103	4.938873	0.295343
40	H	4.738877	-2.218825	1.554052
41	H	5.9881	-0.963689	-2.351401
42	H	4.120882	0.655023	-2.419993
43	H	3.756552	-1.0971	3.330325
44	H	1.98256	-0.983251	3.509066
45	H	2.699988	-2.355103	2.619122
46	H	7.007128	-2.590057	-1.062254
47	H	-1.933684	-0.841076	-2.43365
48	H	-0.258396	-0.9246	-1.955426
49	H	-0.867862	-2.784796	-0.934396
50	H	-0.953188	-2.643072	1.370338
51	H	-0.16526	-1.189822	0.831436
52	H	-1.516333	-0.405704	2.571503
53	H	-5.143577	-0.418968	0.564331
54	H	-4.938859	-2.044091	1.200922
55	H	-3.206427	-2.328022	-2.904115
56	H	-2.486643	-3.879457	-2.437588
57	H	-4.224322	-3.587371	-2.204597
58	H	-4.194071	-4.185628	0.112768
59	H	-2.465501	-4.558421	0.043538
60	H	-3.078581	-3.46599	1.290152
61	H	-4.475522	1.071007	2.418626
62	H	-4.852614	-0.400771	3.313526
63	H	-3.317578	0.435843	3.602888
**2S, 2"R-1** Conf. 9		Standard Orientation (Ångstroms)		
I	atom	X	Y	Z
1	C	2.919244	1.865633	1.097747
2	C	3.066776	3.168238	0.613909
3	C	2.033765	3.753707	-0.108482
4	C	0.830633	3.02497	-0.350094
5	C	0.720875	1.712284	0.17421
6	C	1.744663	1.102125	0.904372
7	C	-0.216199	3.575097	-1.1835
8	C	-1.399985	2.664187	-1.4638
9	C	-1.63022	1.724743	-0.283911
10	O	-0.40566	0.973827	-0.02897
11	O	-0.16361	4.711868	-1.683372

12	C	-2.751692	0.727379	-0.429097
13	O	3.985468	1.377777	1.789534
14	O	2.192352	4.9964	-0.585253
15	C	-3.301617	0.134057	0.733617
16	C	-4.342805	-0.789237	0.645509
17	C	-4.847443	-1.143747	-0.611152
18	C	-4.318578	-0.579186	-1.771992
19	C	-3.278876	0.345203	-1.661249
20	O	-2.749479	0.526421	1.917361
21	C	-3.314588	0.042684	3.128703
22	O	-5.865174	-2.053197	-0.6253
23	C	1.531963	-0.283342	1.480734
24	C	1.725965	-1.448226	0.469636
25	C	3.214045	-1.63048	0.090886
26	C	3.457084	-2.257813	-1.26342
27	C	2.718164	-3.201072	-1.858383
28	C	1.53045	-3.868522	-1.193116
29	O	1.58552	-3.900699	0.223184
30	C	1.076134	-2.776574	0.969451
31	C	1.471295	-3.128221	2.410911
32	C	-0.457431	-2.726974	0.852058
33	C	3.013499	-3.690052	-3.254744
34	H	3.986589	3.711765	0.793112
35	H	-1.169037	2.091562	-2.372484
36	H	-2.281348	3.278656	-1.668684
37	H	-1.80963	2.327676	0.615042
38	H	3.807671	0.461873	2.053593
39	H	1.366915	5.213811	-1.095773
40	H	-4.779407	-1.252146	1.52127
41	H	-4.711297	-0.852434	-2.748593
42	H	-2.880304	0.781995	-2.571277
43	H	-3.214492	-1.046855	3.210361
44	H	-2.748175	0.519162	3.930482
45	H	-4.373201	0.317801	3.214737
46	H	-6.125926	-2.220366	-1.544468
47	H	2.183368	-0.445661	2.352322
48	H	0.511395	-0.316021	1.873868
49	H	1.193314	-1.152911	-0.442244
50	H	3.691959	-0.643446	0.075764
51	H	3.73334	-2.202569	0.875297
52	H	4.314772	-1.867025	-1.811139
53	H	1.502093	-4.922191	-1.502325

54	H	0.589144	-3.423282	-1.555114
55	H	2.559	-3.141501	2.534827
56	H	1.043625	-2.424445	3.131341
57	H	1.09946	-4.129857	2.646819
58	H	-0.781542	-2.534837	-0.175687
59	H	-0.883338	-3.682853	1.175326
60	H	-0.882584	-1.931505	1.47183
61	H	3.854249	-3.150617	-3.701322
62	H	3.252652	-4.762871	-3.26064
63	H	2.144661	-3.561228	-3.916525
**2S, 2"R-1** Conf. 10		Standard Orientation (Ångstroms)		
I	atom	X	Y	Z
1	C	-1.687147	3.085283	-0.922449
2	C	-1.117977	4.244965	-0.388454
3	C	0.115556	4.172444	0.253422
4	C	0.790949	2.922405	0.350505
5	C	0.178289	1.776437	-0.222431
6	C	-1.064786	1.820981	-0.857953
7	C	2.028135	2.807801	1.095332
8	C	2.598102	1.40941	1.226659
9	C	2.222628	0.555627	0.011132
10	O	0.788561	0.559434	-0.167952
11	O	2.589338	3.780102	1.629864
12	C	2.674691	-0.88176	0.120632
13	O	-2.892309	3.138957	-1.558341
14	O	0.640622	5.285635	0.782473
15	C	4.007738	-1.215848	-0.201189
16	C	4.462678	-2.530268	-0.101835
17	C	3.583919	-3.531709	0.328329
18	C	2.2646	-3.222327	0.658285
19	C	1.827923	-1.900119	0.552661
20	O	4.794751	-0.170767	-0.60175
21	C	6.136909	-0.43653	-0.984367
22	O	4.090875	-4.799342	0.402767
23	C	-1.670918	0.583507	-1.485273
24	C	-2.337179	-0.402013	-0.480674
25	C	-3.458892	0.321287	0.288089
26	C	-4.284772	-0.40541	1.321679
27	C	-4.575729	-1.70218	1.492384
28	C	-4.090413	-2.861252	0.648434
29	O	-2.888779	-2.716489	-0.074906
30	C	-2.778949	-1.743297	-1.14049
31	C	-4.069515	-1.626036	-1.964646

32	C	-1.670404	-2.346181	-2.018886
33	C	-5.524407	-2.141481	2.586607
34	H	-1.623	5.204725	-0.453988
35	H	2.19218	0.948961	2.138356
36	H	3.684003	1.467214	1.33576
37	H	2.666983	1.011335	-0.883535
38	H	-3.203227	4.058025	-1.563288
39	H	1.49596	5.015603	1.217014
40	H	5.479979	-2.808545	-0.346025
41	H	1.580228	-4.000075	0.989439
42	H	0.799733	-1.659004	0.799145
43	H	6.557935	0.525585	-1.281088
44	H	6.717817	-0.846009	-0.148361
45	H	6.181249	-1.13204	-1.83191
46	H	3.391952	-5.396882	0.711152
47	H	-2.403474	0.893603	-2.237687
48	H	-0.868391	0.057808	-2.007971
49	H	-1.569939	-0.698397	0.244726
50	H	-4.152521	0.77622	-0.437493
51	H	-2.999977	1.180656	0.793331
52	H	-4.768775	0.281419	2.019211
53	H	-3.898774	-3.716296	1.310554
54	H	-4.917031	-3.178797	-0.012781
55	H	-4.915692	-1.262371	-1.374957
56	H	-3.920142	-0.929158	-2.796424
57	H	-4.339054	-2.599471	-2.389721
58	H	-1.527056	-1.783821	-2.946139
59	H	-0.717986	-2.377545	-1.479077
60	H	-1.946802	-3.372572	-2.279368
61	H	-5.945431	-1.286914	3.124609
62	H	-6.36154	-2.726636	2.17889
63	H	-5.019841	-2.789265	3.317367
**2S, 2"R-1** Conf. 11		Standard Orientation (Ångstroms)		
I	atom	X	Y	Z
1	C	-3.709862	-0.822922	-0.732397
2	C	-4.053452	-2.080808	-0.229367
3	C	-3.049817	-2.979458	0.120635
4	C	-1.684058	-2.608995	-0.037947
5	C	-1.38749	-1.311309	-0.533007
6	C	-2.375781	-0.383018	-0.872223
7	C	-0.629976	-3.575874	0.193219
8	C	0.774255	-3.123773	-0.152088

9	C	0.918369	-1.610615	0.038431
10	O	-0.096029	-0.918477	-0.722922
11	O	-0.845393	-4.725334	0.615081
12	C	2.267265	-1.079107	-0.387347
13	O	-4.687757	0.052548	-1.104484
14	O	-3.391453	-4.189281	0.582355
15	C	3.364409	-1.19647	0.487329
16	C	4.629441	-0.732991	0.115213
17	C	4.807551	-0.148691	-1.145477
18	C	3.734216	-0.029341	-2.028044
19	C	2.481218	-0.497006	-1.637724
20	O	3.098959	-1.790923	1.691498
21	C	4.14588	-1.90935	2.642924
22	O	6.023027	0.320642	-1.558873
23	C	-2.024291	0.983241	-1.430451
24	C	-1.310922	2.008209	-0.494914
25	C	-0.789465	3.190871	-1.354447
26	C	0.45479	3.864033	-0.819574
27	C	0.758858	4.082314	0.464598
28	C	-0.186879	3.746901	1.600665
29	O	-1.556869	3.738915	1.241478
30	C	-2.146408	2.523916	0.722026
31	C	-3.557312	2.983644	0.333486
32	C	-2.234582	1.486985	1.855045
33	C	2.071157	4.691432	0.891134
34	H	-5.091856	-2.382361	-0.123452
35	H	0.977538	-3.382938	-1.200643
36	H	1.493653	-3.661013	0.470909
37	H	0.749448	-1.376484	1.097851
38	H	-5.553284	-0.356734	-0.947063
39	H	-2.540499	-4.686539	0.730309
40	H	5.475532	-0.822044	0.789257
41	H	3.891693	0.428407	-2.998521
42	H	1.638741	-0.395908	-2.313002
43	H	4.964995	-2.533424	2.262761
44	H	4.540538	-0.926135	2.929931
45	H	3.702413	-2.390156	3.516424
46	H	6.674992	0.182983	-0.854538
47	H	-1.340356	0.821923	-2.272892
48	H	-2.925464	1.434729	-1.853193
49	H	-0.427453	1.499821	-0.092398
50	H	-1.5886	3.929865	-1.508159
51	H	-0.549837	2.810961	-2.355642

52	H	1.181717	4.17292	-1.571319
53	H	0.116748	2.804051	2.085324
54	H	-0.09593	4.522905	2.373627
55	H	-4.023229	3.465056	1.199236
56	H	-3.524419	3.720893	-0.475264
57	H	-4.182312	2.145616	0.018026
58	H	-2.726487	1.93924	2.723064
59	H	-2.80812	0.60843	1.549793
60	H	-1.244507	1.13642	2.16738
61	H	2.726937	4.875472	0.034609
62	H	1.918491	5.644471	1.417523
63	H	2.610355	4.033937	1.588824
**2S, 2"R-1** Conf. 12		Standard Orientation (Ångstroms)		
I	atom	X	Y	Z
1	C	2.100845	2.530208	0.522741
2	C	1.640245	3.822074	0.251802
3	C	0.317146	4.01319	-0.134585
4	C	-0.555567	2.893148	-0.246887
5	C	-0.041807	1.601205	0.049168
6	C	1.283178	1.382279	0.433265
7	C	-1.909467	3.063412	-0.729932
8	C	-2.718466	1.793997	-0.904526
9	C	-2.264894	0.718909	0.088075
10	O	-0.84188	0.498881	-0.040255
11	O	-2.393823	4.171985	-1.019148
12	C	-2.965482	-0.604666	-0.109573
13	O	3.390768	2.329243	0.910558
14	O	-0.108693	5.253904	-0.407812
15	C	-4.225676	-0.822165	0.488056
16	C	-4.90603	-2.026429	0.308875
17	C	-4.334339	-3.029818	-0.482063
18	C	-3.0957	-2.831091	-1.092334
19	C	-2.429838	-1.61886	-0.899469
20	O	-4.713604	0.218662	1.228241
21	C	-5.959019	0.056998	1.893045
22	O	-5.052296	-4.185519	-0.616008
23	C	1.789933	0.003086	0.800177
24	C	2.10755	-0.917094	-0.41145
25	C	3.230902	-0.326864	-1.287354
26	C	4.654734	-0.339981	-0.782216
27	C	5.294817	-1.167024	0.05711
28	C	4.724459	-2.362367	0.796273

29	O	3.344318	-2.399862	1.095143
30	C	2.371728	-2.388017	0.031724
31	C	1.126697	-3.016334	0.680016
32	C	2.796623	-3.274267	-1.153225
33	C	6.777117	-0.996419	0.314652
34	H	2.296475	4.684356	0.331865
35	H	-2.579307	1.428002	-1.9315
36	H	-3.780179	2.017988	-0.771644
37	H	-2.451412	1.082863	1.106324
38	H	3.839261	3.187784	0.965549
39	H	-1.060891	5.169952	-0.690166
40	H	-5.871075	-2.216555	0.761208
41	H	-2.652706	-3.610515	-1.708312
42	H	-1.462198	-1.460964	-1.363469
43	H	-5.925175	-0.77291	2.610015
44	H	-6.134514	0.992221	2.427271
45	H	-6.774446	-0.113237	1.178809
46	H	-4.555347	-4.799414	-1.178777
47	H	2.685599	0.096784	1.416278
48	H	1.024654	-0.476635	1.415629
49	H	1.209639	-0.94221	-1.044579
50	H	3.217587	-0.824301	-2.26962
51	H	2.96605	0.714626	-1.507358
52	H	5.268663	0.442131	-1.232035
53	H	5.205407	-2.417994	1.781422
54	H	5.035584	-3.278666	0.262317
55	H	0.882588	-2.525149	1.624641
56	H	0.255231	-2.948427	0.020634
57	H	1.319179	-4.071814	0.89881
58	H	2.005162	-3.286739	-1.911193
59	H	3.714424	-2.929294	-1.635951
60	H	2.954782	-4.30444	-0.815335
61	H	7.206464	-0.203217	-0.304686
62	H	6.974444	-0.751973	1.367935
63	H	7.329608	-1.923257	0.101974
**2S, 2"R-1** Conf. 13		Standard Orientation (Ångstroms)		
I	atom	X	Y	Z
1	C	2.264081	2.466902	0.443796
2	C	1.835936	3.770023	0.173197
3	C	0.512927	3.998296	-0.192867
4	C	-0.395013	2.904526	-0.27965
5	C	0.085973	1.600701	0.016022
6	C	1.4128	1.343244	0.370695

7	C	-1.752842	3.110976	-0.738411
8	C	-2.602797	1.864775	-0.884348
9	C	-2.162302	0.789552	0.113903
10	O	-0.749892	0.522944	-0.043671
11	O	-2.208477	4.231067	-1.028462
12	C	-2.907876	-0.514545	-0.044033
13	O	3.551923	2.233692	0.819588
14	O	0.119802	5.249057	-0.468035
15	C	-4.173426	-0.669891	0.561974
16	C	-4.89532	-1.854801	0.422083
17	C	-4.359795	-2.902279	-0.336864
18	C	-3.115671	-2.766278	-0.95287
19	C	-2.408119	-1.572035	-0.800067
20	O	-4.622215	0.410096	1.270421
21	C	-5.872432	0.314424	1.939283
22	O	-5.117312	-4.035927	-0.433516
23	C	1.874665	-0.059971	0.713202
24	C	2.125495	-0.955309	-0.535853
25	C	3.374002	-0.498985	-1.330551
26	C	4.68437	-0.450031	-0.575405
27	C	5.114229	-1.2857	0.376881
28	C	4.327928	-2.497873	0.83094
29	O	3.466322	-3.040363	-0.153137
30	C	2.135101	-2.50786	-0.290215
31	C	1.271408	-2.960325	0.900129
32	C	1.624185	-3.216005	-1.556708
33	C	6.426465	-1.079975	1.092833
34	H	2.518225	4.613103	0.238197
35	H	-2.492781	1.482317	-1.908831
36	H	-3.654562	2.122144	-0.734933
37	H	-2.312705	1.175481	1.130319
38	H	4.024223	3.080163	0.86199
39	H	-0.839237	5.192164	-0.733066
40	H	-5.865539	-1.996363	0.880928
41	H	-2.69976	-3.580048	-1.542668
42	H	-1.436844	-1.462689	-1.270272
43	H	-5.866905	-0.491388	2.683957
44	H	-6.015308	1.272774	2.441213
45	H	-6.693332	0.148106	1.230491
46	H	-4.644618	-4.682914	-0.979942
47	H	2.778052	0.001987	1.324969
48	H	1.09187	-0.515243	1.323818

49	H	1.263595	-0.781759	-1.192841
50	H	3.485227	-1.133123	-2.221153
51	H	3.172415	0.50863	-1.71417
52	H	5.33583	0.382556	-0.839699
53	H	5.031125	-3.305406	1.077312
54	H	3.787509	-2.273603	1.766405
55	H	1.65426	-2.599995	1.859687
56	H	0.240503	-2.603787	0.798769
57	H	1.25655	-4.054792	0.935871
58	H	1.754553	-4.297501	-1.451125
59	H	0.561266	-3.005749	-1.718111
60	H	2.180507	-2.894949	-2.442669
61	H	6.930981	-0.170338	0.752692
62	H	6.282482	-0.995128	2.179898
63	H	7.107366	-1.928504	0.934884
**2S, 2"R-1** Conf. 14		Standard Orientation (Ångstroms)		
I	atom	X	Y	Z
1	C	2.491539	2.535218	0.475102
2	C	3.641852	1.980804	1.044871
3	C	3.97424	0.659655	0.76502
4	C	3.151396	-0.10849	-0.1093
5	C	1.985228	0.494477	-0.65569
6	C	1.623014	1.814478	-0.37037
7	C	3.526251	-1.45414	-0.47213
8	C	2.673991	-2.13102	-1.52603
9	C	1.222521	-1.64062	-1.55156
10	O	1.179372	-0.17804	-1.51924
11	O	4.518194	-2.03874	0.002534
12	C	0.300643	-2.22557	-0.50125
13	O	2.152207	3.829629	0.737498
14	O	5.077761	0.135294	1.316117
15	C	-1.05012	-2.49042	-0.84034
16	C	-1.94756	-2.98536	0.105814
17	C	-1.51294	-3.21849	1.414624
18	C	-0.18363	-2.99205	1.769897
19	C	0.700199	-2.50256	0.808237
20	O	-1.40739	-2.23867	-2.13465
21	C	-2.70124	-2.62819	-2.57735
22	O	-2.45165	-3.67462	2.296547
23	C	0.367767	2.43287	-0.94963
24	C	-0.94156	1.973367	-0.24728
25	C	-1.04438	2.522387	1.194821
26	C	-1.87499	1.679022	2.135198

27	C	-2.9583	0.95528	1.829189
28	C	-3.58942	0.958871	0.449439
29	O	-3.3992	2.157822	-0.28291
30	C	-2.21067	2.302425	-1.0903
31	C	-2.27571	3.771873	-1.52822
32	C	-2.31766	1.39323	-2.32826
33	C	-3.6447	0.070547	2.841086
34	H	4.284563	2.56041	1.701603
35	H	2.724452	-3.21481	-1.39103
36	H	3.138595	-1.90306	-2.49589
37	H	0.794018	-1.86096	-2.52802
38	H	2.817219	4.214218	1.33011
39	H	5.144616	-0.80016	0.979761
40	H	-2.98305	-3.19013	-0.13443
41	H	0.160858	-3.19079	2.781966
42	H	1.732142	-2.34071	1.100549
43	H	-2.72927	-2.40081	-3.64414
44	H	-2.86721	-3.7019	-2.42493
45	H	-3.48943	-2.06082	-2.06732
46	H	-2.04202	-3.77082	3.170608
47	H	0.452784	3.52139	-0.89388
48	H	0.315837	2.168507	-2.01063
49	H	-0.88631	0.880764	-0.17195
50	H	-1.41413	3.55792	1.179178
51	H	-0.03489	2.585272	1.614786
52	H	-1.52135	1.643901	3.166524
53	H	-4.67801	0.860838	0.561891
54	H	-3.26262	0.074407	-0.12197
55	H	-2.1863	4.447117	-0.67175
56	H	-1.48616	4.011904	-2.24658
57	H	-3.24347	3.961995	-2.00304
58	H	-1.52363	1.606289	-3.0512
59	H	-2.23434	0.333649	-2.06732
60	H	-3.28066	1.562217	-2.82237
61	H	-3.15814	0.132999	3.819986
62	H	-4.70068	0.348677	2.967889
63	H	-3.63239	-0.98449	2.530824

Functional		Solvent?		Basis Set	
mPW1PW91		PCM		6-311G(d,p)	
		DP4+	0.00%	100.00%	-
Nuclei	sp2?	Experimenta	Isomer 1	Isomer 2	Isomer 3
C	x	166.3	172.20	171.30	
C	x	96.3	100.65	99.34	
C	x	163.3	170.65	170.40	
C	x	103.3	106.61	106.74	
C	x	162.5	168.63	168.96	
C	x	108.2	111.72	111.65	
C	x	198.7	202.90	203.04	
C		42.7	46.55	46.58	
C		75.5	80.32	80.25	
C	x	119.2	125.55	125.40	
C	x	159.3	164.28	164.53	
C	x	99.8	100.88	100.88	
C	x	160.5	165.38	165.45	
C	x	108	109.81	110.10	
C	x	129.1	133.64	134.62	
C		55.9	56.61	56.58	
C		26	28.20	27.85	
C		48.9	51.70	52.33	
C		28.5	34.63	31.32	
C	x	126.6	131.52	132.86	
C	x	136.8	147.66	147.82	
C		65.7	70.71	69.70	
C		80.3	83.55	83.21	
C		24.3	28.39	25.83	
C		22.5	24.61	24.13	
C		20.9	24.83	24.19	
H	x	5.94	6.19	6.06	
H		2.66	2.61	2.52	
H		3.13	2.66	2.77	
H		5.61	5.49	5.53	
H	x	6.48	6.51	6.50	
H	x	6.43	6.63	6.68	
H	x	7.32	7.80	7.80	
H		3.81	3.83	3.84	
H		2.20	2.45	2.38	
H		2.40	2.64	2.49	
H		2.20	1.97	2.19	
H		1.75	2.24	1.81	
H		2.18	2.26	2.40	
H	x	5.29	5.75	5.71	
H		3.68	4.13	3.96	
H		4.26	4.42	4.50	
H		1.08	1.15	1.10	
H		1.18	1.26	1.27	
H		1.52	1.65	1.61	

Functional	Solvent?	Basis Set	Type of Data		
mPW1PW91	PCM	6-311G(d,p)	Unscaled Shifts		

	Isomer 1	Isomer 2	Isomer 3	Isomer 4	Isomer 5	Isomer 6
sDP4+ (H data)	0.87%	99.13%	-	-	-	-
sDP4+ (C data)	14.38%	85.62%	-	-	-	-
sDP4+ (all data)	0.15%	99.85%	-	-	-	-
uDP4+ (H data)	0.73%	99.27%	-	-	-	-
uDP4+ (C data)	75.93%	24.07%	-	-	-	-
uDP4+ (all data)	2.27%	97.73%	-	-	-	-
DP4+ (H data)	0.01%	99.99%	-	-	-	-
DP4+ (C data)	34.63%	65.37%	-	-	-	-
DP4+ (all data)	0.00%	100.00%	-	-	-	-

Figure S21. DP4+ analysis of compound **1** with isomers **2S, 2"S-1** and **2S, 2"R-1**.

Table S13. Experimental chemical shifts and Boltzmann-averaged calculated ^{13}C NMR shifts of two diastereomers of 2 (δ in ppm).

no.	2R, 3R, 2"R-2			2R, 3R, 2"S-2		
	δ_C, exptl[a]	δ_C, (adj_calcd)[b]	δ_C, $\Delta\delta$[c]	δ_C, exptl[a]	δ_C, (adj_calcd)[b]	δ_C, $\Delta\delta$[c]
1	164.00	171.2836	7.28	164.00	171.252	7.25
2	97.8	101.229	3.43	97.8	101.435	3.64
3	162.5	169.2548	6.75	162.5	169.1793	6.68
4	102.1	103.7371	1.64	102.1	103.9101	1.81
5	161.6	167.7958	6.20	161.6	167.9298	6.33
6	102.7	107.5502	4.85	102.7	107.3552	4.66
7	199.3	202.9395	3.64	199.3	202.6325	3.33
8	72.5	76.17435	3.67	72.5	76.85606	4.36
9	79.7	83.98376	4.28	79.7	82.23224	2.53
10	116.9	122.079	5.18	116.9	123.196	6.30
11	160.8	166.4308	5.63	160.8	166.2121	5.41
12	100.1	100.779	0.68	100.1	102.1427	2.04
13	161.00	166.0957	5.10	161.00	165.7144	4.71
14	108.2	110.8581	2.66	108.2	111.9915	3.79
15	130.9	137.0828	6.18	130.9	136.238	5.34
16	56	56.32348	0.32	56	57.78203	1.78
17	22.9	25.32537	2.43	22.9	24.94104	2.04
18	42.3	45.45439	3.15	42.3	45.10251	2.80
19	81	85.93853	4.94	81	85.74292	4.74
20	28	29.51936	1.52	28	29.07247	1.07
21	20.5	21.51937	1.02	20.5	22.11256	1.61
22	26.6	28.37688	1.78	26.6	28.32151	1.72
23	42.5	44.40087	1.90	42.5	43.44797	0.95
24	71.3	75.08281	3.78	71.3	74.80164	3.50
25	29.2	32.84145	3.64	29.2	28.8287	0.37

26	29	28.11868	0.88	29	33.13018	4.13

[a]Recorded in CD_3OD at 150 MHz. [b]Calculated in CD_3OD. [c]$\Delta\delta = |\delta_{adj\_calcd}-\delta_{exptl}|$

Table S14. Experimental chemical shifts and Boltzmann-averaged calculated ^{1}H NMR shifts of two diastereomers of 2 (δ in ppm).

no.	2R, 3R, 2"R-2			2R, 3R, 2"S-2		
	δ_H, exptl[a]	δ_H, (adj_calcd)[b]	δ_H, $\Delta\delta$[c]	δ_H, exptl[a]	δ_H, (adj_calcd)[b]	δ_H, $\Delta\delta$[c]
1	5.87	6.105284	0.235284	5.87	6.108414	0.238414
2	4.77	4.603482	0.166518	4.77	4.440173	0.32983
3	5.42	5.242987	0.177013	5.42	5.44605	0.02605
4	6.5	6.410686	0.089314	6.5	6.452346	0.04765
5	6.46	6.788412	0.328412	6.46	6.876775	0.416775
6	7.32	7.734094	0.414094	7.32	7.852474	0.532474
7	3.82	3.752464	0.067536	3.82	3.744096	0.0759
8	3.82	3.729889	0.090111	3.82	3.739969	0.08003
9	3.82	4.00559	0.185590	3.82	4.007806	0.187806
10	2.74	2.671733	0.068267	2.74	2.730715	0.00929
11	2	1.975677	0.024323	2	1.946769	0.05323
12	1.58	1.459443	0.120557	1.58	1.551149	0.02885
13	1.43	1.573396	0.143396	1.43	1.474707	0.044707
14	1.43	1.463281	0.033281	1.43	1.542922	0.112922
15	1.43	1.316074	0.113926	1.43	1.28624	0.14376
16	1.16	1.159768	0.000232	1.16	1.254452	0.094452
17	1.16	1.250439	0.090439	1.16	1.130911	0.02909
18	1.16	1.096561	0.063439	1.16	1.119553	0.04045
19	1.1	1.879093	0.779093	1.1	1.887601	0.787601
20	1.71	1.115696	0.594304	1.71	1.054314	0.65569
21	1.35	1.64568	0.295680	1.35	1.582323	0.232323
22	1.66	1.093782	0.566218	1.66	1.227242	0.43276
23	1.16	1.191758	0.031758	1.16	1.028643	0.13136
24	1.16	1.218398	0.058398	1.16	1.334465	0.174465
25	1.16	1.114696	0.045304	1.16	1.00473	0.15527
26	1.16	0.898629	0.261371	1.16	1.223791	0.063791
27	1.16	1.073416	0.086584	1.16	1.207864	0.047864
28	1.16	1.316318	0.156318	1.16	1.190506	0.030506
29	1.16	0.162381	0.997619	1.16	0.403476	0.75652

[a]Recorded in CD_3OD at 600 MHz. [b]Calculated in CD_3OD. [c]$\Delta\delta = |\delta_{adj\_calcd}-\delta_{exptl}|$

Table S15. Conformers and Bolzmann distributions of the optimized 2R, 3R, 2"R-2

species	*E'=E+ZPE*	*E*	*H*	*G*	*ΔG*	*ΔE(kcal/mol)*	*p%*
1	-1611.275833	-1611.242765	-1611.241821	-1611.340997	0	0	61.38%
2	-1611.274915	-1611.24194	-1611.240996	-1611.339502	0.001495	0.938126702	12.59%
3	-1611.274633	-1611.241657	-1611.240712	-1611.339413	0.001584	0.993975048	11.45%
4	-1611.274043	-1611.241082	-1611.240138	-1611.33847	0.002527	1.585716507	4.22%

5	-1611.27273	-1611.239768	-1611.238824	-1611.338107	0.00289	1.813502455	2.87%
6	-1611.272932	-1611.239896	-1611.238951	-1611.337981	0.003016	1.892568652	2.51%
7	-1611.272467	-1611.239514	-1611.23857	-1611.337549	0.003448	2.163652756	1.59%
8	-1611.272089	-1611.239065	-1611.238121	-1611.337335	0.003662	2.297939789	1.27%
9	-1611.27208	-1611.238904	-1611.23796	-1611.337118	0.003879	2.434109351	1.01%
10	-1611.271835	-1611.238964	-1611.23802	-1611.336618	0.004379	2.7478641	0.59%
11	-1611.271625	-1611.238669	-1611.237725	-1611.336504	0.004493	2.819400183	0.52%

***E*, *E'*, *H, G*: total energy, total energy with zero point energy (ZPE), enthalpy, and Gibbs free energy**

Table S16. Conformers and Bolzmann distributions of the optimized 2R, 3R, 2"S-2

species	*E'=E+ZPE*	*E*	*H*	*G*	*ΔG*	*ΔE(kcal/mol)*	*p%*
1	-1611.275415	-1611.242256	-1611.241312	-1611.341182	0	0	50.50%
2	-1611.274673	-1611.241514	-1611.24057	-1611.340046	0.001136	0.712850792	15.15%
3	-1611.274603	-1611.241519	-1611.240574	-1611.339602	0.00158	0.99146501	9.46%
4	-1611.274282	-1611.241311	-1611.240367	-1611.339023	0.002159	1.35479301	5.12%
5	-1611.273724	-1611.240632	-1611.239688	-1611.338869	0.002313	1.451429473	4.35%
6	-1611.272736	-1611.239696	-1611.238752	-1611.338763	0.002419	1.51794548	3.89%
7	-1611.274051	-1611.241115	-1611.240171	-1611.338474	0.002708	1.699295726	2.86%
8	-1611.273988	-1611.241028	-1611.240084	-1611.338461	0.002721	1.707453349	2.82%
9	-1611.2732	-1611.240269	-1611.239325	-1611.338307	0.002875	1.804089812	2.40%
10	-1611.272939	-1611.239862	-1611.238918	-1611.338188	0.002994	1.878763443	2.11%
11	-1611.272499	-1611.239491	-1611.238547	-1611.337739	0.003443	2.160515208	1.31%

Table S17. Stable conformers of compound 2 with 2R, 3R, 2"R-2 configurations

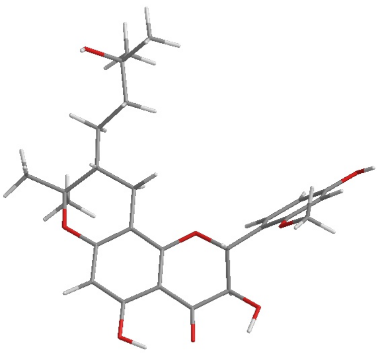

Conformer 1 61.38%

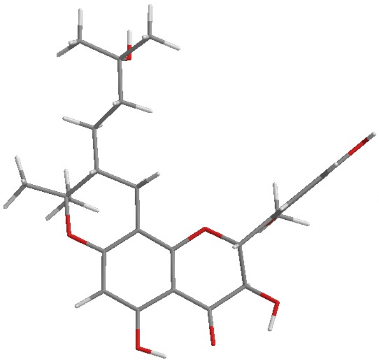

Conformer 2 12.59%

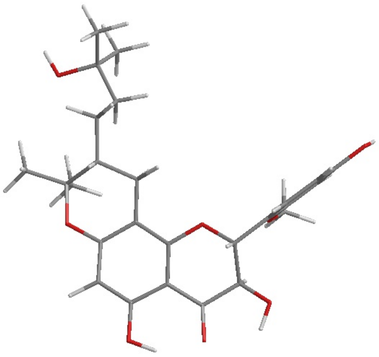

Conformer 3 11.45%

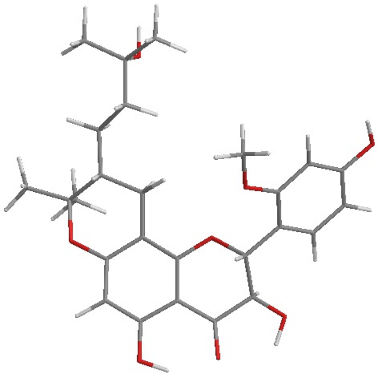

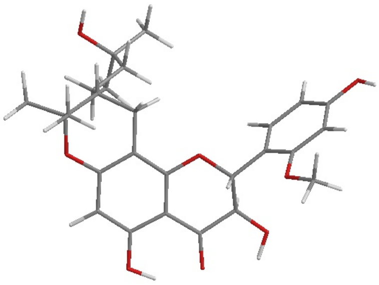

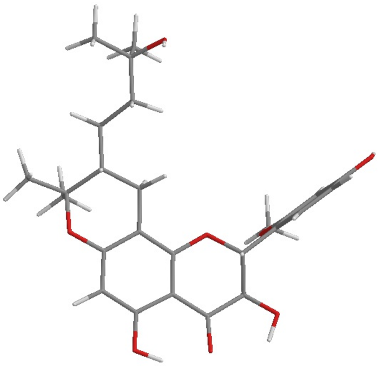

Conformer 4 4.22% **Conformer 5 2.87%** **Conformer 6 2.51%**

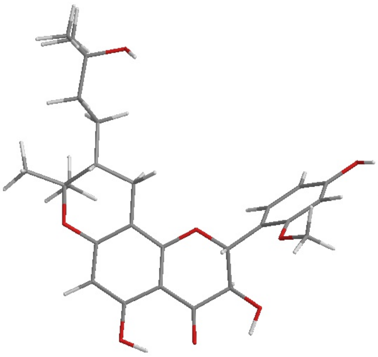

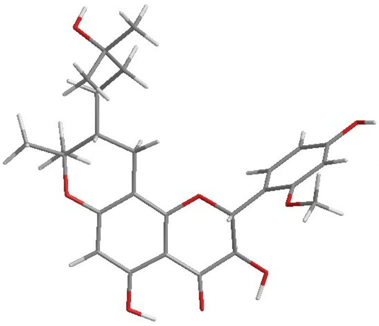

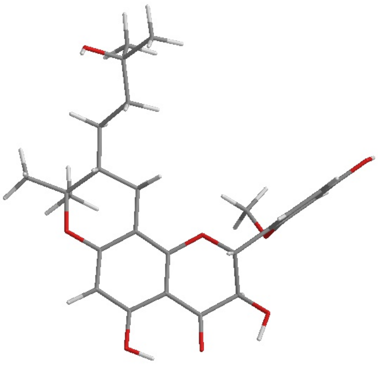

Conformer 7 1.59% **Conformer 8 1.27%** **Conformer 9 1.01%**

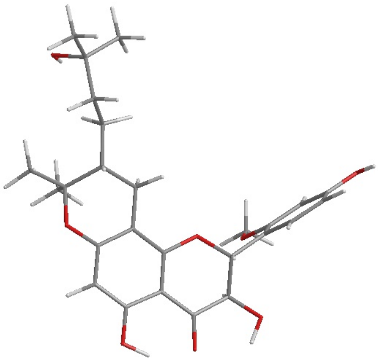

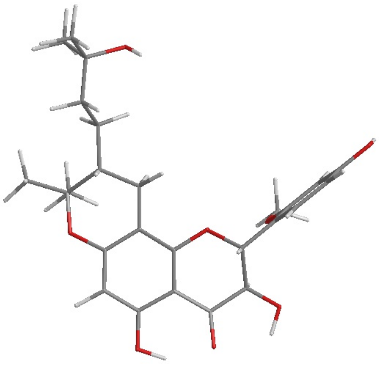

Conformer 10 0.59% **Conformer 11 0.52%**

Table S18. Stable conformers of compound 2 with 2R, 3R, 2"S-2 configurations

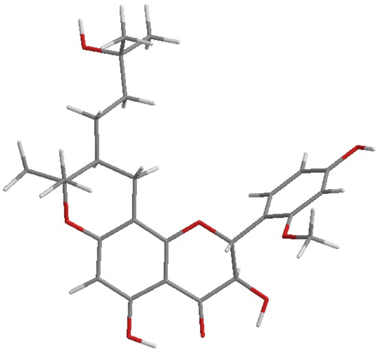

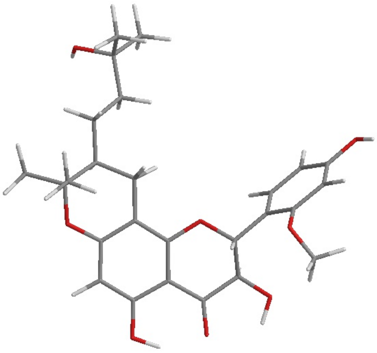

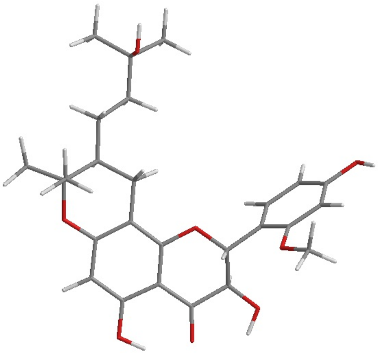

Conformer 1 50.50% **Conformer 2 15.15%** **Conformer 3 9.46%**

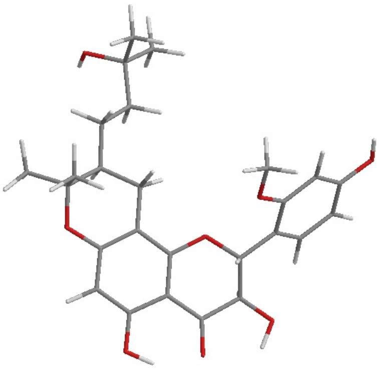

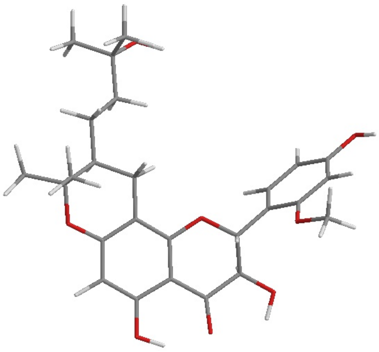

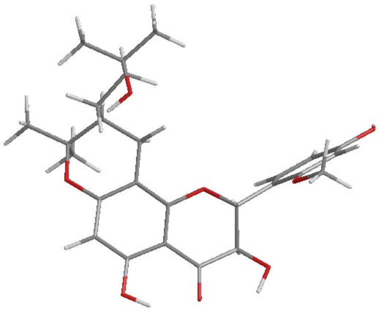

Conformer 4 5.12% **Conformer 5 4.35%** **Conformer 6 3.89%**

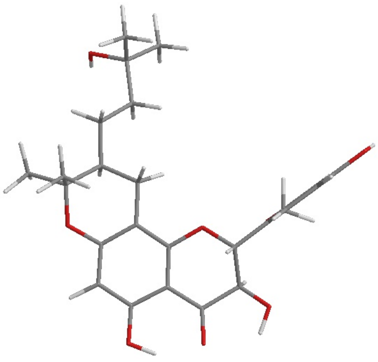

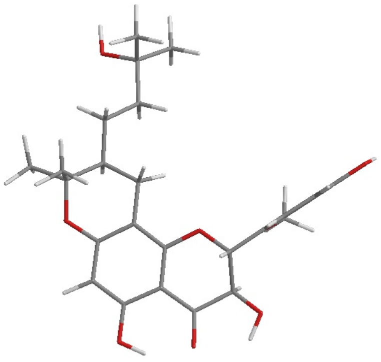

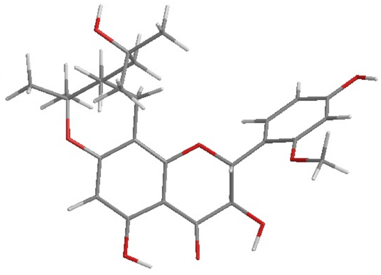

Conformer 7 2.86% **Conformer 8 2.82%** **Conformer 9 2.40%**

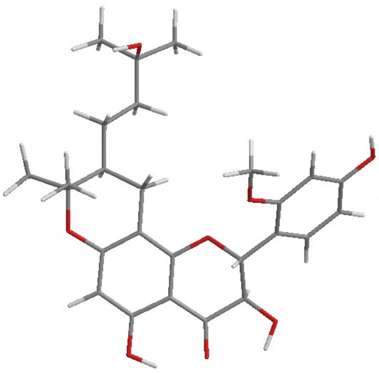

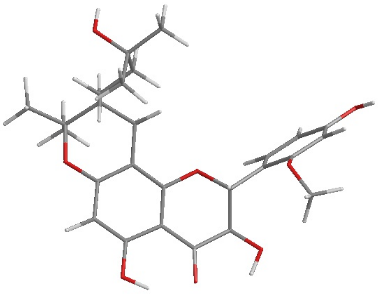

Conformer 10 2.11% **Conformer 11 1.31%**

Table S19. Experimental chemical shifts and calculated ^{13}C NMR shifts of conformers of 2R, 3R, 2"S-2 (δ in ppm).

no.	2R, 3R, 2"S-2 conf. 1			2R, 3R, 2"S-2 conf. 2		
	δ_C, exptl[a]	δ_C, (adj_calcd)[b]	δ_C, $\Delta\delta$[c]	δ_C, exptl[a]	δ_C, (adj_calcd)[b]	δ_C, $\Delta\delta$[c]
1	164.00	171.1894	7.19	164.00	171.3972	7.40
2	97.8	101.4618	3.66	97.8	101.5723	3.77
3	162.5	169.1840	6.68	162.5	169.2083	6.71
4	102.1	103.8959	1.80	102.1	103.8916	1.79
5	161.6	167.8620	6.26	161.6	167.8460	6.25
6	102.7	107.5180	4.82	102.7	107.7243	5.02
7	199.3	202.5968	3.30	199.3	201.9326	2.63
8	72.5	77.9081	5.41	72.5	76.7683	4.27
9	79.7	81.5211	1.82	79.7	81.9813	2.28
10	116.9	122.6969	5.80	116.9	128.2921	11.39
11	160.8	165.4344	4.63	160.8	167.7845	6.98
12	100.1	99.8653	0.23	100.1	112.4312	12.33
13	161.00	165.4996	4.50	161.00	165.4593	4.46
14	108.2	111.2985	3.10	108.2	116.7641	8.56
15	130.9	135.7500	4.85	130.9	135.9854	5.09
16	56	56.2603	0.26	56	65.6132	9.61
17	22.9	25.1586	2.26	22.9	25.3208	2.42
18	42.3	45.3723	3.07	42.3	45.6064	3.31

no.						
19	81	85.9001	4.90	81	85.9656	4.97
20	28	21.1411	6.86	28	21.1256	6.87
21	20.5	29.5690	9.07	20.5	29.5665	9.07
22	26.6	28.7223	2.12	26.6	28.4880	1.89
23	42.5	42.7260	0.23	42.5	44.8097	2.31
24	71.3	74.6495	3.35	71.3	75.1619	3.86
25	29.2	28.7637	0.44	29.2	26.3084	2.89
26	29	34.2956	5.30	29	34.1960	5.20

no.	**2R, 3R, 2"S-2 conf. 3**			**2R, 3R, 2"S-2 conf.4**		
	δ_C, exptl[a]	δ_C, (adj_calcd)[b]	δ_C, $\Delta\delta$[c]	δ_C, exptl[a]	δ_C, (adj_calcd)[b]	δ_C, $\Delta\delta$[c]
1	164.00	171.1840	7.18	164.00	171.1789	7.18
2	97.8	101.4503	3.65	97.8	101.3197	3.52
3	162.5	169.1758	6.68	162.5	169.0982	6.60
4	102.1	103.9283	1.83	102.1	104.0400	1.94
5	161.6	167.9064	6.31	161.6	167.6083	6.01
6	102.7	107.5134	4.81	102.7	107.5155	4.82
7	199.3	202.6002	3.30	199.3	203.3939	4.09
8	72.5	77.9104	5.41	72.5	71.7076	0.79
9	79.7	81.5065	1.81	79.7	81.0217	1.32
10	116.9	122.7089	5.81	116.9	119.3729	2.47
11	160.8	165.4524	4.65	160.8	168.0222	7.22
12	100.1	99.8730	0.23	100.1	101.0497	0.95
13	161.00	165.5127	4.51	161.00	166.7873	5.79
14	108.2	111.2942	3.09	108.2	110.3760	2.18
15	130.9	135.7708	4.87	130.9	136.9720	6.07
16	56	56.2575	0.26	56	56.4231	0.42
17	22.9	24.9497	2.05	22.9	25.2523	2.35
18	42.3	44.5870	2.29	42.3	45.8076	3.51
19	81	85.9229	4.92	81	85.6864	4.69
20	28	21.0120	6.99	28	21.0940	6.91
21	20.5	29.6511	9.15	20.5	29.6378	9.14
22	26.6	28.7530	2.15	26.6	28.2049	1.60
23	42.5	41.6659	0.83	42.5	44.6409	2.14
24	71.3	74.5846	3.28	71.3	75.1133	3.81
25	29.2	34.5441	5.34	29.2	26.3227	2.88
26	29	28.7682	0.23	29	34.0863	5.09

no.	**2R, 3R, 2"S-2 conf.5**			**2R, 3R, 2"S-2 conf.6**		
	δ_C, exptl[a]	δ_C, (adj_calcd)[b]	δ_C, $\Delta\delta$[c]	δ_C, exptl[a]	δ_C, (adj_calcd)[b]	δ_C, $\Delta\delta$[c]
1	164.00	171.0483	7.05	164.00	171.6801	7.68
2	97.8	101.4579	3.66	97.8	101.4515	3.65
3	162.5	169.1900	6.69	162.5	169.1120	6.61
4	102.1	103.9417	1.84	102.1	104.0290	1.93
5	161.6	167.8648	6.26	161.6	168.9858	7.39
6	102.7	107.3610	4.66	102.7	105.9130	3.21

7	199.3	202.6193	3.32	199.3	202.7735	3.47
8	72.5	77.8600	5.36	72.5	78.0190	5.52
9	79.7	81.5099	1.81	79.7	81.5522	1.85
10	116.9	122.6459	5.75	116.9	123.1832	6.28
11	160.8	165.4667	4.67	160.8	165.7555	4.96
12	100.1	99.8796	0.22	100.1	100.3819	0.28
13	161.00	165.5287	4.53	161.00	165.9184	4.92
14	108.2	111.3185	3.12	108.2	110.0810	1.88
15	130.9	135.8077	4.91	130.9	135.2463	4.35
16	56	56.2832	0.28	56	56.4567	0.46
17	22.9	25.1109	2.21	22.9	23.1261	0.23
18	42.3	45.1064	2.81	42.3	42.4299	0.13
19	81	85.5461	4.55	81	84.1556	3.16
20	28	29.5358	1.54	28	28.2171	0.22
21	20.5	20.9859	0.49	20.5	27.7162	7.22
22	26.6	31.5410	4.94	26.6	23.8773	2.72
23	42.5	47.1144	4.61	42.5	43.9863	1.49
24	71.3	75.3610	4.06	71.3	74.2996	3.00
25	29.2	27.5107	1.69	29.2	31.9107	2.71
26	29	30.0496	1.05	29	28.9668	0.03

no.	**2R, 3R, 2"S-2 conf.7**			**2R, 3R, 2"S-2 conf.8**		
	δ_C, exptl[a]	δ_C, (adj_calcd)[b]	δ_C, $\Delta\delta$[c]	δ_C, exptl[a]	δ_C, (adj_calcd)[b]	δ_C, $\Delta\delta$[c]
1	164.00	171.0100	7.01	164.00	171.0445	7.04
2	97.8	101.1339	3.33	97.8	101.1052	3.31
3	162.5	169.2329	6.73	162.5	169.2287	6.73
4	102.1	103.8100	1.71	102.1	103.7742	1.67
5	161.6	167.7964	6.20	161.6	167.7920	6.19
6	102.7	107.2282	4.53	102.7	107.2206	4.52
7	199.3	203.5985	4.30	199.3	203.5403	4.24
8	72.5	72.2602	0.24	72.5	72.2494	0.25
9	79.7	89.9514	10.25	79.7	89.9010	10.20
10	116.9	120.0773	3.18	116.9	120.1112	3.21
11	160.8	167.9265	7.13	160.8	167.9104	7.11
12	100.1	101.5737	1.47	100.1	101.5708	1.47
13	161.00	166.8435	5.84	161.00	166.8430	5.84
14	108.2	110.4346	2.23	108.2	110.4099	2.21
15	130.9	140.7890	9.89	130.9	140.7407	9.84
16	56	56.1204	0.12	56	56.1285	0.13
17	22.9	25.1925	2.29	22.9	25.0267	2.13
18	42.3	45.9223	3.62	42.3	45.7408	3.44
19	81	85.9348	4.93	81	86.0233	5.02
20	28	21.2288	6.77	28	21.2586	6.74
21	20.5	29.6864	9.19	20.5	29.6818	9.18
22	26.6	28.2743	1.67	26.6	28.4260	1.83

23	42.5	44.7830	2.28	42.5	42.6153	0.12
24	71.3	75.1766	3.88	71.3	74.7070	3.41
25	29.2	26.2670	2.93	29.2	28.7185	0.48
26	29	34.3244	5.32	29	34.4027	5.40

no.	**2R, 3R, 2"S-2 conf.9**			**2R, 3R, 2"S-2 conf.10**		
	δ_C, exptl[a]	δ_C, (adj_calcd)[b]	δ_C, $\Delta\delta$[c]	δ_C, exptl[a]	δ_C, (adj_calcd)[b]	δ_C, $\Delta\delta$[c]
1	164.00	171.8763	7.88	164.00	171.0937	7.09
2	97.8	101.3520	3.55	97.8	101.3533	3.55
3	162.5	169.1013	6.60	162.5	169.0900	6.59
4	102.1	103.8769	1.78	102.1	104.1421	2.04
5	161.6	168.6945	7.09	161.6	167.6366	6.04
6	102.7	105.5507	2.85	102.7	107.5215	4.82
7	199.3	202.6531	3.35	199.3	203.3581	4.06
8	72.5	78.0316	5.53	72.5	71.8325	0.67
9	79.7	81.6388	1.94	79.7	81.4772	1.78
10	116.9	122.8473	5.95	116.9	119.7164	2.82
11	160.8	165.4771	4.68	160.8	168.0698	7.27
12	100.1	100.0151	0.08	100.1	100.9905	0.89
13	161.00	165.6909	4.69	161.00	166.6816	5.68
14	108.2	111.0996	2.90	108.2	110.2073	2.01
15	130.9	135.4086	4.51	130.9	136.8765	5.98
16	56	56.2028	0.20	56	56.4298	0.43
17	22.9	22.4573	0.44	22.9	25.0676	2.17
18	42.3	42.9596	0.66	42.3	44.7836	2.48
19	81	84.5833	3.58	81	85.4396	4.44
20	28	28.2361	0.24	28	20.9695	7.03
21	20.5	27.8267	7.33	20.5	29.6231	9.12
22	26.6	23.9798	2.62	26.6	27.6116	1.01
23	42.5	44.7107	2.21	42.5	43.2185	0.72
24	71.3	75.2742	3.97	71.3	74.4070	3.11
25	29.2	26.1753	3.02	29.2	31.9736	2.77
26	29	34.2315	5.23	29	28.5562	0.44

no.	**2R, 3R, 2"S-2 conf.11**		
	δ_C, exptl[a]	δ_C, (adj_calcd)[b]	δ_C, $\Delta\delta$[c]
1	164.00	172.032225	8.03
2	97.8	101.4170	3.62
3	162.5	169.1595	6.66
4	102.1	103.8668	1.77
5	161.6	168.7039	7.10
6	102.7	105.6453	2.95
7	199.3	201.9777	2.68
8	72.5	76.7703	4.27
9	79.7	81.9099	2.21
10	116.9	128.4052	11.51

11	160.8	168.0052	7.21
12	100.1	112.6387	12.54
13	161.00	165.6652	4.67
14	108.2	116.6447	8.44
15	130.9	135.8678	4.97
16	56	65.7511	9.75
17	22.9	22.4613	0.44
18	42.3	42.9382	0.64
19	81	84.6986	3.70
20	28	27.8936	0.11
21	20.5	28.2149	7.71
22	26.6	24.0325	2.57
23	42.5	44.7313	2.23
24	71.3	75.2210	3.92
25	29.2	26.1485	3.05
26	29	34.2506	5.25

[a]Recorded in CD_3OD at 150 MHz. [b]Calculated in CD_3OD. [c]$\Delta\delta = |\delta_{adj\_calcd} - \delta_{exptl}|$

Table S20. Experimental chemical shifts and calculated ^{13}C NMR shifts of conformers of 2R, 3R, 2"R-2 (δ in ppm).

no.	2R, 3R, 2"R-2 conf. 1			2R, 3R, 2"R-2 conf. 2		
	δ_C, exptl[a]	δ_C, (adj_calcd)[b]	δ_C, $\Delta\delta$[c]	δ_C, exptl[a]	δ_C, (adj_calcd)[b]	δ_C, $\Delta\delta$[c]
1	164.00	171.3422	7.34	164.00	171.1133	7.11
2	97.8	101.3369	3.54	97.8	100.9357	3.14
3	162.5	169.2265	6.73	162.5	169.3184	6.82
4	102.1	103.7295	1.63	102.1	103.6553	1.56
5	161.6	167.7867	6.19	161.6	167.7673	6.17
6	102.7	107.7031	5.00	102.7	107.2846	4.58
7	199.3	202.6321	3.33	199.3	203.5417	4.24
8	72.5	78.0294	5.53	72.5	72.3848	0.12
9	79.7	81.5413	1.84	79.7	89.8865	10.19
10	116.9	122.8664	5.97	116.9	120.5632	3.66
11	160.8	165.5106	4.71	160.8	168.3529	7.55
12	100.1	100.0958	0.00	100.1	101.9918	1.89
13	161.00	165.6952	4.70	161.00	167.0498	6.05
14	108.2	111.2947	3.09	108.2	109.1174	0.92
15	130.9	135.6060	4.71	130.9	140.2256	9.33
16	56	56.1978	0.20	56	56.3212	0.32
17	22.9	25.4291	2.53	22.9	24.9929	2.09
18	42.3	45.8961	3.60	42.3	44.4920	2.19
19	81	85.9743	4.97	81	85.9032	4.90
20	28	29.7342	1.73	28	29.6698	1.67
21	20.5	21.1871	0.69	20.5	21.0325	0.53
22	26.6	28.2946	1.69	26.6	28.7699	2.17
23	42.5	44.8597	2.36	42.5	41.6773	0.82

no.	δ_C, exptl[a]	δ_C, (adj_calcd)[b]	δ_C, $\Delta\delta$[c]	δ_C, exptl[a]	δ_C, (adj_calcd)[b]	δ_C, $\Delta\delta$[c]
24	71.3	75.1640	3.86	71.3	74.5357	3.24
25	29.2	34.2851	5.09	29.2	28.7679	0.43
26	29	26.3276	2.67	29	34.4716	5.47

no.	**2R, 3R, 2"R-2 conf. 3**			**2R, 3R, 2"R-2 conf.4**		
	δ_C, exptl[a]	δ_C, (adj_calcd)[b]	δ_C, $\Delta\delta$[c]	δ_C, exptl[a]	δ_C, (adj_calcd)[b]	δ_C, $\Delta\delta$[c]
1	164.00	171.0783	7.08	164.00	171.2592	7.26
2	97.8	100.9570	3.16	97.8	101.2125	3.41
3	162.5	169.3452	6.85	162.5	169.1808	6.68
4	102.1	103.6614	1.56	102.1	104.0669	1.97
5	161.6	167.7397	6.14	161.6	167.6235	6.02
6	102.7	107.2592	4.56	102.7	107.9915	5.29
7	199.3	203.6077	4.31	199.3	203.3416	4.04
8	72.5	72.3947	0.11	72.5	72.1895	0.31
9	79.7	89.9229	10.22	79.7	81.9030	2.20
10	116.9	120.0743	3.17	116.9	120.0355	3.14
11	160.8	168.0432	7.24	160.8	168.2846	7.48
12	100.1	101.6298	1.53	100.1	101.0204	0.92
13	161.00	166.8753	5.88	161.00	166.5988	5.60
14	108.2	110.2325	2.03	108.2	110.3258	2.13
15	130.9	140.8059	9.91	130.9	136.9796	6.08
16	56	56.1072	0.11	56	56.5707	0.57
17	22.9	25.1387	2.24	22.9	25.1144	2.21
18	42.3	45.7509	3.45	42.3	44.6431	2.34
19	81	85.8187	4.82	81	86.2749	5.27
20	28	29.6244	1.62	28	29.7080	1.71
21	20.5	21.1651	0.67	20.5	21.1141	0.61
22	26.6	28.2533	1.65	26.6	28.6601	2.06
23	42.5	44.7285	2.23	42.5	42.1669	0.33
24	71.3	75.1757	3.88	71.3	74.4366	3.14
25	29.2	34.1605	4.96	29.2	28.7207	0.48
26	29	26.3224	2.68	29	34.6463	5.65

no.	**2R, 3R, 2"R-2 conf.5**			**2R, 3R, 2"R-2 conf.6**		
	δ_C, exptl[a]	δ_C, (adj_calcd)[b]	δ_C, $\Delta\delta$[c]	δ_C, exptl[a]	δ_C, (adj_calcd)[b]	δ_C, $\Delta\delta$[c]
1	164.00	171.7973	7.80	164.00	170.9822	6.98
2	97.8	101.4050	3.61	97.8	100.9586	3.16
3	162.5	169.1303	6.63	162.5	169.3377	6.84
4	102.1	104.0841	1.98	102.1	103.7208	1.62
5	161.6	168.7625	7.16	161.6	167.8153	6.22
6	102.7	105.3875	2.69	102.7	107.1887	4.49
7	199.3	202.6902	3.39	199.3	203.6632	4.36
8	72.5	78.0283	5.53	72.5	72.4322	0.07
9	79.7	81.7153	2.02	79.7	89.9692	10.27
10	116.9	122.6679	5.77	116.9	120.0111	3.11
11	160.8	165.4960	4.70	160.8	168.0768	7.28

12	100.1	100.1513	0.05	100.1	101.6142	1.51
13	161.00	165.8448	4.84	161.00	166.8925	5.89
14	108.2	111.1303	2.93	108.2	110.2255	2.03
15	130.9	135.5957	4.70	130.9	140.7655	9.87
16	56	56.1439	0.14	56	56.1057	0.11
17	22.9	22.2170	0.68	22.9	25.0706	2.17
18	42.3	42.8416	0.54	42.3	45.2234	2.92
19	81	85.0152	4.02	81	85.4629	4.46
20	28	28.0879	0.09	28	29.5801	1.58
21	20.5	28.2487	7.75	20.5	21.0316	0.53
22	26.6	23.7485	2.85	26.6	31.3968	4.80
23	42.5	44.9440	2.44	42.5	47.2362	4.74
24	71.3	75.2939	3.99	71.3	75.4768	4.18
25	29.2	34.4374	5.24	29.2	27.3181	1.88
26	29	26.2229	2.78	29	30.1196	1.12

no.	**2R, 3R, 2"R-2 conf.7**			**2R, 3R, 2"R-2 conf.8**		
	δ_C, exptl[a]	δ_C, (adj_calcd)[b]	δ_C, $\Delta\delta$[c]	δ_C, exptl[a]	δ_C, (adj_calcd)[b]	δ_C, $\Delta\delta$[c]
1	164.00	171.3894	7.39	164.00	171.2722	7.27
2	97.8	101.3733	3.57	97.8	101.3721	3.57
3	162.5	169.2821	6.78	162.5	169.2163	6.72
4	102.1	103.7631	1.66	102.1	103.5903	1.49
5	161.6	167.7700	6.17	161.6	167.6710	6.07
6	102.7	108.3055	5.61	102.7	107.7572	5.06
7	199.3	202.7193	3.42	199.3	202.8103	3.51
8	72.5	78.0599	5.56	72.5	78.0002	5.50
9	79.7	81.6139	1.91	79.7	81.5723	1.87
10	116.9	122.7757	5.88	116.9	122.5583	5.66
11	160.8	165.4872	4.69	160.8	165.5719	4.77
12	100.1	100.0558	0.04	100.1	100.0561	0.04
13	161.00	165.6677	4.67	161.00	165.6821	4.68
14	108.2	111.3115	3.11	108.2	111.2471	3.05
15	130.9	135.5565	4.66	130.9	135.6176	4.72
16	56	56.1847	0.18	56	56.2343	0.23
17	22.9	28.5888	5.69	22.9	25.8054	2.91
18	42.3	45.1250	2.83	42.3	44.5761	2.28
19	81	86.5723	5.57	81	86.4605	5.46
20	28	30.4432	2.44	28	21.2315	6.77
21	20.5	20.6918	0.19	20.5	29.6658	9.17
22	26.6	29.6925	3.09	26.6	28.0571	1.46
23	42.5	48.5513	6.05	42.5	41.2312	1.27
24	71.3	75.0767	3.78	71.3	75.9598	4.66
25	29.2	26.3339	2.87	29.2	27.0504	2.15
26	29	34.3424	5.34	29	34.1761	5.18

no.	**2R, 3R, 2"R-2 conf.9**			**2R, 3R, 2"R-2 conf.10**		

	δ_C, exptl[a]	δ_C, (adj_calcd)[b]	δ_C, $\Delta\delta$[c]	δ_C, exptl[a]	δ_C, (adj_calcd)[b]	δ_C, $\Delta\delta$[c]
1	164.00	171.1620	7.16	164.00	171.1222	7.12
2	97.8	101.2461	3.45	97.8	101.0427	3.24
3	162.5	169.4470	6.95	162.5	169.4424	6.94
4	102.1	103.8520	1.75	102.1	103.7080	1.61
5	161.6	167.6687	6.07	161.6	167.6289	6.03
6	102.7	107.4130	4.71	102.7	107.6787	4.98
7	199.3	203.3264	4.03	199.3	203.6228	4.32
8	72.5	73.6378	1.14	72.5	72.3471	0.15
9	79.7	89.9478	10.25	79.7	89.9468	10.25
10	116.9	126.7341	9.83	116.9	120.4517	3.55
11	160.8	170.6153	9.82	160.8	168.2591	7.46
12	100.1	113.8696	13.77	100.1	101.9523	1.85
13	161.00	166.5818	5.58	161.00	167.0030	6.00
14	108.2	115.1694	6.97	108.2	109.1838	0.98
15	130.9	140.2570	9.36	130.9	140.3484	9.45
16	56	64.6793	8.68	56	56.3391	0.34
17	22.9	25.5945	2.69	22.9	28.2474	5.35
18	42.3	45.7229	3.42	42.3	43.1228	0.82
19	81	85.9051	4.91	81	86.2185	5.22
20	28	29.6351	1.64	28	29.7553	1.76
21	20.5	21.2176	0.72	20.5	20.4867	0.01
22	26.6	28.2306	1.63	26.6	29.1025	2.50
23	42.5	44.6763	2.18	42.5	46.7647	4.26
24	71.3	75.1475	3.85	71.3	75.0729	3.77
25	29.2	34.1489	4.95	29.2	31.9810	2.78
26	29	26.2996	2.70	29	28.5978	0.40

no.	**2R, 3R, 2"R-2 conf.11**		
	δ_C, exptl[a]	δ_C, (adj_calcd)[b]	δ_C, $\Delta\delta$[c]
1	164.00	171.1601	7.16
2	97.8	100.9964	3.20
3	162.5	169.3941	6.89
4	102.1	103.6882	1.59
5	161.6	167.7150	6.12
6	102.7	107.8503	5.15
7	199.3	203.6671	4.37
8	72.5	72.4167	0.08
9	79.7	89.9414	10.24
10	116.9	120.0273	3.13
11	160.8	168.0090	7.21
12	100.1	101.5959	1.50
13	161.00	166.8504	5.85
14	108.2	110.2075	2.01
15	130.9	140.7662	9.87

16	56	56.1165	0.12
17	22.9	28.3195	5.42
18	42.3	44.4813	2.18
19	81	86.0870	5.09
20	28	20.5484	7.45
21	20.5	29.7555	9.26
22	26.6	29.5975	3.00
23	42.5	48.4099	5.91
24	71.3	75.1522	3.85
25	29.2	26.3091	2.89
26	29	34.2665	5.27

[a]Recorded in CD_3OD at 150 MHz. [b]Calculated in CD_3OD. [c]$\Delta\delta = |\delta_{adj\_calcd}-\delta_{exptl}|$

Table S21. Experimental chemical shifts and calculated ^{1}H NMR shifts of conformers of 2R, 3R, 2"S-2 and 2R, 3R, 2"R-2 (δ in ppm).

no.	2R, 3R, 2"S -2 conf.1			2R, 3R, 2"S -2 conf.2		
	δ_H, exptl[a]	δ_H, (adj_calcd)[b]	δ_H, $\Delta\delta$[c]	δ_H, exptl[a]	δ_H, (adj_calcd)[b]	δ_H, $\Delta\delta$[c]
1	5.87	6.103992	0.233992	5.87	6.116992	0.246992
2	4.77	4.318792	0.451208	4.77	4.390392	0.379608
3	5.42	5.528592	0.108592	5.42	5.515492	0.095492
4	6.5	6.362792	0.137208	6.5	6.810092	0.310092
5	6.46	6.866692	0.406692	6.46	7.085192	0.625192
6	7.32	7.885492	0.565492	7.32	7.946292	0.626292
7	3.82	3.651992	0.168008	3.82	3.969392	0.149392
8	3.82	3.701992	0.118008	3.82	3.676592	0.143408
9	3.82	4.095992	0.275992	3.82	3.926492	0.106492
10	2.74	2.780392	0.040392	2.74	2.697492	0.042508
11	2	1.879492	0.120508	2	1.861092	0.138908
12	1.58	1.547292	0.032708	1.58	1.525492	0.054508
13	1.43	1.210292	0.219708	1.43	1.205092	0.224908
14	1.43	1.101892	0.328108	1.43	1.102192	0.327808
15	1.43	1.057292	0.372708	1.43	1.060992	0.369008
16	1.16	1.512192	0.352192	1.16	1.522092	0.362092
17	1.16	1.609692	0.449692	1.16	1.637892	0.477892
18	1.16	1.245092	0.085092	1.16	1.243392	0.083392
19	1.1	1.953292	0.853292	1.1	1.971692	0.871692
20	1.71	1.057192	0.652808	1.71	1.031992	0.678008
21	1.35	1.556092	0.206092	1.35	1.673292	0.323292
22	1.66	1.308192	0.351808	1.66	1.098792	0.561208
23	1.16	0.994792	0.165208	1.16	1.059892	0.100108
24	1.16	1.444592	0.284592	1.16	1.319492	0.159492
25	1.16	0.994092	0.165908	1.16	0.773292	0.386708
26	1.16	1.219692	0.059692	1.16	1.058992	0.101008
27	1.16	1.289492	0.129492	1.16	1.295492	0.135492

28	1.16	1.177692	0.017692	1.16	1.319692	0.159692

no.	2R, 3R, 2"S -2 conf.3			2R, 3R, 2"S -2 conf.4		
	δ_H, exptl[a]	δ_H, (adj_calcd)[b]	δ_H, $\Delta\delta$[c]	δ_H, exptl[a]	δ_H, (adj_calcd)[b]	δ_H, $\Delta\delta$[c]
1	5.87	6.104992	0.234992	5.87	6.103692	0.233692
2	4.77	4.321592	0.448408	4.77	4.821592	0.051592
3	5.42	5.538692	0.118692	5.42	5.298292	0.121708
4	6.5	6.371992	0.128008	6.5	6.427692	0.072308
5	6.46	6.876592	0.416592	6.46	6.807192	0.347192
6	7.32	7.895592	0.575592	7.32	7.827692	0.507692
7	3.82	3.661992	0.158008	3.82	3.608492	0.211508
8	3.82	3.701592	0.118408	3.82	3.796392	0.023608
9	3.82	4.098092	0.278092	3.82	4.131092	0.311092
10	2.74	2.841992	0.101992	2.74	2.513992	0.226008
11	2	1.870592	0.129408	2	1.903392	0.096608
12	1.58	1.579992	8.33E-06	1.58	1.436992	0.143008
13	1.43	1.190892	0.239108	1.43	1.285792	0.144208
14	1.43	1.096792	0.333208	1.43	1.139192	0.290808
15	1.43	1.040992	0.389008	1.43	1.084892	0.345108
16	1.16	1.519292	0.359292	1.16	1.634092	0.474092
17	1.16	1.606992	0.446992	1.16	1.236492	0.076492
18	1.16	1.255992	0.095992	1.16	1.519892	0.359892
19	1.1	1.683692	0.583692	1.1	1.958592	0.858592
20	1.71	1.284992	0.425008	1.71	1.068492	0.641508
21	1.35	1.677292	0.327292	1.35	1.606492	0.256492
22	1.66	1.203592	0.456408	1.66	0.992092	0.667908
23	1.16	1.180592	0.020592	1.16	1.069792	0.090208
24	1.16	1.207892	0.047892	1.16	1.326892	0.166892
25	1.16	1.316792	0.156792	1.16	0.818592	0.341408
26	1.16	1.521192	0.361192	1.16	1.293192	0.133192
27	1.16	1.055192	0.104808	1.16	0.983292	0.176708
28	1.16	1.041492	0.118508	1.16	1.218592	0.058592

no.	2R, 3R, 2"S -2 conf.5			2R, 3R, 2"S -2 conf.6		
	δ_H, exptl[a]	δ_H, (adj_calcd)[b]	δ_H, $\Delta\delta$[c]	δ_H, exptl[a]	δ_H, (adj_calcd)[b]	δ_H, $\Delta\delta$[c]
1	5.87	6.107292	0.237292	5.87	6.143392	0.273392
2	4.77	4.328192	0.441808	4.77	4.322292	0.447708
3	5.42	5.541892	0.121892	5.42	5.489592	0.069592
4	6.5	6.369492	0.130508	6.5	6.528392	0.028392
5	6.46	6.865292	0.405292	6.46	6.666492	0.206492
6	7.32	7.884292	0.564292	7.32	7.824892	0.504892
7	3.82	3.707592	0.112408	3.82	4.099592	0.279592
8	3.82	4.098792	0.278792	3.82	3.667392	0.152608
9	3.82	3.656192	0.163808	3.82	3.711792	0.108208
10	2.74	2.822492	0.082492	2.74	2.548492	0.191508
11	2	1.884092	0.115908	2	2.661592	0.661592

12	1.58	1.602892	0.022892	1.58	1.601692	0.021692
13	1.43	1.575692	0.145692	1.43	1.786292	0.356292
14	1.43	1.259192	0.170808	1.43	1.173392	0.256608
15	1.43	1.520492	0.090492	1.43	1.492992	0.062992
16	1.16	1.046292	0.113708	1.16	1.209492	0.049492
17	1.16	1.194192	0.034192	1.16	1.163692	0.003692
18	1.16	1.059292	0.100708	1.16	1.624392	0.464392
19	1.1	1.550792	0.450792	1.1	1.679992	0.579992
20	1.71	0.961192	0.748808	1.71	0.783292	0.926708
21	1.35	1.552292	0.202292	1.35	1.479592	0.129592
22	1.66	1.422392	0.237608	1.66	1.188992	0.471008
23	1.16	1.167892	0.007892	1.16	1.032692	0.127308
24	1.16	1.012592	0.147408	1.16	1.183092	0.023092
25	1.16	1.287492	0.127492	1.16	1.032392	0.127608
26	1.16	1.182692	0.022692	1.16	1.114592	0.045408
27	1.16	0.849592	0.310408	1.16	1.331692	0.171692
28	1.16	1.363392	0.203392	1.16	1.000092	0.159908

no.	**2R, 3R, 2"S -2 conf.7**			**2R, 3R, 2"S -2 conf.8**		
	δ_H, exptl[a]	δ_H, (adj_calcd)[b]	δ_H, $\Delta\delta$[c]	δ_H, exptl[a]	δ_H, (adj_calcd)[b]	δ_H, $\Delta\delta$[c]
1	5.87	6.086092	0.216092	5.87	6.086092	0.216092
2	4.77	5.282692	0.512692	4.77	5.276792	0.506792
3	5.42	4.745392	0.674608	5.42	4.750792	0.669208
4	6.5	6.436792	0.063208	6.5	6.439092	0.060908
5	6.46	6.703392	0.243392	6.46	6.702592	0.242592
6	7.32	7.410192	0.090192	7.32	7.412092	0.092092
7	3.82	3.778692	0.041308	3.82	3.778192	0.041808
8	3.82	3.684392	0.135608	3.82	3.694192	0.125808
9	3.82	4.151192	0.331192	3.82	4.156992	0.336992
10	2.74	2.664992	0.075008	2.74	2.700792	0.039208
11	2	1.885792	0.114208	2	1.881792	0.118208
12	1.58	1.509592	0.070408	1.58	1.524992	0.055008
13	1.43	1.210992	0.219008	1.43	1.047892	0.382108
14	1.43	1.092492	0.337508	1.43	1.213292	0.216708
15	1.43	1.045392	0.384608	1.43	1.090992	0.339008
16	1.16	1.512092	0.352092	1.16	1.510492	0.350492
17	1.16	1.626392	0.466392	1.16	1.608092	0.448092
18	1.16	1.235092	0.075092	1.16	1.239792	0.079792
19	1.1	1.946792	0.846792	1.1	1.933392	0.833392
20	1.71	1.027592	0.682408	1.71	1.043892	0.666108
21	1.35	1.687192	0.337192	1.35	1.535592	0.185592
22	1.66	1.094192	0.565808	1.66	1.274492	0.385508
23	1.16	1.046492	0.113508	1.16	0.982692	0.177308
24	1.16	1.315792	0.155792	1.16	1.440092	0.280092
25	1.16	0.787792	0.372208	1.16	1.002192	0.157808

no.	δ_H, exptl[a]	δ_H, (adj_calcd)[b]	δ_H, $\Delta\delta$[c]	δ_H, exptl[a]	δ_H, (adj_calcd)[b]	δ_H, $\Delta\delta$[c]
26	1.16	1.292792	0.132792	1.16	1.155892	0.004108
27	1.16	1.080692	0.079308	1.16	1.213392	0.053392
28	1.16	1.290992	0.130992	1.16	1.258892	0.098892

no.	**2R, 3R, 2"S -2 conf.9**			**2R, 3R, 2"S -2 conf.10**		
	δ_H, exptl[a]	δ_H, (adj_calcd)[b]	δ_H, $\Delta\delta$[c]	δ_H, exptl[a]	δ_H, (adj_calcd)[b]	δ_H, $\Delta\delta$[c]
1	5.87	6.142092	0.272092	5.87	6.113092	0.243092
2	4.77	4.277992	0.492008	4.77	4.817892	0.047892
3	5.42	5.462592	0.042592	5.42	5.292492	0.127508
4	6.5	6.339892	0.160108	6.5	6.438092	0.061908
5	6.46	6.823992	0.363992	6.46	6.809592	0.349592
6	7.32	7.874192	0.554192	7.32	7.836092	0.516092
7	3.82	3.707392	0.112608	3.82	3.803492	0.016508
8	3.82	4.090692	0.270692	3.82	4.138292	0.318292
9	3.82	3.623792	0.196208	3.82	3.631192	0.188808
10	2.74	2.534092	0.205908	2.74	2.608292	0.131708
11	2	2.610692	0.610692	2	1.921592	0.078408
12	1.58	1.628192	0.048192	1.58	1.567792	0.012208
13	1.43	1.167592	0.262408	1.43	1.283492	0.146508
14	1.43	1.463092	0.033092	1.43	1.140792	0.289208
15	1.43	1.766792	0.336792	1.43	1.081692	0.348308
16	1.16	1.157292	0.002708	1.16	1.610792	0.450792
17	1.16	1.625692	0.465692	1.16	1.265392	0.105392
18	1.16	1.249092	0.089092	1.16	1.538892	0.378892
19	1.1	1.947392	0.847392	1.1	1.631992	0.531992
20	1.71	1.004092	0.705908	1.71	1.067592	0.642408
21	1.35	1.456992	0.106992	1.35	1.564092	0.214092
22	1.66	0.968392	0.691608	1.66	1.136892	0.523108
23	1.16	0.648092	0.511908	1.16	1.071992	0.088008
24	1.16	0.986792	0.173208	1.16	1.207892	0.047892
25	1.16	1.198992	0.038992	1.16	1.089192	0.070808
26	1.16	1.255892	0.095892	1.16	1.371592	0.211592
27	1.16	0.990392	0.169608	1.16	0.972692	0.187308
28	1.16	1.174192	0.014192	1.16	1.122292	0.037708

no.	**2R, 3R, 2"S -2 conf.11**		
	δ_H, exptl[a]	δ_H, (adj_calcd)[b]	δ_H, $\Delta\delta$[c]
1	5.87	6.148092	0.278092
2	4.77	4.374992	0.395008
3	5.42	5.449592	0.029592
4	6.5	6.804592	0.304592
5	6.46	7.059392	0.599392
6	7.32	7.936992	0.616992
7	3.82	3.979292	0.159292
8	3.82	3.680192	0.139808
9	3.82	3.924092	0.104092

10	2.74	2.492692	0.247308
11	2	2.576792	0.576792
12	1.58	1.607692	0.027692
13	1.43	1.616592	0.186592
14	1.43	1.257692	0.172308
15	1.43	1.159692	0.270308
16	1.16	1.171592	0.011592
17	1.16	1.470292	0.310292
18	1.16	1.774792	0.614792
19	1.1	1.960192	0.860192
20	1.71	0.971392	0.738608
21	1.35	1.447492	0.097492
22	1.66	0.964892	0.695108
23	1.16	0.645392	0.514608
24	1.16	0.988792	0.171208
25	1.16	1.200892	0.040892
26	1.16	1.257192	0.097192
27	1.16	0.984592	0.175408
28	1.16	1.182892	0.022892

no.	**2R, 3R, 2"R -2 conf.1**			**2R, 3R, 2"R -2 conf.2**		
	δ_H, exptl[a]	δ_H, (adj_calcd)[b]	δ_H, $\Delta\delta$[c]	δ_H, exptl[a]	δ_H, (adj_calcd)[b]	δ_H, $\Delta\delta$[c]
1	5.87	6.112092	0.242092	5.87	6.085592	0.215592
2	4.77	4.294392	0.475608	4.77	5.278792	0.508792
3	5.42	5.477292	0.057292	5.42	4.708592	0.711408
4	6.5	6.346792	0.153208	6.5	6.629992	0.129992
5	6.46	6.853492	0.393492	6.46	6.543592	0.083592
6	7.32	7.872692	0.552692	7.32	7.365292	0.045292
7	3.82	3.646292	0.173708	3.82	4.137692	0.317692
8	3.82	3.691492	0.128508	3.82	3.752692	0.067308
9	3.82	4.082492	0.262492	3.82	3.730892	0.089108
10	2.74	2.664392	0.075608	2.74	2.732192	0.007808
11	2	1.977992	0.022008	2	1.869492	0.130508
12	1.58	1.439292	0.140708	1.58	1.477392	0.102608
13	1.43	1.627792	0.197792	1.43	1.522392	0.092392
14	1.43	1.522792	0.092792	1.43	1.239092	0.190908
15	1.43	1.244992	0.185008	1.43	1.612592	0.182592
16	1.16	1.122892	0.037108	1.16	1.251192	0.091192
17	1.16	1.287592	0.127592	1.16	1.059192	0.100808
18	1.16	1.069292	0.090708	1.16	1.114692	0.045308
19	1.1	1.962592	0.862592	1.1	1.662592	0.562592
20	1.71	1.067492	0.642508	1.71	1.285992	0.424008
21	1.35	1.672992	0.322992	1.35	1.580692	0.230692
22	1.66	1.037392	0.622608	1.66	1.130292	0.529708
23	1.16	1.238692	0.078692	1.16	1.030692	0.129308

24	1.16	1.273892	0.113892	1.16	1.022192	0.137808
25	1.16	1.021092	0.138908	1.16	1.499792	0.339792
26	1.16	0.823992	0.336008	1.16	1.132692	0.027308
27	1.16	1.057792	0.102208	1.16	1.111592	0.048408
28	1.16	1.336192	0.176192	1.16	1.231392	0.071392
no.	**2R, 3R, 2"R -2 conf.3**			**2R, 3R, 2"R -2 conf.4**		
	δ_H, exptl[a]	δ_H, (adj_calcd)[b]	δ_H, $\Delta\delta$[c]	δ_H, exptl[a]	δ_H, (adj_calcd)[b]	δ_H, $\Delta\delta$[c]
1	5.87	6.083292	0.213292	5.87	6.111992	0.241992
2	4.77	5.303392	0.533392	4.77	4.801892	0.031892
3	5.42	4.698692	0.721308	5.42	5.197392	0.222608
4	6.5	6.444392	0.055608	6.5	6.460992	0.039008
5	6.46	6.698092	0.238092	6.46	6.809792	0.349792
6	7.32	7.413092	0.093092	7.32	7.856292	0.536292
7	3.82	3.750292	0.069708	3.82	4.154892	0.334892
8	3.82	3.702592	0.117408	3.82	3.645392	0.174608
9	3.82	4.139592	0.319592	3.82	3.882392	0.062392
10	2.74	2.628892	0.111108	2.74	2.865992	0.125992
11	2	1.889392	0.110608	2	1.788392	0.211608
12	1.58	1.421292	0.158708	1.58	1.537192	0.042808
13	1.43	1.621492	0.191492	1.43	1.536292	0.106292
14	1.43	1.516792	0.086792	1.43	1.258492	0.171508
15	1.43	1.223892	0.206108	1.43	1.634392	0.204392
16	1.16	1.124292	0.035708	1.16	1.187792	0.027792
17	1.16	1.269792	0.109792	1.16	1.029192	0.130808
18	1.16	1.076292	0.083708	1.16	1.068192	0.091808
19	1.1	1.936192	0.836192	1.1	1.688392	0.588392
20	1.71	1.045992	0.664008	1.71	1.275092	0.434908
21	1.35	1.629092	0.279092	1.35	1.734892	0.384892
22	1.66	1.023592	0.636408	1.66	1.258492	0.401508
23	1.16	1.224192	0.064192	1.16	1.089592	0.070408
24	1.16	1.269192	0.109192	1.16	1.057392	0.102608
25	1.16	1.008792	0.151208	1.16	1.533692	0.373692
26	1.16	0.790692	0.369308	1.16	1.209792	0.049792
27	1.16	1.049792	0.110208	1.16	1.276792	0.116792
28	1.16	1.320292	0.160292	1.16	1.343592	0.183592
no.	**2R, 3R, 2"R -2 conf.5**			**2R, 3R, 2"R -2 conf.6**		
	δ_H, exptl[a]	δ_H, (adj_calcd)[b]	δ_H, $\Delta\delta$[c]	δ_H, exptl[a]	δ_H, (adj_calcd)[b]	δ_H, $\Delta\delta$[c]
1	5.87	6.135092	0.265092	5.87	6.088192	0.218192
2	4.77	4.299692	0.470308	4.77	5.301092	0.531092
3	5.42	5.496192	0.076192	5.42	4.703792	0.716208
4	6.5	6.362592	0.137408	6.5	6.447392	0.052608
5	6.46	6.841092	0.381092	6.46	6.696892	0.236892
6	7.32	7.887192	0.567192	7.32	7.409892	0.089892
7	3.82	3.642292	0.177708	3.82	3.707892	0.112108

no.						
8	3.82	3.700392	0.119608	3.82	4.143192	0.323192
9	3.82	4.082292	0.262292	3.82	3.755292	0.064708
10	2.74	2.506492	0.233508	2.74	2.703892	0.036108
11	2	2.746392	0.746392	2	1.893392	0.106608
12	1.58	1.612992	0.032992	1.58	1.490892	0.089108
13	1.43	1.091592	0.338408	1.43	1.573692	0.143692
14	1.43	1.235092	0.194908	1.43	1.519592	0.089592
15	1.43	1.529592	0.099592	1.43	1.235092	0.194908
16	1.16	1.130792	0.029208	1.16	1.255592	0.095592
17	1.16	1.712292	0.552292	1.16	1.061792	0.098208
18	1.16	1.455192	0.295192	1.16	1.084092	0.075908
19	1.1	1.965792	0.865792	1.1	1.496192	0.396192
20	1.71	1.118592	0.591408	1.71	0.988992	0.721008
21	1.35	1.721392	0.371392	1.35	1.652392	0.302392
22	1.66	1.079792	0.580208	1.66	1.156092	0.503908
23	1.16	1.364592	0.204592	1.16	0.914092	0.245908
24	1.16	1.340492	0.180492	1.16	1.172792	0.012792
25	1.16	1.115892	0.044108	1.16	1.125192	0.034808
26	1.16	0.746092	0.413908	1.16	1.199192	0.039192
27	1.16	1.035592	0.124408	1.16	0.914092	0.245908
28	1.16	1.258092	0.098092	1.16	1.480192	0.320192

no.	**2R, 3R, 2"R -2 conf.7**			**2R, 3R, 2"R -2 conf.8**		
	δ_H, exptl[a]	δ_H, (adj_calcd)[b]	δ_H, $\Delta\delta$[c]	δ_H, exptl[a]	δ_H, (adj_calcd)[b]	δ_H, $\Delta\delta$[c]
1	5.87	6.115492	0.245492	5.87	6.117292	0.247292
2	4.77	4.294992	0.475008	4.77	4.301792	0.468208
3	5.42	5.483892	0.063892	5.42	5.480792	0.060792
4	6.5	6.338892	0.161108	6.5	6.344992	0.155008
5	6.46	6.850992	0.390992	6.46	6.846692	0.386692
6	7.32	7.875292	0.555292	7.32	7.883992	0.563992
7	3.82	3.689192	0.130808	3.82	3.697692	0.122308
8	3.82	4.082292	0.262292	3.82	4.089092	0.269092
9	3.82	3.642392	0.177608	3.82	3.643792	0.176208
10	2.74	2.540992	0.199008	2.74	2.860592	0.120592
11	2.00	2.222392	0.222392	2.00	1.919692	0.080308
12	1.58	1.380192	0.199808	1.58	1.624892	0.044892
13	1.43	1.437592	0.007592	1.43	1.106392	0.323608
14	1.43	1.533892	0.103892	1.43	1.281792	0.148208
15	1.43	1.249092	0.180908	1.43	1.076192	0.353808
16	1.16	1.277592	0.117592	1.16	1.732492	0.572492
17	1.16	1.010992	0.149008	1.16	1.547492	0.387492
18	1.16	1.237692	0.077692	1.16	1.354292	0.194292
19	1.1	1.425092	0.325092	1.1	2.110192	1.010192
20	1.71	1.471292	0.238708	1.71	1.024192	0.685808
21	1.35	1.298692	0.051308	1.35	1.412492	0.062492

22	1.66	1.853292	0.193292	1.66	1.499092	0.160908
23	1.16	0.863992	0.296008	1.16	1.415092	0.255092
24	1.16	1.072592	0.087408	1.16	0.803992	0.356008
25	1.16	1.326392	0.166392	1.16	1.056992	0.103008
26	1.16	1.273192	0.113192	1.16	0.956392	0.203608
27	1.16	1.061392	0.098608	1.16	1.212292	0.052292
28	1.16	1.267092	0.107092	1.16	1.238792	0.078792
no.	**2R, 3R, 2"R -2 conf.9**			**2R, 3R, 2"R –2 conf.10**		
	δ_H, exptl[a]	δ_H, (adj_calcd)[b]	δ_H, $\Delta\delta$[c]	δ_H, exptl[a]	δ_H, (adj_calcd)[b]	δ_H, $\Delta\delta$[c]
1	5.87	6.105392	0.235392	5.87	6.092192	0.222192
2	4.77	5.244692	0.474692	4.77	5.299392	0.529392
3	5.42	4.703892	0.716108	5.42	4.718192	0.701808
4	6.5	6.908392	0.408392	6.5	6.623992	0.123992
5	6.46	6.901892	0.441892	6.46	6.539492	0.079492
6	7.32	7.468192	0.148192	7.32	7.369592	0.049592
7	3.82	3.819592	0.000408	3.82	3.719692	0.100308
8	3.82	3.932492	0.112492	3.82	4.144192	0.324192
9	3.82	4.009992	0.189992	3.82	3.769892	0.050108
10	2.74	2.676092	0.063908	2.74	2.570492	0.169508
11	2.00	1.956892	0.043108	2.00	2.158592	0.158592
12	1.58	1.432092	0.147908	1.58	1.426792	0.153208
13	1.43	1.228192	0.201808	1.43	1.259492	0.170508
14	1.43	1.626092	0.196092	1.43	1.503792	0.073792
15	1.43	1.522992	0.092992	1.43	1.481792	0.051792
16	1.16	1.117792	0.042208	1.16	1.257592	0.097592
17	1.16	1.267392	0.107392	1.16	1.294592	0.134592
18	1.16	1.083192	0.076808	1.16	0.989592	0.170408
19	1.1	1.940792	0.840792	1.1	1.325992	0.225992
20	1.71	1.037992	0.672008	1.71	1.170492	0.539508
21	1.35	1.629392	0.279392	1.35	1.653492	0.303492
22	1.66	1.030292	0.629708	1.66	1.554792	0.105208
23	1.16	1.220092	0.060092	1.16	1.069692	0.090308
24	1.16	1.264392	0.104392	1.16	1.213292	0.053292
25	1.16	1.008592	0.151408	1.16	1.162192	0.002192
26	1.16	0.770492	0.389508	1.16	1.082992	0.077008
27	1.16	1.040392	0.119608	1.16	1.206692	0.046692
28	1.16	1.310692	0.150692	1.16	0.991192	0.168808
no.	**2R, 3R, 2"R -2 conf.11**					
	δ_H, exptl[a]	δ_H, (adj_calcd)[b]	δ_H, $\Delta\delta$[c]			
1	5.87	6.085792	0.215792			
2	4.77	5.288592	0.518592			
3	5.42	4.707092	0.712908			
4	6.5	6.440092	0.059908			
5	6.46	6.693992	0.233992			

6	7.32	7.412792	0.092792
7	3.82	4.140292	0.320292
8	3.82	3.740092	0.079908
9	3.82	3.712092	0.107908
10	2.74	2.543492	0.196508
11	2	2.117692	0.117692
12	1.58	1.342392	0.237608
13	1.43	1.009192	0.420808
14	1.43	1.225692	0.204308
15	1.43	1.278892	0.151108
16	1.16	1.408292	0.248292
17	1.16	1.495192	0.335192
18	1.16	1.219192	0.059192
19	1.1	1.408792	0.308792
20	1.71	1.402292	0.307708
21	1.35	1.318092	0.031908
22	1.66	1.756092	0.096092
23	1.16	0.852592	0.307408
24	1.16	1.068292	0.091708
25	1.16	1.324892	0.164892
26	1.16	1.280492	0.120492
27	1.16	1.041692	0.118308
28	1.16	1.265892	0.105892

Table S22. The coordinate for the lowe-energy conformer 2R, 3R, 2"S-2 and 2R, 3R, 2"R-2 in NMR and ECD calculations

**2R, 3R, 2"S-2** Conf. 1		Standard Orientation (Ångstroms)		
I	atom	X	Y	Z
1	C	-1.59161	-2.81296	-0.01623
2	C	-0.90639	-4.02144	-0.21216
3	C	0.464097	-4.00148	-0.42523
4	C	1.163602	-2.7563	-0.45049
5	C	0.433783	-1.55994	-0.22881
6	C	-0.94354	-1.56065	-0.02135
7	C	2.560527	-2.68988	-0.76607
8	C	3.157335	-1.29923	-0.96229
9	C	2.484837	-0.32639	0.02089
10	O	1.051959	-0.34623	-0.22408
11	O	3.305356	-3.68024	-0.91034
12	O	4.547401	-1.32729	-0.77482
13	C	2.955485	1.097076	-0.11911
14	C	3.873504	1.643882	0.798557
15	C	4.336797	2.95519	0.641204

16	C	3.882887	3.729343	-0.43273
17	C	2.96738	3.206969	-1.34738
18	C	2.523098	1.899016	-1.17782
19	O	4.307139	5.014524	-0.62661
20	O	4.256838	0.833453	1.826449
21	C	5.211988	1.31191	2.758134
22	C	-1.72207	-0.28304	0.168421
23	C	-3.23164	-0.5014	-0.0364
24	C	-3.69398	-1.79	0.691509
25	O	-2.92502	-2.92638	0.175257
26	C	-3.49098	-1.75448	2.212455
27	C	-5.13529	-2.17095	0.347473
28	C	-4.04982	0.748111	0.346954
29	C	-3.73209	1.972245	-0.52717
30	C	-4.69138	3.165534	-0.34657
31	C	-4.68766	3.706489	1.092171
32	C	-4.32349	4.280978	-1.33736
33	O	-5.99806	2.654946	-0.66943
34	O	1.119699	-5.15871	-0.61432
35	H	-1.44715	-4.95976	-0.18787
36	H	2.899092	-0.97419	-1.98801
37	H	2.664567	-0.68359	1.040352
38	H	4.791449	-2.26963	-0.86791
39	H	5.045863	3.381588	1.34368
40	H	2.623788	3.823976	-2.17043
41	H	1.804458	1.487732	-1.88035
42	H	4.946393	5.245667	0.064846
43	H	6.161797	1.559252	2.266814
44	H	4.84243	2.19236	3.300899
45	H	5.373942	0.495826	3.464417
46	H	-1.35608	0.470507	-0.53618
47	H	-1.53576	0.138192	1.167947
48	H	-3.3987	-0.69718	-1.10631
49	H	-2.4431	-1.58522	2.477917
50	H	-4.09328	-0.96214	2.66824
51	H	-3.79861	-2.71107	2.646443
52	H	-5.34428	-3.17998	0.716144
53	H	-5.84865	-1.48302	0.809055
54	H	-5.29193	-2.16236	-0.7361
55	H	-5.11925	0.540285	0.248548
56	H	-3.86556	0.988501	1.402122
57	H	-2.71098	2.327035	-0.34183
58	H	-3.77766	1.674899	-1.58365

59	H	-5.36281	4.568179	1.178142
60	H	-5.02599	2.944335	1.800225
61	H	-3.68775	4.041341	1.391968
62	H	-4.3694	3.906823	-2.36531
63	H	-3.31491	4.668489	-1.152
64	H	-5.02295	5.122772	-1.24944
65	H	-6.62965	3.383576	-0.56
66	H	2.075822	-4.93755	-0.74289
**2R, 3R, 2"S-2** Conf. 2		Standard Orientation (Ångstroms)		
I	atom	X	Y	Z
1	C	-1.494367	-2.829801	-0.039043
2	C	-0.770728	-4.013864	-0.246694
3	C	0.599148	-3.948565	-0.452826
4	C	1.258841	-2.681244	-0.460111
5	C	0.490517	-1.510709	-0.229127
6	C	-0.886397	-1.55707	-0.027922
7	C	2.654183	-2.566742	-0.761175
8	C	3.212497	-1.155618	-0.930517
9	C	2.499444	-0.21447	0.053663
10	O	1.071146	-0.278818	-0.210612
11	O	3.433595	-3.52963	-0.91188
12	O	4.603505	-1.157729	-0.720957
13	C	2.919953	1.225971	-0.057687
14	C	3.733077	1.814063	0.92148
15	C	4.138322	3.147842	0.810178
16	C	3.732502	3.910929	-0.285533
17	C	2.915013	3.342779	-1.270469
18	C	2.521041	2.017052	-1.144783
19	O	4.091202	5.219836	-0.44393
20	O	4.092699	1.09173	2.037806
21	C	5.454303	0.645183	2.062541
22	C	-1.705843	-0.307135	0.174176
23	C	-3.206919	-0.572021	-0.037084
24	C	-3.629743	-1.882708	0.675128
25	O	-2.823526	-2.987616	0.146857
26	C	-3.431717	-1.85878	2.196828
27	C	-5.057193	-2.305308	0.322569
28	C	-4.065702	0.645613	0.357938
29	C	-3.788131	1.895502	-0.492122
30	C	-4.836092	3.024682	-0.337479
31	C	-4.931933	3.534197	1.103602
32	C	-4.501162	4.180601	-1.29189

33	O	-6.151206	2.52194	-0.640409
34	O	1.291679	-5.082117	-0.651734
35	H	-1.281784	-4.968911	-0.234602
36	H	2.969581	-0.825284	-1.957195
37	H	2.679696	-0.570856	1.073609
38	H	4.86616	-2.094336	-0.830784
39	H	4.749752	3.581367	1.598306
40	H	2.600407	3.950938	-2.112026
41	H	1.874218	1.581903	-1.901094
42	H	4.652672	5.486415	0.300575
43	H	5.648856	-0.033872	1.226014
44	H	6.151808	1.492084	2.021898
45	H	5.582266	0.120399	3.012124
46	H	-1.363202	0.466407	-0.520629
47	H	-1.535774	0.108296	1.178757
48	H	-3.364239	-0.762012	-1.109909
49	H	-2.391556	-1.654741	2.467942
50	H	-4.063828	-1.094978	2.660757
51	H	-3.70565	-2.831325	2.617568
52	H	-5.23697	-3.322926	0.68278
53	H	-5.793211	-1.643079	0.78627
54	H	-5.209289	-2.294744	-0.761844
55	H	-5.128835	0.406534	0.252192
56	H	-3.896926	0.878125	1.41705
57	H	-2.795526	2.30615	-0.26771
58	H	-3.763508	1.60596	-1.55501
59	H	-5.657992	4.35131	1.16336
60	H	-5.26582	2.740438	1.778114
61	H	-3.961372	3.904273	1.451577
62	H	-4.464332	3.830706	-2.332367
63	H	-3.527037	4.625574	-1.058807
64	H	-5.265545	4.961369	-1.221699
65	H	-6.154458	2.266981	-1.577528
66	H	2.241216	-4.831199	-0.770668
**2R, 3R, 2"S-2** Conf. 3		Standard Orientation (Ångstroms)		
I	atom	X	Y	Z
1	C	-1.6283	-2.82021	-0.03137
2	C	-0.94655	-4.0347	-0.1996
3	C	0.427316	-4.02433	-0.39005
4	C	1.133054	-2.78289	-0.42116
5	C	0.40599	-1.5799	-0.22712
6	C	-0.97463	-1.57117	-0.04054
7	C	2.534722	-2.72761	-0.71744

8	C	3.140556	-1.34303	-0.92695
9	C	2.459689	-0.35128	0.031209
10	O	1.02974	-0.36946	-0.23115
11	O	3.276676	-3.72355	-0.83615
12	O	4.52786	-1.37425	-0.72033
13	C	2.938372	1.067417	-0.12782
14	C	3.84521	1.627299	0.792985
15	C	4.316688	2.933436	0.618136
16	C	3.882124	3.689239	-0.47661
17	C	2.97807	3.1537	-1.39502
18	C	2.525366	1.851141	-1.20768
19	O	4.31532	4.96863	-0.68836
20	O	4.210506	0.834145	1.840698
21	C	5.149418	1.328427	2.780542
22	C	-1.74929	-0.28672	0.120203
23	C	-3.25617	-0.50007	-0.11069
24	C	-3.73569	-1.77805	0.626867
25	O	-2.96592	-2.92442	0.136756
26	C	-3.55539	-1.72136	2.150031
27	C	-5.17354	-2.16001	0.26783
28	C	-4.0824	0.753475	0.24644
29	C	-3.73509	1.982844	-0.60736
30	C	-4.5373	3.257663	-0.27463
31	C	-4.00825	4.432625	-1.11223
32	C	-6.04804	3.08358	-0.4999
33	O	-4.28175	3.511581	1.118718
34	O	1.080078	-5.18723	-0.5528
35	H	-1.49262	-4.96986	-0.17195
36	H	2.89769	-1.03325	-1.96116
37	H	2.625111	-0.69162	1.058803
38	H	4.768878	-2.31895	-0.79547
39	H	5.017316	3.3698	1.322948
40	H	2.648932	3.756985	-2.23398
41	H	1.815012	1.429955	-1.9128
42	H	4.941059	5.211801	0.011277
43	H	6.108267	1.565727	2.301905
44	H	4.771317	2.219013	3.300346
45	H	5.297535	0.525157	3.50434
46	H	-1.36423	0.454282	-0.58692
47	H	-1.57968	0.147022	1.116882
48	H	-3.40287	-0.71076	-1.1812
49	H	-2.50951	-1.56079	2.427863

50	H	-4.15285	-0.91376	2.584452
51	H	-3.88105	-2.66728	2.594269
52	H	-5.38504	-3.16835	0.636753
53	H	-5.89294	-1.47324	0.722055
54	H	-5.31906	-2.15545	-0.81757
55	H	-5.14629	0.523744	0.122099
56	H	-3.93652	1.012927	1.30084
57	H	-2.67563	2.232311	-0.47919
58	H	-3.8803	1.751345	-1.67177
59	H	-4.54195	5.358821	-0.86091
60	H	-2.94255	4.590111	-0.91665
61	H	-4.14321	4.254685	-2.18548
62	H	-6.45878	2.311357	0.156737
63	H	-6.27075	2.81317	-1.53877
64	H	-6.57593	4.021625	-0.28341
65	H	-4.75428	4.325182	1.356791
66	H	2.03926	-4.97226	-0.66879
**2R, 3R, 2"S-2** Conf. 4		Standard Orientation (Ångstroms)		
I	atom	X	Y	Z
1	C	-1.23135	-2.91993	0.050379
2	C	-0.48328	-4.10196	-0.06266
3	C	0.887938	-4.02482	-0.25657
4	C	1.524469	-2.74867	-0.33991
5	C	0.732892	-1.57933	-0.19973
6	C	-0.64731	-1.63916	-0.01877
7	C	2.920618	-2.62664	-0.63855
8	C	3.458471	-1.21862	-0.89009
9	C	2.723381	-0.2486	0.037467
10	O	1.293743	-0.34277	-0.26095
11	O	3.716292	-3.58312	-0.72992
12	O	4.846369	-1.18017	-0.68087
13	C	3.112829	1.200784	-0.06946
14	C	2.799417	2.073947	0.994589
15	C	3.126302	3.431403	0.92663
16	C	3.772473	3.931057	-0.21104
17	C	4.096283	3.085081	-1.27165
18	C	3.761668	1.736534	-1.18384
19	O	4.110977	5.24945	-0.32719
20	O	2.172839	1.507732	2.066873
21	C	1.901481	2.310061	3.204079
22	C	-1.49198	-0.39341	0.070242
23	C	-2.98257	-0.70538	-0.15024
24	C	-3.39391	-1.96333	0.657223

25	O	-2.56182	-3.08977	0.226983
26	C	-3.21808	-1.8168	2.175345
27	C	-4.80938	-2.43683	0.321062
28	C	-3.87456	0.522114	0.120512
29	C	-3.60417	1.696112	-0.83353
30	C	-4.67602	2.812876	-0.80411
31	C	-4.81578	3.447817	0.582542
32	C	-4.3403	3.885192	-1.8515
33	O	-5.97424	2.258594	-1.08855
34	O	1.603756	-5.15595	-0.37171
35	H	-0.97737	-5.06349	0.006983
36	H	3.200504	-0.96582	-1.93591
37	H	2.857659	-0.58702	1.071797
38	H	5.131057	-2.11437	-0.74217
39	H	2.884435	4.104805	1.742888
40	H	4.607454	3.488583	-2.13866
41	H	4.038873	1.080273	-2.0017
42	H	3.847463	5.718873	0.479339
43	H	2.821315	2.734543	3.627146
44	H	1.202647	3.123012	2.966401
45	H	1.441357	1.64404	3.93615
46	H	-1.14734	0.329538	-0.67615
47	H	-1.35157	0.102666	1.042439
48	H	-3.11138	-0.98671	-1.20663
49	H	-2.18273	-1.58534	2.44267
50	H	-3.86101	-1.02279	2.568778
51	H	-3.49208	-2.75467	2.66855
52	H	-5.56276	-1.75698	0.728182
53	H	-4.94938	-2.5072	-0.7628
54	H	-4.97522	-3.42931	0.751417
55	H	-4.93017	0.250755	0.016737
56	H	-3.73329	0.851275	1.158073
57	H	-2.62386	2.14556	-0.62999
58	H	-3.55158	1.311928	-1.86489
59	H	-5.5597	4.250336	0.552931
60	H	-5.14877	2.710025	1.318076
61	H	-3.86115	3.869129	0.916293
62	H	-5.12081	4.652852	-1.86909
63	H	-4.27163	3.443436	-2.85463
64	H	-3.38037	4.369543	-1.63865
65	H	-5.94999	1.917607	-1.99757
66	H	2.548801	-4.89408	-0.50384

**2R, 3R, 2"S-2** Conf. 5		Standard Orientation (Ångstroms)		
I	atom	X	Y	Z
1	C	-1.61215	-2.82286	-0.01976
2	C	-0.92873	-4.03414	-0.20085
3	C	0.443748	-4.01844	-0.40353
4	C	1.145853	-2.77495	-0.43303
5	C	0.417365	-1.57557	-0.22609
6	C	-0.96176	-1.57243	-0.02873
7	C	2.545529	-2.7135	-0.7397
8	C	3.145586	-1.32564	-0.94502
9	C	2.469169	-0.34202	0.024574
10	O	1.036733	-0.36302	-0.22647
11	O	3.28881	-3.70648	-0.87023
12	O	4.534187	-1.35324	-0.74779
13	C	2.940992	1.079589	-0.12812
14	C	3.847649	1.638883	0.79334
15	C	4.311471	2.948635	0.624884
16	C	3.869726	3.708565	-0.46418
17	C	2.965783	3.173482	-1.38324
18	C	2.52084	1.867367	-1.2024
19	O	4.294526	4.991434	-0.66955
20	O	4.219803	0.841812	1.835542
21	C	5.164564	1.332618	2.7717
22	C	-1.73749	-0.29085	0.144567
23	C	-3.24658	-0.50709	-0.06595
24	C	-3.71443	-1.79061	0.669457
25	O	-2.94855	-2.93061	0.160766
26	C	-5.1561	-2.17188	0.325514
27	C	-3.51456	-1.74546	2.190725
28	C	-4.06507	0.747914	0.30615
29	C	-3.7389	1.971076	-0.56729
30	C	-4.59151	3.240586	-0.33065
31	C	-6.06877	3.018547	-0.6664
32	C	-4.42983	3.787902	1.09598
33	O	-4.16165	4.241889	-1.27129
34	O	1.097916	-5.1783	-0.57858
35	H	-1.47151	-4.97121	-0.17384
36	H	2.894378	-1.01046	-1.97559
37	H	2.643521	-0.68865	1.048557
38	H	4.779202	-2.29597	-0.83323
39	H	5.011873	3.384557	1.330176
40	H	2.632482	3.779315	-2.21874
41	H	1.811845	1.446146	-1.90895

42	H	4.925914	5.232715	0.025688
43	H	4.788141	2.219011	3.299801
44	H	5.319832	0.525227	3.489349
45	H	6.119168	1.575164	2.287427
46	H	-1.36489	0.453068	-0.56639
47	H	-1.55485	0.140329	1.140544
48	H	-3.40936	-0.70924	-1.1352
49	H	-5.87125	-1.48821	0.791218
50	H	-5.31423	-2.16225	-0.75796
51	H	-5.36267	-3.18203	0.69207
52	H	-3.82772	-2.69762	2.630316
53	H	-2.4664	-1.57988	2.456964
54	H	-4.1136	-0.94745	2.641067
55	H	-5.13245	0.521161	0.208693
56	H	-3.89084	0.992021	1.362119
57	H	-2.68624	2.253381	-0.42178
58	H	-3.84332	1.704092	-1.62744
59	H	-6.60728	3.969354	-0.60121
60	H	-6.17523	2.637951	-1.68774
61	H	-6.53817	2.310264	0.023427
62	H	-4.96429	4.738627	1.190015
63	H	-3.37131	3.967843	1.327202
64	H	-4.82199	3.097263	1.850716
65	H	-3.2231	4.419824	-1.09664
66	H	2.055618	-4.96076	-0.70164
**2R, 3R, 2"S-2** Conf. 6		Standard Orientation (Ångstroms)		
I	atom	X	Y	Z
1	C	2.46531	-1.92567	0.210057
2	C	2.280849	-3.0396	-0.62085
3	C	1.001989	-3.3659	-1.0497
4	C	-0.11199	-2.57681	-0.63176
5	C	0.124315	-1.43797	0.180079
6	C	1.402357	-1.08901	0.613588
7	C	-1.45776	-2.94591	-0.96559
8	C	-2.57508	-2.13762	-0.31246
9	C	-2.12539	-0.67076	-0.20275
10	O	-0.89704	-0.63101	0.577124
11	O	-1.76842	-3.88141	-1.72925
12	O	-3.76348	-2.25009	-1.04822
13	C	-3.12799	0.2236	0.476465
14	C	-3.94555	1.092362	-0.27914
15	C	-4.89637	1.898462	0.349907

16	C	-5.03938	1.844847	1.740162
17	C	-4.23722	0.996693	2.505452
18	C	-3.29593	0.195663	1.85973
19	O	-5.98941	2.660427	2.289847
20	O	-3.73678	1.092898	-1.62533
21	C	-4.54679	1.924248	-2.44208
22	C	1.650274	0.152902	1.438193
23	C	3.136473	0.566224	1.38329
24	C	4.010821	-0.69718	1.62072
25	O	3.748332	-1.67383	0.566974
26	C	5.51606	-0.43833	1.518511
27	C	3.704215	-1.36189	2.97357
28	C	3.474537	1.31232	0.070688
29	C	2.853848	2.716126	-0.0184
30	C	3.132057	3.482753	-1.33623
31	C	2.345817	4.797177	-1.33799
32	C	4.62909	3.760521	-1.5498
33	O	2.611357	2.746966	-2.45652
34	O	0.824502	-4.44047	-1.83538
35	H	3.131615	-3.64199	-0.91573
36	H	-2.70351	-2.52774	0.715204
37	H	-1.90741	-0.29646	-1.20831
38	H	-3.63528	-3.03823	-1.61239
39	H	-5.53671	2.571813	-0.20535
40	H	-4.34499	0.960765	3.587267
41	H	-2.65976	-0.45745	2.449431
42	H	-5.9987	2.525323	3.250115
43	H	-4.21269	1.749872	-3.4662
44	H	-5.60751	1.657772	-2.35433
45	H	-4.41642	2.985206	-2.19225
46	H	1.340861	-0.00788	2.479229
47	H	1.009018	0.960409	1.073603
48	H	3.339365	1.249011	2.220033
49	H	5.807973	-0.13698	0.510359
50	H	5.816871	0.345957	2.221752
51	H	6.067626	-1.35091	1.764756
52	H	4.345811	-2.23823	3.107391
53	H	3.898701	-0.66387	3.79576
54	H	2.665641	-1.69382	3.043779
55	H	4.56076	1.40426	-0.02088
56	H	3.135678	0.704678	-0.77881
57	H	1.764748	2.650271	0.085562
58	H	3.215396	3.323617	0.824001

59	H	2.652077	5.439271	-0.50491
60	H	2.513523	5.336869	-2.27566
61	H	1.273063	4.596883	-1.24798
62	H	4.773028	4.328212	-2.47486
63	H	5.205397	2.831101	-1.63527
64	H	5.052247	4.338461	-0.71971
65	H	3.153948	1.950277	-2.56417
66	H	-0.14425	-4.50785	-2.02741
**2R, 3R, 2"S-2** Conf. 7		Standard Orientation (Ångstroms)		
I	atom	X	Y	Z
1	C	1.413648	-2.8638	0.021571
2	C	0.7019	-4.0555	0.225349
3	C	-0.68249	-4.01778	0.299223
4	C	-1.37027	-2.77158	0.173851
5	C	-0.61185	-1.59336	-0.05207
6	C	0.780534	-1.61256	-0.11849
7	C	-2.79169	-2.68396	0.340846
8	C	-3.40052	-1.28721	0.391669
9	C	-2.62068	-0.37235	-0.56546
10	O	-1.21165	-0.38343	-0.20575
11	O	-3.55289	-3.66458	0.47024
12	O	-4.75789	-1.32905	0.023329
13	C	-3.08779	1.057106	-0.62895
14	C	-3.08572	1.913792	0.493439
15	C	-3.54521	3.229714	0.382716
16	C	-4.0165	3.704971	-0.84788
17	C	-4.02708	2.876789	-1.9692
18	C	-3.56022	1.572035	-1.83816
19	O	-4.47501	4.982921	-0.99881
20	O	-2.61582	1.386414	1.661162
21	C	-2.6282	2.18825	2.831378
22	C	1.585736	-0.35088	-0.30593
23	C	3.064321	-0.56439	0.063534
24	C	3.582682	-1.89549	-0.5394
25	O	2.759504	-2.99362	-0.02771
26	C	3.523685	-1.95274	-2.07212
27	C	4.982826	-2.25822	-0.03953
28	C	3.932348	0.65589	-0.3008
29	C	3.540342	1.93606	0.453298
30	C	4.57737	3.08183	0.361036
31	C	4.832918	3.519213	-1.08415
32	C	4.109679	4.277171	1.205481

33	O	5.856806	2.629958	0.842449
34	O	-1.36402	-5.15855	0.4959
35	H	1.233829	-4.99496	0.314449
36	H	-3.27453	-0.91006	1.418748
37	H	-2.70812	-0.80754	-1.5709
38	H	-5.02148	-2.26096	0.157709
39	H	-3.54416	3.891987	1.242533
40	H	-4.39546	3.259284	-2.91454
41	H	-3.56847	0.921712	-2.70895
42	H	-4.4293	5.441241	-0.14555
43	H	-1.98774	3.072916	2.719259
44	H	-3.64602	2.506573	3.091755
45	H	-2.23227	1.556895	3.628627
46	H	1.15686	0.44507	0.311493
47	H	1.506959	0.009865	-1.34272
48	H	3.118602	-0.70194	1.154356
49	H	2.505851	-1.80297	-2.44396
50	H	4.168769	-1.18899	-2.51809
51	H	3.866701	-2.9335	-2.41621
52	H	5.215146	-3.29001	-0.32068
53	H	5.742703	-1.60507	-0.47696
54	H	5.039294	-2.18096	1.051445
55	H	4.983371	0.449195	-0.0743
56	H	3.870588	0.834481	-1.38178
57	H	2.570918	2.312835	0.103013
58	H	3.403863	1.694796	1.519847
59	H	5.545075	4.350415	-1.10261
60	H	5.259383	2.700332	-1.67043
61	H	3.903417	3.845612	-1.56332
62	H	4.862629	5.071824	1.184467
63	H	3.954857	3.980402	2.251657
64	H	3.162652	4.685714	0.83432
65	H	5.751768	2.413989	1.783431
66	H	-2.32658	-4.92664	0.509901
**2R, 3R, 2"S-2** Conf. 8		Standard Orientation (Ångstroms)		
I	atom	X	Y	Z
1	C	-1.41493	-2.86841	-0.02317
2	C	-0.70183	-4.05982	-0.22537
3	C	0.682514	-4.02128	-0.29638
4	C	1.36944	-2.77467	-0.16949
5	C	0.609659	-1.59696	0.055208
6	C	-0.78273	-1.61686	0.118712
7	C	2.790598	-2.68592	-0.33563

8	C	3.398027	-1.28854	-0.38754
9	C	2.617345	-0.3735	0.56865
10	O	1.208288	-0.3866	0.210723
11	O	3.553201	-3.66574	-0.46399
12	O	4.755419	-1.32897	-0.01868
13	C	3.082599	1.056851	0.628624
14	C	3.07299	1.912199	-0.49483
15	C	3.530013	3.229333	-0.38784
16	C	4.006601	3.706911	0.839773
17	C	4.024946	2.880029	1.961889
18	C	3.560224	1.574034	1.834713
19	O	4.462921	4.986301	0.98686
20	O	2.598711	1.382267	-1.65935
21	C	2.594749	2.185258	-2.82888
22	C	-1.58909	-0.35553	0.303374
23	C	-3.06651	-0.56933	-0.0708
24	C	-3.58645	-1.90041	0.530335
25	O	-2.76036	-2.99913	0.023215
26	C	-3.53321	-1.95794	2.063329
27	C	-4.98414	-2.26425	0.024569
28	C	-3.93664	0.650728	0.290749
29	C	-3.53716	1.926723	-0.46803
30	C	-4.54454	3.088542	-0.3584
31	C	-4.76806	3.534907	1.095096
32	C	-4.06586	4.27278	-1.21298
33	O	-5.77438	2.576303	-0.90355
34	O	1.365431	-5.16163	-0.49189
35	H	-1.23314	-4.99946	-0.31622
36	H	3.27217	-0.91244	-1.41503
37	H	2.706796	-0.80677	1.574793
38	H	5.019279	-2.26095	-0.15222
39	H	3.523229	3.890506	-1.24847
40	H	4.397451	3.264368	2.90488
41	H	3.57425	0.924702	2.70617
42	H	4.412346	5.442962	0.132981
43	H	1.951158	3.066588	-2.70897
44	H	3.60821	2.508772	-3.0998
45	H	2.193302	1.552718	-3.62237
46	H	-1.15811	0.439782	-0.31326
47	H	-1.51327	0.00553	1.340357
48	H	-3.11839	-0.70561	-1.1615
49	H	-3.8774	-2.93877	2.406133

50	H	-2.51681	-1.80788	2.43908
51	H	-4.18001	-1.19415	2.506823
52	H	-5.21596	-3.29692	0.303162
53	H	-5.74625	-1.61268	0.460442
54	H	-5.03733	-2.18353	-1.06612
55	H	-4.98519	0.442	0.058965
56	H	-3.87644	0.829311	1.372204
57	H	-2.55991	2.290741	-0.12786
58	H	-3.43284	1.687727	-1.53514
59	H	-5.47002	4.378487	1.132388
60	H	-5.18815	2.72266	1.695251
61	H	-3.83253	3.86465	1.561872
62	H	-4.79429	5.09367	-1.1801
63	H	-3.94832	3.964745	-2.25713
64	H	-3.10748	4.666507	-0.85466
65	H	-6.43929	3.279622	-0.83267
66	H	2.327698	-4.92869	-0.50478
**2R, 3R, 2"S-2** Conf. 9		Standard Orientation (Ångstroms)		
I	atom	X	Y	Z
1	C	2.433502	-1.96427	0.221175
2	C	2.225611	-3.08961	-0.58931
3	C	0.938965	-3.40587	-1.00096
4	C	-0.16071	-2.59291	-0.58976
5	C	0.09918	-1.44344	0.200291
6	C	1.384218	-1.10708	0.619998
7	C	-1.51366	-2.94914	-0.90476
8	C	-2.61425	-2.11155	-0.25954
9	C	-2.1403	-0.65001	-0.18345
10	O	-0.90855	-0.61276	0.58765
11	O	-1.84537	-3.8947	-1.64752
12	O	-3.80897	-2.22141	-0.98604
13	C	-3.12548	0.2745	0.481597
14	C	-3.96331	1.104289	-0.28875
15	C	-4.90493	1.932613	0.332289
16	C	-5.01362	1.937925	1.727717
17	C	-4.18661	1.128009	2.507341
18	C	-3.25992	0.307214	1.871463
19	O	-5.91848	2.729533	2.37846
20	O	-3.78683	1.053332	-1.64034
21	C	-4.64293	1.816489	-2.47355
22	C	1.660224	0.134078	1.434793
23	C	3.144497	0.542732	1.334819
24	C	4.023866	-0.7159	1.575421

25	O	3.721759	-1.72486	0.560111
26	C	5.522325	-0.45835	1.402252
27	C	3.768878	-1.34736	2.953765
28	C	3.477713	1.26007	0.007413
29	C	2.658742	2.536001	-0.24183
30	C	3.243219	3.468504	-1.33062
31	C	3.371094	2.768838	-2.68673
32	C	2.373617	4.72867	-1.45917
33	O	4.587312	3.852442	-0.98324
34	O	0.739442	-4.49208	-1.76534
35	H	3.065646	-3.70773	-0.88235
36	H	-2.74196	-2.47755	0.776802
37	H	-1.92304	-0.30285	-1.19933
38	H	-3.69562	-3.02506	-1.5315
39	H	-5.5536	2.573711	-0.25609
40	H	-4.28217	1.150393	3.587378
41	H	-2.60533	-0.31852	2.470394
42	H	-6.43453	3.226007	1.724694
43	H	-4.53165	2.893904	-2.29103
44	H	-4.3394	1.592314	-3.49761
45	H	-5.69368	1.530648	-2.33653
46	H	1.386969	-0.03	2.485669
47	H	1.009644	0.945128	1.096922
48	H	3.362606	1.240568	2.155634
49	H	5.85524	0.326395	2.090457
50	H	6.084545	-1.37097	1.623352
51	H	5.763949	-0.15402	0.381907
52	H	3.992545	-0.62942	3.751055
53	H	2.733682	-1.67778	3.070208
54	H	4.415823	-2.22016	3.085583
55	H	4.532539	1.550083	0.009701
56	H	3.345575	0.561745	-0.82753
57	H	1.624231	2.292199	-0.51416
58	H	2.596736	3.116609	0.693204
59	H	2.398688	2.395076	-3.02534
60	H	3.754738	3.470462	-3.43444
61	H	4.066723	1.927161	-2.62806
62	H	2.810534	5.415899	-2.19115
63	H	2.301037	5.253972	-0.49718
64	H	1.354162	4.482974	-1.77794
65	H	4.535165	4.37639	-0.16724
66	H	-0.23184	-4.55007	-1.94674

**2R, 3R, 2"S-2** Conf. 10		Standard Orientation (Ångstroms)		
I	atom	X	Y	Z
1	C	-1.18006	-2.97271	0.00451
2	C	-0.40602	-4.14035	-0.07376
3	C	0.969092	-4.03669	-0.22545
4	C	1.581498	-2.74862	-0.30295
5	C	0.762474	-1.59476	-0.20168
6	C	-0.62108	-1.68142	-0.05951
7	C	2.984698	-2.59961	-0.55394
8	C	3.502602	-1.18328	-0.80246
9	C	2.713957	-0.21533	0.082417
10	O	1.298395	-0.34841	-0.26466
11	O	3.802118	-3.54026	-0.60692
12	O	4.880809	-1.1146	-0.54221
13	C	3.069355	1.241652	-0.03875
14	C	2.637341	2.134615	0.966107
15	C	2.921156	3.500446	0.879733
16	C	3.645846	3.988416	-0.21496
17	C	4.090743	3.122531	-1.21369
18	C	3.794733	1.765869	-1.11058
19	O	3.94717	5.314279	-0.34643
20	O	1.944915	1.577262	2.001531
21	C	1.538379	2.405816	3.078557
22	C	-1.48949	-0.45024	-0.00339
23	C	-2.97021	-0.79078	-0.24856
24	C	-3.37121	-2.05547	0.555988
25	O	-2.51307	-3.16609	0.142156
26	C	-3.22152	-1.90139	2.076543
27	C	-4.77201	-2.55862	0.199927
28	C	-3.89369	0.41906	0.006504
29	C	-3.60212	1.620437	-0.90707
30	C	-4.55468	2.830692	-0.73494
31	C	-4.01519	4.023662	-1.52915
32	C	-5.99358	2.511805	-1.17077
33	O	-4.54963	3.270968	0.636189
34	O	1.71104	-5.15379	-0.30514
35	H	-0.88234	-5.11113	-0.00915
36	H	3.279102	-0.94806	-1.8603
37	H	2.818148	-0.53333	1.127043
38	H	5.186622	-2.04319	-0.58281
39	H	2.585777	4.189501	1.648319
40	H	4.661339	3.517742	-2.04679
41	H	4.164923	1.095417	-1.87849

42	H	3.589649	5.799628	0.413126
43	H	0.828984	3.176175	2.748946
44	H	1.044191	1.745044	3.792979
45	H	2.397726	2.886687	3.564045
46	H	-1.14161	0.267131	-0.75292
47	H	-1.37472	0.061831	0.963743
48	H	-3.07758	-1.07498	-1.30619
49	H	-2.19498	-1.64938	2.358214
50	H	-3.88455	-1.11892	2.459766
51	H	-3.48483	-2.84325	2.567909
52	H	-5.54651	-1.90065	0.604178
53	H	-4.89868	-2.62537	-0.88578
54	H	-4.91937	-3.55725	0.622486
55	H	-4.93495	0.104257	-0.13042
56	H	-3.7903	0.738119	1.052598
57	H	-2.5854	1.982116	-0.71654
58	H	-3.63121	1.296025	-1.9571
59	H	-3.93908	3.782839	-2.59504
60	H	-4.67636	4.888343	-1.41194
61	H	-3.02117	4.302156	-1.16414
62	H	-6.42914	1.700024	-0.57469
63	H	-6.03384	2.207134	-2.22285
64	H	-6.62601	3.396283	-1.04222
65	H	-5.03829	2.613525	1.155306
66	H	2.654342	-4.87377	-0.40945
**2R, 3R, 2"S-2** Conf. 11		Standard Orientation (Ångstroms)		
I	atom	X	Y	Z
1	C	2.389275	-1.98474	0.277416
2	C	2.15796	-3.12577	-0.50485
3	C	0.864899	-3.42847	-0.90494
4	C	-0.21826	-2.5848	-0.51069
5	C	0.065269	-1.42183	0.252004
6	C	1.356688	-1.10006	0.661384
7	C	-1.57637	-2.91783	-0.81975
8	C	-2.66196	-2.03782	-0.20387
9	C	-2.15544	-0.58732	-0.15789
10	O	-0.92742	-0.5647	0.620758
11	O	-1.93112	-3.87377	-1.53912
12	O	-3.85022	-2.14429	-0.94864
13	C	-3.11464	0.383525	0.475185
14	C	-3.82976	1.300092	-0.30875
15	C	-4.73343	2.192421	0.276582

16	C	-4.93221	2.177477	1.657892
17	C	-4.2224	1.273365	2.45793
18	C	-3.32803	0.394508	1.861235
19	O	-5.79624	3.029155	2.286286
20	O	-3.60126	1.366441	-1.6659
21	C	-4.63487	0.806207	-2.48577
22	C	1.658781	0.155028	1.445061
23	C	3.147971	0.538553	1.319957
24	C	4.01164	-0.72574	1.584478
25	O	3.682572	-1.7595	0.602254
26	C	3.764452	-1.31521	2.982885
27	C	5.511595	-0.49635	1.386772
28	C	3.472707	1.217646	-0.02961
29	C	2.69334	2.517202	-0.28272
30	C	3.265166	3.393516	-1.42396
31	C	3.293254	2.653639	-2.7645
32	C	2.448033	4.688679	-1.54555
33	O	4.641013	3.723021	-1.15278
34	O	0.643052	-4.52854	-1.64269
35	H	2.986113	-3.7647	-0.78675
36	H	-2.81696	-2.37932	0.836014
37	H	-1.92703	-0.26321	-1.17905
38	H	-3.74407	-2.97057	-1.46282
39	H	-5.2555	2.906176	-0.35656
40	H	-4.38054	1.280065	3.531225
41	H	-2.76486	-0.29409	2.484209
42	H	-6.21866	3.597705	1.62379
43	H	-4.74667	-0.26361	-2.28155
44	H	-5.593	1.316588	-2.32196
45	H	-4.31755	0.961964	-3.5195
46	H	1.393293	0.019277	2.501872
47	H	1.017185	0.96752	1.093937
48	H	3.385421	1.253157	2.120683
49	H	2.725901	-1.62608	3.121597
50	H	4.399744	-2.19391	3.130971
51	H	4.009245	-0.57906	3.757052
52	H	5.863301	0.306059	2.044556
53	H	6.063017	-1.40916	1.632835
54	H	5.746339	-0.23033	0.35427
55	H	4.536537	1.469885	-0.06275
56	H	3.292993	0.508165	-0.84607
57	H	1.639145	2.308308	-0.50338
58	H	2.695613	3.125878	0.636402

59	H	2.289227	2.316398	-3.04405
60	H	3.669249	3.317336	-3.54977
61	H	3.95302	1.782774	-2.7171
62	H	2.877434	5.33471	-2.31834
63	H	2.449284	5.242404	-0.59685
64	H	1.403586	4.482095	-1.80549
65	H	4.653014	4.272145	-0.35188
66	H	-0.32878	-4.57276	-1.8225
**2R, 3R, 2"R-2** Conf. 1		Standard Orientation (Ångstroms)		
I	atom	X	Y	Z
1	C	-1.66901	-2.78187	0.055883
2	C	-1.00607	-3.99437	0.295559
3	C	0.380879	-4.02298	0.303264
4	C	1.119879	-2.82643	0.05451
5	C	0.408645	-1.61535	-0.14741
6	C	-0.98332	-1.56636	-0.1517
7	C	2.549725	-2.83821	-0.05101
8	C	3.217721	-1.5401	-0.4956
9	C	2.442866	-0.35463	0.104852
10	O	1.063054	-0.43776	-0.34686
11	O	3.271899	-3.82923	0.177932
12	O	4.563753	-1.51711	-0.10164
13	C	2.980967	0.990192	-0.30623
14	C	3.787036	1.740202	0.572175
15	C	4.319839	2.9699	0.168878
16	C	4.047549	3.459186	-1.11386
17	C	3.243175	2.734148	-1.99426
18	C	2.72769	1.510464	-1.57748
19	O	4.54626	4.65441	-1.55156
20	O	3.992915	1.204938	1.809711
21	C	4.83222	1.890701	2.723381
22	C	-1.73653	-0.2687	-0.3047
23	C	-3.19829	-0.40485	0.154597
24	C	-3.81475	-1.71307	-0.40415
25	O	-3.01934	-2.84531	0.078407
26	C	-5.20178	-1.99984	0.174382
27	C	-3.84115	-1.78844	-1.9371
28	C	-4.02338	0.856054	-0.16923
29	C	-3.52816	2.11454	0.561158
30	C	-4.50868	3.311875	0.52085
31	C	-3.94483	4.475155	1.350808
32	C	-4.80425	3.772233	-0.90938

33	O	-5.78739	2.920752	1.055482
34	O	1.015309	-5.18691	0.520031
35	H	-1.5772	-4.89926	0.464321
36	H	3.122614	-1.48129	-1.59641
37	H	2.455651	-0.44868	1.195856
38	H	4.783303	-2.45082	0.088691
39	H	4.943514	3.551285	0.840468
40	H	3.038629	3.132698	-2.98191
41	H	2.093618	0.94367	-2.25252
42	H	5.095609	5.044026	-0.85399
43	H	5.849127	2.003546	2.326068
44	H	4.42934	2.879965	2.979332
45	H	4.863336	1.271671	3.621647
46	H	-1.22912	0.509786	0.274305
47	H	-1.70682	0.079175	-1.34831
48	H	-3.19497	-0.53254	1.247982
49	H	-5.94883	-1.30923	-0.22601
50	H	-5.50346	-3.01928	-0.08539
51	H	-5.19384	-1.91394	1.266128
52	H	-4.46837	-0.99566	-2.35718
53	H	-2.83865	-1.69599	-2.3652
54	H	-4.25389	-2.7527	-2.24976
55	H	-5.06991	0.702834	0.113292
56	H	-4.01027	1.029908	-1.25286
57	H	-2.55973	2.441666	0.161908
58	H	-3.35018	1.864559	1.619398
59	H	-2.99261	4.835086	0.944454
60	H	-4.65442	5.30894	1.3636
61	H	-3.76444	4.163647	2.388519
62	H	-3.88113	4.05683	-1.42606
63	H	-5.47502	4.637184	-0.89257
64	H	-5.295	2.979659	-1.48145
65	H	-5.65388	2.700531	1.99188
66	H	1.987299	-5.00431	0.480675
**2R, 3R, 2"R-2** Conf. 2		Standard Orientation (Ångstroms)		
I	atom	X	Y	Z
1	C	1.431215	-2.87481	-0.07261
2	C	0.718196	-4.06784	-0.26463
3	C	-0.66225	-4.02304	-0.39001
4	C	-1.34568	-2.77063	-0.31243
5	C	-0.58119	-1.58508	-0.15388
6	C	0.806872	-1.61228	-0.02664
7	C	-2.77698	-2.69785	-0.33872

8	C	-3.40912	-1.33678	-0.06752
9	C	-2.5124	-0.24733	-0.67629
10	O	-1.17123	-0.3615	-0.12663
11	O	-3.53342	-3.66835	-0.54928
12	O	-4.69708	-1.27449	-0.63105
13	C	-2.98503	1.169083	-0.48707
14	C	-3.13736	1.762832	0.788968
15	C	-3.58472	3.078454	0.912395
16	C	-3.89406	3.819642	-0.23392
17	C	-3.75545	3.25558	-1.50137
18	C	-3.29979	1.941071	-1.60362
19	O	-4.32789	5.100115	-0.03446
20	O	-2.82526	0.980933	1.861083
21	C	-2.96787	1.52203	3.166293
22	C	1.619123	-0.34725	0.090271
23	C	3.105992	-0.59354	-0.22305
24	C	3.593277	-1.88741	0.481643
25	O	2.773498	-3.01102	0.023422
26	C	5.001682	-2.29927	0.046661
27	C	3.493394	-1.83693	2.012368
28	C	3.97793	0.638801	0.097118
29	C	3.625157	1.875545	-0.74357
30	C	4.452954	3.138383	-0.42886
31	C	5.95645	2.94079	-0.68086
32	C	3.926683	4.319749	-1.25924
33	O	4.226475	3.399392	0.968376
34	O	-1.34704	-5.16644	-0.55985
35	H	1.245736	-5.01306	-0.30631
36	H	-3.44083	-1.19598	1.024311
37	H	-2.44772	-0.448	-1.75506
38	H	-4.95995	-2.20905	-0.7477
39	H	-3.70817	3.555204	1.876293
40	H	-3.99578	3.82912	-2.39359
41	H	-3.19032	1.497866	-2.58994
42	H	-4.52147	5.501847	-0.8957
43	H	-2.66929	0.726511	3.851055
44	H	-2.31477	2.391622	3.31216
45	H	-4.007	1.809225	3.371366
46	H	1.206945	0.402028	-0.59275
47	H	1.517401	0.089501	1.094933
48	H	3.191395	-0.80118	-1.30078
49	H	5.208179	-3.31511	0.397351

50	H	5.091451	-2.28934	-1.04474
51	H	5.759293	-1.63283	0.467945
52	H	2.466982	-1.65581	2.344539
53	H	3.820721	-2.79258	2.434099
54	H	4.130089	-1.04528	2.419756
55	H	5.030105	0.384	-0.07078
56	H	3.881813	0.90182	1.156459
57	H	2.572987	2.139251	-0.58724
58	H	3.738968	1.644924	-1.81199
59	H	6.156013	2.663145	-1.72255
60	H	6.50236	3.871563	-0.47799
61	H	6.367254	2.164738	-0.02884
62	H	4.47705	5.238731	-1.01761
63	H	2.866523	4.491973	-1.04663
64	H	4.041841	4.138774	-2.3343
65	H	4.721079	4.202384	1.197533
66	H	-2.30558	-4.92678	-0.62487
**2R, 3R, 2"R-2** Conf. 3		Standard Orientation (Ångstroms)		
I	atom	X	Y	Z
1	C	1.410462	-2.8519	-0.03638
2	C	0.704641	-4.03995	-0.27836
3	C	-0.67172	-3.99295	-0.44348
4	C	-1.35931	-2.74372	-0.35345
5	C	-0.602	-1.56232	-0.14036
6	C	0.781907	-1.5915	0.024453
7	C	-2.78942	-2.67117	-0.42458
8	C	-3.43198	-1.31793	-0.13913
9	C	-2.51702	-0.21288	-0.68997
10	O	-1.1957	-0.34054	-0.09482
11	O	-3.53695	-3.63635	-0.68444
12	O	-4.70169	-1.24127	-0.74026
13	C	-2.99664	1.199232	-0.48618
14	C	-3.2115	1.757643	0.792799
15	C	-3.66508	3.073677	0.925638
16	C	-3.91463	3.84629	-0.21582
17	C	-3.7104	3.314909	-1.48838
18	C	-3.25312	2.004713	-1.59791
19	O	-4.35881	5.135337	-0.13073
20	O	-2.95158	0.950068	1.861247
21	C	-3.17704	1.44769	3.170486
22	C	1.590361	-0.32995	0.192009
23	C	3.080718	-0.56993	-0.10504
24	C	3.561485	-1.87418	0.582906

25	O	2.749814	-2.99036	0.092297
26	C	4.977373	-2.27095	0.159676
27	C	3.439938	-1.8547	2.112723
28	C	3.943051	0.661106	0.236524
29	C	3.585982	1.906997	-0.58921
30	C	4.624708	3.05233	-0.51217
31	C	4.195129	4.207804	-1.42889
32	C	4.828009	3.556033	0.919718
33	O	5.919225	2.573553	-0.92245
34	O	-1.34877	-5.13198	-0.66422
35	H	1.234519	-4.98336	-0.33018
36	H	-3.49787	-1.20665	0.954564
37	H	-2.41282	-0.38806	-1.76995
38	H	-4.95916	-2.17217	-0.89264
39	H	-3.82966	3.506905	1.906958
40	H	-3.90952	3.925278	-2.36218
41	H	-3.09374	1.58533	-2.58789
42	H	-4.47459	5.372248	0.802386
43	H	-2.53946	2.315048	3.385926
44	H	-4.22904	1.72215	3.322459
45	H	-2.9154	0.63263	3.847348
46	H	1.191338	0.441284	-0.4748
47	H	1.473195	0.075459	1.20855
48	H	3.178737	-0.75969	-1.18495
49	H	5.724167	-1.59998	0.592698
50	H	5.189958	-3.28789	0.503476
51	H	5.077525	-2.25089	-0.93063
52	H	4.075928	-1.07594	2.545682
53	H	2.409814	-1.67632	2.435073
54	H	3.757354	-2.82044	2.518527
55	H	5.000403	0.43972	0.058479
56	H	3.84255	0.888528	1.305537
57	H	2.606387	2.302646	-0.29242
58	H	3.486974	1.617251	-1.64764
59	H	3.236402	4.635658	-1.11419
60	H	4.950445	5.000385	-1.41621
61	H	4.078644	3.862529	-2.46496
62	H	3.882525	3.907063	1.347541
63	H	5.542757	4.385196	0.926577
64	H	5.228326	2.764017	1.55911
65	H	5.850691	2.32007	-1.85745
66	H	-2.30525	-4.89198	-0.75359

**2R, 3R, 2"R-2** Conf. 4		Standard Orientation (Ångstroms)		
I	atom	X	Y	Z
1	C	1.100609	-3.02987	-0.08159
2	C	0.302453	-4.1523	-0.35102
3	C	-1.07768	-4.01604	-0.38009
4	C	-1.67216	-2.74285	-0.12467
5	C	-0.82591	-1.62892	0.113846
6	C	0.561588	-1.74635	0.140403
7	C	-3.09354	-2.57739	-0.05858
8	C	-3.61938	-1.2044	0.361147
9	C	-2.6816	-0.13475	-0.20392
10	O	-1.34652	-0.39329	0.334093
11	O	-3.92721	-3.47235	-0.30553
12	O	-4.93838	-1.03108	-0.09001
13	C	-3.02571	1.294928	0.116898
14	C	-2.38254	2.327916	-0.5988
15	C	-2.65799	3.669809	-0.32229
16	C	-3.58597	3.993771	0.67564
17	C	-4.23948	2.989261	1.38857
18	C	-3.94881	1.658366	1.099408
19	O	-3.889	5.289702	0.98482
20	O	-1.49723	1.927081	-1.55852
21	C	-0.881	2.909579	-2.37433
22	C	1.456034	-0.54698	0.327678
23	C	2.901672	-0.84383	-0.11149
24	C	3.348201	-2.22884	0.426783
25	O	2.434777	-3.25037	-0.09086
26	C	4.700595	-2.67105	-0.13728
27	C	3.340192	-2.33478	1.95807
28	C	3.86763	0.298263	0.266719
29	C	3.559126	1.621978	-0.44959
30	C	4.474869	2.803155	-0.06858
31	C	5.951588	2.541775	-0.4053
32	C	3.988434	4.08018	-0.77266
33	O	4.320444	2.956399	1.353582
34	O	-1.84315	-5.09258	-0.62652
35	H	0.765409	-5.11575	-0.52704
36	H	-3.56207	-1.16596	1.465324
37	H	-2.61983	-0.26259	-1.29205
38	H	-5.25915	-1.93838	-0.2682
39	H	-2.16094	4.465349	-0.86835
40	H	-4.96412	3.259788	2.148528
41	H	-4.48161	0.882721	1.638136

42	H	-3.37693	5.882025	0.412858
43	H	-0.26243	2.360547	-3.0865
44	H	-1.62427	3.503957	-2.92179
45	H	-0.24269	3.581007	-1.78469
46	H	1.044294	0.289856	-0.24559
47	H	1.44799	-0.21375	1.376008
48	H	2.90601	-0.94557	-1.20764
49	H	4.863246	-3.72831	0.093605
50	H	4.728258	-2.55098	-1.22552
51	H	5.521389	-2.09651	0.300827
52	H	2.347604	-2.13535	2.371961
53	H	3.63341	-3.34584	2.25788
54	H	4.045783	-1.62635	2.40375
55	H	4.892323	-0.00466	0.025375
56	H	3.836481	0.474077	1.347732
57	H	2.531765	1.930419	-0.22385
58	H	3.615853	1.477826	-1.53771
59	H	6.090871	2.343458	-1.47449
60	H	6.561741	3.418528	-0.15105
61	H	6.340134	1.690193	0.160185
62	H	4.601724	4.942392	-0.47964
63	H	2.950192	4.292833	-0.49647
64	H	4.048505	3.987176	-1.86347
65	H	4.890376	3.691069	1.631712
66	H	-2.78646	-4.79517	-0.59826
**2R, 3R, 2"R-2** Conf. 5		Standard Orientation (Ångstroms)		
I	atom	X	Y	Z
1	C	1.306734	-2.89552	-0.0644
2	C	0.565192	-4.06718	-0.27057
3	C	-0.81503	-3.98788	-0.39082
4	C	-1.46836	-2.72146	-0.29161
5	C	-0.67612	-1.5582	-0.1142
6	C	0.711256	-1.6193	0.006146
7	C	-2.89913	-2.61397	-0.32656
8	C	-3.50276	-1.24046	-0.05351
9	C	-2.57063	-0.16617	-0.63346
10	O	-1.24168	-0.32107	-0.05779
11	O	-3.67351	-3.56594	-0.55092
12	O	-4.77638	-1.13972	-0.64321
13	C	-3.01442	1.25647	-0.42287
14	C	-3.14061	1.826783	0.854691
15	C	-3.55533	3.151191	1.011999

16	C	-3.85613	3.928142	-0.1097
17	C	-3.74314	3.379176	-1.39083
18	C	-3.32786	2.060009	-1.52704
19	O	-4.27216	5.225087	-0.01305
20	O	-2.902	1.049413	1.967129
21	C	-1.76332	1.412803	2.746461
22	C	1.555126	-0.37658	0.135951
23	C	3.03152	-0.65461	-0.19828
24	C	3.49739	-1.96828	0.483774
25	O	2.646862	-3.06432	0.018468
26	C	4.8914	-2.40404	0.026644
27	C	3.415496	-1.93969	2.016255
28	C	3.933826	0.556595	0.119251
29	C	3.584093	1.811579	-0.69791
30	C	4.527024	3.02803	-0.53775
31	C	4.565184	3.538109	0.905189
32	C	5.945044	2.740289	-1.05521
33	O	3.967805	4.12195	-1.288
34	O	-1.52481	-5.11193	-0.57796
35	H	1.06955	-5.02397	-0.33106
36	H	-3.55167	-1.10553	1.038717
37	H	-2.48748	-0.35133	-1.71335
38	H	-5.07025	-2.06458	-0.75976
39	H	-3.66716	3.557518	2.014665
40	H	-3.98333	3.990947	-2.25371
41	H	-3.24239	1.634794	-2.52364
42	H	-4.32213	5.474359	0.922825
43	H	-1.84114	2.439335	3.126859
44	H	-1.7384	0.720013	3.59054
45	H	-0.8441	1.311202	2.157832
46	H	1.156284	0.396447	-0.52832
47	H	1.478855	0.042751	1.15134
48	H	3.098641	-0.84956	-1.27929
49	H	5.668359	-1.75955	0.447193
50	H	5.080476	-3.42842	0.361664
51	H	4.968512	-2.38199	-1.06547
52	H	2.398238	-1.73637	2.36365
53	H	3.719972	-2.91122	2.417965
54	H	4.080037	-1.17488	2.431046
55	H	4.977822	0.285557	-0.07369
56	H	3.863499	0.788493	1.189775
57	H	2.576795	2.162193	-0.44563
58	H	3.560315	1.541685	-1.76613

59	H	3.551325	3.745373	1.26404
60	H	5.03371	2.811155	1.576126
61	H	5.138135	4.46968	0.951713
62	H	6.545053	3.655003	-1.01562
63	H	5.917356	2.396399	-2.09791
64	H	6.455183	1.971135	-0.46504
65	H	3.916484	3.840957	-2.21609
66	H	-2.47827	-4.85045	-0.63851
**2R, 3R, 2"R-2** Conf. 6		Standard Orientation (Ångstroms)		
I	atom	X	Y	Z
1	C	1.418323	-2.87309	-0.05457
2	C	0.705702	-4.06238	-0.26692
3	C	-0.67343	-4.0132	-0.40827
4	C	-1.35515	-2.7604	-0.32546
5	C	-0.59093	-1.57827	-0.14475
6	C	0.795797	-1.6101	-0.00201
7	C	-2.7865	-2.6841	-0.37001
8	C	-3.41884	-1.32413	-0.09388
9	C	-2.51163	-0.23181	-0.68196
10	O	-1.17909	-0.35427	-0.10977
11	O	-3.54122	-3.651	-0.59998
12	O	-4.70027	-1.25308	-0.66988
13	C	-2.98256	1.1851	-0.49452
14	C	-3.16645	1.768819	0.777901
15	C	-3.6117	3.089044	0.895376
16	C	-3.88399	3.840562	-0.25494
17	C	-3.71175	3.283457	-1.52129
18	C	-3.26174	1.969779	-1.61572
19	O	-4.32131	5.132552	-0.18518
20	O	-2.88626	0.980382	1.855754
21	C	-3.07523	1.506424	3.159637
22	C	1.608904	-0.34801	0.13647
23	C	3.097714	-0.59559	-0.16408
24	C	3.576633	-1.89688	0.532854
25	O	2.760245	-3.01284	0.054097
26	C	4.989249	-2.30493	0.107928
27	C	3.461092	-1.8635	2.063162
28	C	3.966338	0.635743	0.170035
29	C	3.61072	1.879446	-0.66168
30	C	4.515014	3.120835	-0.47348
31	C	4.486646	3.639332	0.966765
32	C	5.958263	2.86764	-0.93658

33	O	3.956435	4.196534	-1.25039
34	O	-1.35781	-5.15318	-0.59852
35	H	1.231686	-5.00824	-0.31301
36	H	-3.46157	-1.19384	0.99895
37	H	-2.42815	-0.42656	-1.76035
38	H	-4.96394	-2.18526	-0.8021
39	H	-3.75195	3.542092	1.871484
40	H	-3.92744	3.878075	-2.40196
41	H	-3.1259	1.53082	-2.60072
42	H	-4.40342	5.392457	0.745379
43	H	-4.12137	1.790863	3.332455
44	H	-2.80145	0.703844	3.846568
45	H	-2.42724	2.373912	3.340534
46	H	1.206773	0.410744	-0.54217
47	H	1.497872	0.077184	1.145553
48	H	3.193742	-0.79245	-1.24262
49	H	5.743376	-1.64262	0.54211
50	H	5.192445	-3.32377	0.451556
51	H	5.089353	-2.28568	-0.98239
52	H	2.432806	-1.67868	2.387437
53	H	3.776648	-2.82688	2.475952
54	H	4.10105	-1.08339	2.487961
55	H	5.020499	0.386382	0.004797
56	H	3.863396	0.870547	1.237209
57	H	2.586163	2.202479	-0.44516
58	H	3.631411	1.606912	-1.72945
59	H	3.455273	3.823416	1.286035
60	H	4.947069	2.92758	1.659224
61	H	5.034896	4.584784	1.028802
62	H	6.531375	3.798688	-0.88227
63	H	5.977595	2.515638	-1.9767
64	H	6.46649	2.116671	-0.32181
65	H	3.938756	3.906078	-2.17681
66	H	-2.31512	-4.91143	-0.67357
**2R, 3R, 2"R-2** Conf. 7		Standard Orientation (Ångstroms)		
I	atom	X	Y	Z
1	C	1.466212	2.704073	0.011926
2	C	0.79547	3.903976	0.289097
3	C	-0.59127	3.915369	0.332414
4	C	-1.32206	2.714213	0.0825
5	C	-0.60231	1.51525	-0.15787
6	C	0.789698	1.483449	-0.19917
7	C	-2.75443	2.710322	0.012814

8	C	-3.418	1.411033	-0.43487
9	C	-2.61362	0.226905	0.128559
10	O	-1.24774	0.33348	-0.35914
11	O	-3.48177	3.689067	0.275396
12	O	-4.75353	1.365976	-0.00846
13	C	-3.14695	-1.119	-0.28482
14	C	-3.92723	-1.88461	0.603329
15	C	-4.45646	-3.11582	0.200311
16	C	-4.20604	-3.59122	-1.09211
17	C	-3.42724	-2.85057	-1.98242
18	C	-2.91502	-1.62554	-1.56567
19	O	-4.70235	-4.78727	-1.5301
20	O	-4.11244	-1.36207	1.849732
21	C	-4.92562	-2.06424	2.774319
22	C	1.556345	0.199501	-0.3943
23	C	3.025552	0.349161	0.03661
24	C	3.604024	1.669508	-0.53434
25	O	2.817032	2.783719	-0.00029
26	C	5.014181	1.964517	-0.02324
27	C	3.553638	1.768275	-2.06628
28	C	3.836389	-0.9273	-0.30404
29	C	4.804826	-1.38421	0.798414
30	C	5.436078	-2.77977	0.57515
31	C	6.257139	-2.84977	-0.7161
32	C	6.310193	-3.15351	1.78246
33	O	4.403321	-3.76839	0.409381
34	O	-1.23356	5.067864	0.584274
35	H	1.359676	4.813224	0.457758
36	H	-3.3493	1.369147	-1.53841
37	H	-2.59747	0.306767	1.220696
38	H	-4.97946	2.293935	0.201318
39	H	-5.06035	-3.70918	0.879484
40	H	-3.23811	-3.23913	-2.97706
41	H	-2.29984	-1.0473	-2.24836
42	H	-5.22899	-5.19078	-0.82299
43	H	-4.50713	-3.05112	3.013973
44	H	-4.94529	-1.45285	3.67816
45	H	-5.94909	-2.18501	2.396647
46	H	1.084069	-0.6026	0.184535
47	H	1.506308	-0.13206	-1.44189
48	H	3.032553	0.482439	1.12832
49	H	5.73594	1.256658	-0.44168

50	H	5.312702	2.974742	-0.32088
51	H	5.054296	1.904303	1.069023
52	H	2.52946	1.695419	-2.44236
53	H	3.961219	2.733097	-2.38374
54	H	4.147212	0.976971	-2.53477
55	H	4.380254	-0.78931	-1.24611
56	H	3.136432	-1.75088	-0.48641
57	H	4.257142	-1.41562	1.753748
58	H	5.60969	-0.65197	0.9407
59	H	7.050243	-2.09385	-0.71615
60	H	6.718805	-3.83768	-0.81215
61	H	5.622011	-2.69218	-1.59239
62	H	6.714734	-4.16313	1.656725
63	H	5.724513	-3.13278	2.711308
64	H	7.147221	-2.45635	1.904543
65	H	3.901829	-3.80609	1.240169
66	H	-2.20408	4.874129	0.565772

**2R, 3R, 2"R-2** Conf. 8		Standard Orientation (Ångstroms)		
I	atom	X	Y	Z
1	C	1.889331	2.500378	-0.04095
2	C	1.365129	3.730249	0.383357
3	C	-0.0085	3.879022	0.508586
4	C	-0.87509	2.787258	0.197617
5	C	-0.30009	1.550566	-0.19452
6	C	1.076083	1.383713	-0.32733
7	C	-2.30126	2.933303	0.220949
8	C	-3.12327	1.752688	-0.28946
9	C	-2.41429	0.44988	0.11767
10	O	-1.07918	0.464849	-0.45954
11	O	-2.90575	3.951384	0.613851
12	O	-4.42862	1.803035	0.221363
13	C	-3.10898	-0.7979	-0.35828
14	C	-3.88852	-1.56609	0.528568
15	C	-4.56444	-2.70412	0.074287
16	C	-4.46204	-3.08355	-1.26903
17	C	-3.68705	-2.33976	-2.16011
18	C	-3.02766	-1.20741	-1.69112
19	O	-5.10352	-4.18716	-1.75849
20	O	-3.9243	-1.14159	1.824325
21	C	-4.72741	-1.84559	2.756431
22	C	1.690155	0.06016	-0.70551
23	C	3.179735	-0.00483	-0.32113
24	C	3.897739	1.299369	-0.75632

25	O	3.237302	2.440671	-0.11591
26	C	3.875104	1.545938	-2.27074
27	C	5.326429	1.394314	-0.21573
28	C	3.843952	-1.28619	-0.87129
29	C	3.38177	-2.60324	-0.20485
30	C	4.33556	-3.17905	0.871859
31	C	4.542623	-2.23253	2.05729
32	C	3.803215	-4.53479	1.361348
33	O	5.651033	-3.35648	0.313979
34	O	-0.51058	5.061403	0.901139
35	H	2.031481	4.55527	0.604286
36	H	-3.12408	1.804018	-1.39473
37	H	-2.31861	0.433547	1.208414
38	H	-4.54184	2.726042	0.523871
39	H	-5.16878	-3.29839	0.752257
40	H	-3.61582	-2.65353	-3.19576
41	H	-2.4147	-0.6283	-2.37516
42	H	-5.61232	-4.60179	-1.04475
43	H	-4.39222	-2.88462	2.876854
44	H	-4.61317	-1.3188	3.705387
45	H	-5.78479	-1.83586	2.462257
46	H	1.133445	-0.7462	-0.21671
47	H	1.578622	-0.12378	-1.78471
48	H	3.240774	-0.01898	0.775699
49	H	4.41213	0.754311	-2.80328
50	H	2.85328	1.589273	-2.65917
51	H	4.361373	2.500462	-2.49524
52	H	5.990506	0.683135	-0.71385
53	H	5.714829	2.403	-0.38715
54	H	5.348191	1.199586	0.861494
55	H	4.933459	-1.23408	-0.77796
56	H	3.633613	-1.34206	-1.94698
57	H	3.276872	-3.37554	-0.98067
58	H	2.380833	-2.49327	0.231033
59	H	5.004479	-1.29537	1.733989
60	H	3.590719	-2.00427	2.548468
61	H	5.210347	-2.6963	2.790622
62	H	3.689457	-5.23582	0.523721
63	H	2.823395	-4.43276	1.841933
64	H	4.499422	-4.97535	2.08245
65	H	5.574512	-4.00303	-0.40672
66	H	-1.49585	4.969686	0.924906

**2R, 3R, 2"R-2** Conf. 9		Standard Orientation (Ångstroms)		
I	atom	X	Y	Z
1	C	1.302339	-2.87392	-0.04928
2	C	0.567986	-4.04578	-0.28044
3	C	-0.81037	-3.97051	-0.42096
4	C	-1.47079	-2.70812	-0.31564
5	C	-0.68575	-1.54432	-0.11081
6	C	0.699641	-1.60073	0.027971
7	C	-2.90083	-2.60594	-0.37356
8	C	-3.51516	-1.23864	-0.09294
9	C	-2.57744	-0.15239	-0.64054
10	O	-1.25823	-0.30997	-0.04477
11	O	-3.6679	-3.55763	-0.62348
12	O	-4.77828	-1.13486	-0.7045
13	C	-3.03094	1.265315	-0.41573
14	C	-3.1866	1.813962	0.868119
15	C	-3.6108	3.133789	1.03857
16	C	-3.89172	3.928132	-0.07605
17	C	-3.74841	3.401283	-1.36338
18	C	-3.32439	2.086157	-1.51265
19	O	-4.31562	5.221789	0.033547
20	O	-2.96839	1.021178	1.973884
21	C	-1.8355	1.365342	2.770386
22	C	1.538093	-0.35712	0.182015
23	C	3.016848	-0.62616	-0.14764
24	C	3.48578	-1.94167	0.526648
25	O	2.640753	-3.03936	0.051057
26	C	4.883565	-2.36545	0.071059
27	C	3.398217	-1.92492	2.058778
28	C	3.910772	0.586272	0.178862
29	C	3.564722	1.841387	-0.63754
30	C	4.627497	2.965502	-0.57392
31	C	4.205834	4.133145	-1.47873
32	C	4.865438	3.459048	0.856173
33	O	5.90453	2.461892	-1.00801
34	O	-1.51252	-5.09494	-0.63387
35	H	1.077152	-4.99972	-0.34552
36	H	-3.5848	-1.11946	0.999942
37	H	-2.47555	-0.32076	-1.72161
38	H	-5.06553	-2.05941	-0.8391
39	H	-3.74597	3.521934	2.04559
40	H	-3.97429	4.025865	-2.22096
41	H	-3.21664	1.67793	-2.51418

42	H	-4.39794	5.4509	0.972295
43	H	-0.91048	1.259689	2.191819
44	H	-1.90834	2.389074	3.159547
45	H	-1.82773	0.664325	3.607936
46	H	1.14181	0.425359	-0.47321
47	H	1.454957	0.046252	1.203345
48	H	3.087422	-0.81447	-1.22987
49	H	4.96003	-2.34397	-1.02111
50	H	5.65304	-1.71094	0.489307
51	H	5.083132	-3.38749	0.407349
52	H	4.058874	-1.16067	2.480514
53	H	2.37904	-1.72785	2.404415
54	H	3.704897	-2.89823	2.454497
55	H	4.959621	0.343416	-0.01989
56	H	3.835979	0.813426	1.250097
57	H	2.598046	2.255454	-0.32369
58	H	3.442825	1.556959	-1.69498
59	H	3.262402	4.580192	-1.1451
60	H	4.977963	4.909412	-1.47704
61	H	4.06314	3.794472	-2.51371
62	H	3.93457	3.826144	1.302217
63	H	5.595778	4.274462	0.853589
64	H	5.2621	2.656971	1.485232
65	H	5.817254	2.220381	-1.9446
66	H	-2.46586	-4.83654	-0.70672
**2R, 3R, 2"R-2** Conf. 10		Standard Orientation (Ångstroms)		
I	atom	X	Y	Z
1	C	1.266534	-2.64371	0.009766
2	C	0.602188	-3.85823	-0.21571
3	C	-0.7737	-3.85968	-0.39267
4	C	-1.50295	-2.6326	-0.33145
5	C	-0.78712	-1.42278	-0.13585
6	C	0.595823	-1.40301	0.039576
7	C	-2.9344	-2.60936	-0.41193
8	C	-3.62342	-1.27369	-0.15456
9	C	-2.74286	-0.14777	-0.71912
10	O	-1.42015	-0.22077	-0.11857
11	O	-3.6473	-3.60371	-0.65832
12	O	-4.89024	-1.24988	-0.76657
13	C	-3.27283	1.249026	-0.53717
14	C	-3.50611	1.821745	0.736196
15	C	-4.00974	3.117488	0.852137

16	C	-4.29443	3.860037	-0.2998
17	C	-4.07494	3.316952	-1.56506
18	C	-3.56487	2.022062	-1.65929
19	O	-4.78637	5.120125	-0.10741
20	O	-3.21197	1.040355	1.814187
21	C	-3.45093	1.554371	3.116238
22	C	1.362527	-0.11326	0.189413
23	C	2.859707	-0.31785	-0.09276
24	C	3.365888	-1.58354	0.648126
25	O	2.609198	-2.73616	0.156537
26	C	4.812418	-1.9333	0.296983
27	C	3.177503	-1.53316	2.171886
28	C	3.676161	0.966325	0.204385
29	C	4.715454	1.306804	-0.87656
30	C	5.756856	2.382849	-0.48627
31	C	6.780543	2.541763	-1.61381
32	C	5.108091	3.735001	-0.15687
33	O	6.524688	1.921857	0.644109
34	O	-1.41073	-5.0245	-0.59635
35	H	1.162678	-4.78481	-0.24354
36	H	-3.70159	-1.14485	0.9364
37	H	-2.62889	-0.33587	-1.79602
38	H	-5.11749	-2.19147	-0.89993
39	H	-4.19639	3.577385	1.814027
40	H	-4.29578	3.891396	-2.46168
41	H	-3.39374	1.595139	-2.64398
42	H	-4.95666	5.524802	-0.97221
43	H	-4.51158	1.792346	3.266479
44	H	-3.15532	0.762334	3.806345
45	H	-2.84693	2.449812	3.310157
46	H	0.958007	0.637745	-0.49919
47	H	1.223078	0.310181	1.195216
48	H	2.960313	-0.54858	-1.163
49	H	4.933494	-2.03226	-0.78639
50	H	5.502738	-1.16467	0.657828
51	H	5.085132	-2.88821	0.757593
52	H	3.753355	-0.71582	2.617503
53	H	2.126973	-1.40187	2.446342
54	H	3.525938	-2.47234	2.612503
55	H	4.17885	0.866459	1.17483
56	H	2.977386	1.805033	0.314143
57	H	4.191557	1.625453	-1.78871
58	H	5.276387	0.403416	-1.14027

59	H	6.294815	2.860879	-2.54238
60	H	7.534855	3.285898	-1.33875
61	H	7.291553	1.590886	-1.79737
62	H	5.878488	4.466987	0.107653
63	H	4.414094	3.654143	0.689352
64	H	4.541535	4.12343	-1.01098
65	H	5.981752	2.042069	1.438283
66	H	-2.37408	-4.8185	-0.6963
**2R, 3R, 2"R-2** Conf. 11		Standard Orientation (Ångstroms)		
I	atom	X	Y	Z
1	C	1.259372	-2.74679	-0.00097
2	C	0.564283	-3.93628	-0.26471
3	C	-0.80917	-3.89478	-0.45505
4	C	-1.50429	-2.6495	-0.37058
5	C	-0.75716	-1.46554	-0.13696
6	C	0.6235	-1.48914	0.055356
7	C	-2.93374	-2.58374	-0.46361
8	C	-3.58705	-1.23499	-0.18118
9	C	-2.66892	-0.12396	-0.71446
10	O	-1.3571	-0.2472	-0.0975
11	O	-3.67193	-3.55142	-0.73994
12	O	-4.84835	-1.16179	-0.80013
13	C	-3.15881	1.285147	-0.51598
14	C	-3.38917	1.842512	0.760712
15	C	-3.84984	3.156528	0.888741
16	C	-4.09204	3.927567	-0.25534
17	C	-3.87398	3.396494	-1.52578
18	C	-3.40897	2.088698	-1.63051
19	O	-4.54289	5.214524	-0.17513
20	O	-3.13719	1.03565	1.831479
21	C	-3.36475	1.537131	3.138944
22	C	1.42419	-0.22685	0.251461
23	C	2.919599	-0.46208	-0.01975
24	C	3.382145	-1.76308	0.684856
25	O	2.597603	-2.88028	0.155759
26	C	3.190094	-1.75381	2.20849
27	C	4.818017	-2.14314	0.323547
28	C	3.757402	0.797273	0.324057
29	C	4.757561	1.207716	-0.76765
30	C	5.449514	2.573495	-0.54079
31	C	6.271562	2.604469	0.751397
32	C	6.34046	2.911785	-1.74608

33	O	4.460723	3.606236	-0.37368
34	O	-1.47647	-5.03587	-0.69409
35	H	1.099147	-4.87717	-0.31091
36	H	-3.6689	-1.12819	0.91197
37	H	-2.54557	-0.29639	-1.79289
38	H	-5.09958	-2.09315	-0.95978
39	H	-4.02548	3.58941	1.868266
40	H	-4.06743	4.005797	-2.4016
41	H	-3.23766	1.66991	-2.61876
42	H	-4.66112	5.453962	0.757038
43	H	-3.10337	0.724323	3.818618
44	H	-2.72805	2.405571	3.352219
45	H	-4.41722	1.811085	3.288869
46	H	1.048061	0.555852	-0.4175
47	H	1.28594	0.170507	1.267827
48	H	3.025614	-0.66727	-1.09492
49	H	3.513186	-2.71377	2.623297
50	H	3.781968	-0.96261	2.679532
51	H	2.141523	-1.60501	2.481634
52	H	5.529314	-1.42628	0.744874
53	H	5.052307	-3.13446	0.724264
54	H	4.949559	-2.17354	-0.76272
55	H	4.284312	0.654761	1.274593
56	H	3.080702	1.64373	0.487463
57	H	4.223164	1.259585	-1.72954
58	H	5.529178	0.437268	-0.89698
59	H	7.034877	1.818496	0.747492
60	H	6.771117	3.573246	0.852765
61	H	5.630534	2.466917	1.626849
62	H	6.793339	3.899969	-1.6148
63	H	5.754106	2.925087	-2.67458
64	H	7.142878	2.175848	-1.8732
65	H	3.96199	3.667191	-1.20478
66	H	-2.43264	-4.80021	-0.7977

Functional		Solvent?		Basis Set	
mPW1PW91		PCM		6-311G(d,p)	
		DP4+	0.44%	99.56%	-
Nuclei	sp2?	Experimental	Isomer 1	Isomer 2	Isomer 3
C	x	164	171.3	171.3	
C	x	97.8	101.2	101.4	
C	x	162.5	169.3	169.2	
C	x	102.1	103.7	103.9	
C	x	161.6	167.8	167.9	
C	x	102.7	107.6	107.4	
C	x	199.3	202.9	202.6	
C		72.5	76.2	76.9	
C		79.7	84.0	82.2	
C	x	116.9	122.1	123.2	
C	x	160.8	166.4	166.2	
C	x	100.1	100.78	102.14	
C	x	161	166.10	165.71	
C	x	108.2	110.86	111.99	
C	x	130.9	137.08	136.24	
C		56	56.32	57.78	
C		22.9	25.33	24.94	
C		42.3	45.45	45.10	
C		81	85.94	85.74	
C		28	29.52	29.07	
C		20.5	21.52	22.11	
C		26.6	28.38	28.32	
C		42.5	44.40	43.45	
C		71.3	75.08	74.80	
C		29.2	32.84	28.83	
C		29	28.11867752	33.13018361	
H	x	5.87	6.105283818	6.108414366	
H		4.77	4.603482412	4.440172892	
H		5.42	5.242986509	5.44604967	
H	x	6.5	6.410685957	6.452346184	
H	x	6.46	6.788412393	6.876775404	
H	x	7.32	7.734094051	7.852474052	
H		3.82	3.752464122	3.74409614	
H		3.82	3.729889356	3.739969173	
H		3.82	4.005589621	4.007806081	
H		2.74	2.671733227	2.730714584	
H		2	1.975676729	1.946769127	
H		1.58	1.45944329	1.551149366	
H		1.43	1.573395956	1.474706658	
H		1.43	1.463281489	1.542922469	
H		1.43	1.31607356	1.28623984	
H		1.16	1.159767861	1.254452409	
H		1.16	1.250438765	1.130911332	
H		1.16	1.096560643	1.119552696	

H	1.1	1.879092748	1.887601078
H	1.71	1.115695669	1.054313722
H	1.35	1.645679865	1.582323468
H	1.66	1.093781523	1.227241868
H	1.16	1.191758478	1.028642863
H	1.16	1.218397911	1.334464964
H	1.16	1.114695794	1.004729942
H	1.16	0.898629251	1.223790924
H	1.16	1.073416337	1.207864264
H	1.16	1.316317948	1.190506289
H	1.16	0.162381344	0.403475758

Functional	Solvent?	Basis Set	Type o
mPW1PW91	PCM	6-311G(d,p)	Unscalec

	Isomer 1	Isomer 2	Isomer 3	Isomer 4	Isomer 5
sDP4+ (H data)	13.29%	86.71%	-	-	-
sDP4+ (C data)	5.89%	94.11%	-	-	-
sDP4+ (all data)	0.95%	99.05%	-	-	-
uDP4+ (H data)	61.93%	38.07%	-	-	-
uDP4+ (C data)	22.06%	77.94%	-	-	-
uDP4+ (all data)	31.52%	68.48%	-	-	-
DP4+ (H data)	19.96%	80.04%	-	-	-
DP4+ (C data)	1.74%	98.26%	-	-	-
DP4+ (all data)	0.44%	99.56%	-	-	-

Figure S22. DP4+ analysis of compound **2** with isomers **2R, 3R, 2"R-2** and **2R, 3R, 2"S-2**.

Specific Rotation		
	cal	exp
1-SR	-174.6	-86.4
1-SS	-267.7	
2-RRR	-192.8	+24.2
2-RRS	+42.4	

Figure S23. Caculated and experimental optical rotation values of Compounds 1 and 2
